# An International Expert Survey on the Indications and Practice of Radical Thoracic Reirradiation for Non-Small Cell Lung Cancer

**DOI:** 10.1016/j.adro.2021.100653

**Published:** 2021-01-20

**Authors:** Robert Rulach, David Ball, Kevin L.M. Chua, Max Dahele, Dirk De Ruysscher, Kevin Franks, Daniel Gomez, Matthias Guckenberger, Gerard G. Hanna, Alexander V. Louie, Drew Moghanaki, David A. Palma, Clive Peedell, Ahmed Salem, Shankar Siva, Gregory M.M. Videtic, Anthony J. Chalmers, Stephen Harrow

**Affiliations:** a Institute of Cancer Sciences, The University of Glasgow, Glasgow, UK; b Department of Clinical Oncology, The Beatson West of Scotland Cancer Centre, Glasgow, UK; c Sir Peter MacCallum Department of Oncology, The University of Melbourne, Parkville, Victoria, Australia; d Division of Radiation Oncology, National Cancer Centre, Singapore; e Department of Radiation Oncology, Amsterdam University Medical Centre, Amsterdam, The Netherlands; f Department of Radiation Oncology (Maastro), Maastricht University Medical Centre, GROW School of Oncology, Maastricht, The Netherlands; g Department of Clinical Oncology, St James’s Institute of Oncology, Leeds Cancer Centre, Leeds, UK; h Department of Radiation Oncology, Memorial Sloan Kettering Cancer Center, New York, USA; i Department of Radiation Oncology, University Hospital Zurich, University of Zurich, Zurich, Switzerland; j Department of Radiation Oncology, Sunnybrook Health Sciences Center, Toronto, Canada; k Department of Radiation Oncology, Atlanta VA Health Care System, Decatur, Georgia, USA; l Winship Cancer Institute of Emory University, Atlanta, Georgia, USA; m Division of Radiation Oncology, Western University, London, Canada; n Department of Clinical Oncology, James Cook University Hospital, Middlesbrough, UK; o Department of Clinical Oncology, The Christie NHS Foundation, Manchester, UK; p Division of Cancer Sciences, University of Manchester, UK; q Department of Radiation Oncology, Taussig Cancer Institute, Cleveland Clinic, Cleveland, Ohio, USA

## Abstract

**Purpose:**

Thoracic reirradiation for non-small cell lung cancer with curative intent is potentially associated with severe toxicity. There are limited prospective data on the best method to deliver this treatment. We sought to develop expert consensus guidance on the safe practice of treating non-small cell lung cancer with radiation therapy in the setting of prior thoracic irradiation.

**Methods and Materials:**

Twenty-one thoracic radiation oncologists were invited to participate in an international Delphi consensus process. Guideline statements were developed and refined during 4 rounds on the definition of reirradiation, selection of appropriate patients, pretreatment assessments, planning of radiation therapy, and cumulative dose constraints. Consensus was achieved once ≥75% of respondents agreed with a statement. Statements that did not reach consensus in the initial survey rounds were revised based on respondents’ comments and re-presented in subsequent rounds.

**Results:**

Fifteen radiation oncologists participated in the 4 surveys between September 2019 and March 2020. The first 3 rounds had a 100% response rate, and the final round was completed by 93% of participants. Thirty-three out of 77 statements across all rounds achieved consensus. Key recommendations are as follows: (1) appropriate patients should have a good performance status and can have locally relapsed disease or second primary cancers, and there are no absolute lung function values that preclude reirradiation; (2) a full diagnostic workup should be performed in patients with suspected local recurrence and; (3) any reirradiation should be delivered using optimal image guidance and highly conformal techniques. In addition, consensus cumulative dose for the organs at risk in the thorax are described.

**Conclusions:**

These consensus statements provide practical guidance on appropriate patient selection for reirradiation, appropriate radiation therapy techniques, and cumulative dose constraints.

## Introduction

Curative-intent thoracic reirradiation is a second or subsequent course of radiation therapy to the chest with the goal of long-term disease control. Repeat irradiation can be used in several different clinical scenarios: for locally recurrent lung tumors, a metachronous lung tumor distant from a previously irradiated lung tumor, a new lung tumor arising in the previous radiation therapy field of a different histologic tumor, or to metastatic disease overlapping with a previous treatment. Each scenario will have different underpinning tumor biology, outcomes, and toxicities.

High-level evidence to guide practice of radical thoracic reirradiation is lacking. The majority of the studies of thoracic reirradiation are retrospective reports, group different clinical situations together, and lack consistent reirradiation doses or techniques. Given this heterogeneity, the reported efficacy of high-dose re-treatment strategies vary widely with a median overall survival (OS) between 11.1 and 24 months.^1^

In the setting of non-small cell lung cancer (NSCLC), local relapse occurs in a third of patients 2 years after radical radiation therapy, and the rate of second primary lung cancers at 10 years is 14%.^2^, ^3^, ^4^ We estimate 700 patients with NSCLC annually in the United Kingdom would develop either a new primary or local recurrence (based on UK audit data), for whom reirradiation may be a treatment option.^5^ The number of patients being treated with reirradiation is increasing.^6^ This is due to several factors. The improved use of computed tomography (CT) already results in increased detection of new primary tumors. Relapsed disease will become more commonly diagnosed as recent follow-up recommendations after radical radiation therapy for NSCLC recommend frequent surveillance CT scans.^7^ Furthermore, current radiation therapy technology allows greater normal tissue sparing, thus making reirradiation feasible. Nevertheless, reirradiation is a complex and potentially harmful treatment, with a 5.2% to 23% risk of grade 3 pneumonitis and grade 5 toxicity rate up to 20% depending on technique and tumor location.^8^

No formal guidelines exist on the selection of appropriate patients for curative-intent reirradiation or on relevant cumulative dose constraints and planning/treatment techniques. In addition, there is a lack of contemporary clinical studies, with the last prospective trial of thoracic reirradiation published in 2003.^9^ As reirradiation is becoming more common, there is a need to share current practice, identify areas of uncertainty, and develop ongoing research questions. An international Delphi process was therefore conducted to develop consensus statements on the definition of reirradiation, patient eligibility, radiation therapy planning technique, and cumulative dose constraints. The scope of the statements was limited to NSCLC because this is the most common tumor for which curative-intent reirradiation is considered.

## Methods and Materials

### Participant selection

Thoracic radiation oncologists who have published articles about reirradiation were contacted by e-mail to participate in a series of questionnaires regarding their reirradiation practice. If they were unable to participate in the survey, they were able to nominate another radiation oncologist familiar with lung reirradiation to participate. We invited 21 clinicians from North America, Europe, Asia, and Australia to take part in this process.

### Ethics and consent

Ethics approval was waived by the University of Glasgow Ethics Committee and the West of Scotland Research Ethics Committee. All participants consented to the Delphi process.

### Questionnaires

The Delphi consensus method was selected as an unbiased approach to obtain anonymized responses over a wide geographic distance.^10^ Questionnaires were created using a survey website (webprolol.com). Four rounds of questionnaires took place. Consensus was deemed to be reached once ≥75% of respondents strongly agreed or agreed with a statement. Statements that did not reach consensus in the initial survey rounds were revised by the study organizers (RR/SH) based on respondents’ comments and presented in subsequent rounds. The study organizers did not take part in any of the surveys.

The first round captured the clinicians’ reirradiation practice using mostly open-ended questions. The survey was divided into 6 sections (a total of 36 questions): definition of reirradiation, patient selection and pretreatment assessment, reirradiation planning techniques, cumulative dose constraints, expected toxicity of treatment, and follow-up after reirradiation (Appendix 1, p 1-49). The study organizers reviewed all of the responses, identified common themes, and produced a series of statements based on these answers. Where questions involved numerical values (e.g., minimum lung function test values for reirradiation), the median value of the answers was used in the subsequent statement.

The second round featured 57 statements (Appendix 1, p 50-126), and respondents rated each statement using a 5-point Likert scale (strongly agree, agree, neutral, disagree, strongly disagree). For each statement, there was a free text box for the participants to explain why they agreed or disagreed with a statement and provide links to further information (e.g. pertinent publications).

The comments and additional evidence suggested from round 2 were used to refine the statements that did not reach consensus. The third round consisted of 19 modified statements to be rated using the Likert scale, again with opportunity to comment on them (Appendix 1, p 127-166). The questions relating to expected toxicity of treatment were removed after round 2 because participants commented that reirradiation is a highly individualized treatment and the expected toxicity may vary depending on many factors, and therefore it would be impossible to provide general guidance regarding this.

The fourth round was a single question on the definition of thoracic reirradiation (Appendix 1, p 167-170). Two different definitions of reirradiation were presented and clinicians were asked to choose one, both, or neither. The results of the previous 2 rounds were provided with justification to illustrate how the definition was amended.

### Reporting

The results are presented in 5 sections, with corresponding tables describing the statements and level of agreement. We also describe statements that did not reach consensus; they are areas of controversy where further research may be required. For items without consensus, the statement with the highest degree of agreement from any round is included in the results.

## Results

Fifteen of 21 radiation oncologists from 7 countries agreed to participate in this study. Countries represented were the United Kingdom (3), United States (3), Australia (3), Canada (2), the Netherlands (2), Switzerland (1), and Singapore (1). The 15 participants have a total lung radiation therapy experience of 222 years (median of 12 years each; range, 7- 34 years) and have authored 44 articles about reirradiation or related topics. Additional details are found in Appendix 2. The first round opened on September 23, 2019, and the final survey was on March 2, 2020. Rounds 1 to 3 had a 100% response rate, and the final round had a response rate of 14 of 15 (93.3%).

**Figure 1:**
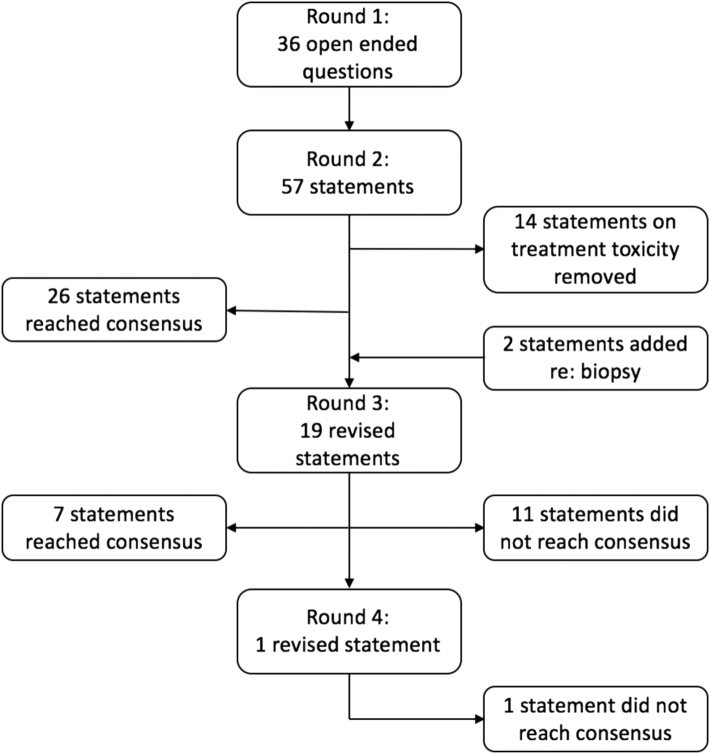
Schema of the consensus building process.

Fifty-seven statements were created after the first round of the Delphi process, and consensus was reached in 26 statements (45.6%). After the second round, 14 statements were removed regarding expected toxicity of treatment because the toxicity rates would depend on each individualized treatment plan; therefore, it would be impossible to form generalized statements. The third round consisted of 19 statements, 7 of which reached consensus (36.8%). Two additional statements were added in the third round to clarify the need for biopsy in reirradiation. The final round was one question on the definition of reirradiation, and consensus was not reached. This process is summarized in Figure 1. A table of all the statements for which consensus was achieved and the highest level of agreement statements where consensus was not reached is presented in Appendix 3.

### Definition of reirradiation

Despite 3 survey rounds to define reirradiation in NSCLC, consensus was not achieved. The highest amount of support was for the following round 2 definition: “any dose of radical radiation for lung cancer, after initial radical radiotherapy to the thorax or surrounding tissues for any tumor histology, provided there is any overlap of previous dose in either the planning target volume (PTV) or the organs-at-risk (OARs)” (67% agreed). The respondents’ reasons for why they disagreed with this definition were focused on the definition of overlap of OARs or PTV. “Any overlap” would include low-dose regions, which may be large and have little contribution to toxicity. In addition, if 1 lesion were in the lung apex and a second lesion in the lung base, there might be no dosimetric overlap at all, but a significant volume of lung would be treated. Sixty percent of respondents disagreed with setting an overlap isodose level that would be considered significant (e.g., various articles have used the 50% isodose level) because there are no data to support it.

A suggested solution to this issue was to consider a treatment to be reirradiation (as opposed to a second radiation therapy to the lungs with no overlap) only if the cumulative dose to either the OARs or PTV would exceed the normal tissue dose constraints/prescribed dose to the PTV that would be allowed for a single course of radiation therapy with no correction for recovery. This option was presented in round 3 but only achieved 60% consensus. The lack of support for this definition centered on the issue that overlap was important for toxicity in serial organs within the thorax. However, overlap in parallel organs would be less significant because the total volume of the organ irradiated may be of greater significance. The round 3 definition would not account for toxicity to parallel organs. Defining a volumetric constraint (e.g. lifetime maximum irradiated volume of lung) would be challenging due to the lack of data and the likely significant variability among patients.

An alternative suggestion was to divide reirradiation into 2 categories: (1) type I reirradiation for the local relapse of NSCLC (ie, “salvage reirradiation”), with likely a high degree of OAR dose overlap in serial organs, and (2) type II reirradiation for a new primary NSCLC, with little or no overlap between the 2 courses of radiation therapy but an increased volume of lung irradiated. Two versions of this concept were voted on in the fourth round of the survey. In addition, 71.4% of respondents were in favor of dividing reirradiation into treatment of local relapse and new primary cancers but could not converge on how to limit the definition to the proximity of the original PTV. In addition, the 2 categories described would not account for the reirradiation of metastases.

### Patient selection and pretreatment assessments

Statements regarding patient selection and pretreatment assessments that reached consensus are presented in Table 1. There was agreement that reirradiation could be considered in new primary NSCLC, local relapse (provided no central overlap), and nodal recurrence (in a previously untreated area). Central overlap is defined as the tumor being within 2 cm of the trachea, bronchi, or proximal bronchial tree or the PTV abutting the mediastinal pleura or pericardium as per the Radiation Therapy Oncology Group (RTOG) 0236 trial.^11^

**Table 1:** Consensus statements regarding suitable patients and pretreatment assessment

	Consensus agreed	SA/A, %	N, %	D/SD, %	Round agreed	Median
1.1	Radical reirradiation can be considered for suspected new lung primaries with minimal overlap with previous radiation therapy fields.	93	7	0	R2	SA
1.2	Radical reirradiation can be considered for lung tumors that develop new nodal disease after an initial course of radiation therapy only to the primary tumor (therefore minimal overlap).	100	0	0	R2	SA
1.3	Radical reirradiation can be considered where a lung tumor relapses locally (or develops a suspected second primary tumor with >50% overlap with the original primary tumor), but low overlap with serial structures in the thorax.	93	0	7	R2	SA
1.4	Alternative treatments (e.g., systemic therapy) are preferred to radical reirradiation to the primary lung cancer where the lung tumors have relapsed both locally and with widespread metastatic disease.	93	7	0	R2	A
1.5	In general, patients should have an ECOG PS of 0-2 to be considered for radical dose reirradiation, with exceptions being made for selected PS 3 patients (e.g., SABR reirradiation, or PS 3 due to nonrespiratory issues).	93	0	7	R2	SA
1.6	Reirradiation should be avoided in patients with interstitial lung disease.	86	7	7	R2	SA
1.7	Reirradiation should be performed cautiously with patients who developed grade 3 or higher toxicity with their initial radiation treatment.	86	7	7	R2	A
1.8	Surgery should be considered in all appropriate patients being assessed for reirradiation.	93	0	7	R2	A
1.9	In locally advanced recurrent lung cancer, where there is an increased likelihood of response to immunotherapy (e.g., PD-L1 >50%), immunotherapy may be preferable to high-risk radical reirradiation.	80	0	20	R2	A
1.10	In locally advanced recurrent lung cancer, where there is an actionable mutation (e.g., EGFR mutation, ALK fusion), targeted treatment may be preferable to high-risk radical reirradiation.	79	7	14	R2	A
1.11	Investigations before commencing radical reirradiation are whole body PET-CT, CT chest + contrast, and CT/MRI brain.	>93	-	-	R2	Essential
1.12	Consideration for biopsy must be made in a tumor board/multidisciplinary team meeting before considering radical reirradiation.	86.6	6.7	6.7	R3	SA
1.13	Reirradiation can be considered where the tumor board/multidisciplinary team agrees that there is a high likelihood of cancer, but despite best efforts, histologic confirmation of cancer is not possible.	86.6	6.7	6.7	R3	SA
1.14	For conventionally fractionated reirradiation, the clinician must consider re-treatment to have a positive risk/benefit ratio considering the current pulmonary function tests and the likely exposure of the lung to reirradiation, with no minimum PFTs values applicable.	86.6	6.7	6.7	R3	A
1.15	For reirradiation with SABR, no minimum PFTs apply.	87	0	13	R2	A

*Abbreviations*: ALK = anaplastic lymphoma kinase; CT = computed tomography; D/SD = disagree/strongly disagree; ECOG = Eastern Cooperative Oncology Group; EGFR = epidermal growth factor receptor; MRI = magnetic resonance imaging; N = neutral; PET-CT = positron emission computed tomography; PFT = pulmonary function test; PD-L1 = programmed death-ligand 1; PS = performance status; R2 = round 2; R3 = round 3; SA/A = strongly agree/agree.

Systemic treatment should be considered in metastatic patients. Patients should have an Eastern Cooperative Oncology Group performance status of 0 to 2 (with some exceptions), no interstitial lung disease, and be staged with computed tomography (CT) of the chest, whole body positron emission tomography-CT (PET-CT), and magnetic resonance imaging (MRI) or CT of the head. Minimum pulmonary function test (PFT) values for conventionally fractionated reirradiation could not be agreed on in the second round, with consensus being reached on using an individualized assessment of the potential risks and benefits rather than using threshold values, which have sparse supporting evidence.

A minimum interval between initial treatment and reirradiation was investigated in the second and third rounds, but consensus was not met. Twenty percent of participants did not support any minimum time interval between courses of radiation. However, most clinicians agreed with a minimum of 6 months (73.3%), and a minority would not reirradiate unless the interval was greater than 12 months (6.7%).

The survey section about expected risks of toxicity was removed because the respondents felt unable to fully answer the questions without patient input. Although not a consensus statement, several respondents noted that reirradiation is often a highly individualized treatment, and understanding each patient’s acceptance of the risk of side effects (including death), weighed against the possible benefits of reirradiation or alternative therapies, is crucial before embarking on treatment.

### Planning and delivery of reirradiation

Statements that reached consensus on radiation therapy planning are shown in Table 2. There was agreement that highly conformal treatments should be used for reirradiation, with preference for SABR when the reirradiation target is small with minimal overlap with previously treated OARs. In the event that an OAR dose constraint would be exceeded due to being in the reirradiation PTV, there was agreement that meeting the OAR dose constraint should take priority. The minimum expansion from gross tumor volume to clinical target volume did not reach consensus, with 66.7% agreeing to a minimum expansion of 5 mm.

**Table 2:** Consensus statements regarding radiation therapy planning technique

	Consensus agreed	SA/A, %	N, %	D/SD, %	Round agreed	Median
2.1	When combining initial and reirradiation plans, either rigid or deformable dose registration are acceptable methods (although there are considerable uncertainties in either process, and additional investigation is warranted).	80	6	14	R2	SA
2.2	18-FDG-PET/CT is recommended to aid tumor volume delineation.	86	7	7	R2	SA
2.3	When contouring for conventionally fractionated radical reirradiation, an acceptable minimum expansion from CTV to PTV is 5 mm (or follow institutional guidelines where available).	86	7	7	R2	A
2.4	PTV coverage can be compromised to achieve acceptable OAR doses.	80	6	14	R2	SA
2.5	Radical reirradiation should be performed using highly conformal radiation therapy techniques (e.g., VMAT, tomotherapy, CyberKnife).	100	0	0	R3	SA
2.6	SABR is the preferred reirradiation technique where the tumor is not ultracentral, the tumor volume is small, and there is minimal overlap with OARs.	80	13.3	6.7	R2	SA
2.7	Protons may have a role for reirradiation and requires further evaluation in the context of a clinical trial.	80	20	0	R3	A
2.8	Acceptable doses for conventionally fractionated radical thoracic reirradiation are 60 Gy in 30 fractions or 55 Gy in 20 fractions once daily for non-small cell lung cancer.	93	0	7	R2	A
2.9	Daily cone beam CT is recommended for treatment verification for conventionally fractionated reirradiation.	100	0	0	R2	SA
2.10	Any dose and fractionation that can safely deliver a BED >100 Gy to the tumor is acceptable for radical reirradiation with SABR.	86.7	0	13.3	R3	A
2.11	Daily cone beam CT is recommended for treatment verification for SABR reirradiation.	100	0	0	R2	SA

*Abbreviations*: BED = biologically effective dose; CT = computed tomography; CTV = clinical target volume; D/SD = disagree/strongly disagree; N = neutral; OAR = organ at risk; PTV = planning target volume; R2 = round 2; R3 = round 3; SA/A = strongly agree/agree; VMAT = volumetric arc therapy; 18-FDG-PET/CT = 18-fluorodeoxyglucose positron emission tomography/computed tomography.

### Cumulative dose constraints

Table 3 summarizes the agreed cumulative dose constraints for OARs in the thorax, based on several studies.^12^, ^13^, ^14^, ^15^, ^16^ On the basis of comments from the first round, to allow addition of different dose and fractionations, dose constraints were expressed as a total equieffective dose in 2 Gy/fraction (EQD2) with no adjustment for potential recovery.

**Table 3:** Consensus statements regarding cumulative dose constraints

	Consensus agreed	SA/A, %	N, %	D/SD, %	Round agreed	Median
3.1	There is insufficient evidence to suggest volumetric cumulative dose constraints for the lung due to the changes in anatomy and function of the lung after an initial course of radiation therapy.	80	13.3	6.7	R3	A
3.2	For radical reirradiation, the desirable cumulative maximum point dose constraint to the esophagus is an EQD2 of 75 Gy, although up to 100 Gy is acceptable (using an α/β = 3); the volume of the esophagus getting 55 GY should be less than 35% (V55Gy <35%).^12^	86	7	7	R2	A
3.3	For radical reirradiation, the desirable cumulative maximum point dose constraint to the spinal cord is an EQD2 of 60 Gy (using α/β = 2), with a maximum EQD2 of 67.5 Gy (provided that the initial irradiation dose to the cord did not exceed 50 Gy).^13^	80	13	7	R2	A
3.4	For radical reirradiation, the desirable cumulative maximum dose (D_max_) constraint to the brachial plexus is an EQD2 of 80Gy (α/β = 2) and an acceptable cumulative D_max_ is 95 Gy (if the interval between treatments is >2 years).^14^	80	0	20	R2	A
3.5	For radical reirradiation, the desirable cumulative maximum dose (D_max_) constraint to the aorta is an EQD2 of 115 Gy (α/β = 3). The desirable cumulative D_max_ to the pulmonary artery is an EQD2 of 110 Gy.^15^^,^^16^	80	0	20	R2	A
3.6	There is a lack of information to guide reirradiation dose constraints for the skin and the heart, therefore the use of other guidelines (e.g., QUANTEC or SABR guidelines) and to keep the dose to these organs as low as reasonably achievable are recommended.	100	0	0	R2	A

*Abbreviations:* D/SD = disagree/strongly disagree; EQD2 = equieffective dose in 2 Gy/fraction; N = neutral; QUANTEC = quantitative analyses of normal tissue effects in the clinic; R2 = round 2; R3 = round 3; SA/A = strongly agree/agree.

No consensus was reached on the dose constraint for the proximal bronchial tree. Suggested values for a desired D_max_ EQD2 of <80 Gy with an absolute maximum dose of 105 Gy (α/β = 3) reached 66.7% agreement, based on 2 references.^15^^,^^17^

### Follow-up after radiation therapy

In patients who are fit for further treatment after radical reirradiation, surveillance CT is recommended every 3 to 6 months for the first 2 years and every 6 to 12 months thereafter (86% consensus).

## Discussion

### Summary of results

These statements from an international collaboration of thoracic radiation oncologists provide consensus-based recommendations for curative-intent thoracic reirradiation. This information on patient selection, staging, cumulative dose constraints, and radiation therapy techniques can be used to aid clinicians’ decision making and reduce toxicity from treatment.

### Definition of reirradiation

Thoracic reirradiation may be suitable for a very heterogeneous patient group, and the selection of appropriate patients is highly individualized. Therefore, the development of a single definition for all thoracic reirradiation was difficult. The problems encountered in this Delphi process stemmed from the lack of data on how much overlap would be significant in serial OARs, whether large-volume low-dose reirradiation of the lungs has a significant effect, and how to account for metastatic disease. A majority of clinicians (71.4%) agreed with dividing NSCLC local relapse and new primaries into different entities, but there remains a lack of robust evidence to suggest that efficacy or toxicity is different in either group.

### Patient selection and pretreatment assessments

Minimum PFT values for conventionally fractionated radiation therapy and the need for a tumor biopsy required further clarification after the second round. PFTs provide an objective measure of respiratory reserve. However, the panel did not agree on minimum values for forced expiratory volume in 1 second or diffusing capacity. Several of the panel commented on a threshold diffusing capacity and forced expiratory volume in 1 second of 30% to 40%. However, setting an absolute value was thought to be unwise because other variables, such as the change in lung function from the first to the second irradiation and the site and volume of disease, also need to be considered.

The need for obtaining a biopsy was agreed in the third round as important to consider. If the biopsy was not possible, reirradiation could still be given with agreement of the tumor board. This is a pragmatic approach because often in areas that have been irradiated, the risks of biopsy may be higher and what tissue is retrieved may be nondiagnostic.

One area that was not fully considered in the surveys was how to account for the efficacy and toxicity of the initial radiation therapy. Patients are unlikely to derive benefit from a second course of radiation if they progress shortly after their first. This is supported by retrospective data that show the longer the interval between treatments, the longer the OS after reirradiation.^18^ The interval between treatments may describe tumor behavior, with slower-growing tumors potentially after a less aggressive clinical course. In addition, longer intervals allow for more normal tissue recovery, reducing the risk of high-grade toxicity. The majority of our group suggest a minimum interval of ≥6 months. If grade 3 or above toxicity occurred in the initial radiation therapy, then reirradiation may cause more severe side effects. This effect is seen in rat reirradiation models but unproven in clinical practice.^19^ We suggest that reirradiation be used cautiously considered in this instance.

The panel of thoracic radiation oncologists agreed that it is appropriate to treat targets that were not previously irradiated (nodal relapse, second primary NSCLC), as well as areas of local recurrence within previously irradiated areas. Local recurrence was considered to represent radioresistant disease, which may be less responsive to re-treatment. However, reirradiation may also be useful in this instance to delay need for systemic treatment or prevent serious local complications. In addition, reirradiation could be delivered in a way to overcome radioresistance (e.g. using high doses per fraction as in SABR). Reirradiation using SABR has a promising local control rate (1-year local control of 65%-95%), with a grade 3 pneumonitis rate of 2% to 30%.^20^ A review of using SABR as a salvage treatment after radical radiation therapy described a 2-year OS of 37% to 79% with acceptable toxicities, suggesting this approach may be reasonable.^21^

### Planning and delivery of reirradiation

Reirradiation was considered using conventional fractionation, intermediate hypofractionation, and ablative dose schedules. SABR was recommended in non-ultra-central, small-volume disease. Ultra-central is defined as when the PTV overlaps either the main bronchi or trachea.^22^ The potential benefits of SABR in reirradiation have already been described. Additionally, the highly conformal dose distributions and steep gradients associated with SABR reduce the cumulative dose to OARs compared with conventionally fractionated treatments. Tumor location when considering reirradiation is important. The use of both SABR or conventionally fractionated reirradiation for central local relapse is high risk.^23^, ^24^, ^25^ As the use of SABR has evolved, it is recognized that ultra-central lesions are more likely to have serious side effects than central disease, with a grade 5 toxicity rate of up to 21%.^22^^,^^26^ Therefore, reirradiation using SABR for ultra-central disease is not recommended. Provided the tumor volume is suitable and that OARs can be avoided, SABR may be appropriate for moderately central disease (ie, centrally located, but not ultra-central). For peripheral reirradiation, SABR is preferred.

Protons may provide another way to reduce cumulative OAR dose. A planning study comparing reirradiation with intensity modulated proton therapy and volumetric arc therapy suggested that intensity modulated proton therapy was associated with a statistically significant reduction in dose to the spinal cord, lungs, and heart and a trend for reduced dose in the other thoracic OARs.^27^ Two prospective registry studies of proton reirradiation reported a grade 3 toxicity rate between 7% and 42% and a grade 5 toxicity rate of 3.8% to 10.5%.^28^^,^^29^ Our group suggested investigating this further with a clinical trial.

### Cumulative dose constraints

Cumulative dose constraints are crucial for the safe practice of reirradiation. These have been difficult to establish due to the lack of both preclinical evidence about normal tissue recovery and dose/toxicity data. Radiobiologically, a degree of normal tissue recovery takes place after the first course of radiation therapy, but there is no data in humans as to how much tolerance is regained. Recent publications from Paradis et al*,* Troost et al, and the American Radium Society suggest cumulative reirradiation OAR dose constraints and are summarized in Table 4.^6^^,^^27^^,^^30^

**Table 4:** A comparison of putative cumulative dose constraints

OAR	α/β	This study (EQD2)	Paradis et al (EQD2)∗^6^	Troost et al (EQD2, 9-mo interval)^27^	American Radium Society (EQD2)†
Spinal cord	2	D_max_ 60 Gy	D0.1cc <56.25 Gy	D_max_ <65 Gy	D_max_ <57 Gy
Esophagus	3	D_max_ 75-100 Gy	D0.1cc <90.6 Gy	D_max_ <100 Gy	V60 <40%, D_max_ <100-110 Gy
Brachial plexus	2	D_max_ 80-95 Gy	D0.1cc <85 Gy	D_max_ <85 Gy	D_max_ <85 Gy
Great vessels	3	D_max_ 110 – 115 Gy	D0.1cc <123 Gy	D_max_ <110 Gy	D_max_ <120 Gy
PBT	3	D_max_ <80-105 Gy‡	D0.1cc <90.6 Gy	D_max_ <110 Gy	D_max_ <110 Gy
Skin/Chest wall	2.5	ALARA	D0.1cc <105 Gy	n/a	n/a
Heart	2.5	ALARA	D0.1cc <85 Gy	D_mean_ <70 Gy	V40<50%
Lung	3	Individualized	Individualized	D_mean_ <22 Gy	V20<40%

*Abbreviations:* ALARA = as low as reasonably achievable; EQD2 = equieffective dose in 2 Gy fractions; OAR = organs at risk; PBT = proximal bronchial tree.

∗ Dose constraints converted from α/β ratio of 2.5 to the stated α/β ratios in the table to allow ease of comparison; dose constraints derived using a 6- to 12-month interval, with OARs being treated to tolerance in the first treatment.

† Dose constraints α/β ratios not quoted in the American Radium Society abstract.

‡ Consensus not reached.

In this study, cumulative lung reirradiation dose constraints were not agreed on, with respondents citing lack of evidence for traditional V5Gy and V20Gy limits in this setting. An alternate approach for reirradiation dose constraints for parallel OARs is to use a critical volume-dose (CV) constraint.^31^ This approach has been used in the RTOG 0915 and 0813 trials for lung SABR to ensure a given volume of lung receives less than a threshold dose.^32^^,^^33^ For example, the RTOG 0813 protocol mandates that 1500 cm^3^ of lung should receive less than 12.5 Gy. The principle underlying this constraint is to preserve a minimum volume of functional lung. This was not suggested by the respondents but may be a useful approach in the reirradiation setting and could be adopted in future reirradiation trial designs.

There is a pressing need for prospective data collection of cumulative OAR doses and associated toxicity to refine these estimates. There will be inaccuracies in this process (e.g., registration of dose to OARs from the initial radiation therapy to the reirradiation plan, actual dose delivered to the OAR may be different from the initial plan due to differences in position). Despite this, it will provide some initial validation of the suggested constraints.

### Strengths and limitations

This Delphi consensus process uses international expert opinion to generate contemporary thoracic reirradiation recommendations. The paucity of prospective trials in this area and the wide range of clinical scenarios that reirradiation can be considered for limits the strength of the recommendations that can be made. The selection process of participants initially focused on whether they had publications on reirradiation. This excludes unpublished clinicians with extensive clinical experience of reirradiation. In addition, as it would be impractical to invite all authors with articles related to reirradiation, an inherent limitation of this study is that the participants are a selected group of clinicians. Therefore, it is likely there will be alternate opinions on the statements presented here. Nevertheless, as reirradiation is a nonstandard treatment, guidance in how to identify suitable patients and perform safe reirradiation is useful.

## Conclusions

The key recommendations of this consensus are that a full diagnostic workup should be performed in patients with suspected local recurrence; curative intent treatment such as radical reirradiation or surgery should be considered for localized recurrence; any reirradiation should be delivered using optimal image guidance and highly conformal techniques; and prospective registries and clinical trial data are urgently needed.
