# Supplementary material for: An International Expert Survey on the Indications and Practice of Radical Thoracic Reirradiation for Non-Small Cell Lung Cancer
Source: Adv Radiat Oncol. 2021 Jan 20;6(2):100653. doi: 10.1016/j.adro.2021.100653 (PMC8022147; doi:10.1016/j.adro.2021.100653)
Supplement: Appendix 1 [file mmc1.pdf]

Supplementary Data C

Round 1

An international Delphi consensus on the use of radical thoracic re-irradiation and acceptable cumulative dose constraints

Total number of respondents: 15

1. Please enter your identification number (found on the e-mail with the link to this survey)

Number of respondents: 15

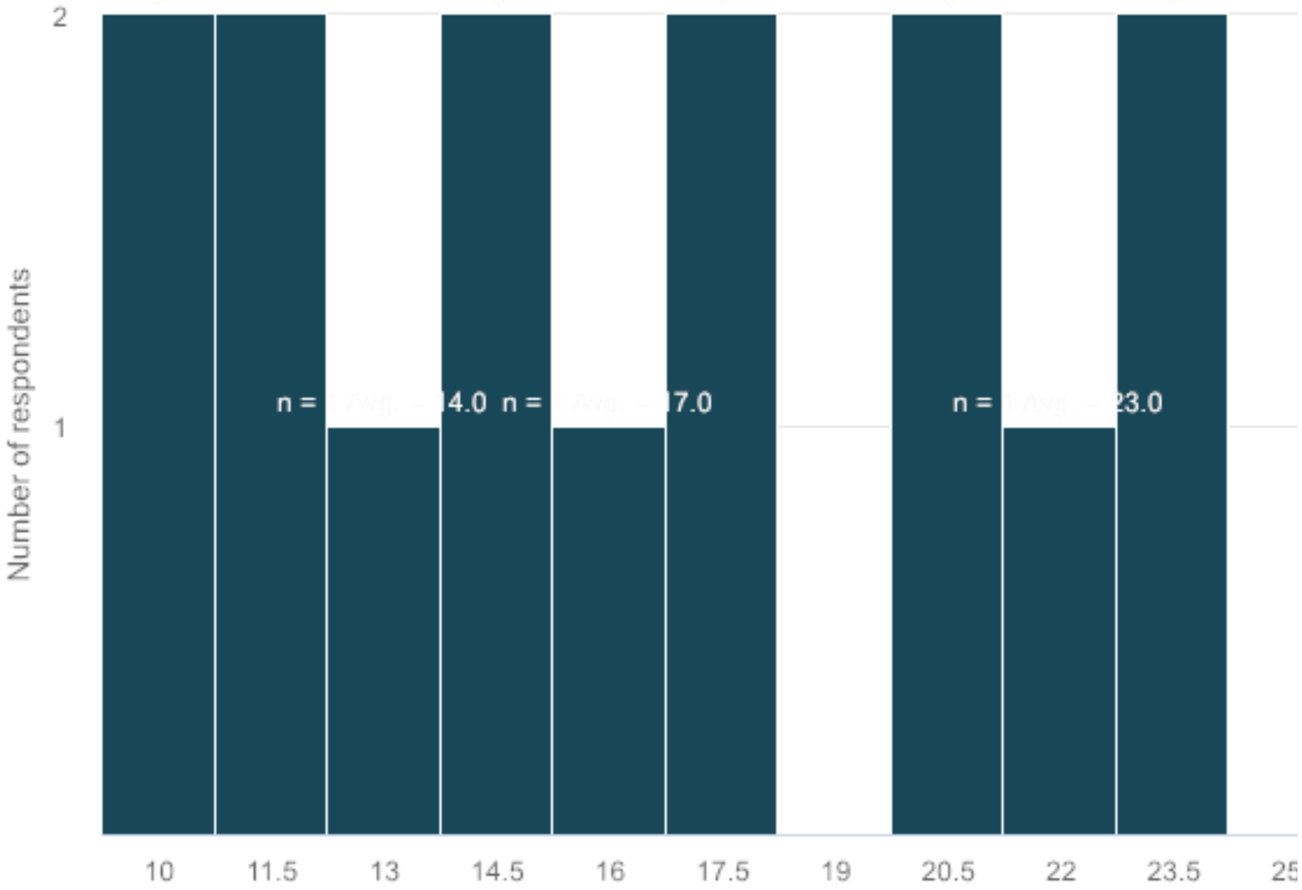

	Min value	Max value	Average	Median	Sum	Standard Deviation
	10	25	17.33	17	260	4.88

2. - I have read and understood the Information Sheet regarding the aims of this Delphi process and the researcher has answered any queries to my satisfaction.- I consent to my responses being stored securely electronically as part of the University of XXX Data Management Policy for a minimum of 10 years.- I understand that my participation is voluntary, that I do not have to answer any question I do not want to, and that I am free to withdraw from the Delphi process at any time.- I understand that I can request the withdrawal from the data collected any personal information (including my e-mail address) and that whenever possible researchers will comply with my request.- I understand that anonymised data (i.e. data that do not identify me personally) cannot be withdrawn once they have been included in the study.- I understand that any information recorded in this study will be anonymised and will remain confidential. No information that identifies me will be made publicly available

Number of respondents: 15

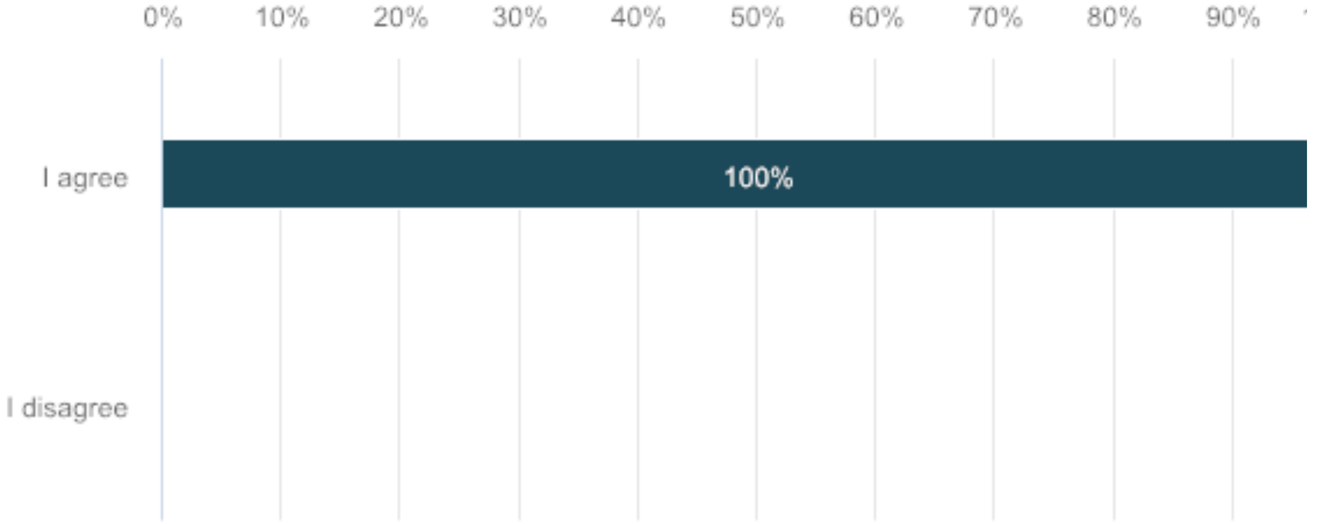

	n	Percent
I agree	15	100%
I disagree	0	0%

3. Please comment if you agree on this definiton of thoracic re-irradiation and/or suggest any modeifications to it.

Number of respondents: 15

Responses
It should include delivery of radiotherapy to the thorax for any reason, not just lung cancer (e.g. esophageal, thymic, HD, lymphoma, etc)<br><br>it could be agnostic to overlap, since the combined effects may contribute to complications. though, "RE-irradiation" is generally considered overlap. I would modify with language to clarify "any overlap with prior radiation portals".
add: and the possibility of cure
I agree
I would add the word "radical" ie 'any second or subsequent dose of "radical" radiation to the thoracic cavity for non-small cell lung cancer, provided that there is a significant overlap of previous dose on either the PTVs or OARs, given with the intent of long-term disease control.
Yes
1. need to define what you mean by significant overlap<br>2. in cases of SCLC, can still re-irradiate in very small number of cases and aim for long-term control (much less frequent but does happen; 1st Dx is NSCLC and then comes back with SCLC or other way)
Agree.<br>The only problem with the definition is the word "significant' - I guess that is in the eye of the beholder? Is there any way to be more precise?
Suggest modifications<br><br>If you are looking for anatomical definition the suggest to replace thoracic cavity by thorax and surrounding regions, for example:<br>Chest wall is not part of the cavity but there are relevant OARs and toxicities for re-RT<br>Also low neck/supraclavicular area which can overlap e.g. with prior Pancoast RT<br>Also spine e.g. treatment of an oligometastasis can overlap with prior thorax RT<br><br>It is not only prior RT for NSCLC in the thoracic cavity that is relevant e.g. previous breast RT may be important<br><br>Regarding the comment about overlap of previous dose, suggest to make some distinction between low and high dose. Significant low dose overlap may be of little consequence, whereas significant high-dose overlap is often relevant
Agree
I would suggest to use an overlap of isodose lines as definition of re-irradiation e.g. the 50% isodose line
Yes I think this is a sensible description.

Agree.
Agree
Agree
I would suggest the following:<br><br>'any second or subsequent dose of radiation to the thoracic cavity for non-small cell lung cancer, with or without significant overlap of previous dose on either the PTVs or OARs, given with the intent of long-term disease control.'<br><br>There is limited data on mechanistic underpinnings of post radiation pneumonitis and depending on the temporal relationship between the 2 RT courses, or the systemic treatment in use, a 2nd course even it's to a different thoracic site, may be relevant.

4. Assuming that the patient is fit, which of these clinical situations would you offer radical thoracic re-irradiation? (Overlap refers to the amount of tissue which will be treated in both the first and second radiation treatment)

Number of respondents: 15

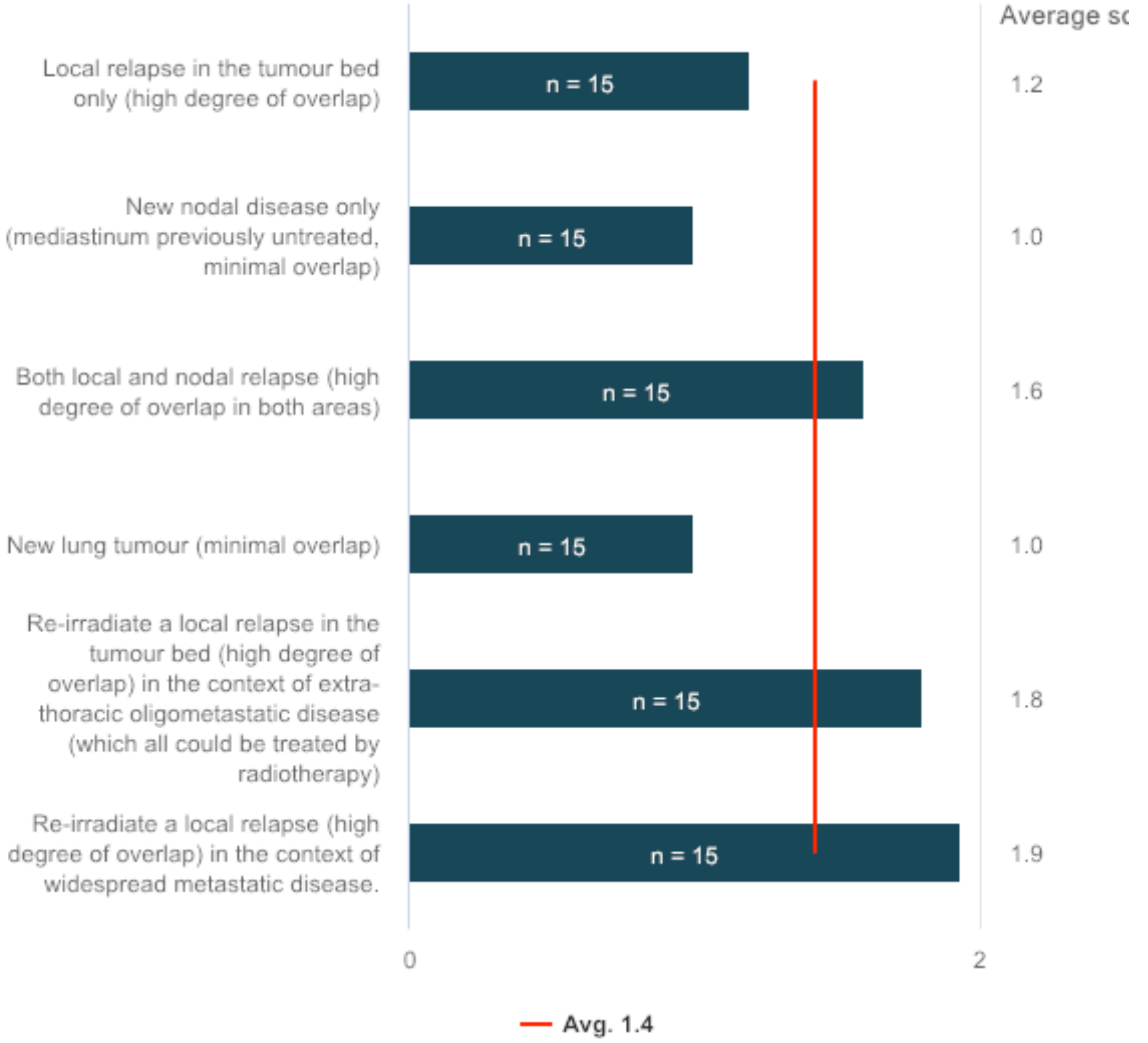

	Offer re-irradiation	Offer alternative treatment	Average	Median
Local relapse in the tumour bed only (high degree of overlap)	80%	20%	1.2	1
New nodal disease only (mediastinum previously untreated, minimal overlap)	100%	0%	1	1

Both local and nodal relapse (high degree of overlap in both areas)	40%	60%	1.6	2
New lung tumour (minimal overlap)	100%	0%	1	1
Re-irradiate a local relapse in the tumour bed (high degree of overlap) in the context of extra-thoracic oligometastatic disease (which all could be treated by radiotherapy)	20%	80%	1.8	2
Re-irradiate a local relapse (high degree of overlap) in the context of widespread metastatic disease.	6.67%	93.33%	1.93	2

5. Please describe any other clinical situations that you would consider radical thoracic re-irradiation

Number of respondents: 11

Responses
new histologic cancer in previously treated field
New primary tumor in a previously irradited area (e.g. second lung cancer after lymphoma treatment) and if there is no alternative such as surgery, MWA.
Radical RT to lung cancer in previoulsy treated breast cancer field
would accept high degree of overlap in tumour bed if overlying lung only and far away from serial structures
The situations above that say 'minimal overlap' wouldn't meet your definition of re-irradiation at the top.
Please note:<br>We would first consider options like salvage surgery<br>Especially for high-risk patients we would take into account if there are draggable mutations and the prior/expected response to systemic therapy<br><br>We would consider not only degree, but also location and dose-level of any overlap (important for example for answer to scenario 3 above)<br><br>Some patients getting radical doses for oligometastases (e.g. chest wall, spine) might meet criteria for overlap
Possibly local relapse in a superior sulcus tumour
Oligoprogression under systemic therapy; palliative intent irrespective of metastatic status
None
above list is comprehensive
To add to point 4) the nature of the relapse is probably important, with re-RT offered especially if disease free interval is long and there is good control post first RT. If disease free interval is short, probably worthwhile considering alternative local treatments - RFA etc.<br><br>Would also consider thoracic Re-RT in setting of definitive treatments for oligomet following previous RT for other disease histologies such as head and neck SCC, oesophageal ca, where second primaries are common.

6. What would you consider the minimum interval between initial radiotherapy and re-irradiation to be?

Number of respondents: 15

Responses
12 months
1 year
1 year
No evidence base for this. I would take this on a case by case basis. In general, I don't scan for at least 3/12 after initial RT anyway.
if high degree of overlap or full overlap, 12 months.<br>If minimal overlap (marginal recurrence, nodal recurrence) then 6 months
no set minimal interval, but the longer the interval the more pushing I will be for re-irradiation
6 months
In general 6 months
If minimal overlap, no minimum interval; would not radically irradiate local infield failure
usually 6 months
6 months
goal of 6 months
6 months minimum, but ideally longer
6 months
At least 6 months interval. Usually the minimum time it takes for imaging changes post RT to stabilise.

7. What is the worst ECOG performance status you would consider re-irradiation for?

Number of respondents: 15

Responses
ECOG 1
1
2
Very highly selected PS3 patient eg A chronic stroke patient in a wheelchair. Not a PS3 patient due to breathlessness
1
PS2
3
In general 2
ECOG 2
2
Ideally 2 but we treat SABR patients with a PS of 3 and would irradiate these patients if they had a local relapse or new primary/met with some overlap.
ECOG 2
2
2
ECOG 2

8. In your clinical practice, what investigations do you perform before offering radical thoracic re-irradiation?
Number of respondents: 15

Responses
high quality chest CT w/IV contrast<br>FDG-PET/CT to evaluate other sites of disease
PET<br>EBUS<br>PFTs<br>MRI brain<br>repeat attempt at biopsy of suspected lesion
Whole body FDG-PET-CT scan<br>MRI brain<br>Pathological confirmation of malignancy<br>EBUS/EUS and on indication even a mediastinocopy
CT<br>PET-CT<br>Lung function tests<br>O2 sats
FDG-PET<br>CT thorax (usually part of PET)<br>brain imaging (CT typically)<br>ideally biopsy confirmation of recurrence<br>Sometime PFTs - but does not usually alter management.
PET CT, PFTs, bloods
PET/CT<br>MR brain<br>Biopsy if possible<br>Consider medistinoscopy or EBUS
CT thorax/upper abdomen<br>Whole-body FDG PET/CT<br>Brain MRI<br>Tissue diagnosis if possible (in general avoiding excessive instrumentation/intervention in areas of high-dose re-RT)<br>Bronchoscopy or endoscopy if indicated (e.g. for knowledge about endobronchial disease or risk of fistula formation/perforation), +/- EBUS/EUS for accurate mediastinal staging<br>MRI spine if indicated (e.g. may be necessary for recurrent Pancoast tumors)<br>Lung function
Biopsy confirmation of recurrence/new primary;<br>FDG PET scan
CT thorax and abdomen; FDG-PET CT; cranial MRI; pulmonary function test; PET lung ventilation/perfusion
CT Chest/abdomen 1st.<br>PET/CT for all<br>Biopsy if possible<br>CT/MRI brain if nodal relapse
Largely dependent on systemic therapy options, which I discuss with the

medical oncologist.
Pulmonary Function Tests<br>Full Blood Count, Urea and Electrolytes, Liver Function Tests, Bone Profile<br>Contrast CT of CAP<br>PET/CT scan<br>Occasionally a V/Q scan
CT restaging +/- mr brain<br><br>PET imaging if for radical intent (covered by government)
PET_CT to complete staging and delineate disease.<br>Lung function tests.

9. What would you accept as the minimum DLCO and FEV1 before re-irradiation?
Number of respondents: 15

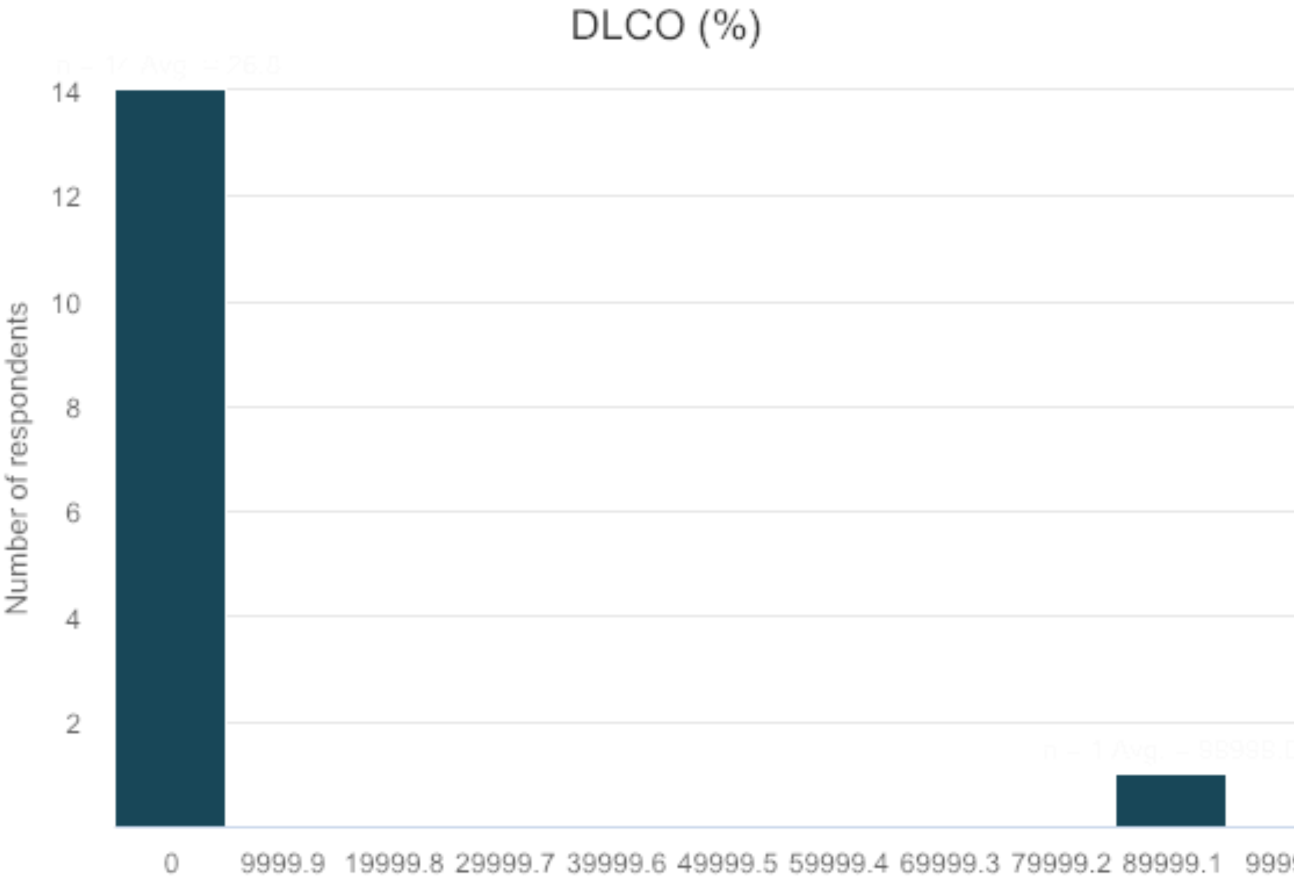

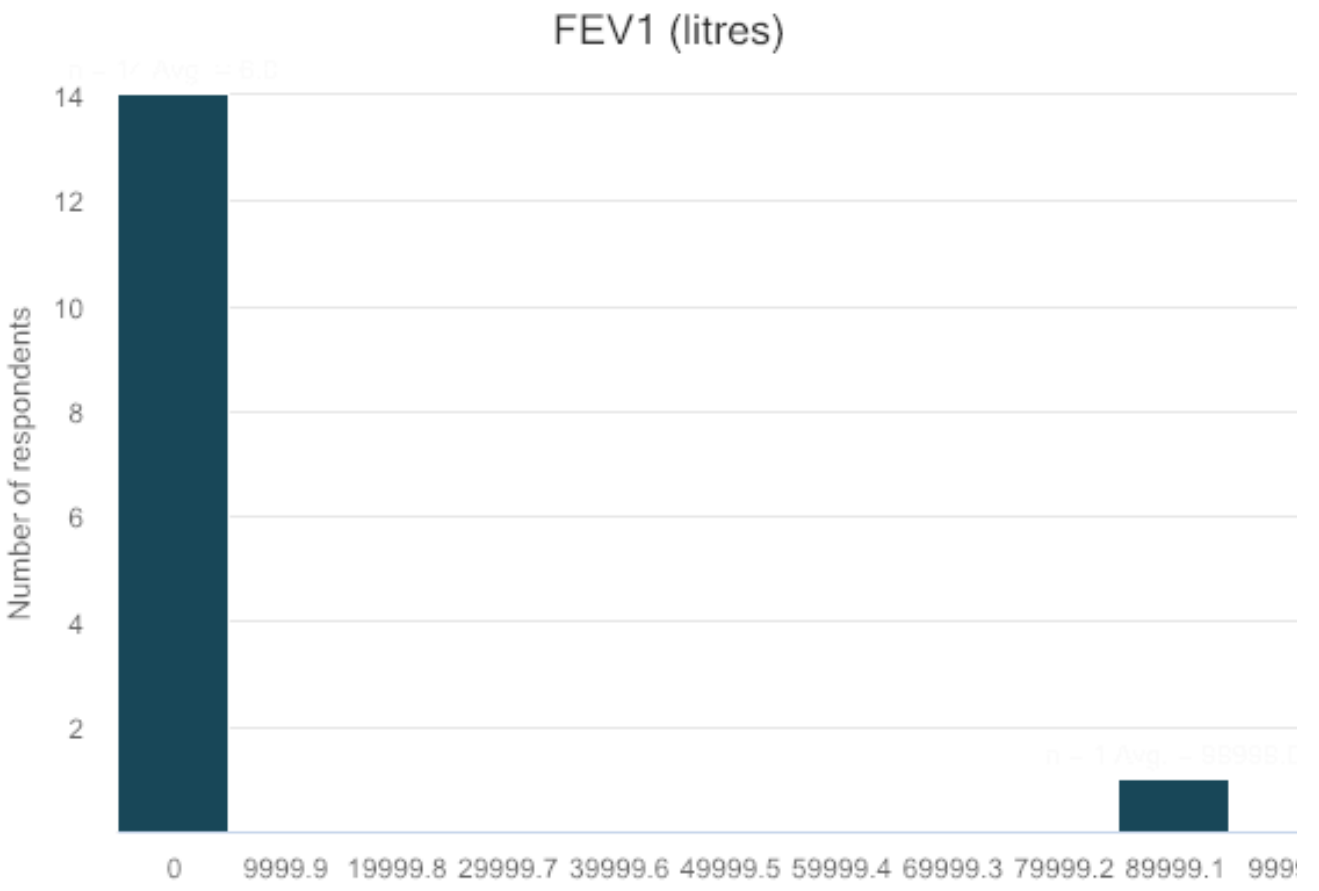

	Min value	Max value	Average	Median	Sum	Standard Deviation
DLCO (%)	0	99999	6691.6	30	100374	25812.72
FEV1 (litres)	0	99999	6672.2	1	100083	25818.08

10. Please describe any other considerations you have before offering thoracic re-irradiation.

Number of respondents: 15

Responses
i don't refer to DLCO or FEV1 for this situation
relationship of new dosing to previously irradiated OARs
A reasonable chance to cure the patient. E.g. no N3 disease.<br><br>Q 9: I would not consider a threshold for re-RT with SBRT. Otherwise, FeV1 30 % (not in absolute litres)
Case by case basis. I don't have any definitive cut off points, but I would not treat ILD patients
NOTE FEV1 limit above - i wanted to enter 0.6L but could not<br><br>Prior toxicities associated with initial course of RT - degree of pneumonitis? Peripheral skin stigmata of late RT fibrosis.
For DLCO and FEV1; no set lower limit (but would be worried if less than 30% predicted); also depends on amount of lung in XRT field<br><br>Suitability for other radical options such as surgery<br>If high risk, suitability for microwave ablation (if lung recurrence)
Both FEV1 and DLCO depend on the volume being treated (stage I vs. stage III). I would consider 30% a soft threshold for SABR for both. FEV1 in litres is not all that useful. % predicted is more reflective. Your FEV1 field above allows only integers so I couldn't put in 0.8
No specific lung function limits<br>Prior RT plan acquired (ideally digital copy) and reviewed along with diagnostic imaging, and indication for re-RT agreed by at least 2 radiation oncologists from our "re-RT" lung team<br>MDT case review in our/referring center, with support for re-RT<br>Option of salvage surgery considered<br>Ideally knowledge of molecular analysis<br>Patient and referring physician understand and agree with the risks<br>If risks considered excessive, especially in selected patients with central disease, we may advise against re-RT or advise an intermediate dose (still with the aim of long-term control)<br>Life expectancy in general >6 months<br>We also take into account volume, especially ion risks seem high, since we think that large volume disease may do less well (https://www.ncbi.nlm.nih.gov/pubmed/25736570)
Previous location and grade of radiation oesophagitis<br><br>Would accept 0.5 l FEV1<br><br>Previous spinal cord dose
No fixed threshold for DLC and FEV1 -> depending on volume to be irradiated and overall life expectancy

No fixed cut off for lung function and depends on size of target and the amount of functioning lung the re-irradiation would give.<br>Always way up risk of cure/long term control versus risk of acute and late toxicity.<br>Alway review cases with peers to get consensus.
Previous fractionation regimens, overall disease course. Note that no strict thresholds for reRT
Previous side-effects from radiotherapy.<br>Time interval between treatments (longer better)<br>Risks to critical organs in the treatment field<br>Likelihood of clinical benefit
if biomarkers not previously done, then I would obtain these (i.e. EGFR, ALK, PDL1). at times these are great 'palliative' options that can have lengthy control without as high upfront risk as re-RT
Background interstitial lung disease?<br>Any treatment - drug or previous RT induced pneumonitis?<br>Concurrent or recent use of gemcitabine or taxis<br>Concurrent or recent use of immune checkpoint inhibitors<br>Concurrent or recent use of tyrosine kinase inhibitors<br>Any ongoing chest infections?<br>Patient's overall performance status.<br>Systemic disease status

11. When re-irradiating patients, please list the treatment techniques you use (e.g. volumetric arc therapy, 3-field conformal radiotherapy)?

Number of respondents: 15

Responses
I focus on very tight fields with as many beams as possible to optimize compactness. more important than technique is considering the anatomic structures that may be best avoided without any beams re-traversing through them (esophagus/spine)
4DCT<br>breath hold<br>IMRT<br>contrast at simulation
VMAT<br>Proton therapy if aproved by insurance company
VMAT<br>(Rarely use 4D CT)
IMRT / VMAT for conventionally RT including mediastinum<br>IMRT / VMAT / DCAT / HybridArc for SABR
VMAT or step and shot IMRT (dependent on treatment centre)
VMAT or other IMRT
Always VMAT, smaller margins and daily on-line volumetric imaging are considered, with target or OAR based set-up depending on the scenario
VMAT, DCAT for stage I second primaries suitable for SABR
VMAT only, homogeneous or inhomogeneous stereotactic
VMAT<br>Daily Volumetric IGRT<br>4DCT if appropriate
IMRT, SBRT, or conformal therapy.
VMAT<br>3DCT<br>SABR<br>Parallel Opposed (Palliative)<br>Protons (if available)
VMAT or IMRT
VMAT/ IMRT

12. Would you offer stereotactic ablative radiosurgery (SABR) in the re-irradiation setting?

Number of respondents: 15

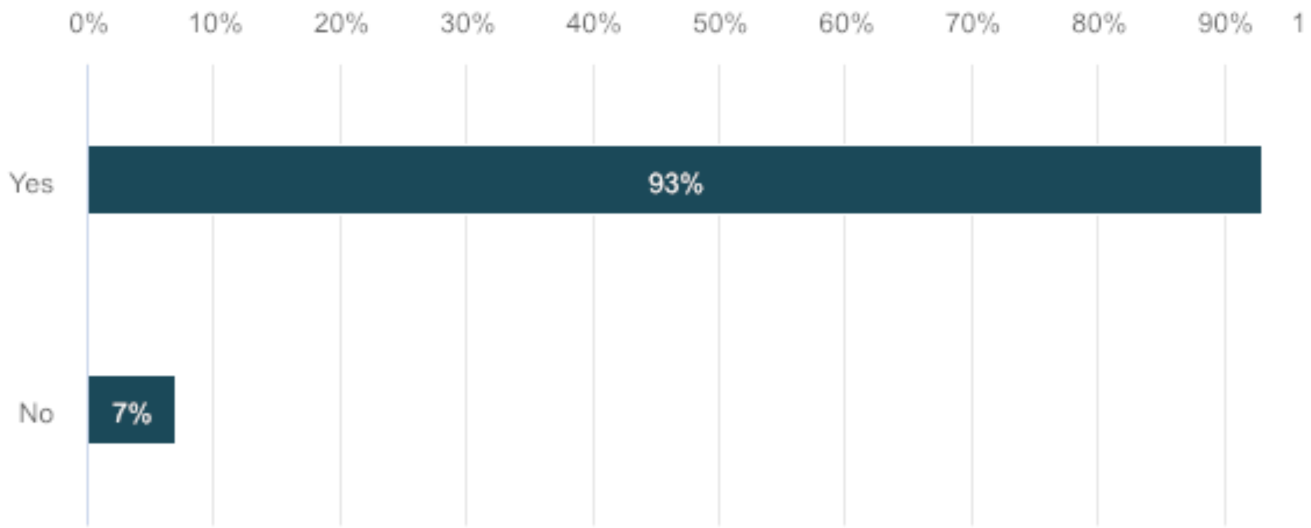

	n	Percent
Yes	14	93.33%
No	1	6.67%

13. Please explain the benefits and risks of using SABR?

Number of respondents: 14

Responses
a relapsed tumor is clearly radioresistant, and needs high BED
high dose conformality with ablative dosing
see phase II study MD Anderson: very good LC in case the volume is small and no overlap with OARs.<br>Convenient and easy.
I would only propose for primary disease recurrence only (not mediastinum)<br>I would only offer SABR to central primaries with minor overlap of prior RT<br>I would not treat ultracentral SABR re-irradiation<br><br>SABR achieves comparable control rates to that of de-novo un-irradiated disease, however with typically double the toxicity rates in the de-novo setting with a 1-2% mortality risk.
If 2nd primary and is eligible for SABR and no other lesions
Re-irradiation papers suggest reasonable toxicity (although higher than with de novo SABR) and reasonable local control (although lower than with de novo SABR)
For selected indications/patients we consider this safe and expect to achieve better chance of long term control
Would not use for a previously irradiated tumour because of uncertain toxicity. Soft tissue matching in the presence of radiation fibrosis may also be an issue.
Volume sparing due to higher accuracy;<br>higher dose for better local control
Only in cases where the overlap is outside the high dose region and OARS are not a concern.<br>For the patient less treatments, potentially less toxicity and higher local control rates
Dependent on location. Fistula, bronchial stenosis, pneumonitis, vascular toxicity, dyspnea, etc.
High rates of local control<br>High conformality around target<br>But risk of high BED.
benefit - higher dose per fraction to overcome radioresistance<br><br>risk - if significant overlap with critical OAR (i.e. esophagus/trachea), then a toxicity can be serious
Benefits<br>1) Achieving a steeper dose fall off to achieve dose constraints better;<br>2) Smaller radiation field, dose escalation and hypofractionation to overcome any presumed radio resistance or fraction size sensitivity;<br>3) Keeping overall duration short in setting of metastatic disease to as to avoid interrupting systemic treatment for too long;<br>4) Hypofractionation synergises better with immunotherapeutics.

Risks
1) High dose per fraction in a re-treatment area may run the risks of greater toxicity either to adjacent critical normal structures (especially 'serial' structures) or radiation pneumonitis.
2) Target delineation is critical in SABR which is sometimes technically challenging in setting of post-1st RT interstitial lung changes

14. Please list the reasons why you feel that SABR is not appropriate for re-irradiation.

Number of respondents: 1

Responses
Needs to be offered in context of a clinical trial

15. Would you offer proton re-irradiation?

Number of respondents: 15

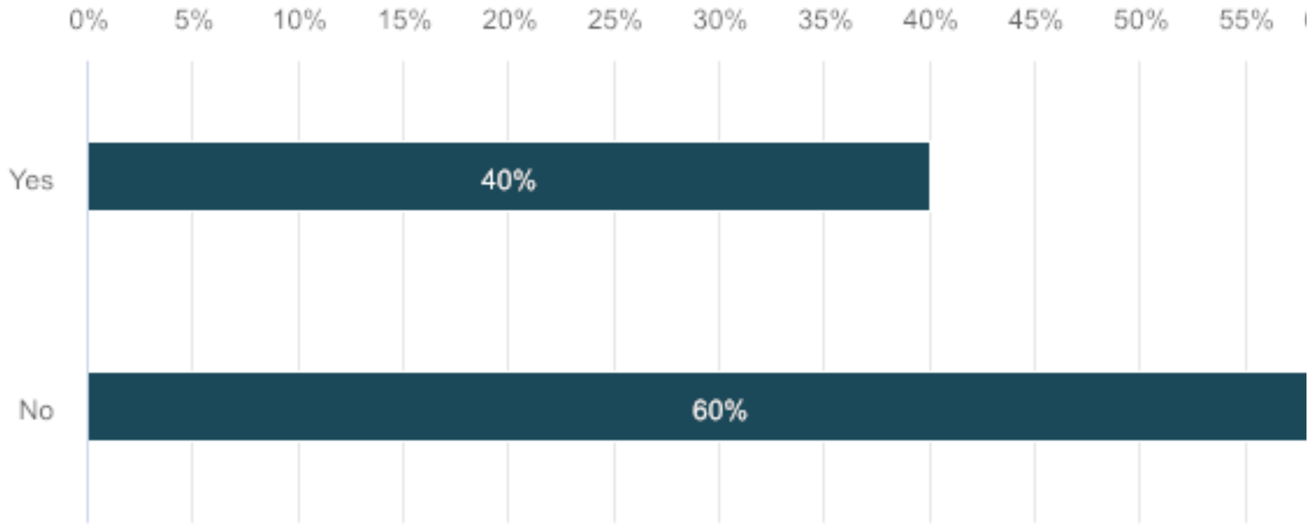

	n	Percent
Yes	6	40%
No	9	60%

16. Please explain the benefits and risk of proton re-irradiation?

Number of respondents: 6

Responses
it can allow for single or few beam tracks that completely avoid OAR areas, when needed
Depends on planning comparison, but in general much lower doses to heart and mediastinum (factor 2-4)
Would reduce dose to OARs (low and medium dose); need to consider opening re-irradiation trial which allows protons to be used (stratification factor)
As above, dependent on location.
Less dose spill to surrounding normal tissues
But delivered with good quality assurance and with robust breath-hold techniques, proton re-RT can theoretically offer dosimetric advantages distal to RT field and reduce overall dose to previously RT-exposed lung.<br><br>Proton re-RT is most useful in setting of significant interstitial lung changes, background interstitial lung disease or previously RT-exposed central structures.

17. Please list the reasons why you feel that protons are not appropriate for re-irradiation

Number of respondents: 9

Responses
not available to me
Needs to be offered in context of a clinical trial.<br>No access to protons at my site, but would refer to a centre with protons if a clinical trial was available
Not available to me.
1. We don't offer protons in the country where I practice<br>2. The only RCT of protons in lung showed no benefit.
Not at this time<br>In the highest risk, central scenarios, OARs are frequently against/in the target and there is no expected benefit from protons<br>There is little experience with protons, IGRT technology may lag behind and uncertainties may make dosimetry less reliable
not accessible to my population. Motion management issues not resolved for protons.
re-irradiation data for protons not convincing in the literature; toxicity is mostly in-field -> no benefit of protons
Not available and not convinced it has a role for most lung cancer cases.
no access to protons! also minimal evidence to support its use

18. Please list the dose and fractionation schedules you use for conventional thoracic re-irradiation?

Number of respondents: 15

Responses
I may use 2 Gy x 30 with carbo/taxol (small field, well tolerated)
60 Gy/30 fx
- Nodal involvement: concurrent cisplatin-etoposide and 60 Gy/ 2 Gy
55-60Gy in 20 fractions
60Gy/30/5<br>54Gy/27/5
60Gy in 30Fx (preferred)<br>55Gy in 20Fx
60 Gy in 15 fractions for stage I<br>60 Gy in 30 fractions (with chemo) for stage III.
Typically 30x2Gy, homogenous dose, especially if there is significant high-dose overlap, with no concurrent chemo/systemic therapy<br>If chemotherapy is given then it is used first, followed by sequential RT<br>Depending on the overlap degree/dose-level we may consider moderate hypo-fractionated schedules like 20x2.5-3Gy<br>An example of an "intermediate dose" would be 20x2Gy
For tumours previously unirradiated: 55 Gy in 20 fractions; 60 Gy in 30 fractions. Would not give a radical dose to a previously radically irradaited tumour
60gy in 30 fractions
45Gy/30 BD<br>60Gy/30
2 Gy x 30 vs. BID regimen (equivalent), 45-60 Gy in 15 fractions.
55 Gy in 20 Fractions over 4 weeks<br>60-66 Gy in 30-33 Fractions over 6-6.5 weeks
60/30<br>45/30<br>40/15<br>30/10
2Gy per fraction 60 in 30fr (with or without concurrent chemo if appropriate)<br>2.5Gy per fraction 50 in 20fr if RT alone<br>2.6Gy per fraction 65 in 25fr if RT alone

19. Please list the dose and fractionation schedules you use for SABR re-irradiation?

Number of respondents: 15

Responses
12 Gy x 4
50 Gy/5 fx
- SBRT: 4x12 Gy
NA
54Gy/3/3<br>50Gy/5/3<br>48Gy/4/3<br>28Gy/1/1
SABR (54Gy in 3, 60Gy in 5 or 8 Fx); if suitable for SABR
60 Gy in 8 fractions
Varies depending on the scenario, could be 5x11Gy, 8x7.5Gy, 12x5Gy
Would not retreat a previously radically irradiated tumour. But would use 54 Gy in 3 fractions, 48 Gy in 4 fractions or 50 Gy in 10 fractons for previously untreated tumours.
5-12 fractions<br>total dose depending on indication (curative, palliative. oligoprogression, oligometastasis, ...)
60/8
12.5 Gy x 4, 18 Gy x 3, 10 Gy x 5
48 Gy in 4 fractions<br>55 Gy in 5 fractions<br>60 Gy in 8 fractions
60/8<br>48/4<br>50/5
48 Gy in 4fr EOD<br>45 - 60 Gy in 5fr EOD<br>60 Gy in 8fr EOD<br>50Gy in 10fr daily

20. Please list the dose and fractionation schedules you use for proton re-irradiation?

Number of respondents: 15

Responses
I prefer SBRT doses for PBT
NA
The same as for photons.<br>Obviously, no PT when feasible with SBRT
NA
N/A
60Gy CGyE, although there is evidence to suggest going to 64/66Gy as it is associated with improved outcomes
N/A
N/A
Not applicable
-
N/A
No differences, dependent on SBRT vs. conventional
None used personally, but would consider any of the above
n/a
Only available in our centre in 2020. Fractionation schemes should not differ though.

21. Please describe the technique you use to image re-irradiation patients for radiotherapy planning.

Number of respondents: 15

Responses
always with 4DCT
PET fusion to CT planning images, with contrast, 4DCT and breath hold
4D-FDG-PET-CT in treatment position with i.v. CT contrast
CT planning scan with contrast. (Rarely use 4D)
4DCT simulation<br>on occasion, 4D PET/CT simulation
4D is a must to minimise volumes
4D-CT
4DCT, usually free-breathing (can consider breath-hold in selected patients), +/- IV contrast (otherwise co-registered contrast diagnostic CT can be used with registration on the specific regions of interest)<br>FDG PET/CT used for delineation (can be the diagnostic scan, option to make new PET scan in RT position and to obtain 4D PET/CT if necessary)<br>MRI if indicated
4D CT and PET/CT
4D-CT, FDG-PET CT
4DCT with contrast
4D CT scan, breathing control when needed, conformal techniques.
4DCT with IV contrast - fused with PET/CT
4DCT, IV contrast for optimizing delineation
4DCT with or without accessories (Abdominal compression or<br>Planning based on CT average

22. When re-irradiating, how do you register the dose of the initial treatment (e.g. rigid dose registration)?

Number of respondents: 15

Responses
i don't defer to dosimetry, and do it myself, given the lung collapses and shifts after the first course of radiotherapy in a manner that may be disparate from the mediastinum. i do not think there is a reliable class solution for this.
rigid
elastic deformation
Fusion of previous plans
rigid dose registration
Yes
Deformable registration. We use MimVista.<br>Any registration is problematic because of fibrosis and retraction.
Typically rigid (for all regions of interest since global registration may be unreliable, especially if there has been tissue deformation or if there is missing tissue)<br>Deformable available (we are using Velocity), but prone to uncertainty and pitfalls<br>Initial plans is also evaluated independently, without registration
Rigid dose registration
rigid image registration
Rigid dose registration
Deformable
Rigid registration
Rigid dose registration
Rigid dose registration.

23. For conventionally fractionated re-irradiation with photons, what margins do you use to expand the GTV to CTV?

Number of respondents: 15

Responses
0
5mm
5 mm with editing for normal structures such as bone or tracheal cartilage, large vessels
8mm
iGTV (motion encompassing GTV) + 5mm
0 or 5mm (would use no CTV margin if high risk)
5 mm
Single expansion of 5-10mm for ITV-PTV<br><br>Immobilisation may be considered for selected patients, e.g. Pancoast, low neck/supraclavicular
0.5 cm
5mm to max. 10 min
5mm
0.5-0.8
5mm
0.5 cm
0 - 5mm from GTV_ITV in metastatic disease (depending on difficulty in delineating active disease)<br>In radical treatment for new lung primary, will conform to conventional expansion margins from GTV_ITV (6mm SCC, 8mm for AdenoCa)

24. For conventionally fractionated re-irradiation with photons, what margins do you use to expand the CTV to PTV?

Number of respondents: 15

Responses
depends on various IGRT factors, though typically 5mm. i may reduce radial margins to 3mm, but i never reduce the sup/inf PTV margin less than 5mm (unless using MR-guided)
5mm
2 mm for set-up, the rest depending on the movement. Typical 8 mm
5mm
5mm-10mm
5mm
5 mm
See above
0.5-1.0 cm
-
5mm
0.3-0.5
Inistutionally dependent on set-up error, but would normally use 0.7 cm laterally and 1.0 cm sup/inf
0.5 cm
3-5mm.

25. When considering re-irradiation dose tolerances, how do you take into account the time interval between the initial treatment and the re-irradiation for each organ at risk (e.g. assume a 50% recovery of the spinal cord tolerance after X amount of time)? Please enter n/a if you have no data regarding this.

Number of respondents: 15

Lung	Bronchial tree	Oesophagus	Spinal cord	Brachial plexus	Aorta	Skin	Heart
n/a	n/a	n/a	n/a	n/a	n/a	n/a	n/a
n/a	n/a	n/a	5 yr	n/a	n/a	n/a	n/a
?	?	?	?	?	?	?	?
n/a	n/a	n/a	n/a	n/a	n/a	n/a	n/a
n/a	n/a	n/a	50% recovery of the spinal cord tolerance after 12 months amount of time	50% recovery of the spinal cord tolerance after 12 months amount of time	not an OAR for re-irradiation	n/a	n/a
No robust data to my knowledge	No robust data to my knowledge	No robust data to my knowledge	assume 50% recovery after 12 m	Chen IJROBP 2017 re-irradiation data often used	No robust data to my knowledge	No robust data to my knowledge	No robust data to my knowledge
25% after 6 months	25% after 6 months	25% after 6 months	Allow BED2 of 120 after 6 months	25% after 6 months	25% after 6 months	25% after 6 months	25% after 6 months
n/a	n/a	n/a	n/a	n/a	n/a	n/a	n/a

n/a	n/a	n/a	50% recovery after 12 months if cord dose did not exceed 45 Gy initially	n/a	n/a	n/a	n/a
no recovery for fibrosis; full recovery for pneumonitis after 6 months	maximum accumulated dose of 120Gy 2Gy EQD	for high-dose parameters 25-50% recovery after 6 months	50% recovery after 6 months	25% recovery after 6 months	maximum accumulated dose of 120Gy 2Gy EQD	mostly irrelevant with VMAT	no hard constraint
n/a	allow 30%	allow 30%	25-50% depending on case/volume/time	as spinal cord	120Gy cumulative dose with no recovery	Allow 30%	N/A
N/A	N/A	N/A	6 months	N/A	N/A	N/A	N/A
Try to minimise dose entirely to unaffected lung. Keep V20 <30%. Keep MLD to < 18 Gy	No constraints but avoid hotspots in re-treatment plan	Try to keep V50 to < 50% and try to avoid hotspots in re-treatment plan	Summed plans (old and new) to less than 50 Gy in 2 Gy per fraction (A/B of 2)	Try and keep summed plans (old and new) to less than 66 Gy in 2 Gy per fraction (A/B of 2)	No constraints but avoid hotspots in re-treatment plan	Until previous toxicity, don't routinely consider	No constraints but avoid hotspots in re-treatment plan
n/a	n/a	n/a	n/a	n/a	n/a	n/a	n/a

If interval >12m, I will assume new course of RT.	N/A on time interval vs recovery	N/A on time interval vs recovery	Accept a max point dose of 70Gy [EQD2(2)] if time interval of at least 5 months between course, provided radiotherapy is delivered with daily image guidance and appropriate immobilisation (vac loc). BED_max point [2nd course]/BED_max point [cumulative over 2 courses] should be 50%. Based on spine re-RT data.<br><br>If whole cord irradiation, with at least a few months in between, accept BED(2) of up to 135 Gy.	N/A on time interval vs recovery	N/A on time interval vs recovery	up to 120Gy [EQD2(3)] if at least 6 months in between. Data from re-RT for recurrent head and neck tumours.	N/A on time interval vs recovery

26. Please list any institutional dose constraints you use for the OARs in the thorax. If you have no dose constraints, please enter n/a.

Number of respondents: 15

Lung cumulative V5	Lung cumulative V20	Cumulative mean lung dose	Bronchial tree	Oesophagus	Spinal cord	Brachial plexus	Aorta	Skin	Heart	Any other constraints
n/a	35%	20 Gy	I refer to RTOG 1021 guidelines	I refer to RTOG 1021 guidelines	I refer to RTOG 1021 guidelines	I refer to RTOG 1021 guidelines	I refer to RTOG 1021 guidelines	I refer to RTOG 1021 guidelines	I refer to RTOG 1021 guidelines	I refer to RTOG 1021 guidelines
n/a	40	20	70	70	55	70	n/a	n/a	60	n/a
60	35	20	76	76	54	74	76	76	10	no
n/a	n/a	n/a	n/a	n/a	n/a	n/a	n/a	n/a	n/a	n/a
n/a	n/a	n/a	n/a	n/a	n/a	n/a	n/a	n/a	n/a	Assume constraints will be exceeded, but aim for to limit to original constraints, and cumulate doses with a 50% discount.
less than 65%	less than 35%	less than 20G	SABR guid	mean dose	for 55Gy in 20;	SABR guid	SABR guid	no constra	V30 Gy less	no

		y	elines	less than 34Gy; V55 less than 35%	max dose less than 44Gy, 1cc max less than 40Gy for 60-66gy in 30-33; max dose less than 48Gy, 1cc max less than 44Gy	elines	elines	ints	than 40%, V40Gy less than 30%; SABR guidelines	
ideal <65%, acceptable <95%	ideal <35%	ideal <22 Gy	point dose <90 Gy based on Cannon data	ALARA	Allow BED2 of 120 after 6 months	<66 Gy with forgiveness of 25%, see comment below.	For standard RT, ALARA. For SABR, we use 8 fraction constraint after forgiveness.	ALARA	ALARA	Note: this are not hard constaints. If exceeded slightly, we will re-consent patient with new discussion of risks.
n/a	n/a	n/a	Dmax (point) 120-	Dmax (point) 100	Dmax (point) 60Gy in 2Gy/fr	Dmax (point) 120-	In general Dmax	n/a	n/a	All constraints cumulative dose

			130 Gy in 2Gy/fr with a/b=3 (for central bronchial tree, but increasingly we are not accepting such high doses due to risk of grade V toxicity; also used, with same proviso, for hilar blood vessels)	Gy in 2Gy/fr with a/b=3, 120 Gy on oes+2mm, oes contoured on 4DCT, dose fall-off inside OAR	with a/b=2, 70Gy on cord+2mm (canal may be used as surrogate), dose fall-off inside OAR so that the "full thickness" dose is considerably less	130 Gy in 2Gy/fr with a/b=2 (on plexus or PRV depending on situation)	(point) 120-130 Gy in 2Gy/fr with a/b=3			from all RT plans using simple BED formula, no routine allowance for recovery Where n/a = as low as possible/individualised according to scenario/geometry Constraints are guidelines, lower whenever possible, and lower within the OAR (this is important - whenever possible we look to get a steep dose fall-off in the OAR)

										Constraints may sometimes be exceeded Trachea also Dmax (point) 120-130Gy in 2Gy/fr with a/b=3 PTV coverage may be compromised to achieve acceptable OAR doses
60% of aerated lung	35% of aerated lung	20 Gy (aerated)	n/a	0 if previous oesophagitis > grade 3	50 Gy cumulative allowing for recovery	n/a	n/a	n/a	n/a	n/a
no hard constraint but should be below 60-80%	30%	20Gy	D1cm3 < 80Gy	V60 < 17%; V74 Gy < 0.03 cm3	D0.1 50Gy	D1cm3 < 74Gy	no hard constraint	no hard constraint	no hard constraint	-
<70%	<35% conventi	<18 Gy	not for conventi	no	depends on dose	<TD for all lung	none for primary	none for pri	as QUANTE	No

	onal RT		onal RT			regimes	RT	mary RT	C	
N/A	N/A	N/A	N/A	N/A	N/A	N/A	N/A	N/A	N/A	N/A
N/A	N/A	N/A	N/A	N/A	N/A	N/A	N/A	N/A	N/A	None
n/a	n/a	n/a	n/a	n/a	n/a	n/a	n/a	n/a	n/a	n/a
≤65 % (Lungs minus GTV)	≤35 % (Lungs minus GTV)	≤20 Gy (Lungs minus GTV)	Cumulative BED (3) up to 126 Gy; V18 Gy/5 fr or BED equivalent < 4cc	Cumulative BED (3) up to 126 Gy; V 27.5 Gy/5 fr or BED equivalent < 5cc	Accept a max point dose of 70Gy [EQD2 (2)] if time interval of at least 5 months between course, provided radiotherapy is delivered with daily image guidance and appropriate immobilisation (vac loc). BED_max point [2nd course]/BED_max	Cumulative EQD2(2)≤66 Gy	Cumulative BED (3) up to 126 Gy	up to 120 Gy [EQD2(3)] if at least 6 months in between.	NA	Stomach for left lower lobe tumours: Dmax ≤ EQD2(3) = 60 Gy

					point [cumulative over 2 courses] should be 50%. Based on spine re-RT data.<br><br>If whole cord irradiation, with at least a few months in between, accept BED(2) of up to 135 Gy.					

27. What percentage rate of grade 1-2 toxicity would you accept for these organs at risk?

Number of respondents: 13

Lung (pneumonitis)	Oesophagus (oesphagitis)	Skin (erythema)	Brachial plexus (plexopathy)	Heart (pericarditis)
50%	50%	50%	50%	20%
30	35	40	15	20
25	50	100	1	3
100	100	100	100	100
100	100	100	30	30
100% - grade 1 is radiologic findings only	80%	80%	40%	40%
100	100	100	5	5
20	70	50	5	5
25	50	50	20	10
50	50	50	5	0
100%	100%	100%	<10%	<10%
50%	75%	50%	10%	5%
15%	Up to 50%	Up to 30%	<5%	<5%

28. What percentage rate of grade 3-4 toxicity would you accept for these organs at risk?

Number of respondents: 14

Lung	Oesophagus	Aorta	Spinal cord	Skin	Brachial plexus	Bronchial tree	Heart
20%	5%	20% (mostly b/c the limit is unknown)	5%	5%	5%	5%	5%
5	5	n/a	0	10	5	5	10
5	5	5	1	5	3	5	10
10	10	10	5	10	5	10	10
40	30	n/a	10	30	20	15	15
10	10	5	0	5	5	5	15
30%	20%	10%	10%	50%	10%	10%	10%
< 5	< 5	0	0	5	0	0	0
5 - 10%	5 - 10%	5 - 10%	5 - 10%	5 - 10%	5 - 10%	5 - 10%	5 - 10%
<5%	<5%	<1%	<1%	<5%	<1%	<5%	<5%
<20%	<20%	<5%	<5%	<5%	<5%	<5%	<5%
20%	33%	10%	5%	25%	5%	10%	10%
<5%	<10%	<5%	<3%	<5%	<3%	<5%	<5%
10							

29. What percentage rate of grade 5 toxicity would you expect from radical thoracic re-irradiation?

Number of respondents: 14

3%
5
3
<5
2%
10
<5%
We are not currently evaluating at level of expected toxicity garde I-IV since this is very hard to predict and may be influenced by non-RT factors<br>In addition, total number of patients treated with radical re-RT is still small<br>In general, we look at the clinical situation and what we think we can achieve and what the risks are<br>We do consider the risk of grade V toxicity since we have experienced this and in some situations this may lead us to advise against re-RT or to modify the dose (typically for centrally located re-RT): https://www.ncbi.nlm.nih.gov/pubmed/25736570<br>Patients may also decline when they hear the risks (could be estimated at up to 25%+ for selected patients requiring central/ultra-central re-RT)<br>Estimate of grade V risk can be complicated by attribution of event, e.g. bleeding, to RT or tumor (especially if endobronchial component/growth through the wall of the organ) and could lead to over-estimate of risks
10% if full overlap, depending on OAR's
2-5%, depending on treatment goal
10%
<5%
5%
<3%

30. Please describe how you perform treatment verification imaging during re-irradiation treatment?

Number of respondents: 15

Responses
CBCT
daily CBCT
daily CBCT
Cone beam CT including 4D cone beam CT
kV image pair to setup,<br>CBCT soft tissue match prior to each fraction
CBCT daily; 4dCBCT if/as needed
CBCT
In general daily on-line volumetric (CBCT) imaging
Daily CBCT
CBCT, frequency mostly daily; adaptive re-planning if needed
Volumetric daily CBCT
daily CBCT
Daily CBCT
standard CBCT
Daily CBCT (with or without 4D CBCT)

31. Do you offer concurrent chemotherapy with re-irradiation?
Number of respondents: 15

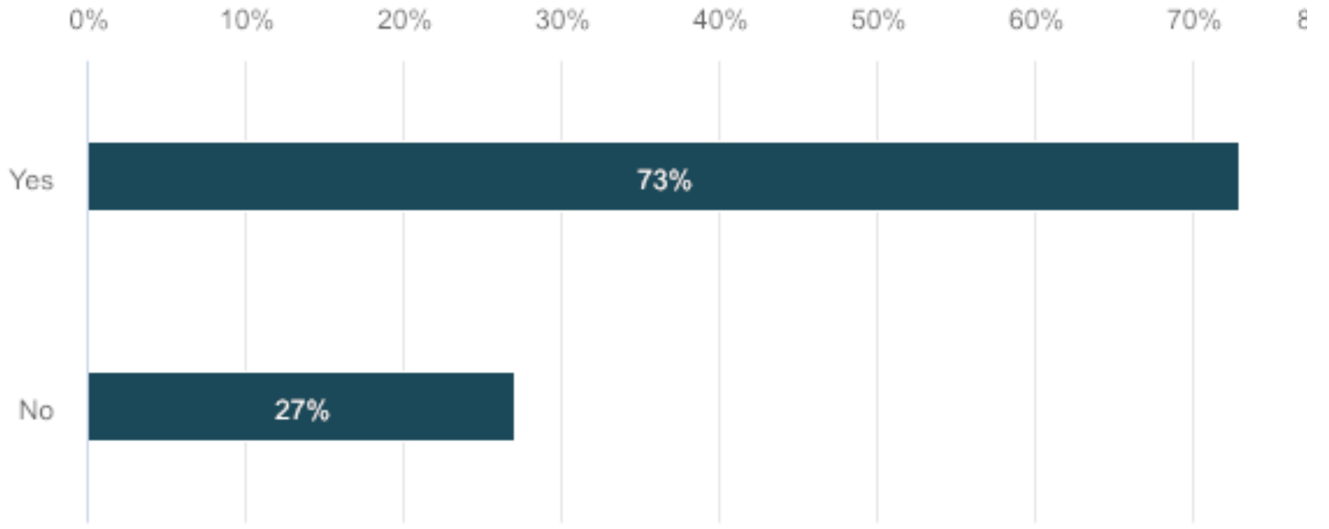

	n	Percent
Yes	11	73.33%
No	4	26.67%

32. In which patients do you recommend concurrent chemotherapy with re-irradiation?

Number of respondents: 11

Responses
only if 2 Gy x 30, small-field relapse
nodal failures after previous RT
N2-N3
Prior tolerance to chemoRT, renal function and hearing permitting platinum, ECOG=0-1, no significant weight loss
Stage III recurrence (i.e. mediastinal)
No overlap with previously treated volume
curative setting; if indicated from a systemic perspective
high performance status, conventional fractionation used
Those with nodal involvement who did not receive concurrent CRT before
good performance status, young
Patients who are receiving re-RT for new or recurrent lung primaries (with or without LNs).<br><br>Patients who are receiving re-RT for small volume metastatic disease

33. What chemotherapy would you use?

Number of respondents: 11

Responses
cis/etop or whatever medonc prefers
platinum doublet weekly
cisppltin-etoposide
platinum doublet (cisplatinum + etoposide or carboplatinum + paclitaxel)
cisplatin-etoposide usually, or another cisplatin doublet, sometimes carbo-taxol.
carboplatin and paclitaxel
Depending on histology and mutational status
standard carbo/taxol or potentially immunotherapy
Carbo/Taxol (weekly)
I don't rx chemotherapy, but platinum doublet i.e. cis pem, cis etop, cis vinblastine etc.
cisplatin doublet. Would avoid carbo-taxol if re-RT volume is large.

34. In which clinical situations do you offer systemic treatment (e.g. chemotherapy, immunotherapy) routinely after completion of re-irradiation?

Number of respondents: 15

Responses
If stage I-III relapse, I would give Durvalamab afterwards. No other consolidation chemo
nodal relapse
chemo: No<br>Immuno: N2-N3 disease
None. I only offer systemic therapy on relapse after re-irradiation
if nodal recurrence, i would recommend discussion of adjuvant durvalumab after conventionally fractionated re-irradiation
no routine
We would now consider Durvalumab for a recurrence that is stage III
Not routinely
immunotherapy only in cases of stable or responding disease; provided any pneumonitis < grade 3
if wide-spreat metastatic disease and RT was done in palliative intent
No-offer it before sometime to shrink disease.
defer to medical oncologist
Adjuvant Durvalumab after concurrent CRT re-irradiation for stage III NSCLC
do not typically offer consolidative chemo in era of durva. if patient has not had durva before, would offer post repeat chemoRT
1) Metastatic disease.<br>2) New lung NSCLC - adj durvalumab

35. How frequently do you perform surveillance scanning in fit patients after completion of re-irradiation treatment?

Number of respondents: 15

Responses
q3mo
every 3 months
Q 3months
Every 6 months until 3 years, then annualy to year 5<br>No evidence base for this, though!
standard assessment pattern - 3 monthly year 1, 4 monthly year 2, 6 monthly year 3-5 and annually thereafter
usually at 6 m. No routine scanning afterwards unless needed.
q 6 months x 5 years with CT
Individualised but is generally 3 monthly to start with (CT thorax/upper abdomen)<br>PET/CT if necessary<br>Imaging review within our radiation oncology re-RT team or in the MDT if any concerns
3 monthly for 2 years, thereafter 4- 6 monthly
every 3 months
6 monthly
every 2-3 months for 2 years
3 monthly until 12 months, then 6 monthly thereafter until 3 years
q 3 months in first year, then q 6 months for next 2 years, then yearly
Every 3-6 months for the first 3 years<br>Then 6 monthly for the next 2 years<br>Annually thereafter

36. Please add below any further comments regarding re-irradiation.

Number of respondents: 9

Responses
I advise pts that evaluating post-ReXRT films will be difficult.
extremely heterogeneous population and all treatments are highly individualized
I do not understand Q 25
Our long term data suggests that re-irradiation is safe and feasible with some very good long term outcomes for some patients, including cure.
Patients advised to report any problems during follow-up, they may come from some distance away in which case their lung physician has easy access to us if there are problems<br>In general we advise against (elective) biopsy/manipulation/instrumentation of re-RT tissues without prior discussion with our team (e.g. central airways due to risk of bleeding/perforation)<br>Small numbers of patients for true high-dose re-RT<br>Principles of management important
We do not offer radical reirradiation to a tumour previously radically irradiated since there is little reason to think thattreatment will be any more effective the second time around, unless there is some alteration, e.g. altered fractionation, or addition of chemotherapy if not used the first time around
none
no
None

Round 2

Total number of respondents: 15

1. Please enter your identification number (found on the e-mail with the link to this survey)

Number of respondents: 15

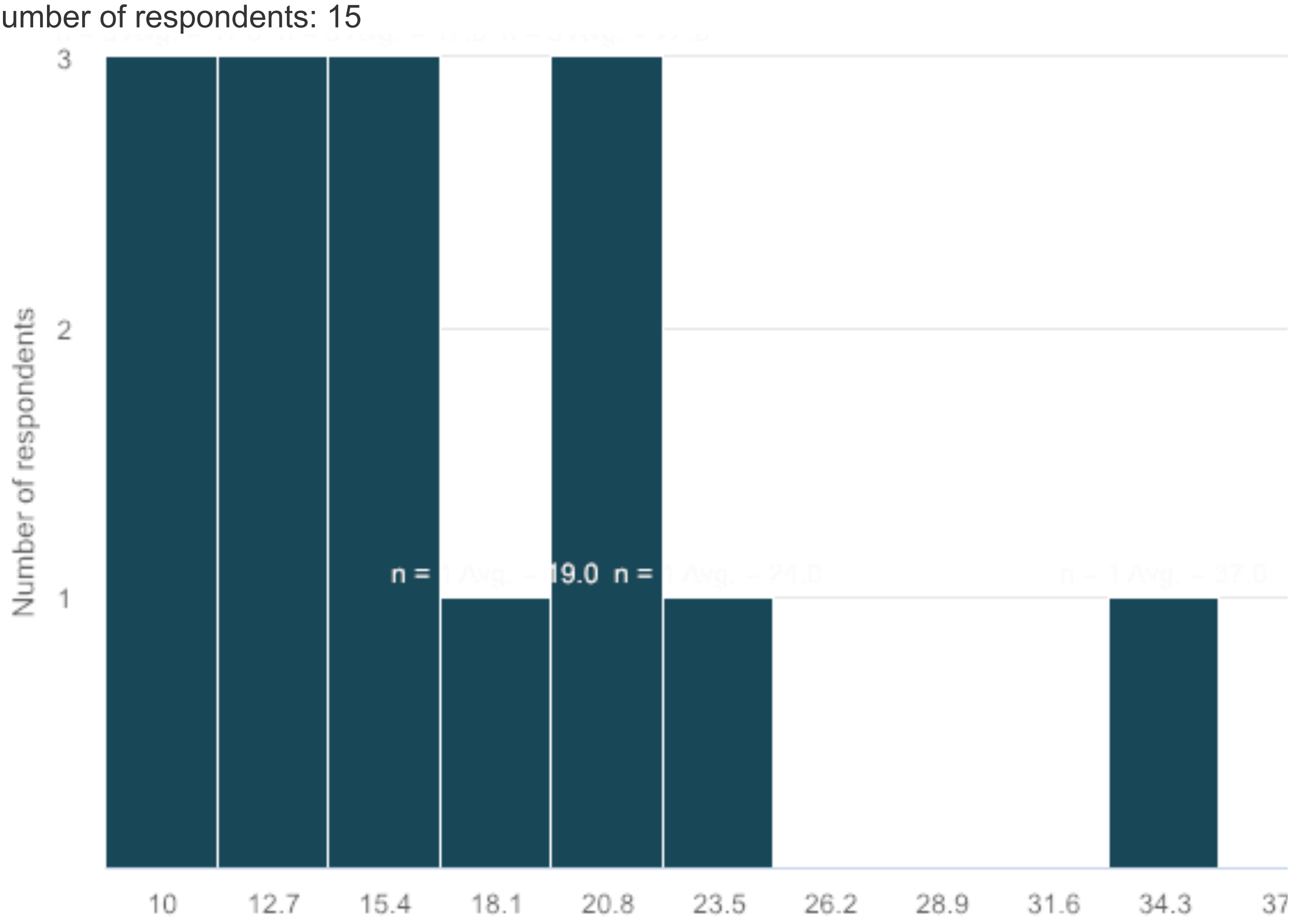

	Min value	Max value	Average	Median	Sum	Standard Deviation
	10	37	18.13	17	272	6.82

2. "Any dose of radical radiation for lung cancer, after initial radical radiotherapy to the thorax or surrounding tissues for any tumour histology, provided there is any overlap of previous dose in either the PTV or the OARs"

Number of respondents: 15

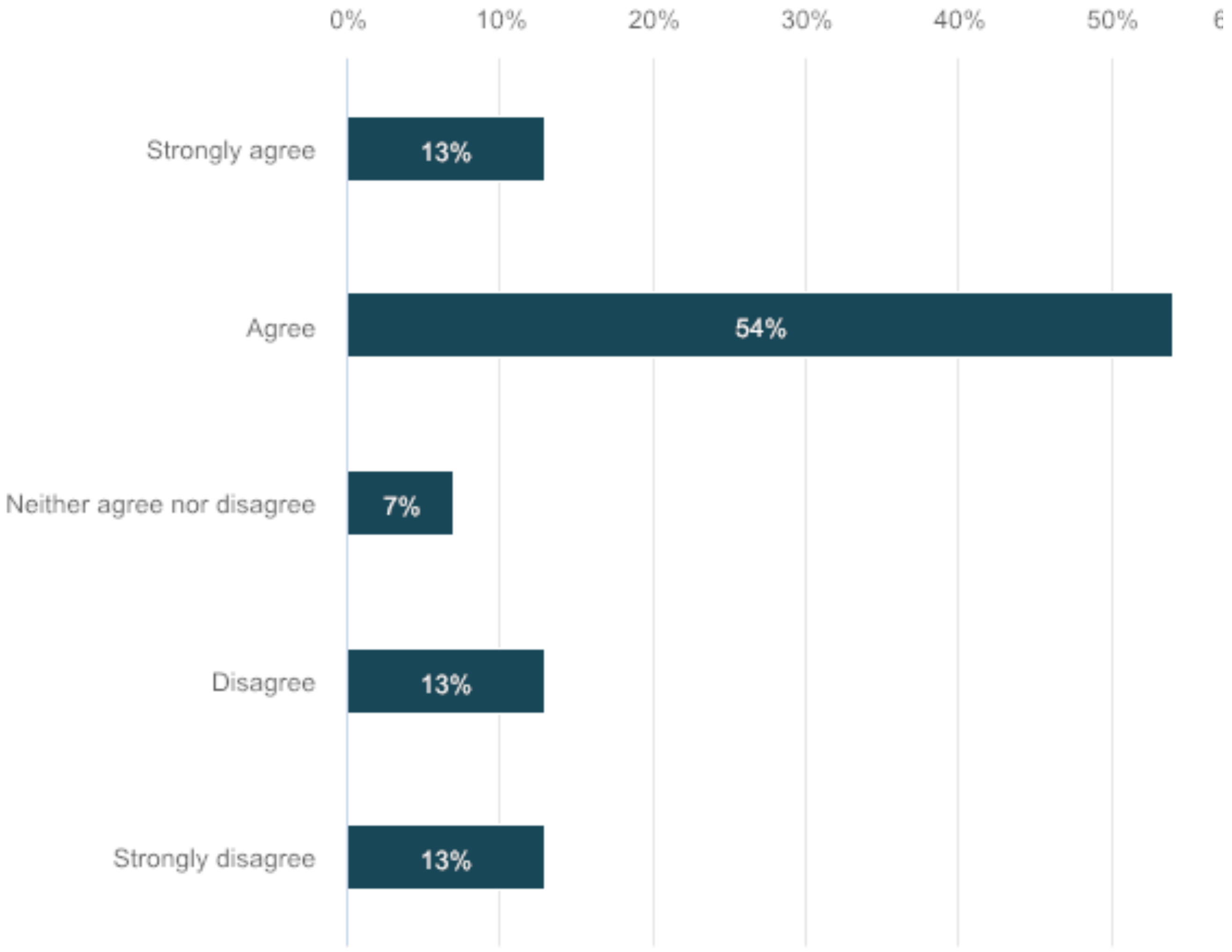

	n	Percent
Strongly agree	2	13.33%
Agree	8	53.34%
Neither agree nor disagree	1	6.67%
Disagree	2	13.33%
Strongly disagree	2	13.33%

3. "Any dose of radical radiation for lung cancer, after initial radical radiotherapy to the thorax or surrounding tissues for any tumour histology, provided there is overlap of the initial treatment at the 50% isodose line of the re-treatment in either the PTV or the OARs"

Number of respondents: 15

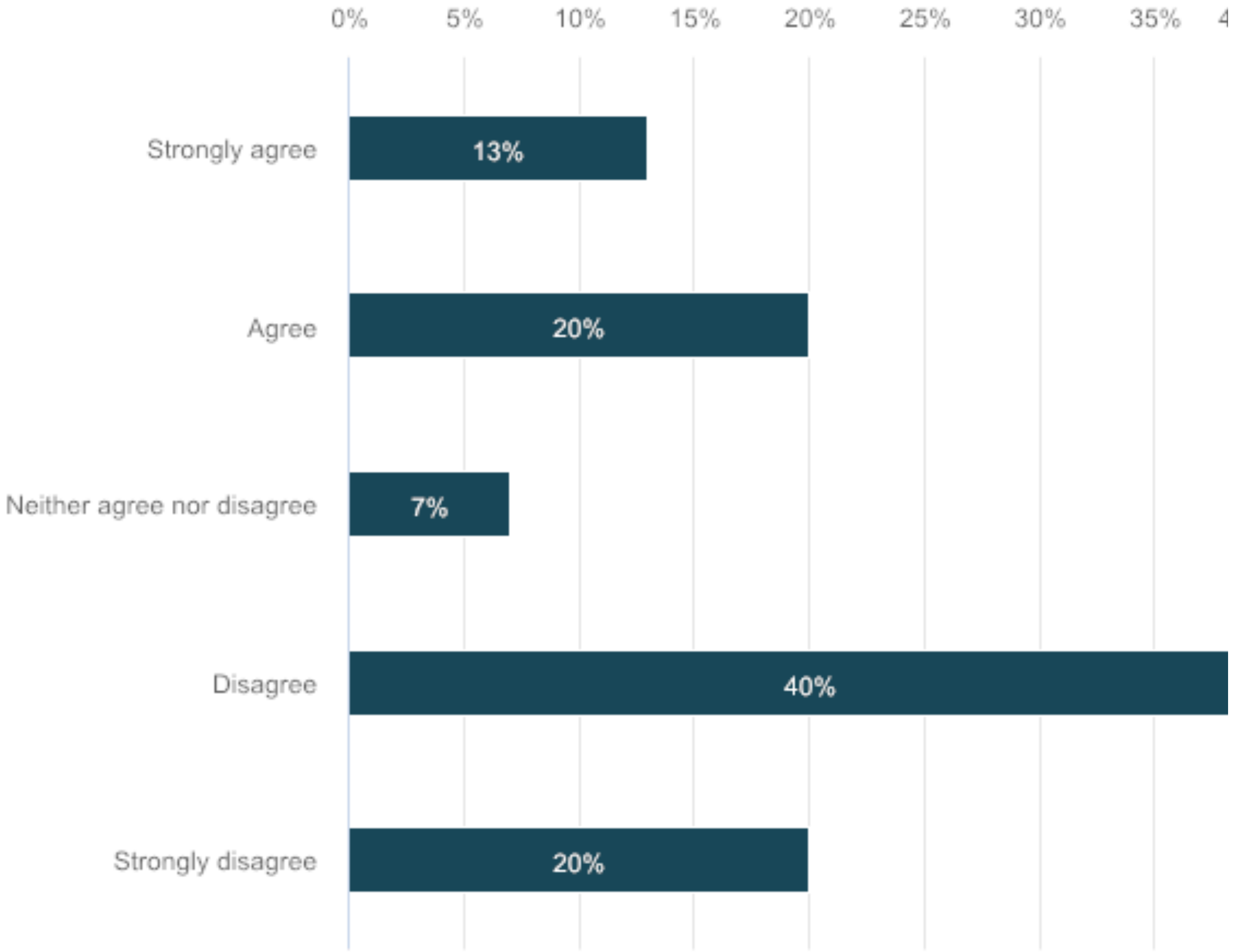

	n	Percent
Strongly agree	2	13.33%
Agree	3	20%
Neither agree nor disagree	1	6.67%
Disagree	6	40%
Strongly disagree	3	20%

4. Please add any comments about either definition here:

Number of respondents: 12

Responses
It's critical to have an accurate definition of "re-irradiation". To select any arbitrary values is, well of course, simply arbitrary. As such, it should include any over-lap, from which to start and understand "how much" we can overlap..
definition may be contingent on the particular OAR
This is a difficult one. I think a distinction needs to be made regarding re-irradiation of local recurrence and re-irradiation of previously treated area due to presence of a new, separate malignant pathology.<br><br><br><br>"Any overlap" will creat all sorts of problems. Low dose wash regions may be very large for example.
the tissue where there is overlaps is of importance (nd volume etc)
Many centres are now using VMAT for lung treatment. This means that there is a low-dose bath throughout the thorax on a given axial slice, and even two treatments that are far away but on the same plane would meet both definitions (since in the second one, the 50% of the second plan would overlap with a 2 Gy isodose line from the other side). Perhaps a better definition would be: a second course of radiation wherein contribution from a previous course of radiation leads to a cumulative dose that is higher than the prescription for the second course, or exceeds standard dose constraints when considered without repair.
50% arbitrary but acceptable
I would not restrict to level of overlap; as could be quite toxic for 25% overlap with current full dose for example for OARs
I think even if there is no overlap with dose to the PTV or serial OARs the lung, one of the key OARS, will need to be considered for any re-irradiation.
Suggest consider something like: "...provided there is significant cumulative dose overlap in the thorax"<br><br>The 50% isodose is too dependent on the prescribed dose/fractionation<br><br>I suggest dose overlap in the thorax because some plans put a lot of dose outside the PTV and into regions not classically considered as OARs (e.g. chest wall)
The 50% isodose line is meaningless without knowing the absolute dose and the OARs affected
"Any overlap" does not solve the issue: 1st SBRT in the upper lobe and 2nd SBRT in the lower lobe will not result in any dose overlap but might still increase the risk of pneumonitis.<br>The 50% isodose line is arbitrary and not based on clinical evidence.<br><br>One proposal:<br>re-irradiartion: overlap of high-doses; high doses being defined as accumulated doses > then a radical dose in a single course (in lung cancer >

60 - 66Gy)<br>repeat RT: two RT courses with dose exposure in the same organ (w/o necessary overlap)
The suggestion with the overlap definition containing OARs is that it may need modification to include “OARs previously irradiated.”

5. Radical re-irradiation can be considered for suspected new lung primaries with minimal overlap with previous radiotherapy fields.
Number of respondents: 15

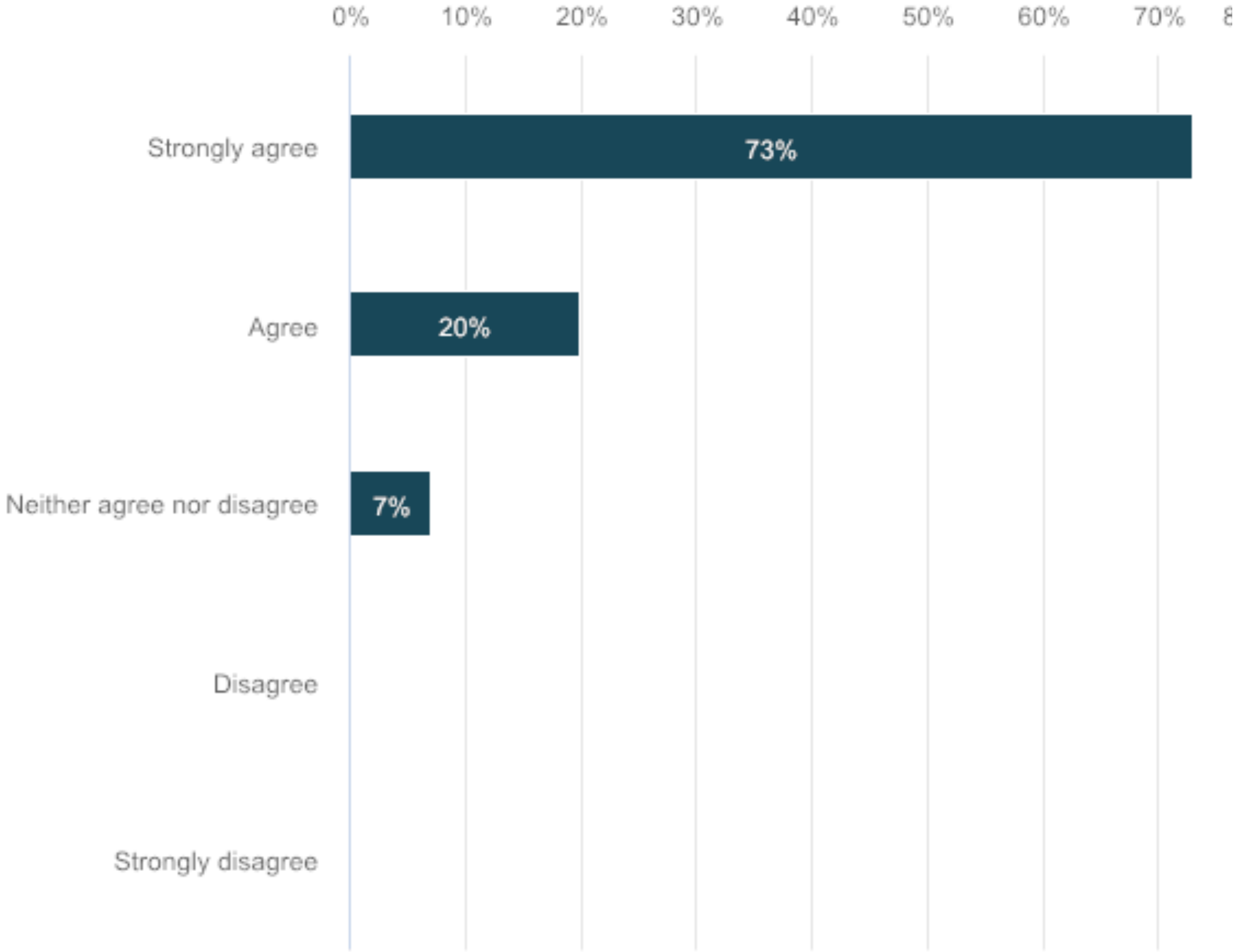

	n	Percent
Strongly agree	11	73.33%
Agree	3	20%
Neither agree nor disagree	1	6.67%
Disagree	0	0%
Strongly disagree	0	0%

6. Radical re-irradiation can be considered for lung tumours which develop new nodal disease after an initial course of radiotherapy only to the primary tumour (therefore minimal overlap).
Number of respondents: 15

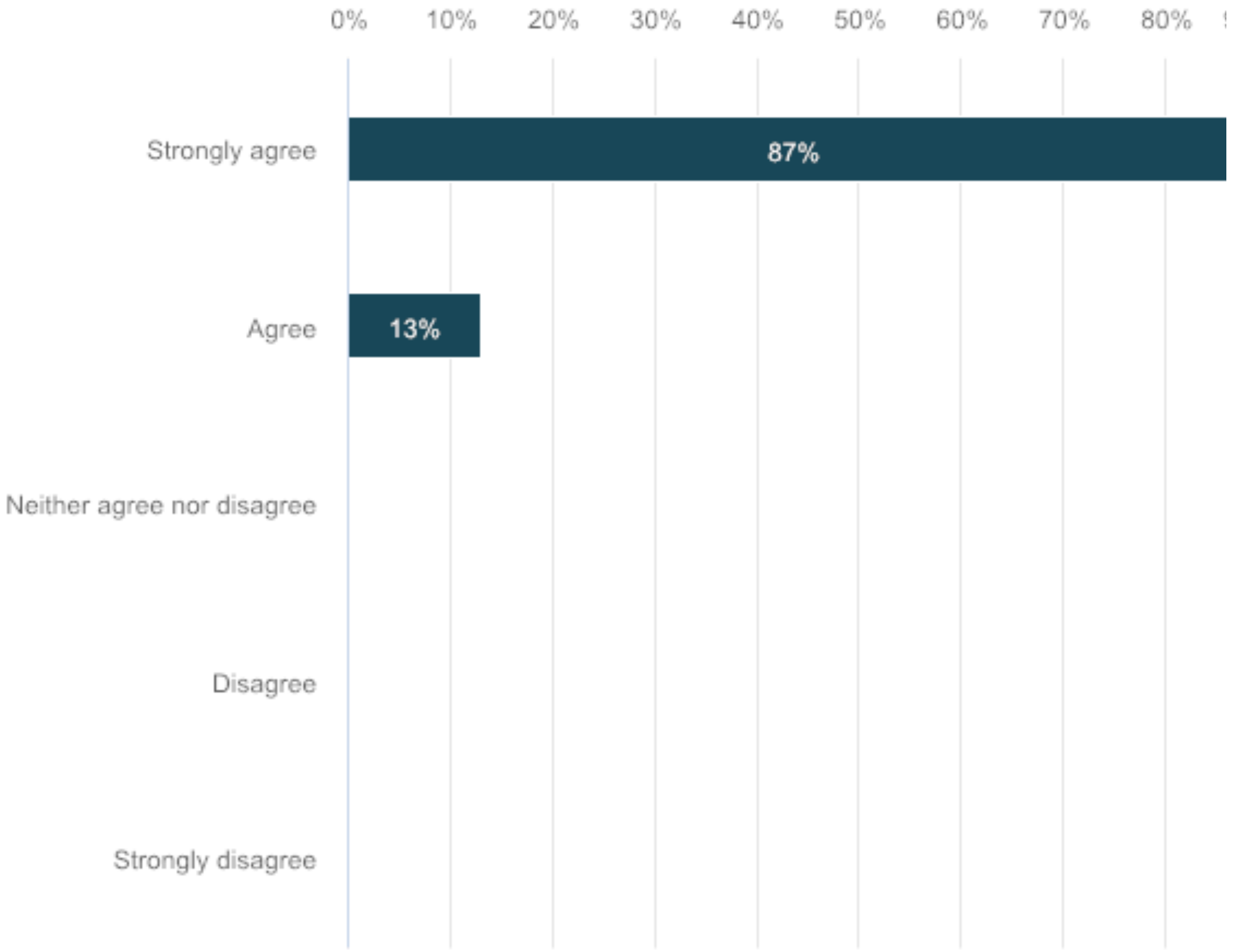

	n	Percent
Strongly agree	13	86.67%
Agree	2	13.33%
Neither agree nor disagree	0	0%
Disagree	0	0%
Strongly disagree	0	0%

7. Radical re-irradiation can be considered where a lung tumour relapses locally (or develops a suspected second primary tumour with >50% overlap with the original primary tumour), but low overlap with serial structures in the thorax.
Number of respondents: 15

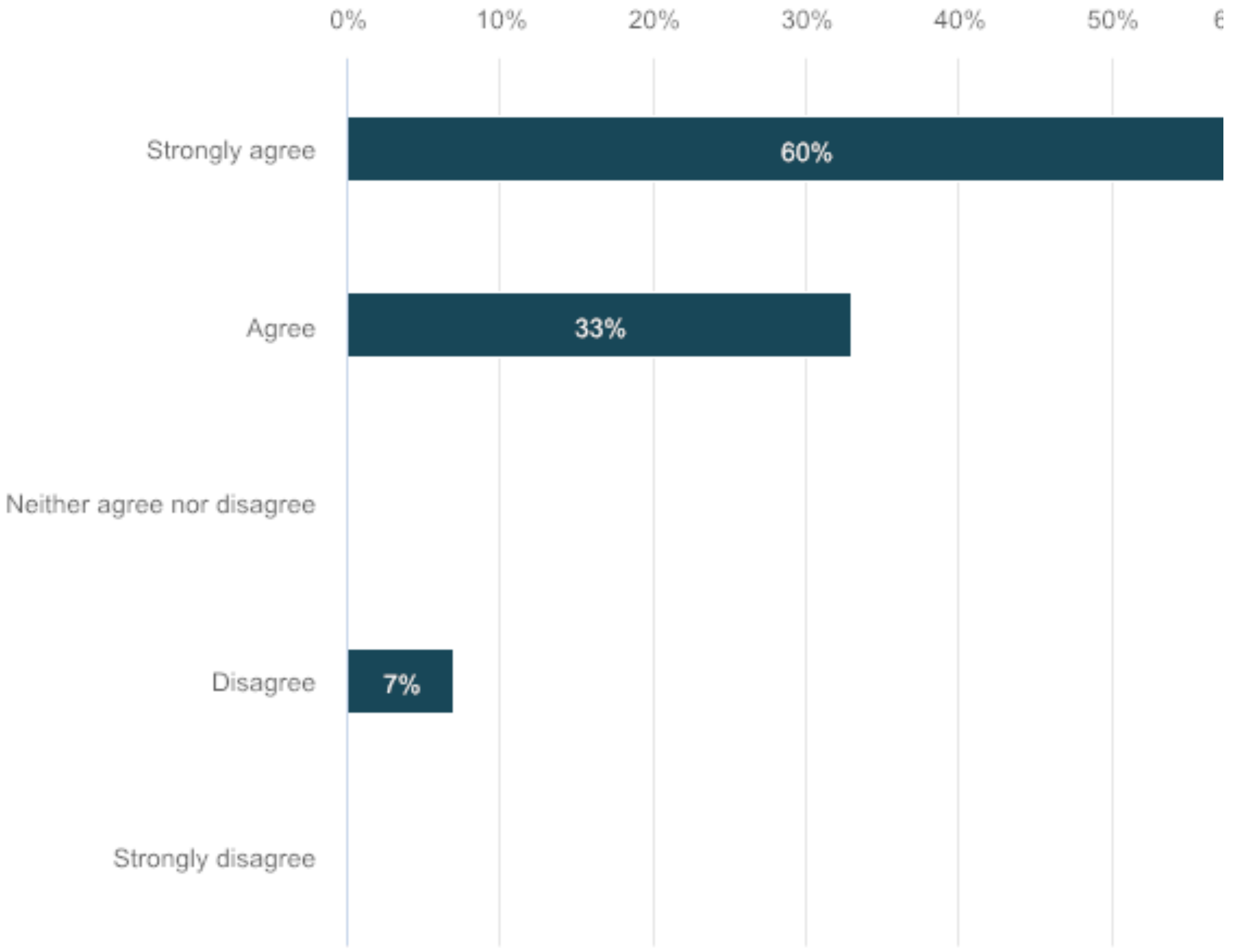

	n	Percent
Strongly agree	9	60%
Agree	5	33.33%
Neither agree nor disagree	0	0%
Disagree	1	6.67%
Strongly disagree	0	0%

8. Alternative treatment (e.g. systemic treatment) is preferable to radical re-irradiation, where a lung cancer has relapsed in both the previously irradiated primary tumour and nodes.
Number of respondents: 15

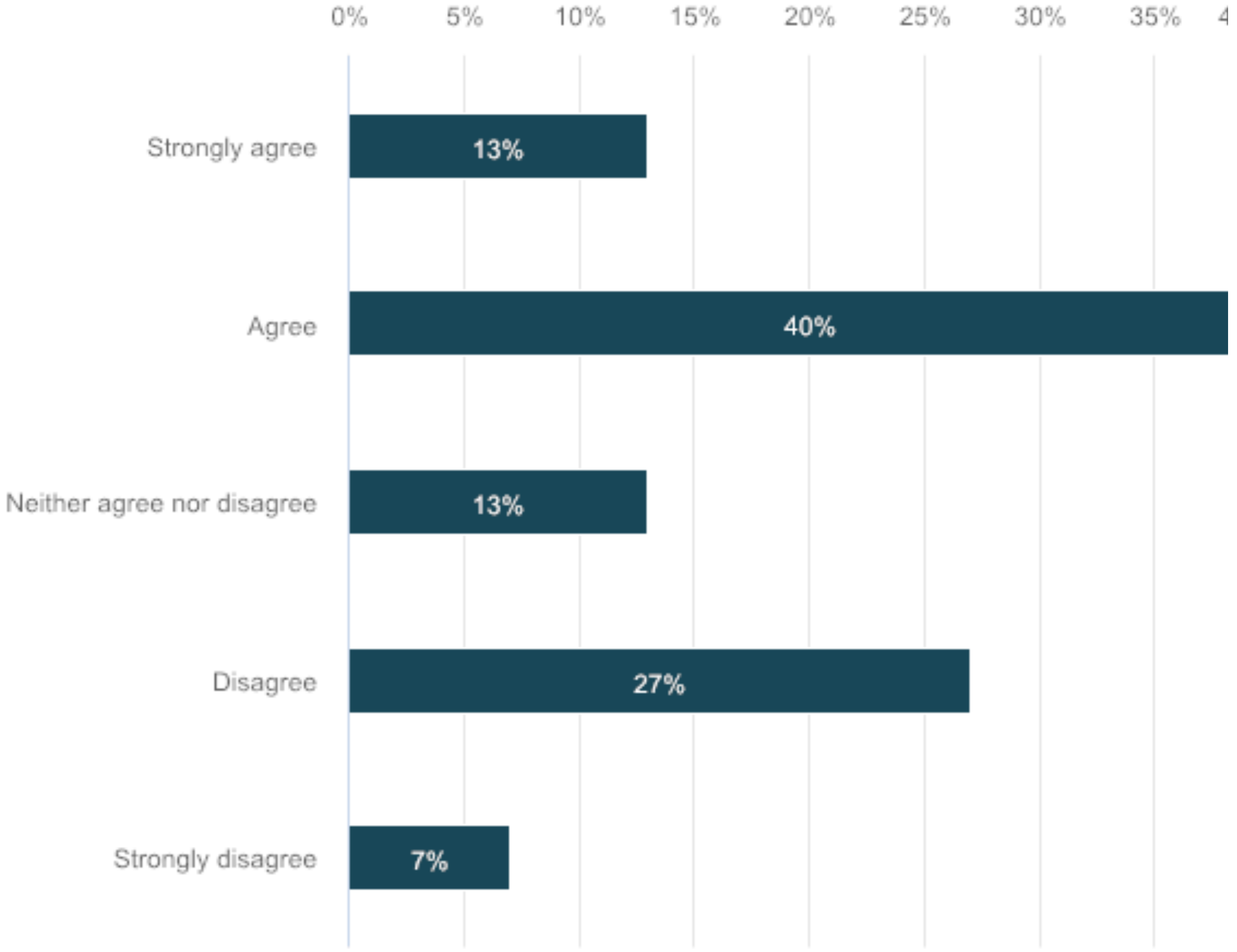

	n	Percent
Strongly agree	2	13.33%
Agree	6	40%
Neither agree nor disagree	2	13.33%
Disagree	4	26.67%
Strongly disagree	1	6.67%

9. Please justify your answer to question 8
Number of respondents: 14

Responses
Re-irradiation of central structures is quite unsafe, would usually be unable to deliver a definitive dose, and thus be a high-risk palliative procedure.
Loco-regional failure should be treated with local therapy if technically feasible. There is however an argument that if the cancer has a driver mutation or is strongly PDL1 +ve (>50%), then systemic therapy should be offered instead
this is mostly systemic disease. No data on long-term OS in this situation.
I would not be keen to re-irradiate both tumour and nodes. I would avoid the term "alternative", since that often refers to alternative medicine.
Newer modalities ie targeted therapy and immunotherapy are more widely available now
Risk of re-irradiation to previously treated nodes and primary is too high
Can still do well if re-irradiated (we have just submitted ESTRO abstract showing that patients who had re-irradiation and repeat radiotrehrapy had exactly same survival as those treated with 1 radikotherapy course using propensity matched analysis)
It depends on patients PS and volume of initial and recurrent disease. If large volume and good PS with a decent DFS then I would give chemo +/- IO first and then consider re-irradiation. If poor PS then I would consider re-irradiation but not sure I would give a radical dose.
Re-RT may still be the preferred option depending on risks of re-RT, time since previous RT, available systemic options and likelihood of response/prior response to systemic therapy
To some extent, the rationale for reirradiation will depend on the extent and duration of previous response. If the disease has not been eradicated first time around, why should it be more likely to be eradicated with a second course. Further, the number of systemic options is now much greater than a decade ago.
Systemic Tx would not be a treatment with a curative potential at the patient should be offered the possibility of such, openly consented and informed about the added risk of toxicity
Re-irradiation of lung and mediastinum to radical doses in the setting of recurrent primary and nodal disease may carry more risk to justify the benefits. Systemic treatment alone in this situation would be preferred, with possibly consolidate RT following evaluation of response.
With relapse in the primary and nodes it will challenging to get necessary dose in, considering the surrounding OARs.
Depends on burden of disease, performance status, and actionable mutations and I/o options. Especially if latter meds are covered. Chemo alone isn’t a fantastic option

10. Alternative treatments (e.g. systemic therapy) are preferred to radical re-irradiation to the primary lung cancer where the lung tumours have relapsed both locally and with oligo-metastatic disease (less than 3 metastases, all mets treatable with radical radiotherapy).
Number of respondents: 15

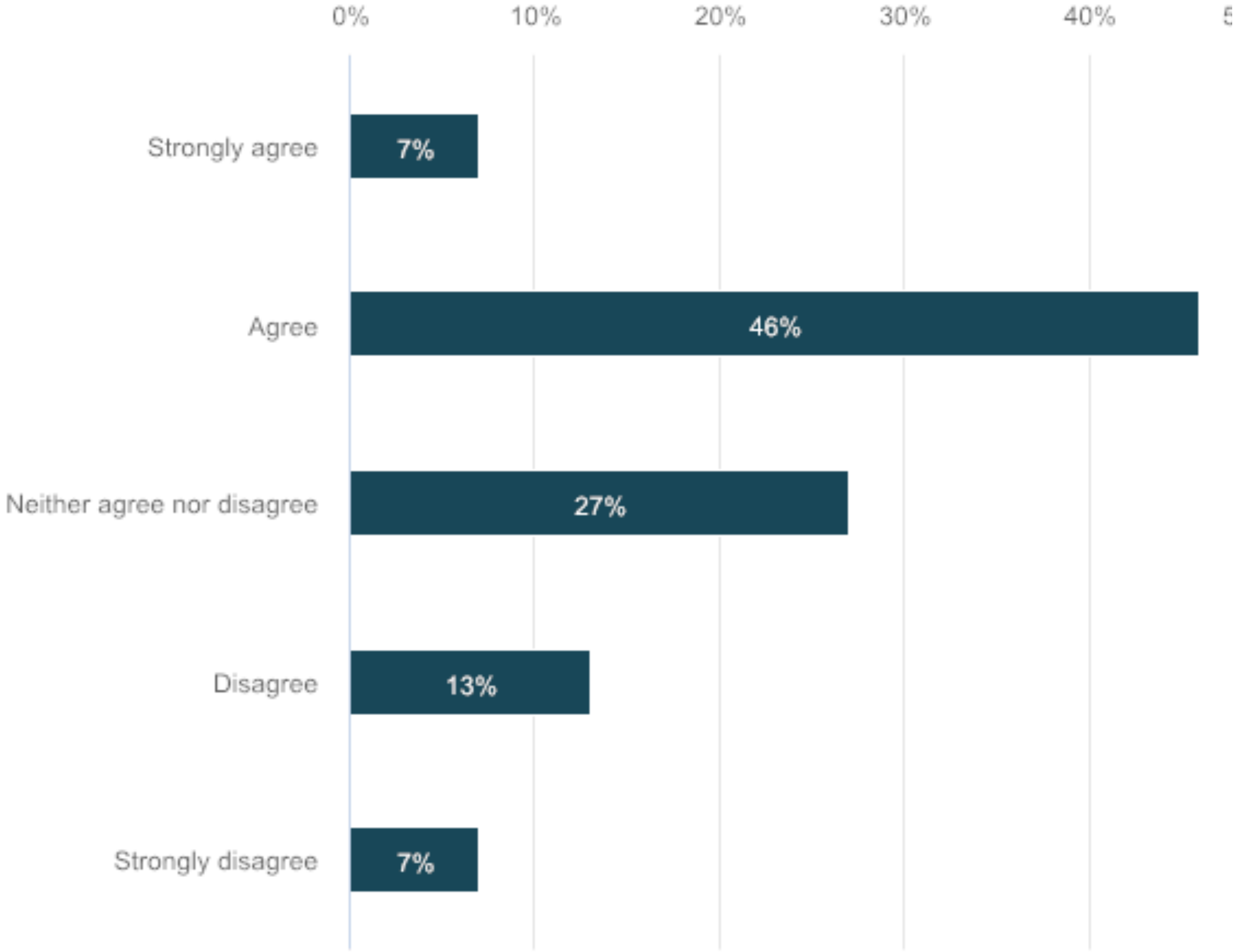

	n	Percent
Strongly agree	1	6.67%
Agree	7	46.66%
Neither agree nor disagree	4	26.67%
Disagree	2	13.33%
Strongly disagree	1	6.67%

11. Please justify your answer to question 10.
Number of respondents: 12

Responses
There are many times with re-irradiation is still an option in this setting.
No evidence for SBRT to oligmets in context of a relapsed primary tumour too. Thi should only be an option in context of a clinical trial (which is unlikely to ever recruit enough patients, unless global)
this is systemic disease. No data on long-term OS in this situation.
Either strategy warranted and unclear which is preferred. No strong data here. However multid discussion warranted especially in light of bio marker status and patient preference
I could potentially justify a small local relapse + oligomets if all lesions could receive SABR, but patient would need to be extremely fit. I have not encountered this in clinical practice yet. I would only propose this with a single oligomet + local relapse
Would give trial of systemic therapy first if fit enough to make sure that they don't progress outside of the local relapse and 3 oligo mets. If not fit or very small volume disease then could consider radical RT/SABR to all sites.
Would offer radical re-RT depending on risks, may consider initial systemic therapy to gauge biological behaviour and select patients, would distinguish between different systemic therapies i.e. chemotherapy vs. targeted therapy It is not a question of one or the other - initial treatment with systemic therapy does not preclude subsequent (re)RT
Same response as 9. Would reserve irradiation of metastatic disease for sites of oligoprogression
Systemic Tx would not be a treatment with a curative potential at the patient should be offered the possibility of such, openly consented and informed about the added risk of toxicity
Provided radical doses can be safely delivered to all metastases, re-RT to the lung and radical RT to the other metastases would be reasonable.
Systemic disease requires a systemic therapy as part of the treatment approach
If there is disease beyond what has been previously radiated then I would almost certainly recommend systemic therapy over radical re-rt and sabr

12. Alternative treatments (e.g. systemic therapy) are preferred to radical re-irradiation to the primary lung cancer where the lung tumours have relapsed both locally and with widespread metastatic disease.

Number of respondents: 15

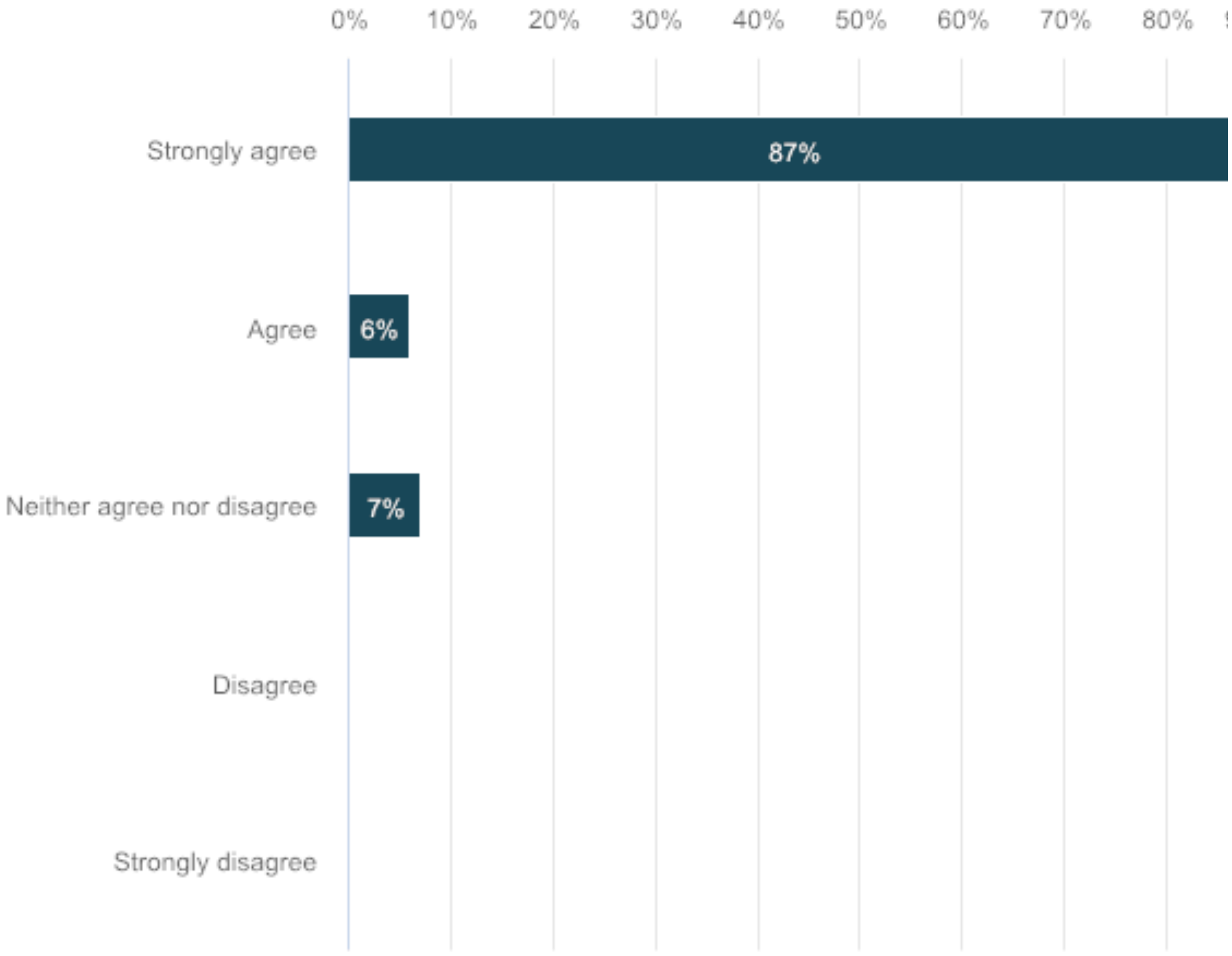

	n	Percent
Strongly agree	13	86.67%
Agree	1	6.66%
Neither agree nor disagree	1	6.67%
Disagree	0	0%
Strongly disagree	0	0%

13. Please justify your answer to question 12.

Number of respondents: 13

Responses
Run-away metastatic disease is unlikely to benefit from any form of local therapy
distant disease drives survival, therefore increasing toxicity from repeat local RT is not warranted since no benefits
Clear evidence of advanced stage IV diease. Local RT should only be given with palliative intent
really systemic disease
If disease widespread repeat radical rt risks may not be warranted
no perceived benefit for local re-irradiation
Only would consider re-irradiation to a palliative dose in this situation if symptomatic either before systemic therapy or if PS precluded systemic therapy and symptomatic
Standard therapy for widespread metastatic disease
Would use low dose palliative irradiation to relapsed disease for symptomatic relief
Re-RT would be justified with palliative intent, there is not indication for radical RT in this setting
In the context of extensive distant metastases, there is no role for re-irradiating the lung tumour.
No evidence for a radiation only approach here.
As above

14. Please add any additional comments here.

Number of respondents: 1

Responses
In some cases, salvage surgery may be a preferred option - should be considered and discussed with an experienced thoracic oncology surgeon

15. In general, patients should have an ECOG performance status (PS) of 0 - 2 to be considered for radical dose re-irradiation, with exceptions being made for selected PS 3 patients (e.g. SABR re-irradiation, or PS 3 due to non-respiratory issues).

Number of respondents: 15

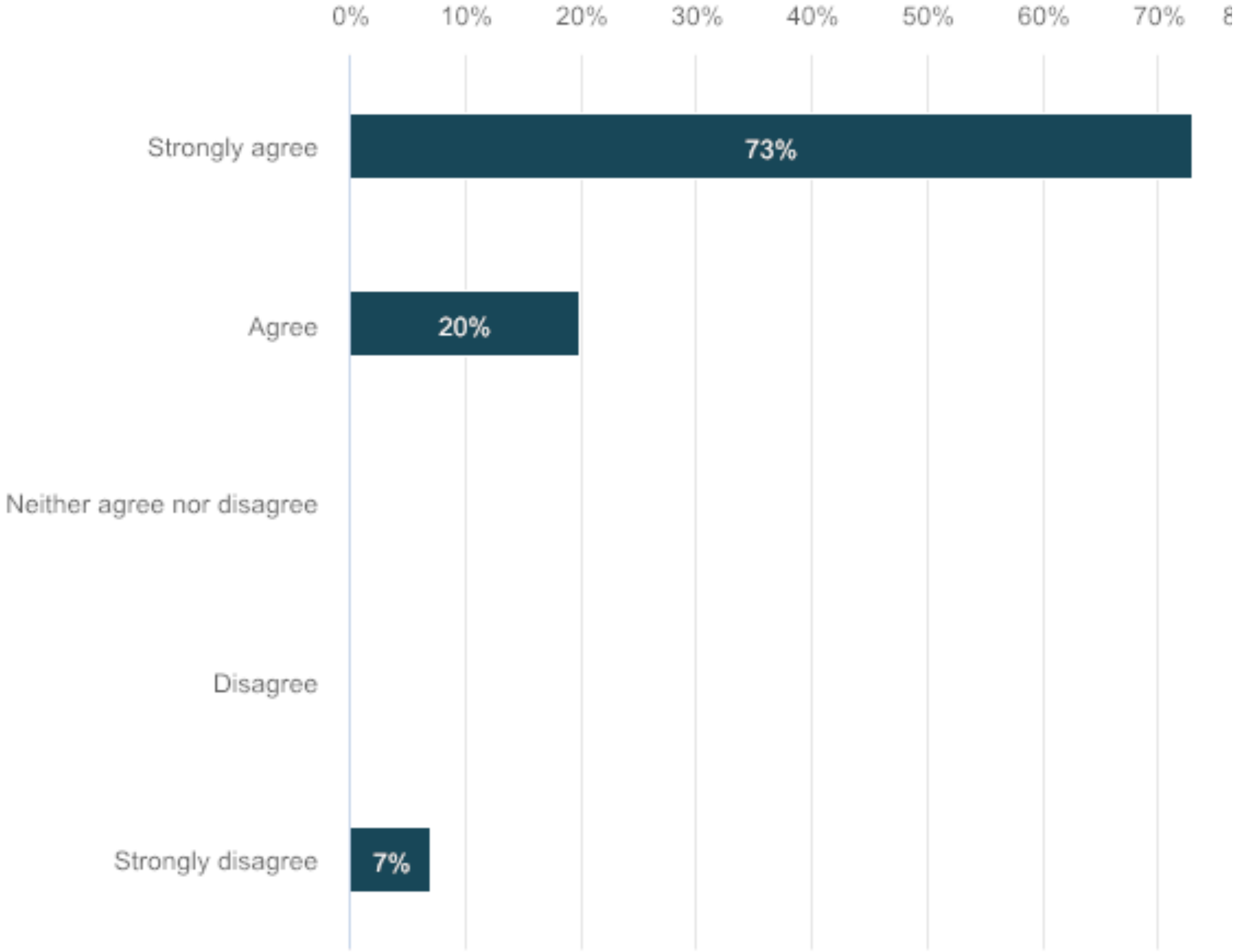

	n	Percent
Strongly agree	11	73.33%
Agree	3	20%
Neither agree nor disagree	0	0%
Disagree	0	0%
Strongly disagree	1	6.67%

16. Re-irradiation should be avoided in patients with interstitial lung disease.

Number of respondents: 15

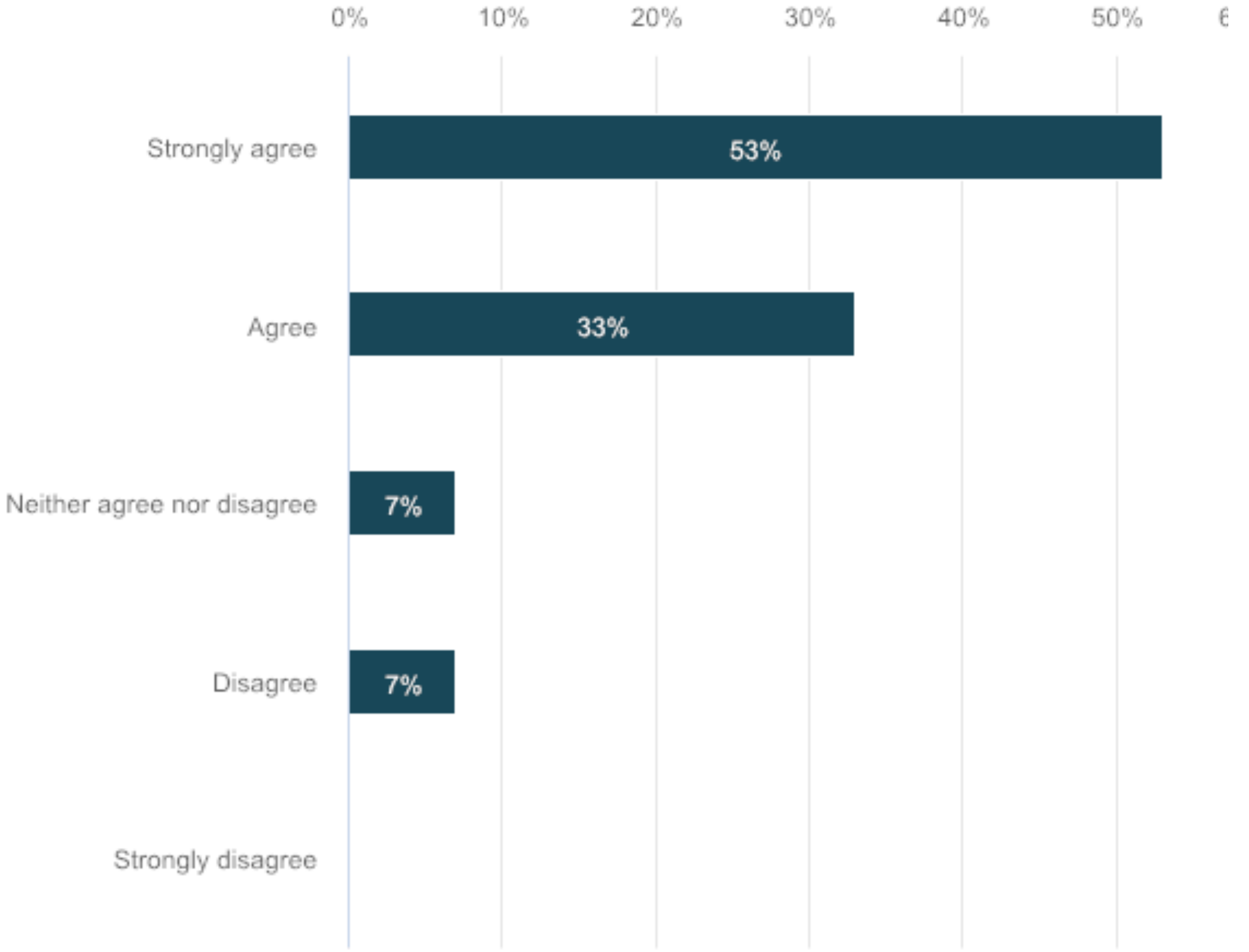

	n	Percent
Strongly agree	8	53.33%
Agree	5	33.33%
Neither agree nor disagree	1	6.67%
Disagree	1	6.67%
Strongly disagree	0	0%

17. Re-irradiation should be avoided with concurrent or recent (<3 months) use of drugs associated with pneumonitis (e.g. tyrosine kinase inhibitors, gemcitabine, immune checkpoint inhibitors).

Number of respondents: 15

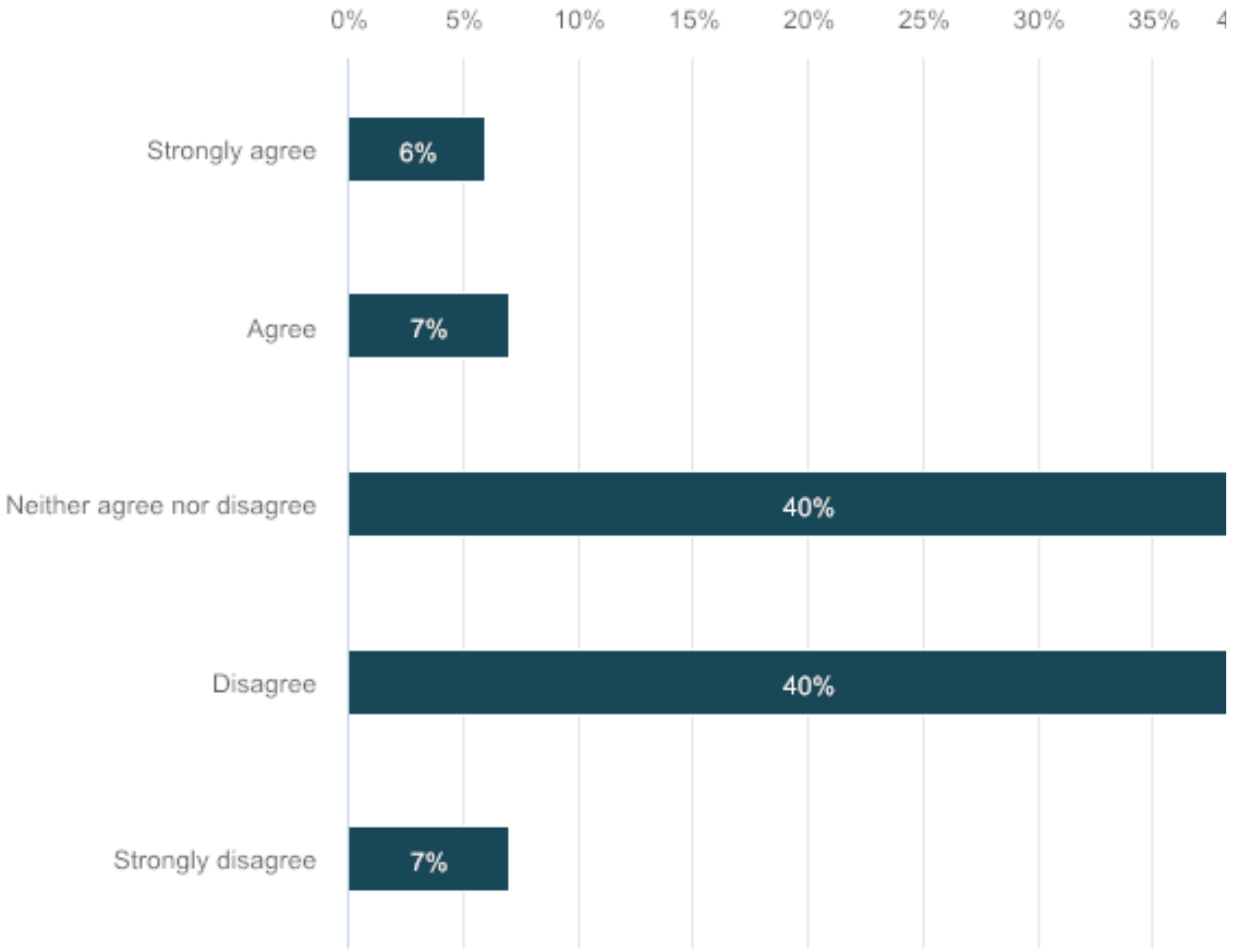

	n	Percent
Strongly agree	1	6.66%
Agree	1	6.67%
Neither agree nor disagree	6	40%
Disagree	6	40%
Strongly disagree	1	6.67%

18. Re-irradiation should be performed cautiously with patients who developed grade 3 or higher toxicity with their initial radiation treatment.
Number of respondents: 15

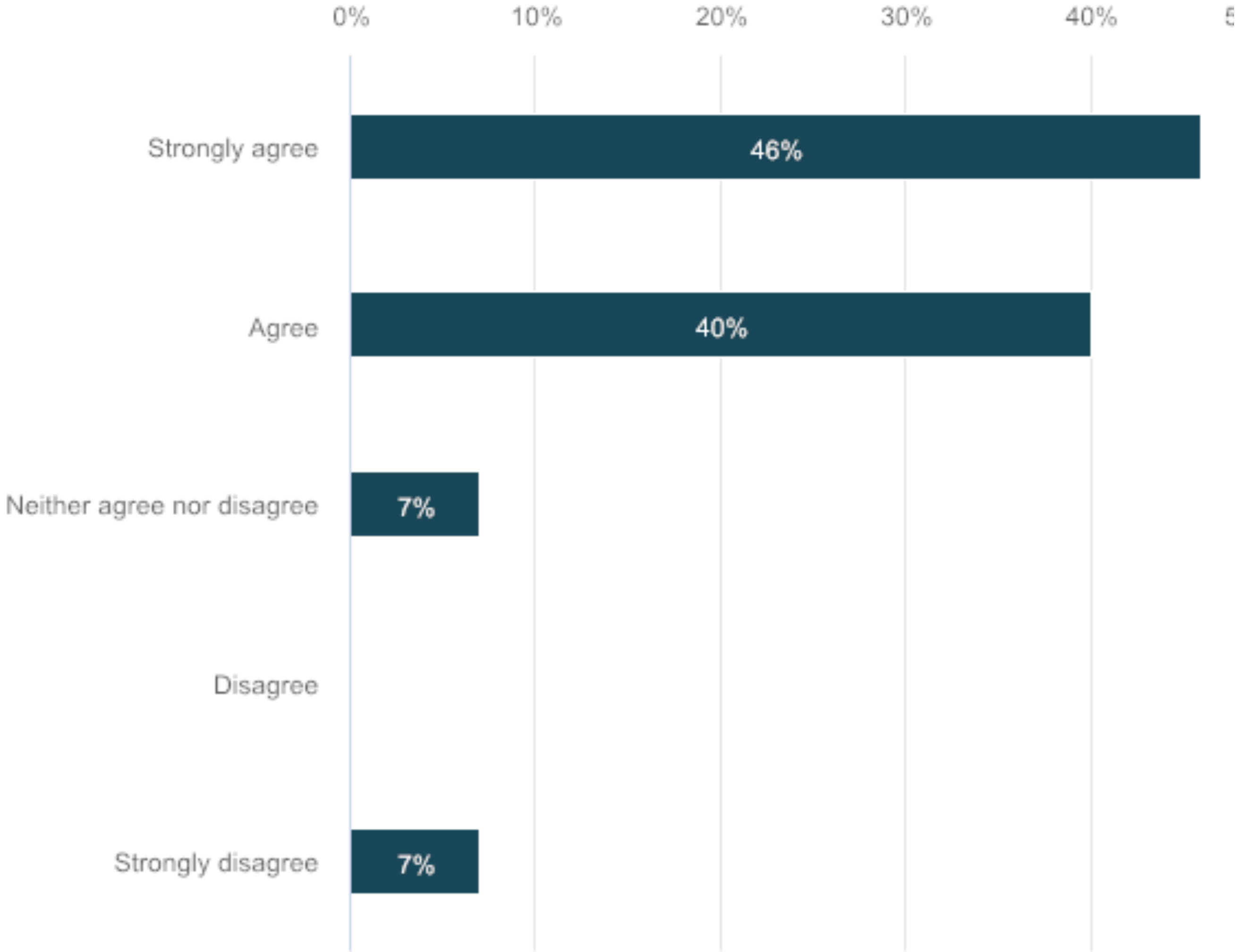

	n	Percent
Strongly agree	7	46.66%
Agree	6	40%
Neither agree nor disagree	1	6.67%
Disagree	0	0%
Strongly disagree	1	6.67%

19. Surgery should be considered in all appropriate patients being assessed for re-irradiation.
Number of respondents: 15

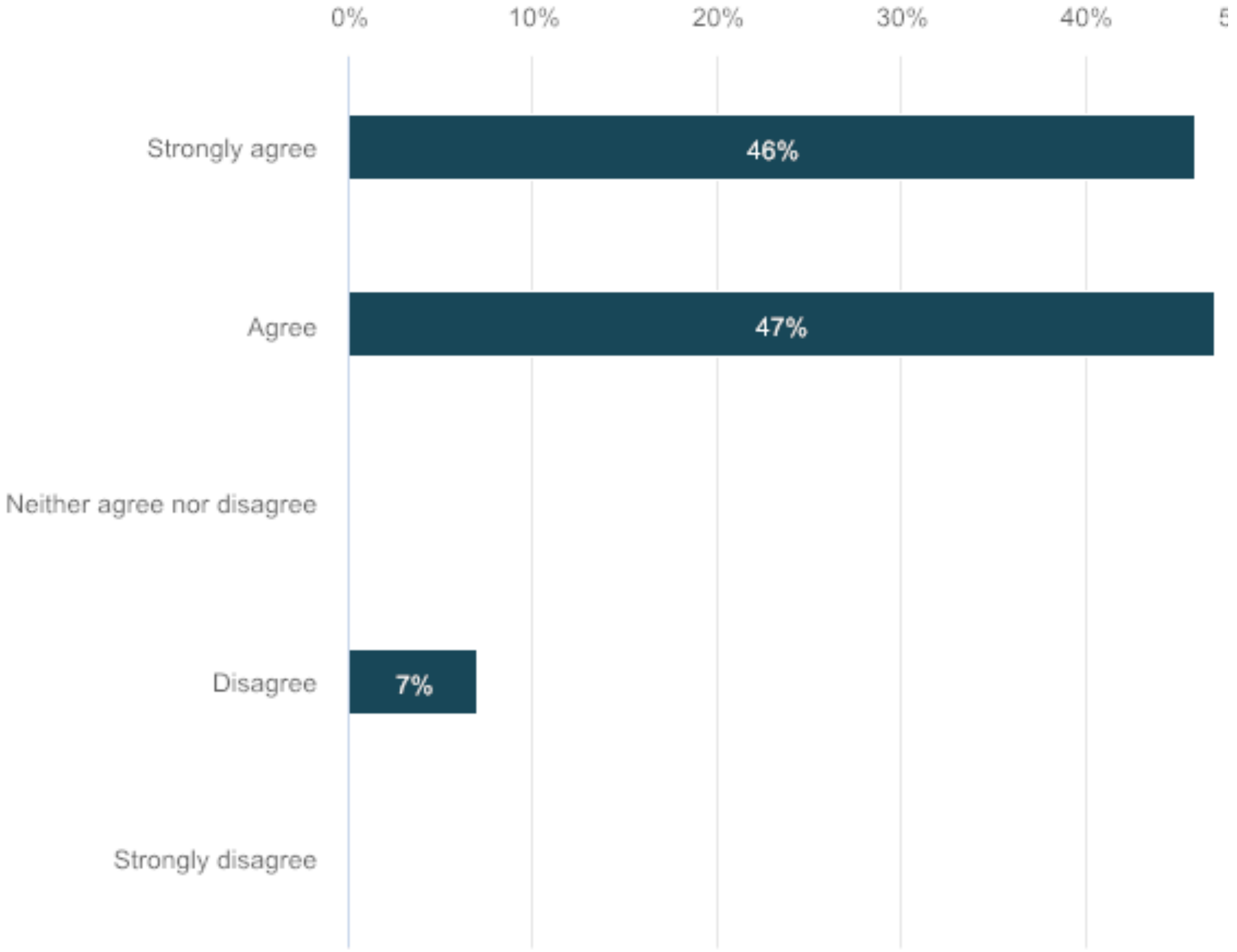

	n	Percent
Strongly agree	7	46.66%
Agree	7	46.67%
Neither agree nor disagree	0	0%
Disagree	1	6.67%
Strongly disagree	0	0%

20. In locally advanced recurrent lung cancer, where there is an increased likelihood of response to immunotherapy (e.g. PD-L1 >50%), immunotherapy may be preferable to high-risk radical re-irradiation.

Number of respondents: 15

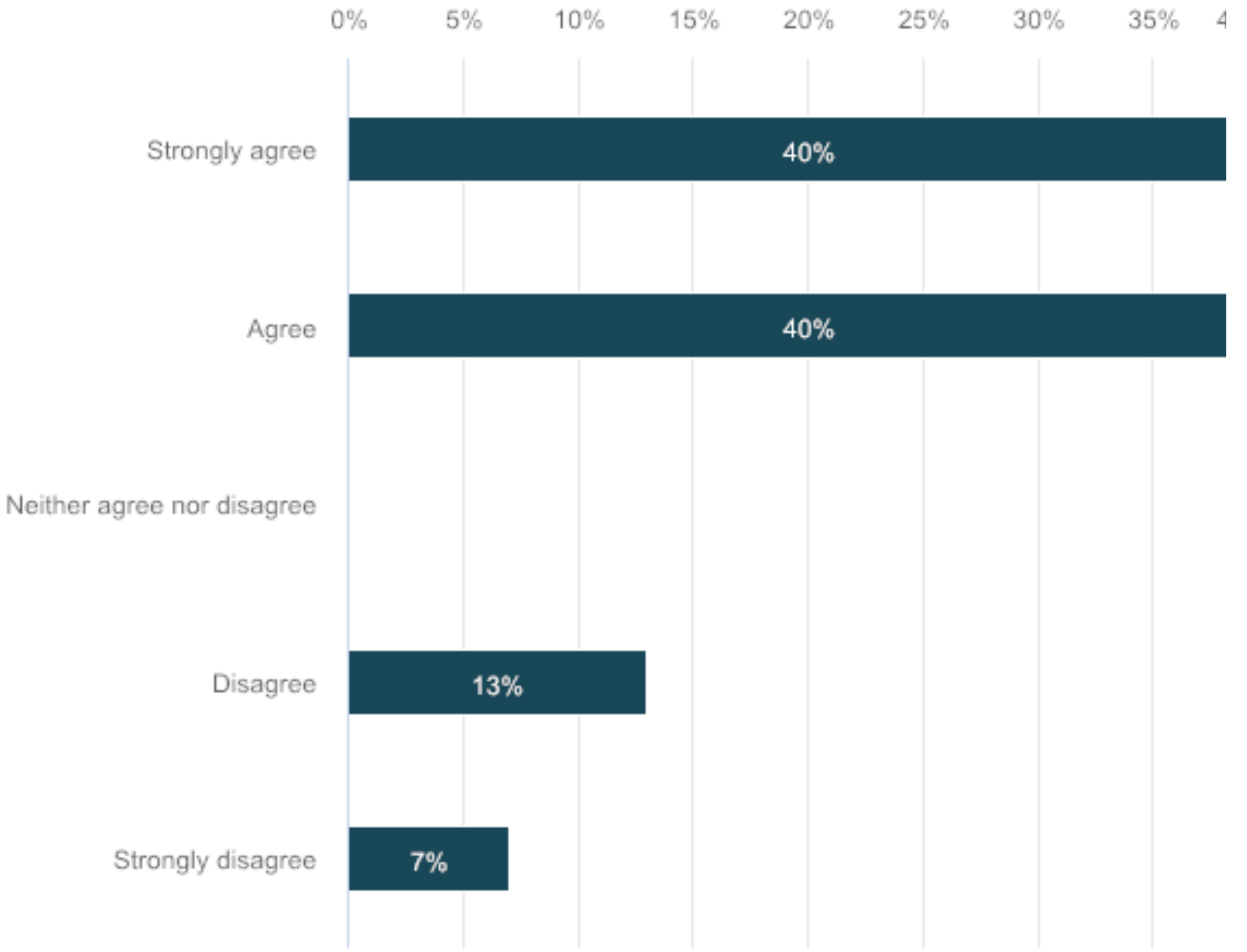

	n	Percent
Strongly agree	6	40%
Agree	6	40%
Neither agree nor disagree	0	0%
Disagree	2	13.33%
Strongly disagree	1	6.67%

21. In locally advanced recurrent lung cancer, where there is an actionable mutation (e.g. EGFR mutation, ALK fusion), targeted treatment may be preferable to high-risk radical re-irradiation.
Number of respondents: 15

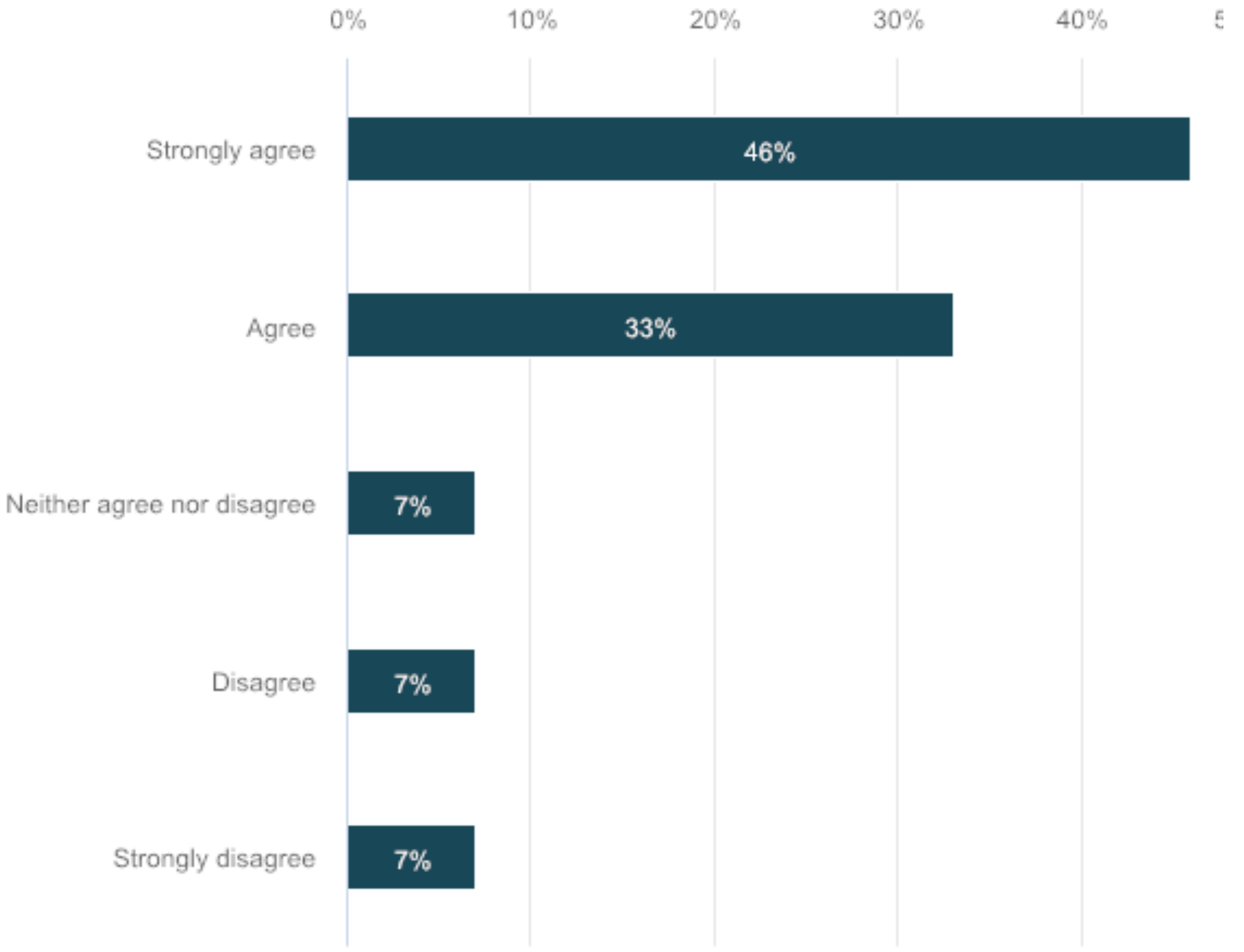

	n	Percent
Strongly agree	7	46.66%
Agree	5	33.33%
Neither agree nor disagree	1	6.67%
Disagree	1	6.67%
Strongly disagree	1	6.67%

22. The minimum interval between initial radiotherapy and re-irradiation where there is minimal overlap is:
Number of respondents: 15

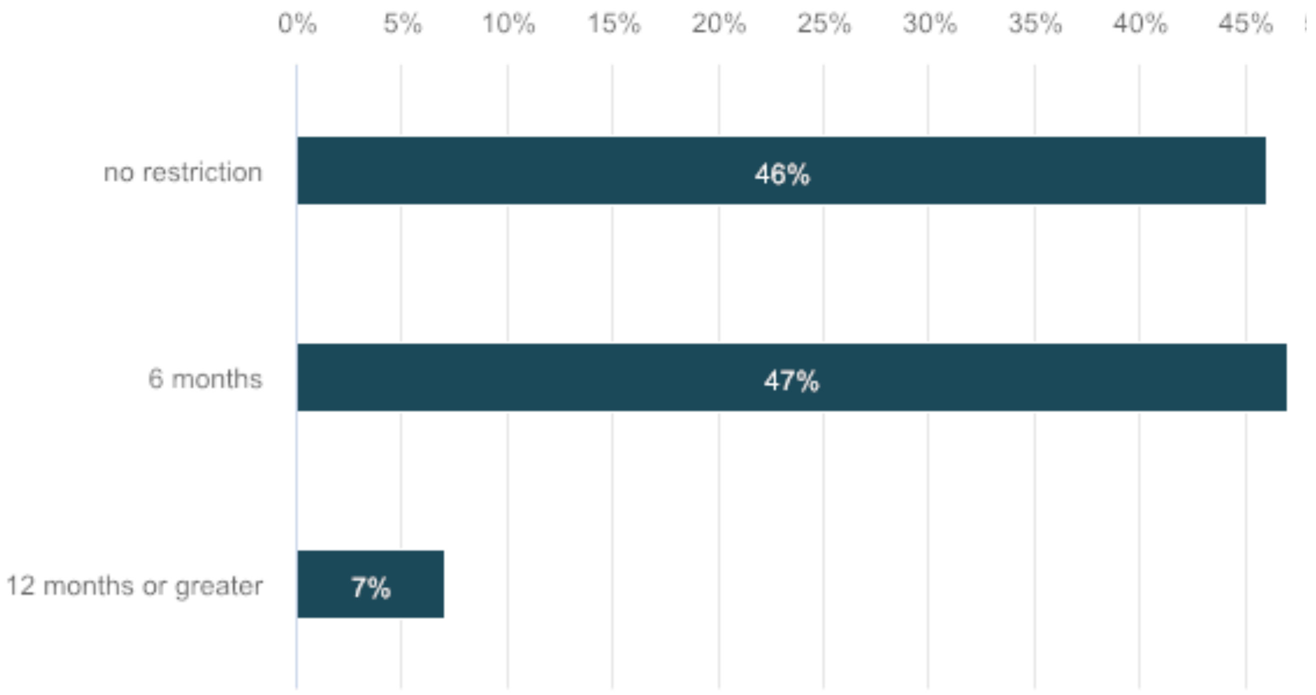

	n	Percent
no restriction	7	46.66%
6 months	7	46.67%
12 months or greater	1	6.67%

23. The minimum interval between initial radiotherapy and re-irradiation where there is an overlap at the 50% or greater isodose lines is:
Number of respondents: 15

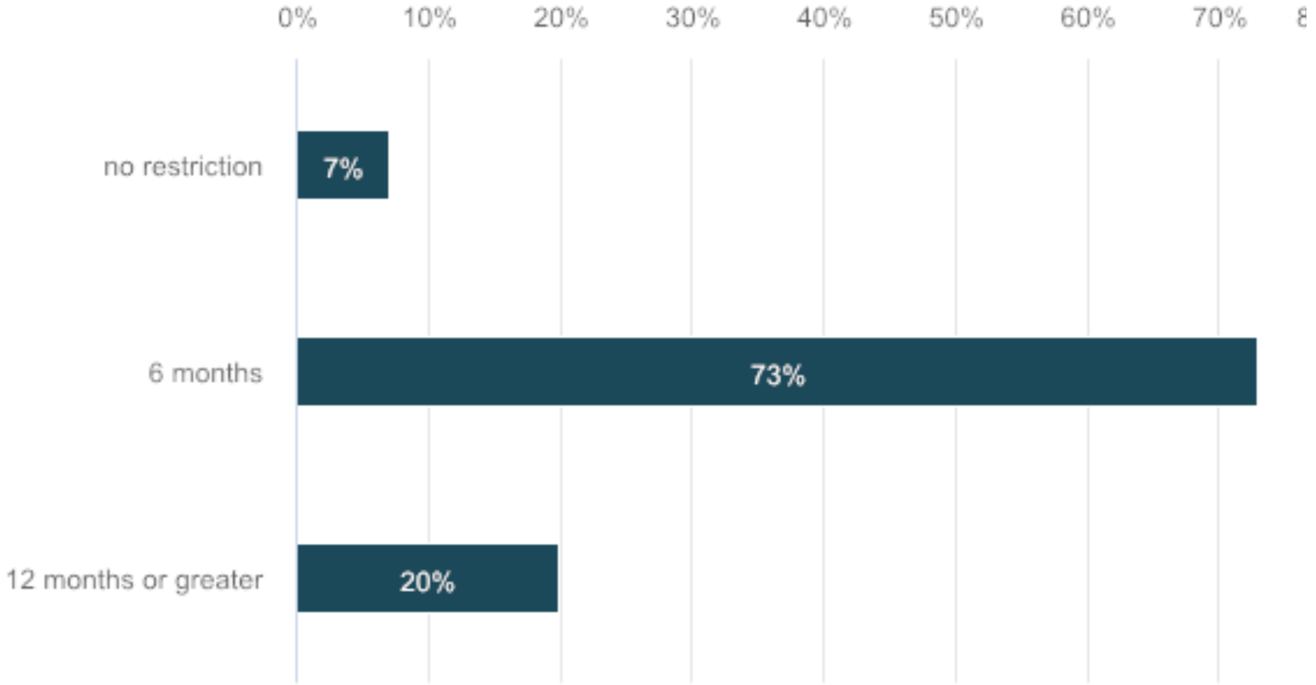

	n	Percent
no restriction	1	6.67%
6 months	11	73.33%
12 months or greater	3	20%

24. Please add any comments here:

Number of respondents: 10

Responses
6 months or 12 months is arbitrary. As long as OARs OK, re-irradiation should have no particular time limit.
Q 17. Where comes "3 months" from? This is highly dependend on the drugs.
#17: I disagree with the <3 months part. Holding systemic therapy for 3 months is a long time. Perhaps just leave it at "recent"<br><br>#20 might be too specific since chemo+immuno is often used. Perhaps just say "systemic therapy might be preferable".
Patients with ECOG 3 should not receive re-irradiation.<br><br>Im not so worried about recent checkpoint blockade or TKI (i.e. 2-3 months ago), but concurrent in the re-irradiation setting would be concerning. I cannot see when someone would have recent TKI, as this implies that the patient has already had metastatic disease and therefore re-irradiation of the primary relapse is not appealing. Therefore mostly basing this on ICI.<br><br>If patients had a G3 toxicity due to concurrent systemic therapy, then i would consider re-rads. If it was a RT related G3 toxicity then i would not give re-rads.<br><br>I would not recommend surgery for patients with nodal relapse after initial RT.
We show in ESTRO abstract, that these patients, even after accounting for lead time bias, pts with inter-radiotherapy time interval more than 12 m do well
Although at 6 months I would consider re-irradiation 12 or longer is preferable.
Qualifier for 23: assuming the indication is re-RT of the previously irradiated lesion. If the overlap is due to the planning technique, but the targets are different then the interval is not so relevant

Question 18: grade 3 esophagitis during the first course of RT should not influence the decision for reirradiation<br><br>Questions 20 & 21: IO and TKIs "may be preferable" - this will ver much depend on the actual risk, but in general a local approach is preferred in the situation of localized disease
Q21. Depends on the toxicity profile of re-irradiation.
Would not give re-rt with concurrent I/o or tki. However if these have been stopped for at least 2-4 weeks then I have no problem with rt (ie reason for my response to question #17

25. Please rate each of these pre-treatment investigations as either essential or non-essential to have before offering radical re-irradiation.
Number of respondents: 15

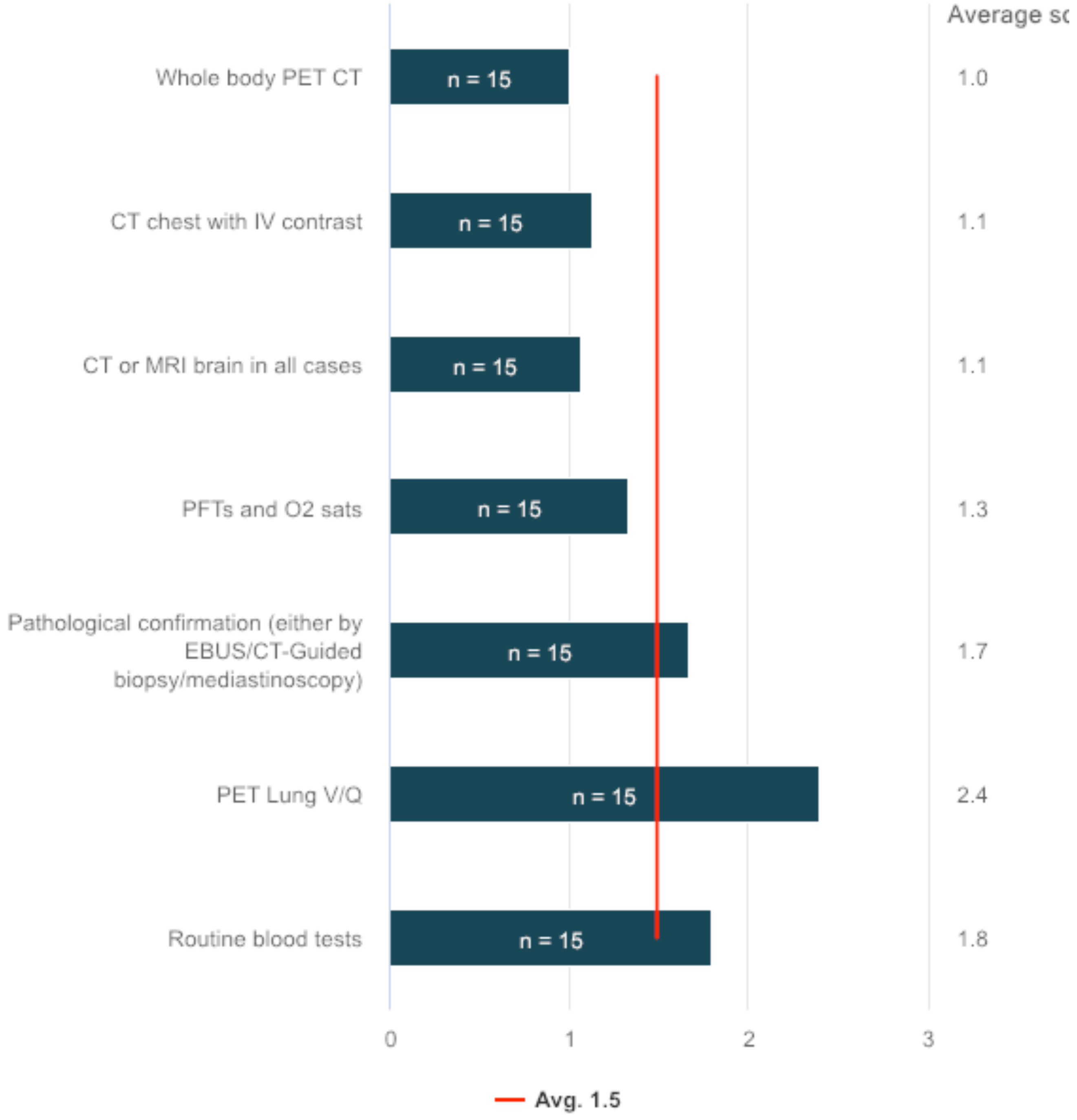

	Essential	Would like, but not essential	Not required	Average	Median

Whole body PET CT	100%	0%	0%	1	1
CT chest with IV contrast	86.67%	13.33%	0%	1.13	1
CT or MRI brain in all cases	93.33%	6.67%	0%	1.07	1
PFTs and O2 sats	66.67%	33.33%	0%	1.33	1
Pathological confirmation (either by EBUS/CT-Guided biopsy/mediastinoscopy)	33.33%	66.67%	0%	1.67	2
PET Lung V/Q	0%	60%	40%	2.4	2
Routine blood tests	46.66%	26.67%	26.67%	1.8	2

26. A CT or MRI Brain is required only with nodal disease, or T3/T4 disease recurrence.
Number of respondents: 1

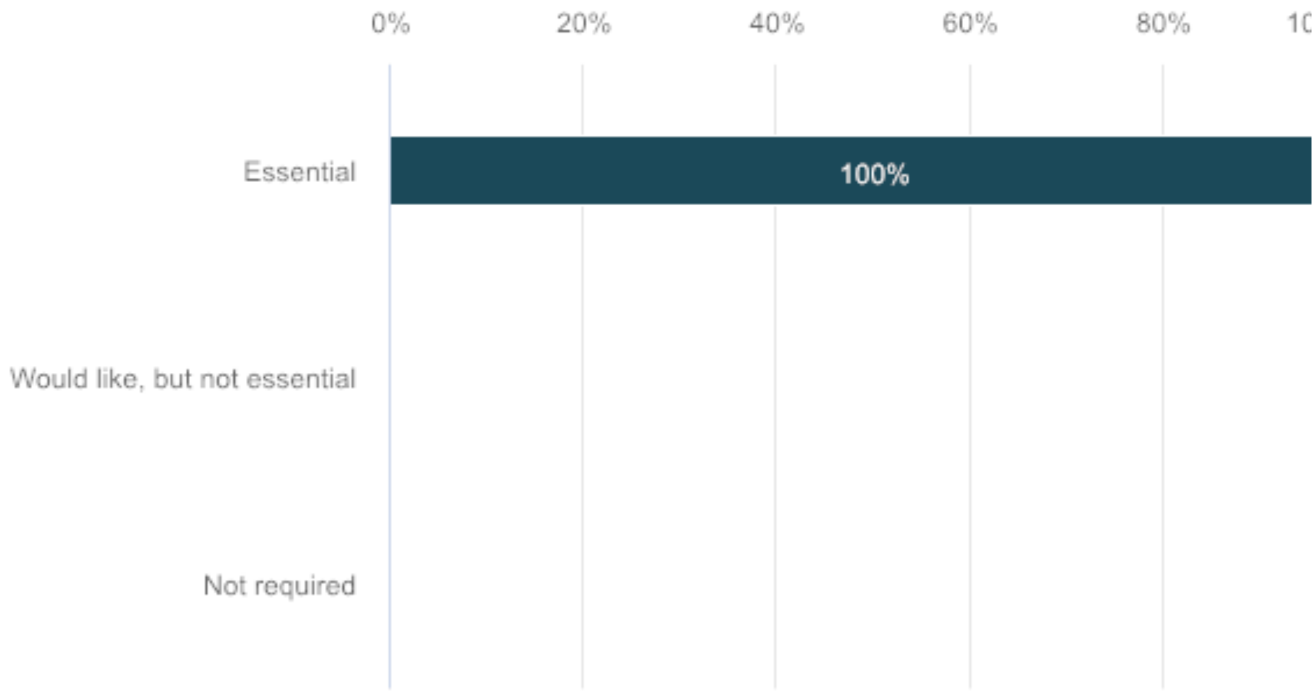

	n	Percent
Essential	1	100%
Would like, but not essential	0	0%
Not required	0	0%

27. For conventionally fractionated re-irradiation, in general the minimum lung function test values are:- DLCO greater than 30%- Forced expiratory volume in one second (FEV1) greater than 1 litre or 30%

Number of respondents: 15

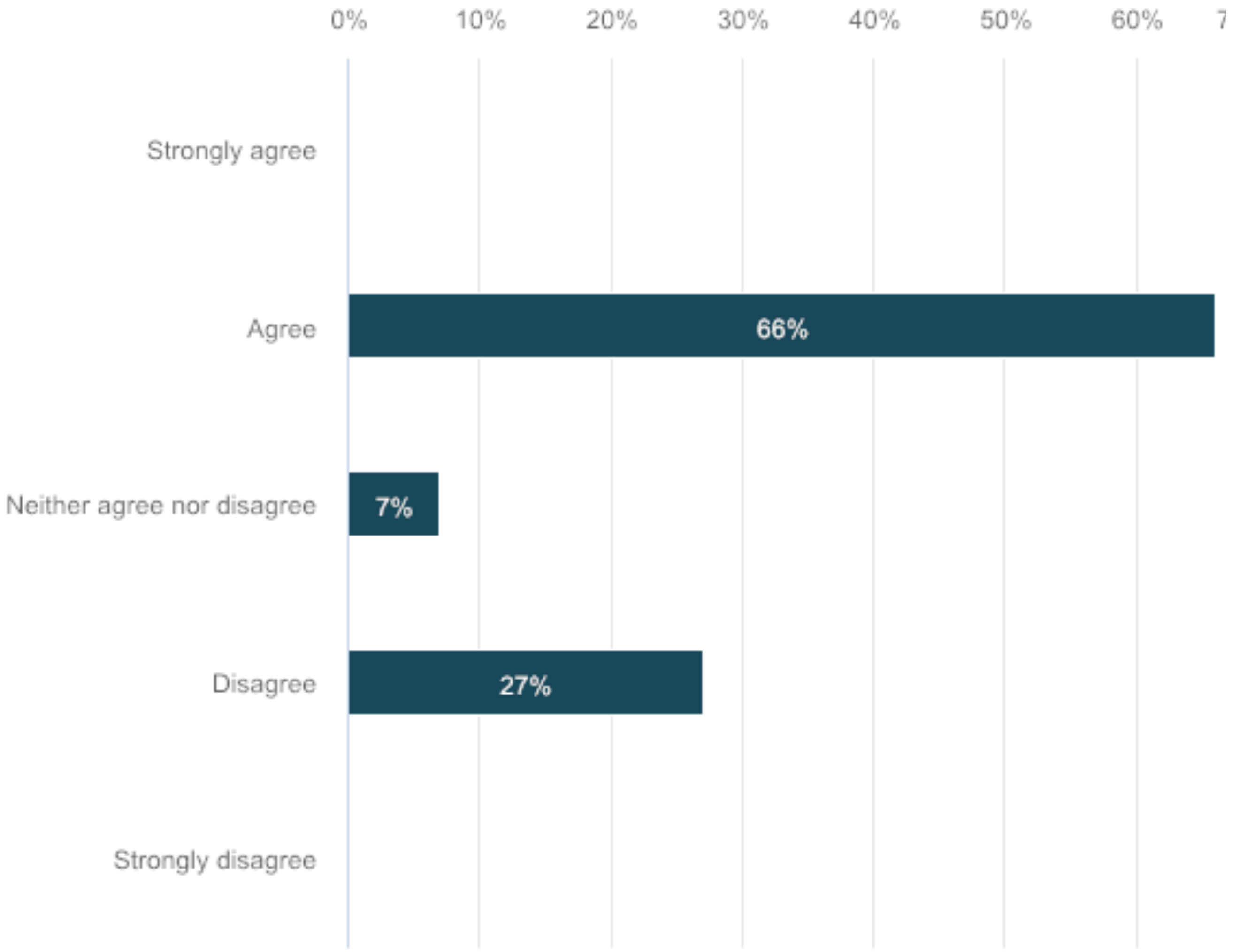

	n	Percent
Strongly agree	0	0%
Agree	10	66.66%
Neither agree nor disagree	1	6.67%
Disagree	4	26.67%
Strongly disagree	0	0%

28. For re-irradiation with SABR, no minimum PFTs apply.

Number of respondents: 15

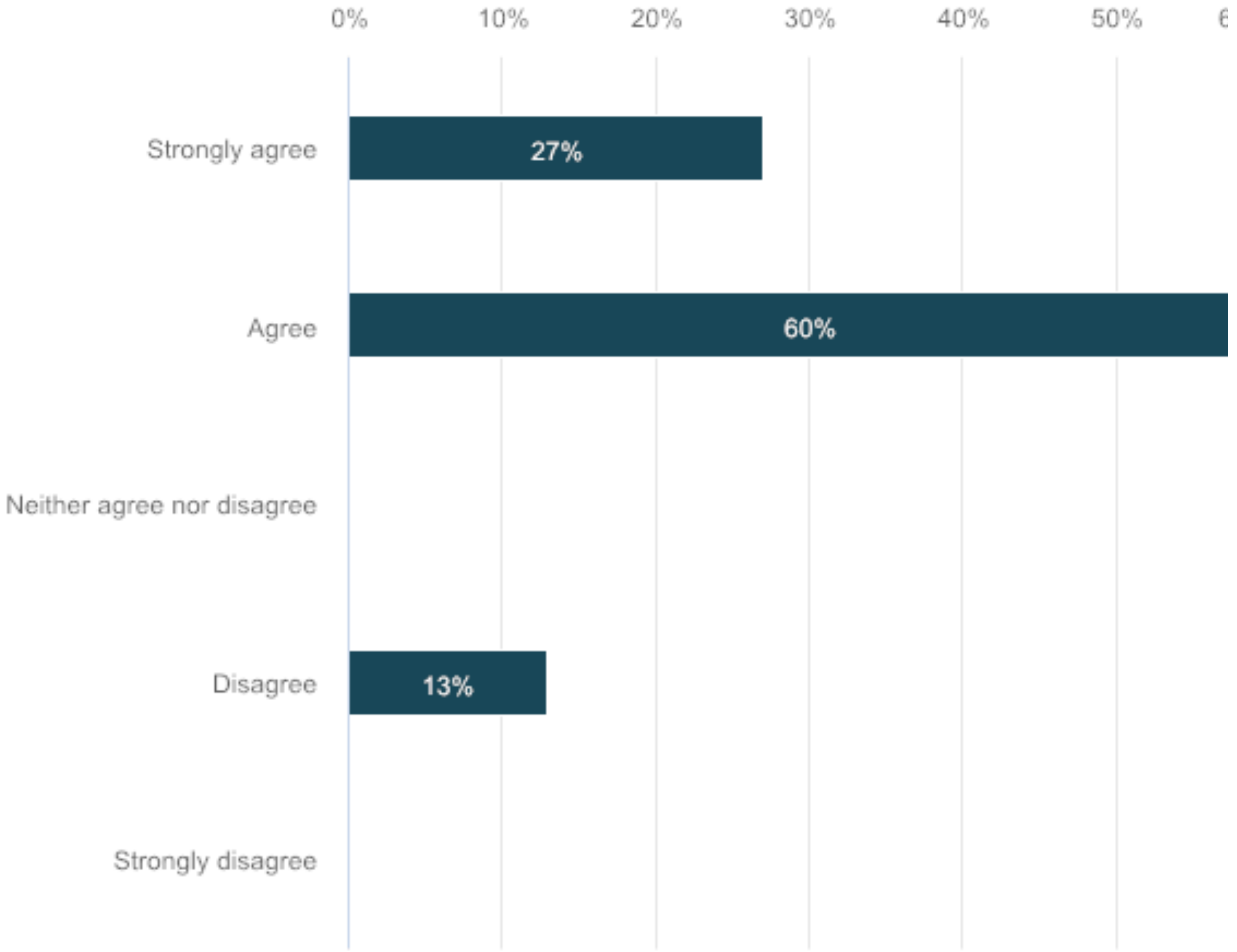

	n	Percent
Strongly agree	4	26.67%
Agree	9	60%
Neither agree nor disagree	0	0%
Disagree	2	13.33%
Strongly disagree	0	0%

29. Please add any comments here.

Number of respondents: 8

Responses
SABR can hurt people with very-poor lung function. Therefore, if FEV1 or DLCO <30%, it might not be wise. Especially if re-irradiating.
We use SABR in patinets with very poor lung function. Decsions need to be made on individual basis. Beware ILD though
none
We show that reducing FEV1 not prognostic in XRT treated NSCLC<br><br>For re-irradiation, I think change in PFTs between 1st XRT and now is more imp in my view
Always depend on size of new volume and location as may have awful lung function but treating lung that is not functioning and can still consider re-irradiation.
Bronchoscopy +/- endoscopy may be necessary to assess for endobronchial disease and risk/presence of fistula/perforation<br>MRI spine may be needed (e.g. for recurrent Pancoast tumors)
For questions 27, this will depend on the actual lung exposure of the reirradiation. Otherwise it is difficult to understand that there is not minimum PFT for SBRT, which has quite some lung expose depending on tumor size and location.<br><br>For both, I would suggest that a phrase such as "positive risk benefit ratio considering the current PF and exposure of the lung by reiirradiation"
Would normally set threshold values of 40% for DLCO and FEV1

30. An acceptable rate of grade 1-2 pneumonitis for radical thoracic re-irradiation is 50%

Number of respondents: 15

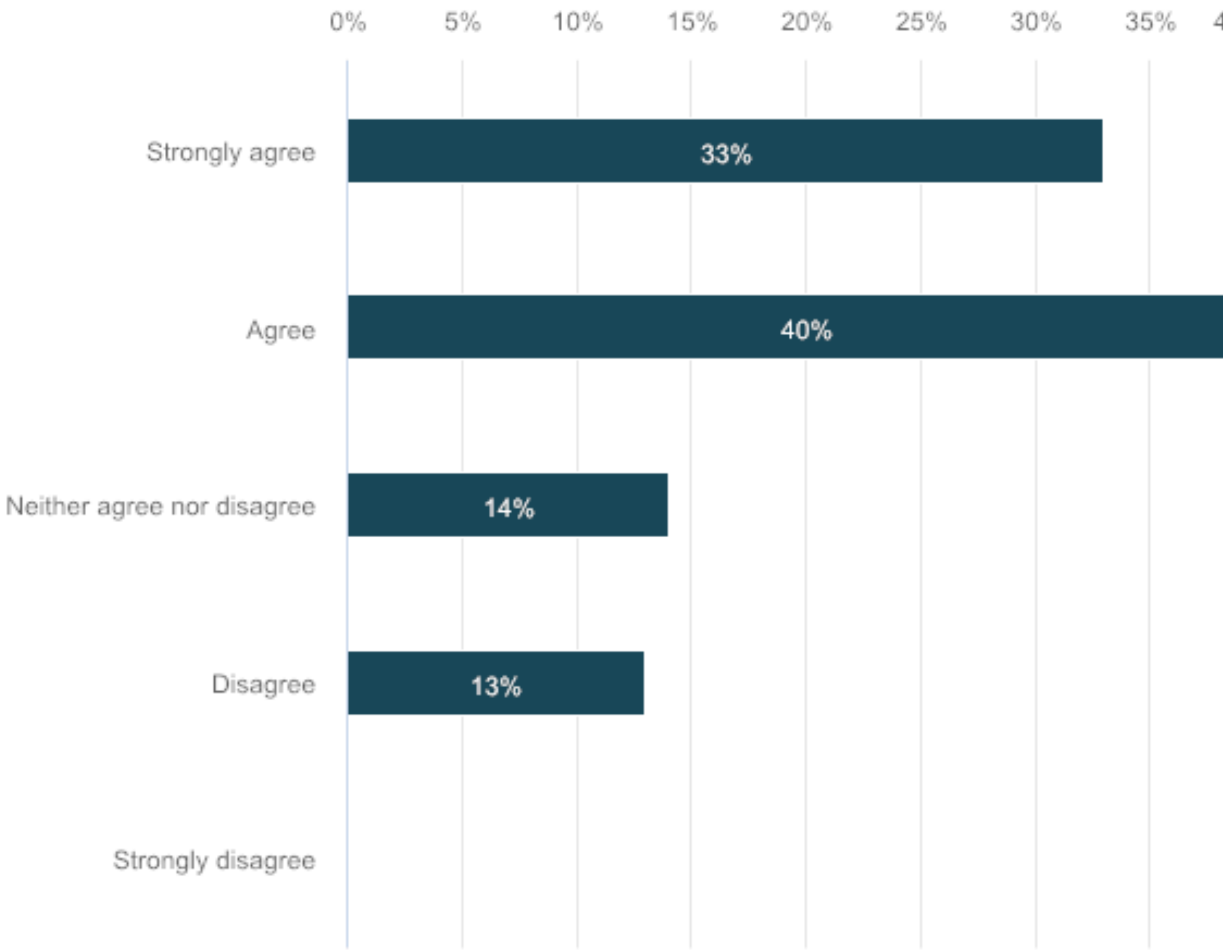

	n	Percent
Strongly agree	5	33.33%
Agree	6	40%
Neither agree nor disagree	2	13.34%
Disagree	2	13.33%
Strongly disagree	0	0%

31. An acceptable rate of grade 1-2 oesophagitis for radical thoracic re-irradiation is 70%
Number of respondents: 15

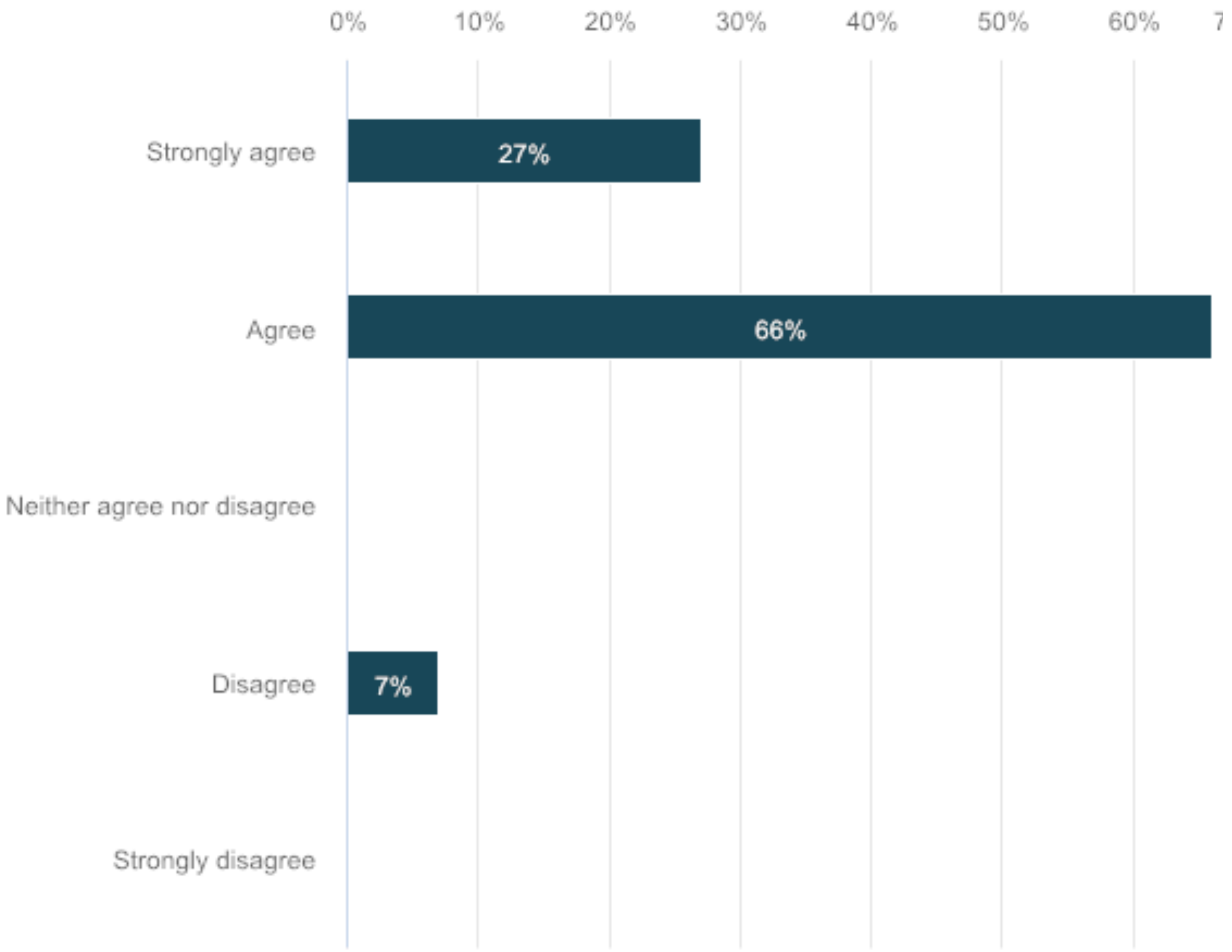

	n	Percent
Strongly agree	4	26.67%
Agree	10	66.66%
Neither agree nor disagree	0	0%
Disagree	1	6.67%
Strongly disagree	0	0%

32. An acceptable rate of grade 1-2 skin erythema for radical thoracic re-irradiation is 50%
Number of respondents: 15

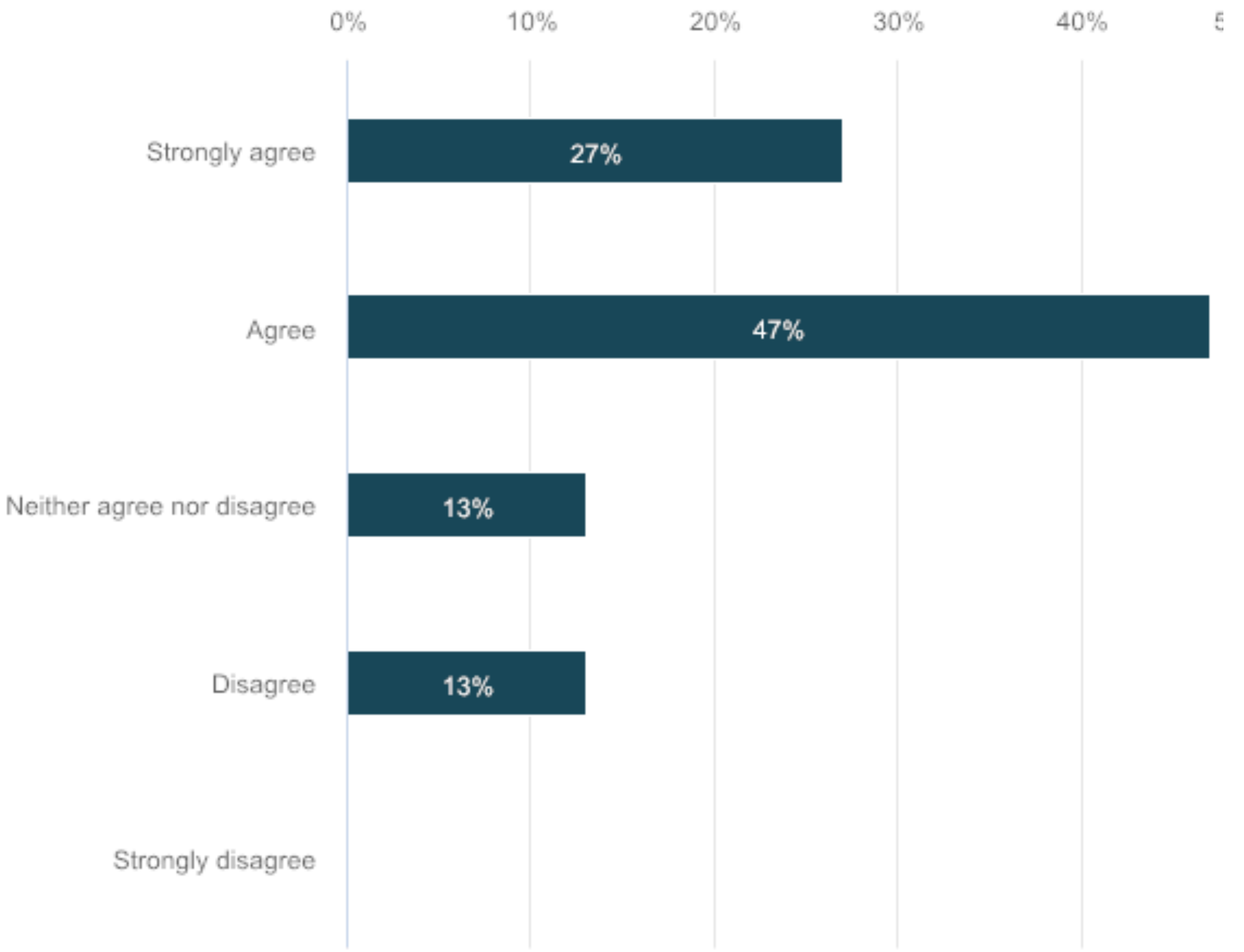

	n	Percent
Strongly agree	4	26.67%
Agree	7	46.67%
Neither agree nor disagree	2	13.33%
Disagree	2	13.33%
Strongly disagree	0	0%

33. An acceptable rate of grade 1-2 brachial plexopathy for radical thoracic re-irradiation is 10%
Number of respondents: 15

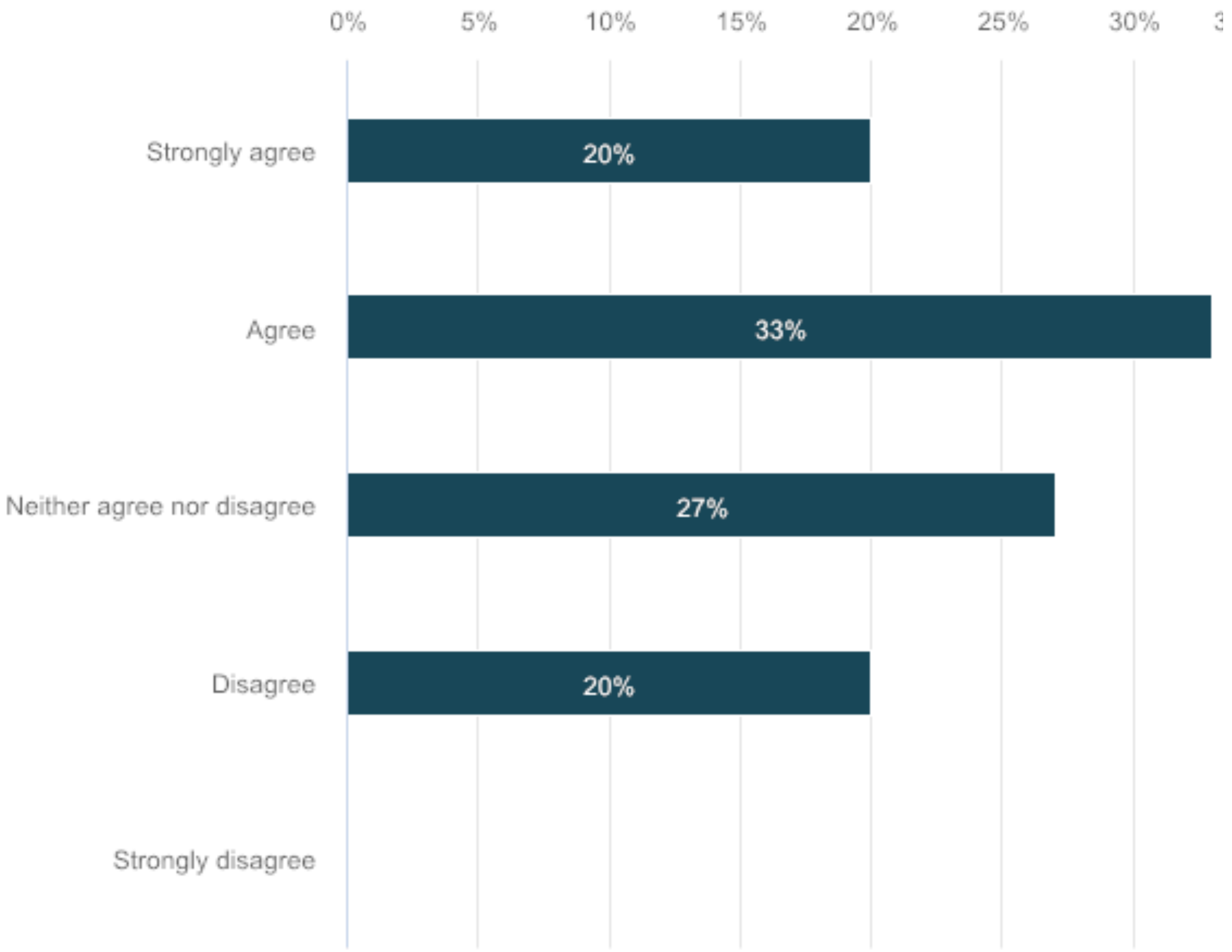

	n	Percent
Strongly agree	3	20%
Agree	5	33.33%
Neither agree nor disagree	4	26.67%
Disagree	3	20%
Strongly disagree	0	0%

34. An acceptable rate of grade 1-2 pericarditis for radical thoracic re-irradiation is 10%
Number of respondents: 15

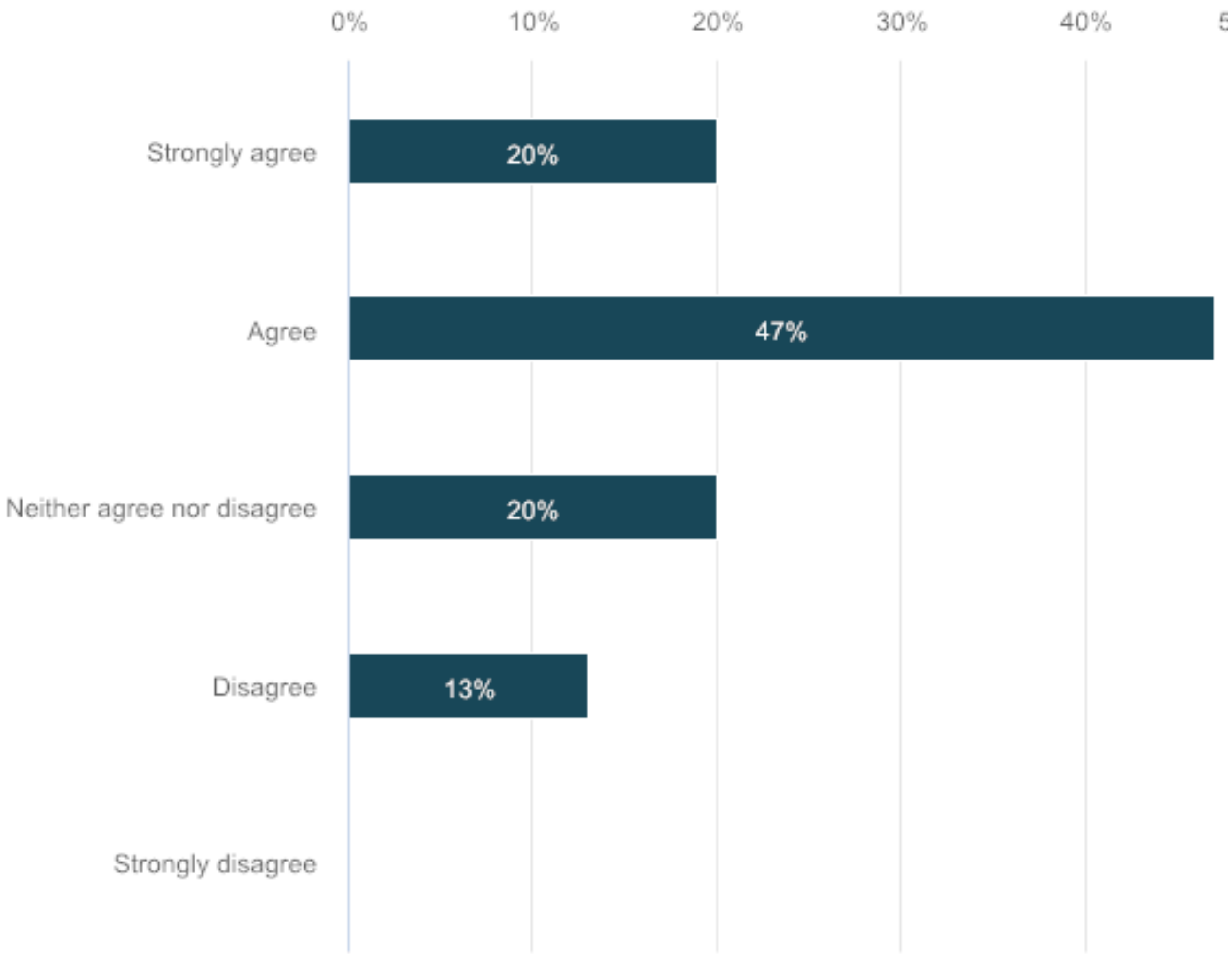

	n	Percent
Strongly agree	3	20%
Agree	7	46.67%
Neither agree nor disagree	3	20%
Disagree	2	13.33%
Strongly disagree	0	0%

35. Please add any comments here.

Number of respondents: 7

Responses
I would accept a high incidence of G 1-2 pericardial effusions. They are very common with re-irradiation and are manageable.
the opinion of the patient is crucial here
I think you should say the percentage "or less". For example, if someone disagrees with 50%, you don't know if it's too high or too low. For #31 and #32, I would accept 100%.
I would be happy to accept higher risks for G1-2 pneumonitis and skin erythema
In general, risks of high-grade toxicity will drive our decision making in this scenario and so in general, would not exclude patients from re-RT based on above parameters/risk of low-grade toxicity
Grade 2 brachial plexopathy rate should be 0% unless there is a risk of neoplastic injury to the plexus
Question 30, in general:<br>I am not in favor of grouping grade 1 and grade 2 toxicity. E.g. regarding grade I pneumoniitis, we all would allow a 100% risk of ASYMPTOMATIC changes in CT follow-up images<br><br>-> Restrict to symptomatic toxicity

36. An acceptable rate of grade 3-4 pneumonitis for radical thoracic re-irradiation is 10%.
Number of respondents: 15

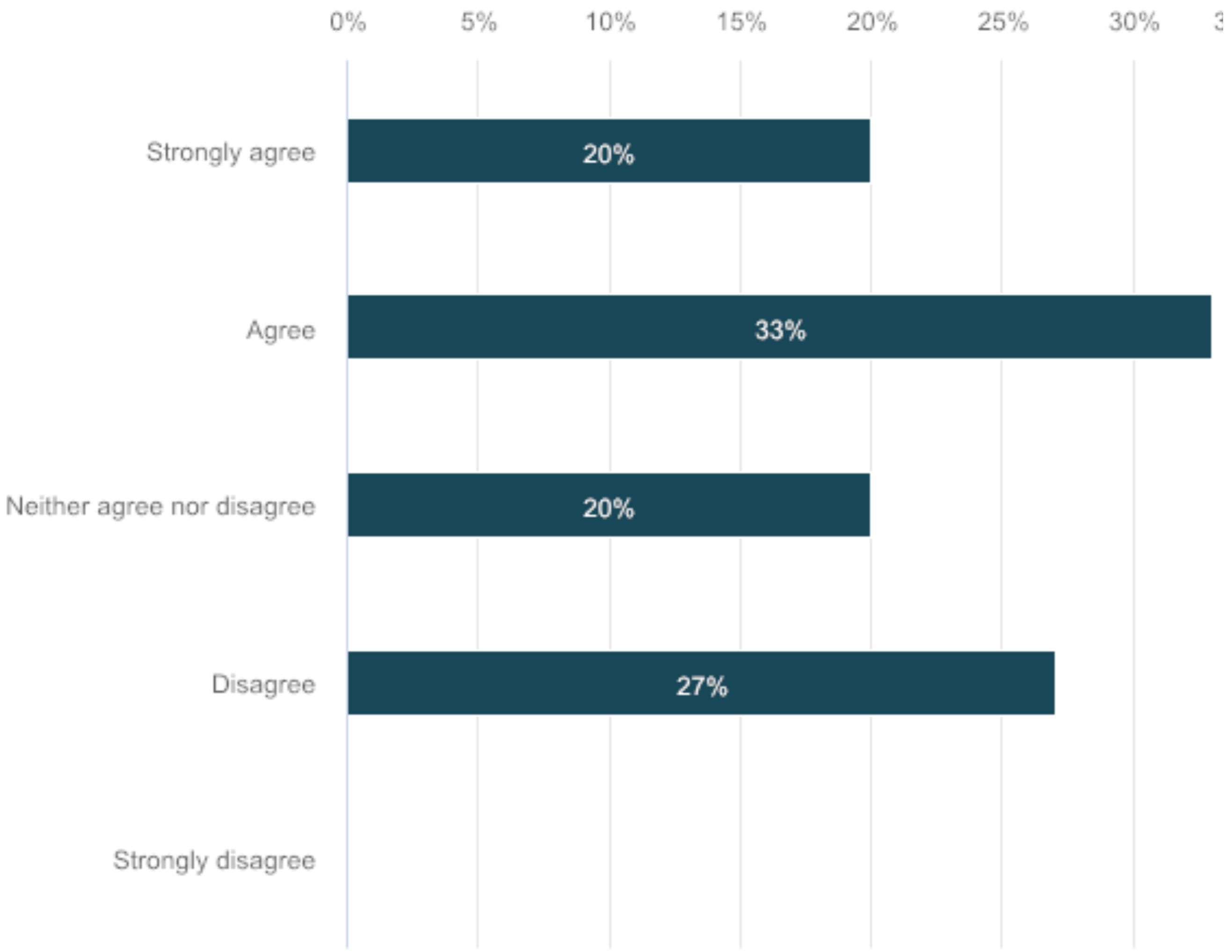

	n	Percent
Strongly agree	3	20%
Agree	5	33.33%
Neither agree nor disagree	3	20%
Disagree	4	26.67%
Strongly disagree	0	0%

37. An acceptable rate of grade 3-4 oesophagitis for radical thoracic re-irradiation is 10%

Number of respondents: 15

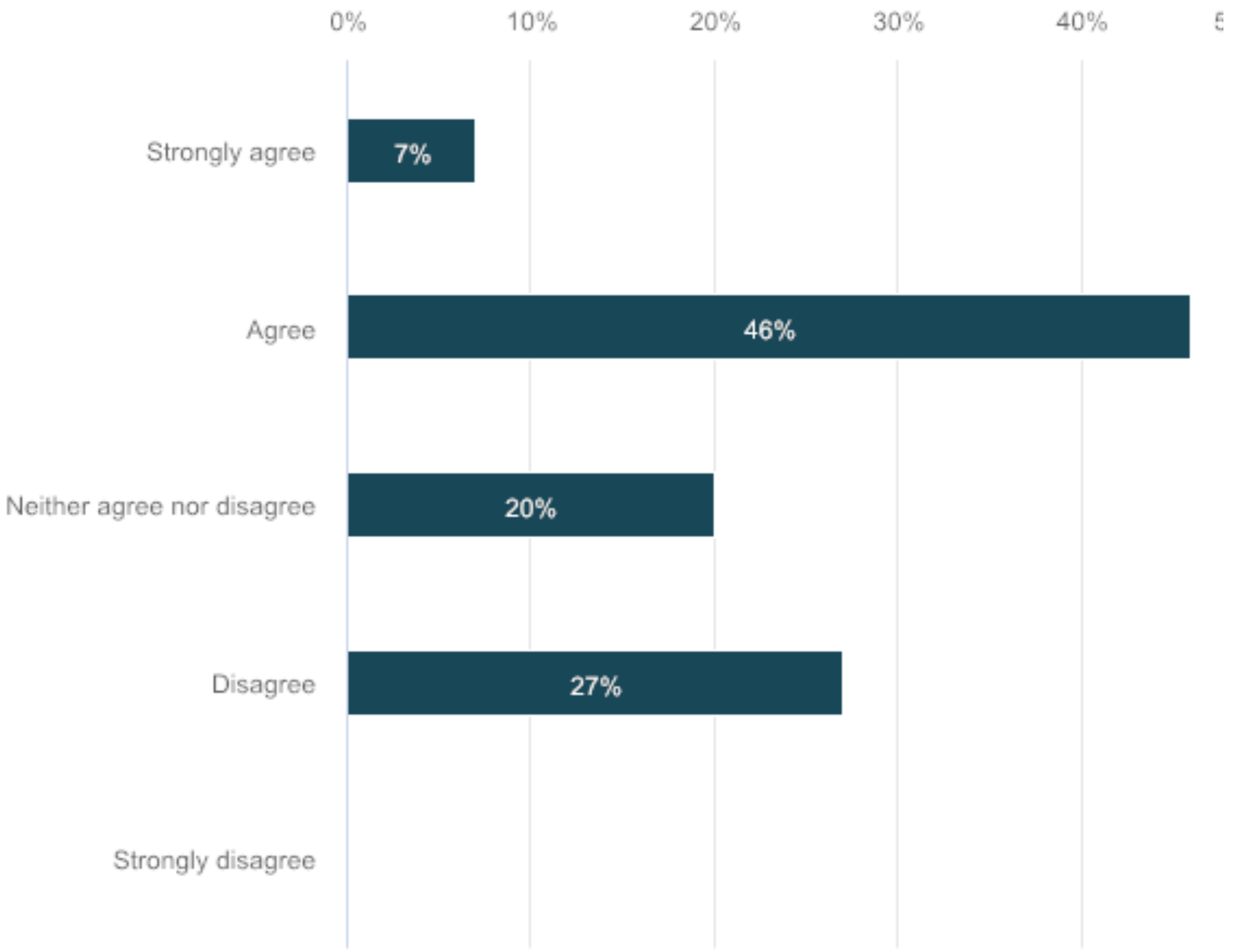

	n	Percent
Strongly agree	1	6.67%
Agree	7	46.66%
Neither agree nor disagree	3	20%
Disagree	4	26.67%
Strongly disagree	0	0%

38. An acceptable rate of grade 3-4 skin toxicity for radical thoracic re-irradiation is 5%.
Number of respondents: 15

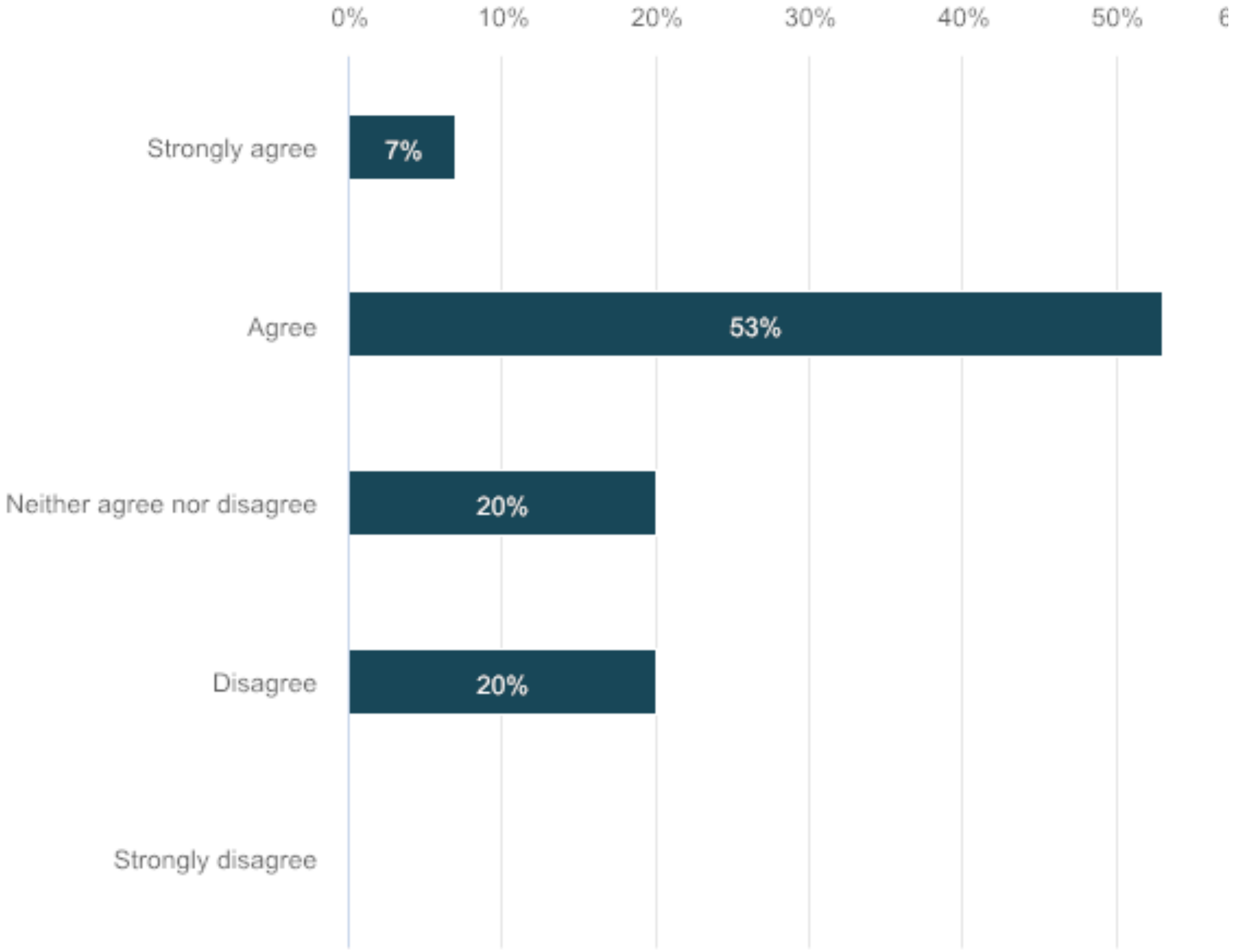

	n	Percent
Strongly agree	1	6.67%
Agree	8	53.33%
Neither agree nor disagree	3	20%
Disagree	3	20%
Strongly disagree	0	0%

39. An acceptable rate of grade 3-4 brachial plexopathy for radical thoracic re-irradiation is 5%.
Number of respondents: 15

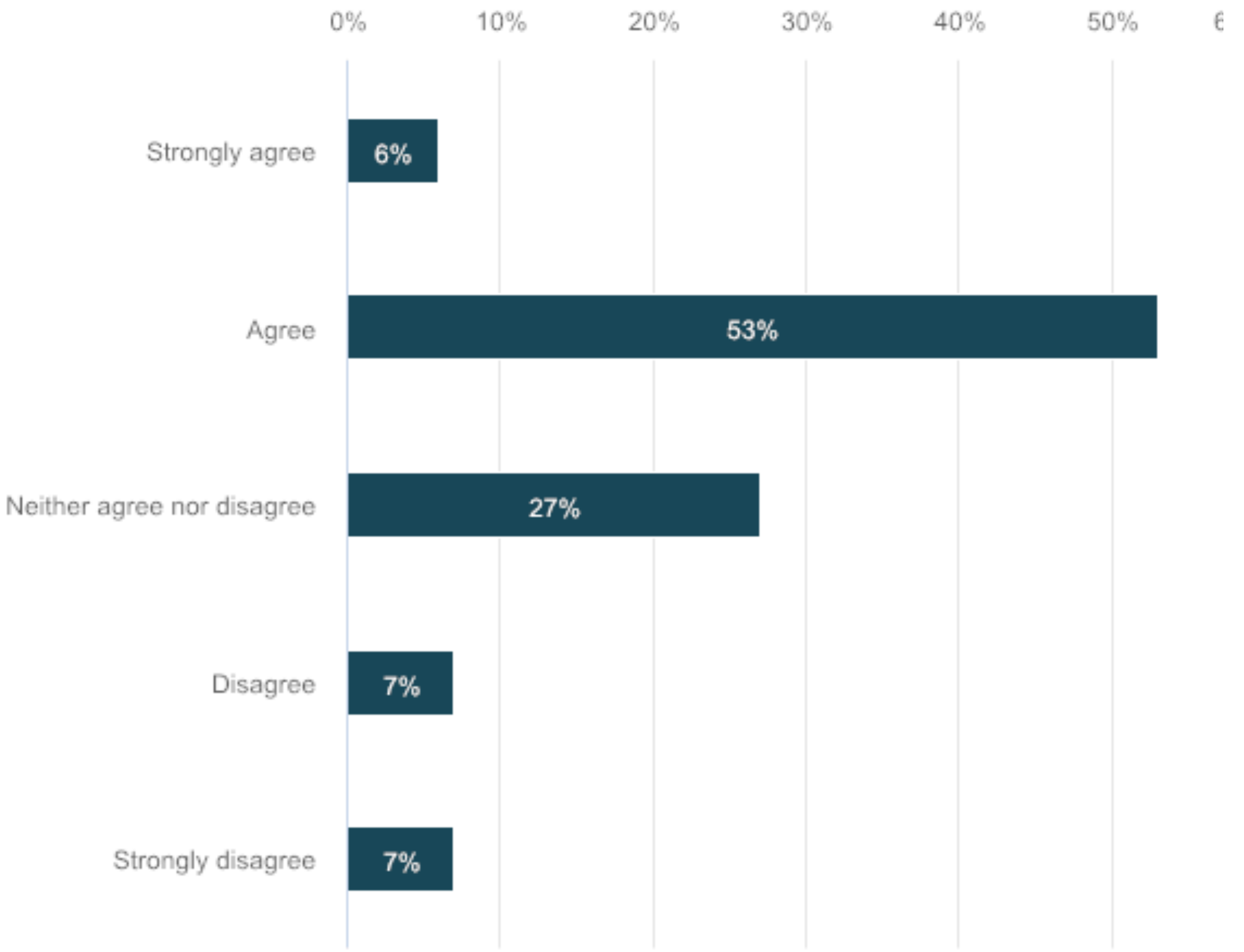

	n	Percent
Strongly agree	1	6.67%
Agree	8	53.33%
Neither agree nor disagree	4	26.66%
Disagree	1	6.67%
Strongly disagree	1	6.67%

40. An acceptable rate of grade 3-4 pericarditis for radical thoracic re-irradiation is 10%
Number of respondents: 15

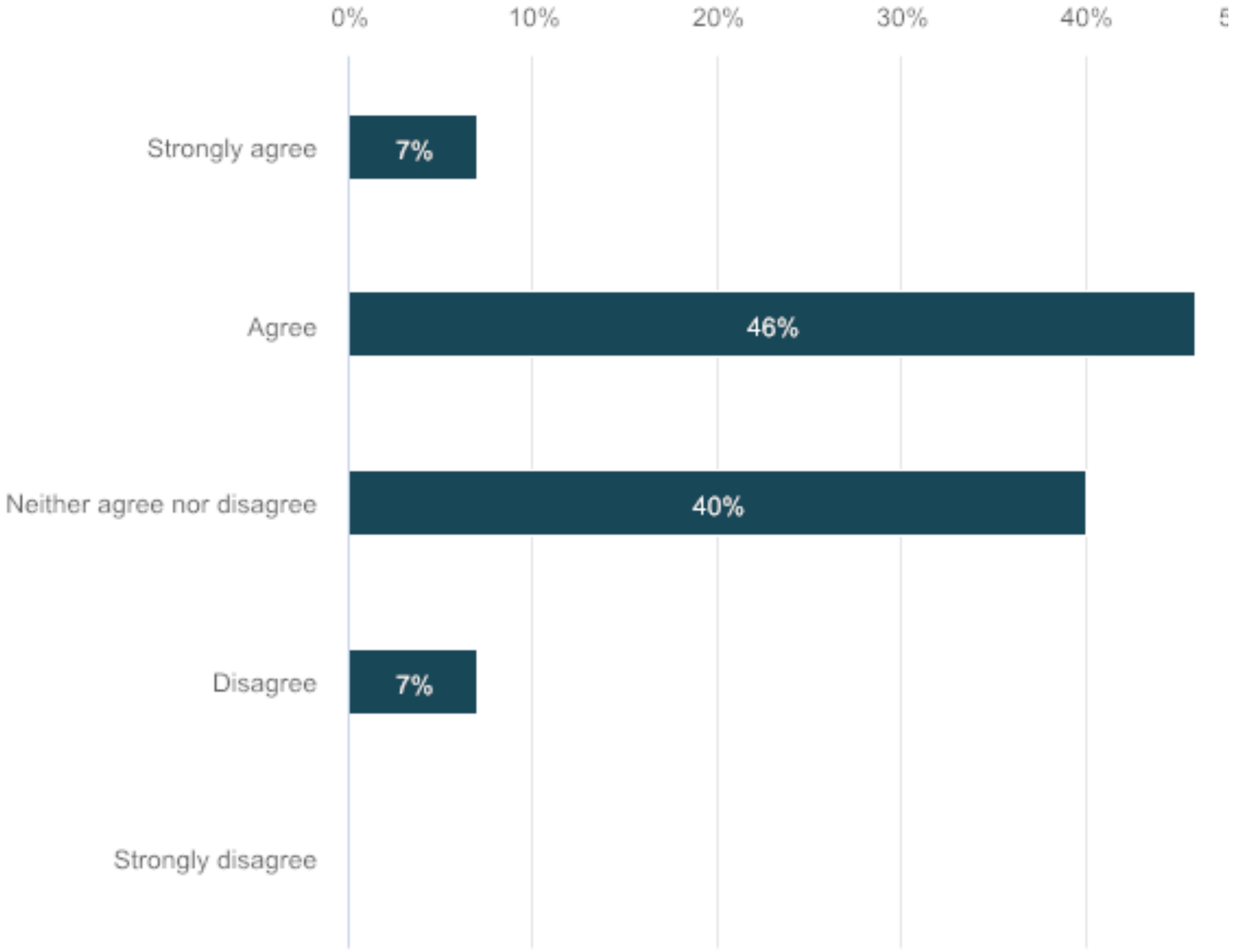

	n	Percent
Strongly agree	1	6.67%
Agree	7	46.66%
Neither agree nor disagree	6	40%
Disagree	1	6.67%
Strongly disagree	0	0%

41. An acceptable rate of grade 3-4 bronchial fibrosis for radical thoracic re-irradiation is 5%

Number of respondents: 15

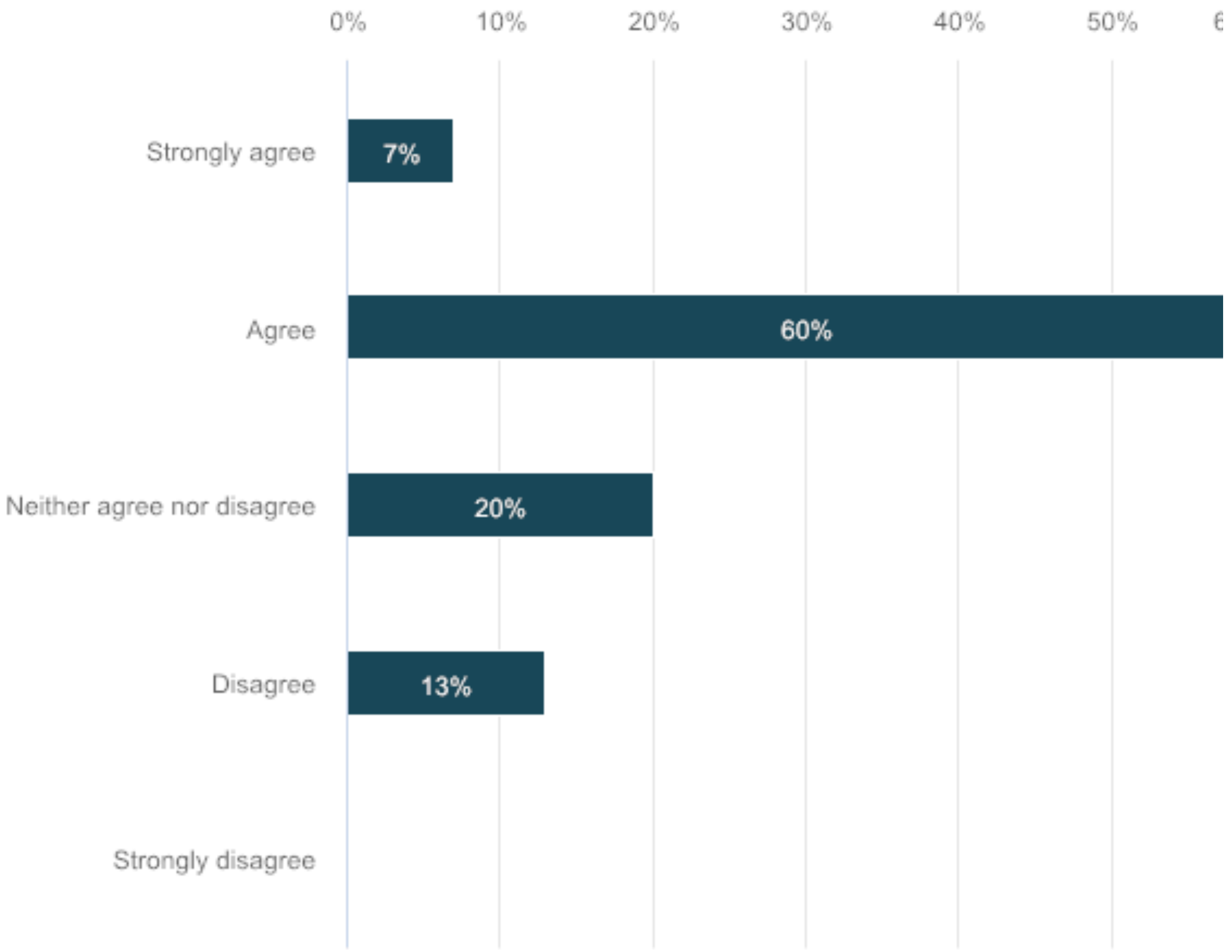

	n	Percent
Strongly agree	1	6.67%
Agree	9	60%
Neither agree nor disagree	3	20%
Disagree	2	13.33%
Strongly disagree	0	0%

42. An acceptable rate of grade 3-4 haemoptysis for radical thoracic re-irradiation is 5%
Number of respondents: 15

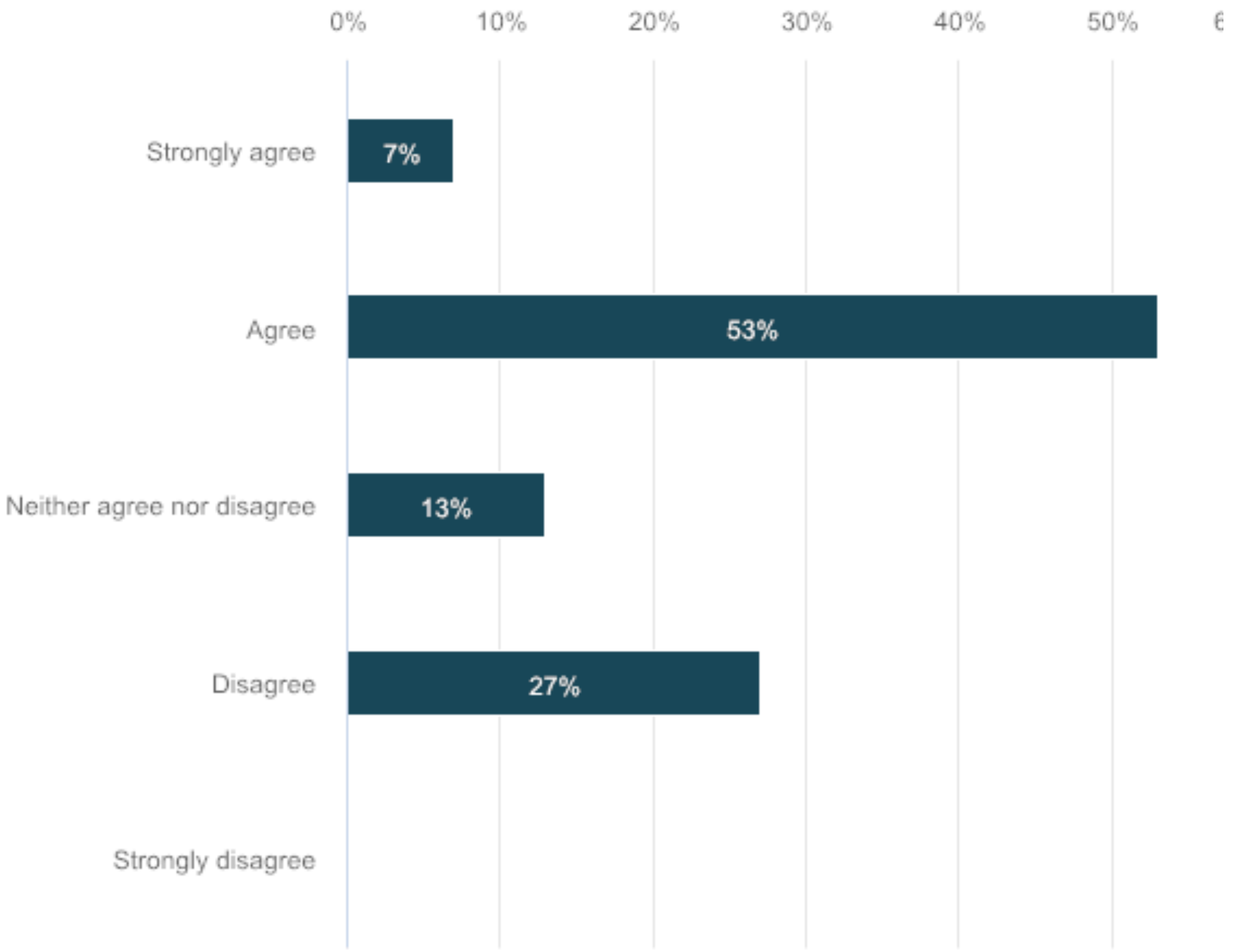

	n	Percent
Strongly agree	1	6.67%
Agree	8	53.33%
Neither agree nor disagree	2	13.33%
Disagree	4	26.67%
Strongly disagree	0	0%

43. An acceptable rate of grade 3-4 spinal cord myelitis for radical thoracic re-irradiation is 5%

Number of respondents: 15

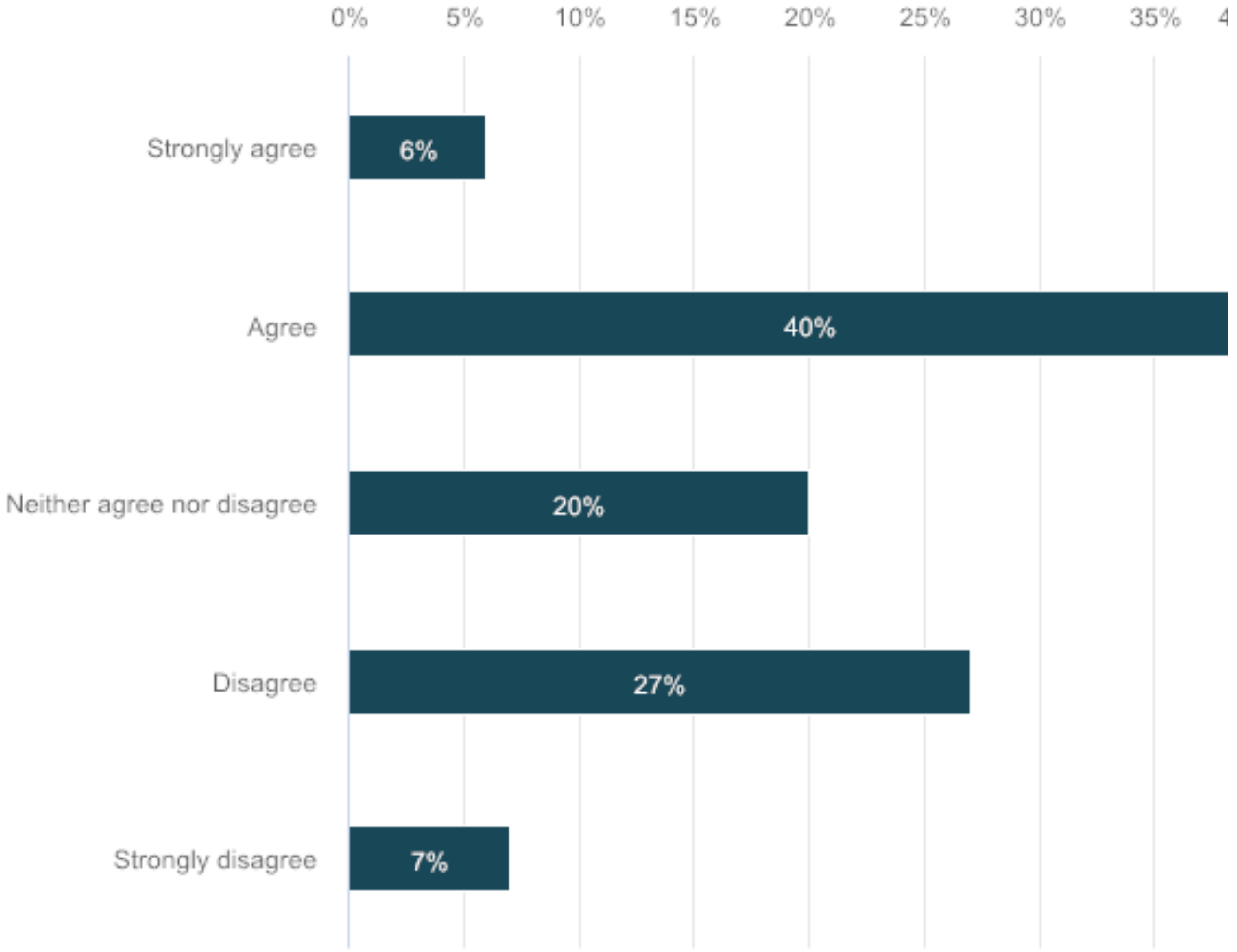

	n	Percent
Strongly agree	1	6.67%
Agree	6	40%
Neither agree nor disagree	3	20%
Disagree	4	26.66%
Strongly disagree	1	6.67%

44. In general, the expected grade 5 toxicity rate from radical thoracic re-irradiation is less than 5%.
Number of respondents: 15

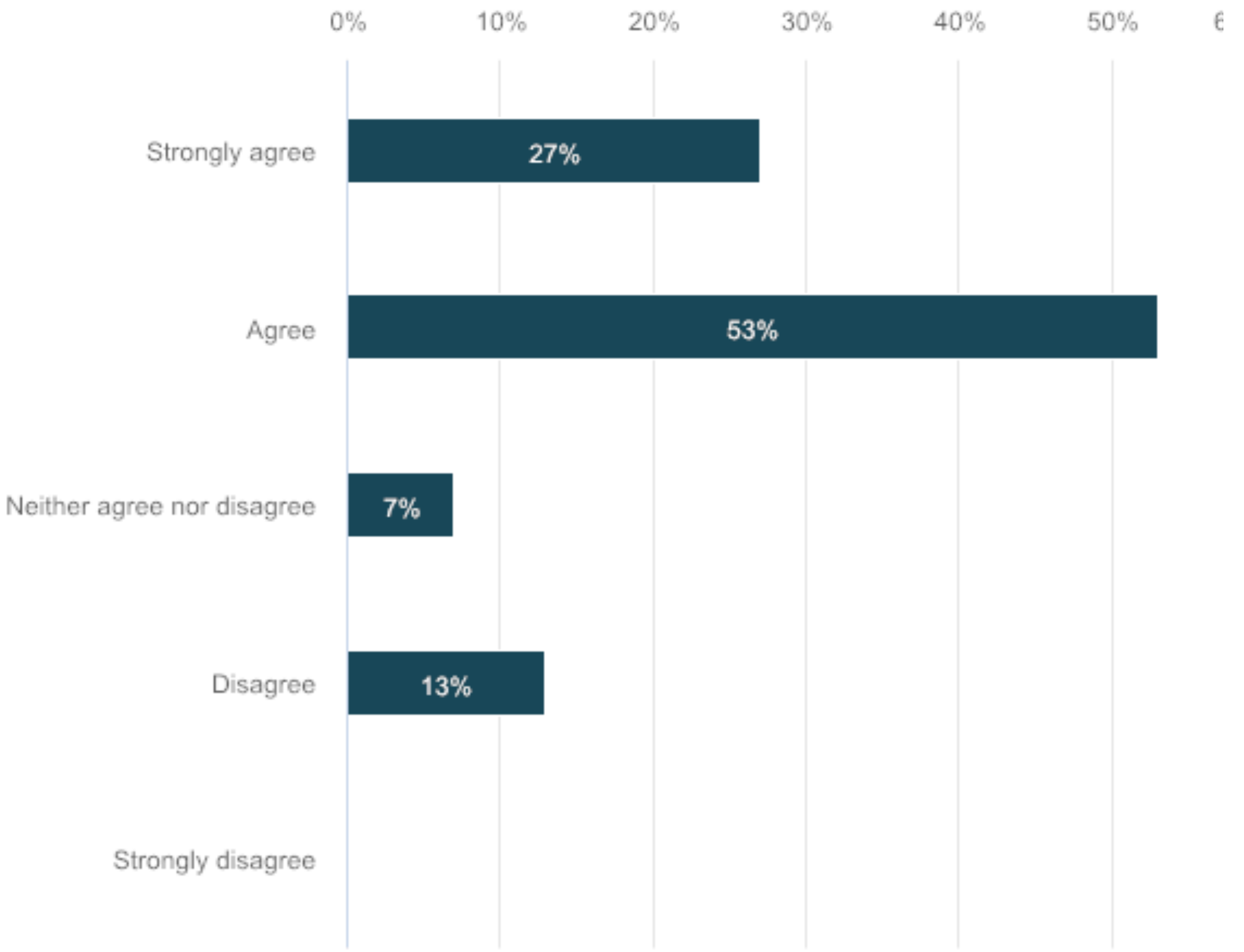

	n	Percent
Strongly agree	4	26.67%
Agree	8	53.33%
Neither agree nor disagree	1	6.67%
Disagree	2	13.33%
Strongly disagree	0	0%

45. Please add any comments here.

Number of respondents: 7

Responses
I dislike the idea of clinicians scoring these questions about "acceptable rate". It would tremendously help this paper, and the field in general, if these questions were asked of lung cancer pts who previously underwent radiotherapy. It will naturally add more time, but would help this truly stand out from other Delphi studies. It would be great to see a title that emphasized "Delphi model developed by clinicians and patients for..."
1. I cannot answer these questions. Is it acute or late toxicity?<br><br>2. The opinion of the patient is crucial here
A 10% rate of pneumonitis is low. That is lower than the rate with standard chemoRT for stage III.
Proviso for all is clear and well documented informed consent with patient and peer review of plan before treatment
In general we would not exclude patients with a risk of G3-4 toxicity higher than listed above<br>In some cases, for example central re-RT, the risk of G5 toxicity may be higher
Some of the thresholds above are indeed close to or even below the risk of toxicity in radical RCT for stage III, in the primary situation !
36) G3-4 Pneumonitis should be ~5% or less. Doses should therefore be tailored to meet this threshold.<br><br>43) Cord myelitis should be <3% or kept to a minimum, given the severe nature of the effect and the fact that cord is not even threatened in the first place.

46. For radical re-irradiation, IV contrast enhanced 4D-CT is the recommended simulation technique.
Number of respondents: 15

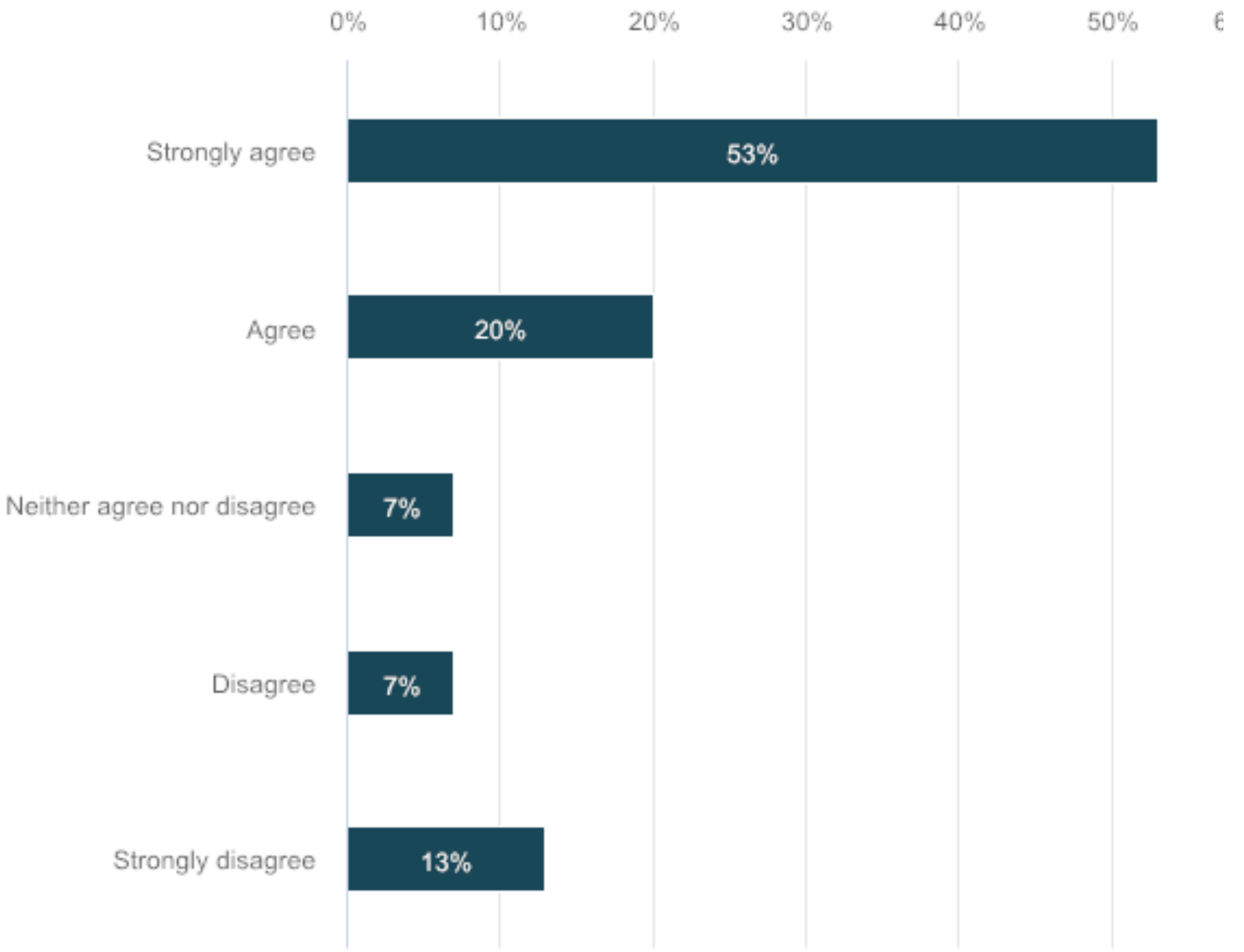

	n	Percent
Strongly agree	8	53.33%
Agree	3	20%
Neither agree nor disagree	1	6.67%
Disagree	1	6.67%
Strongly disagree	2	13.33%

47. When combining initial and re-irradiation plans, either rigid or deformable dose registration are acceptable methods (although there are considerable uncertainties in either process and further investigation is warranted).

Number of respondents: 15

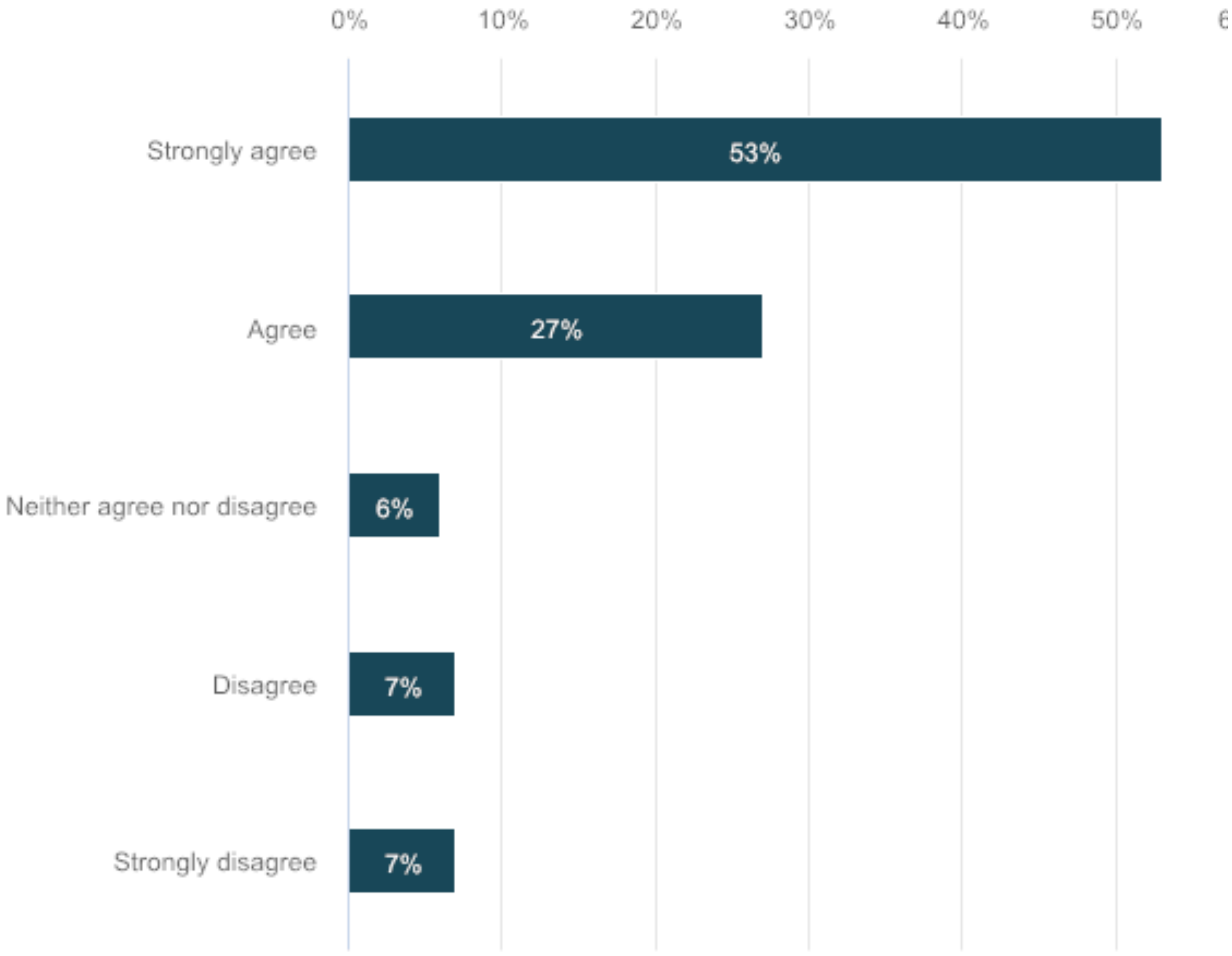

	n	Percent
Strongly agree	8	53.33%
Agree	4	26.66%
Neither agree nor disagree	1	6.67%
Disagree	1	6.67%
Strongly disagree	1	6.67%

48. 18-FDG-PET-CT is recommended to aid tumour volume delineation.
Number of respondents: 15

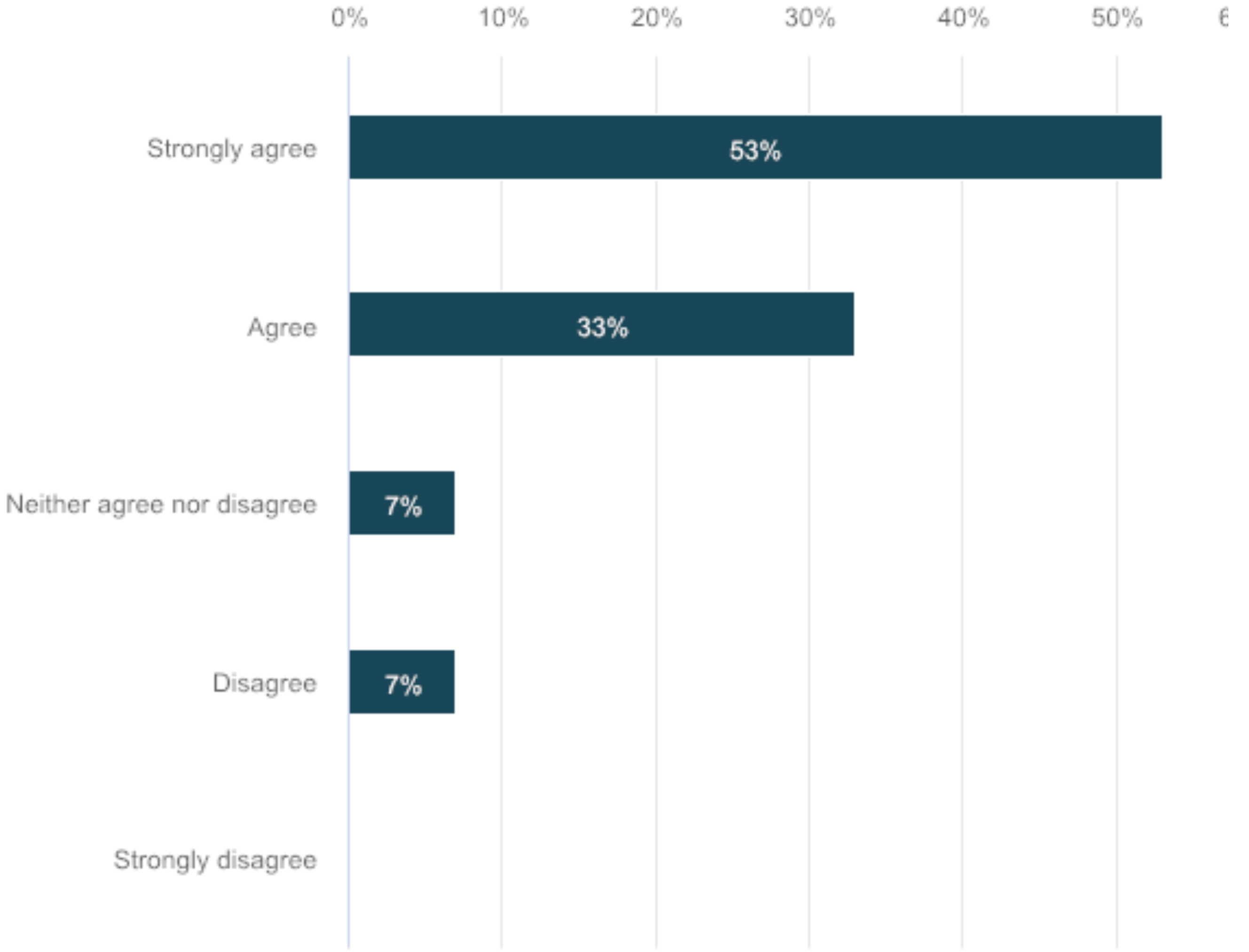

	n	Percent
Strongly agree	8	53.33%
Agree	5	33.33%
Neither agree nor disagree	1	6.67%
Disagree	1	6.67%
Strongly disagree	0	0%

49. When contouring for conventionally fractionated radical re-irradiation, an acceptable minimum expansion from iGTV to CTV is 5mm (with normal structures, excepting lung, edited out of the CTV).

Number of respondents: 15

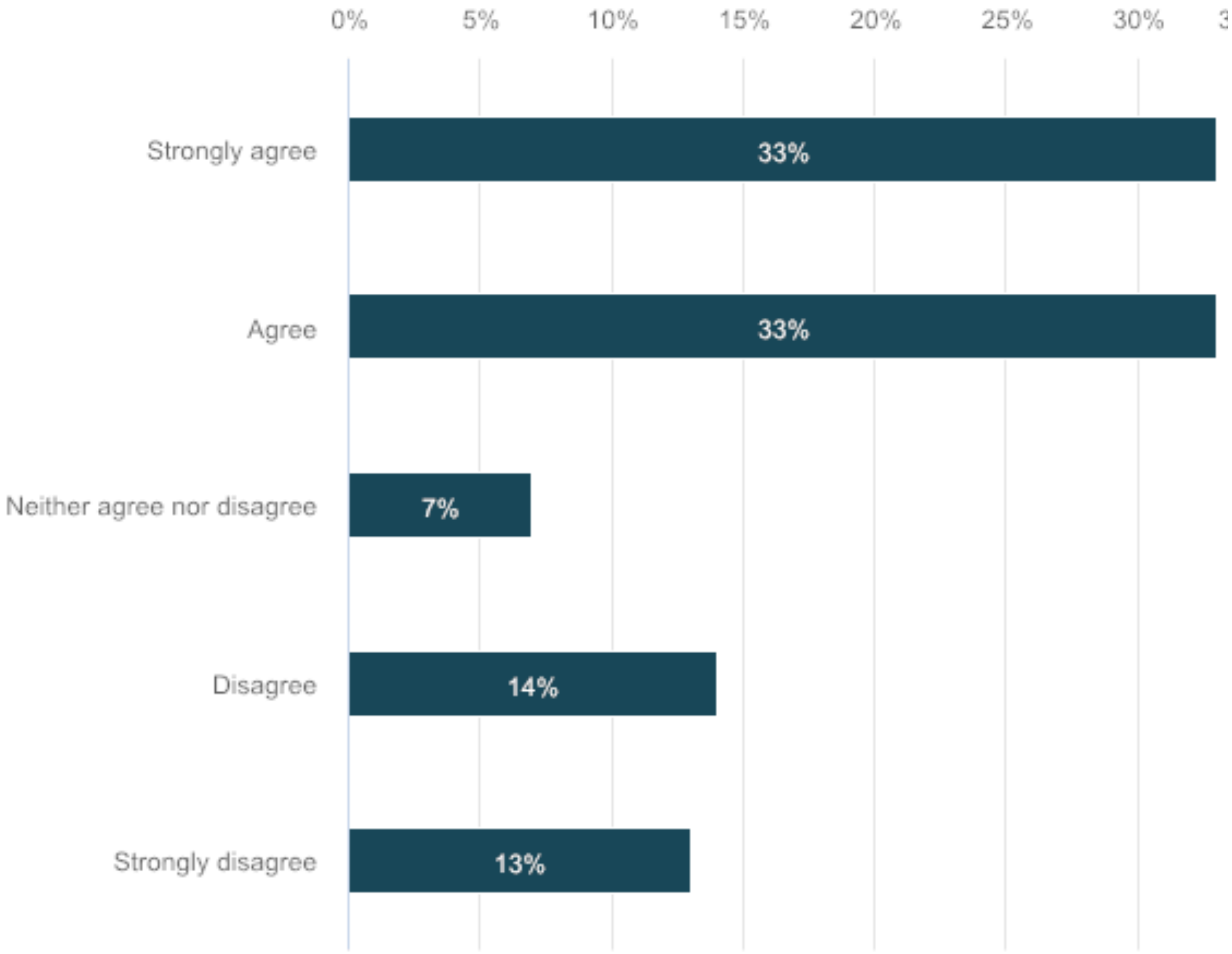

	n	Percent
Strongly agree	5	33.33%
Agree	5	33.33%
Neither agree nor disagree	1	6.67%
Disagree	2	13.34%
Strongly disagree	2	13.33%

50. When contouring for conventionally frationationed radical re-irradiation, an acceptable minimum expansion from CTV to PTV is 5mm (or follow institutional guidelines where available).
Number of respondents: 15

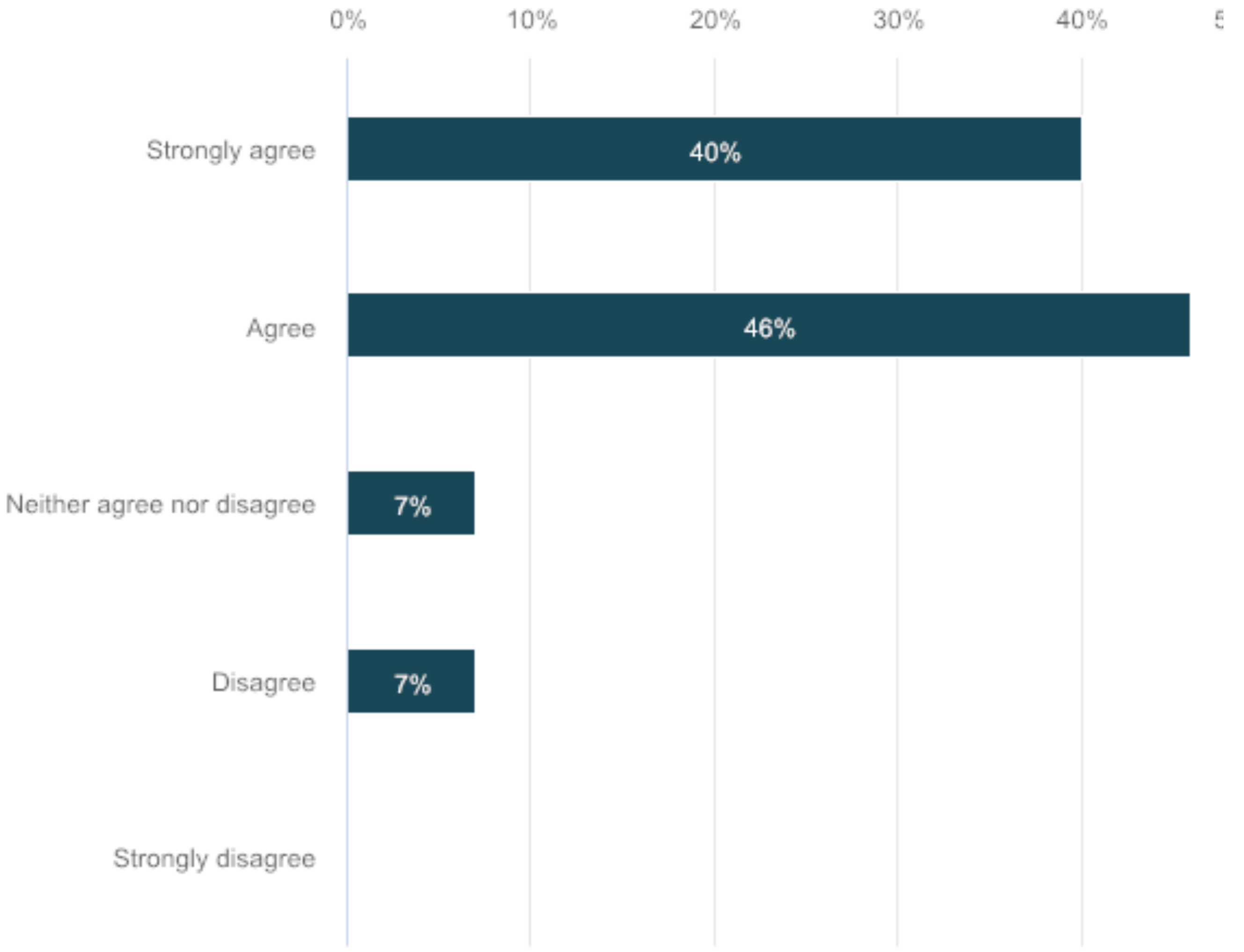

	n	Percent
Strongly agree	6	40%
Agree	7	46.66%
Neither agree nor disagree	1	6.67%
Disagree	1	6.67%
Strongly disagree	0	0%

51. PTV coverage can be compromised to achieve acceptable OAR doses.
Number of respondents: 15

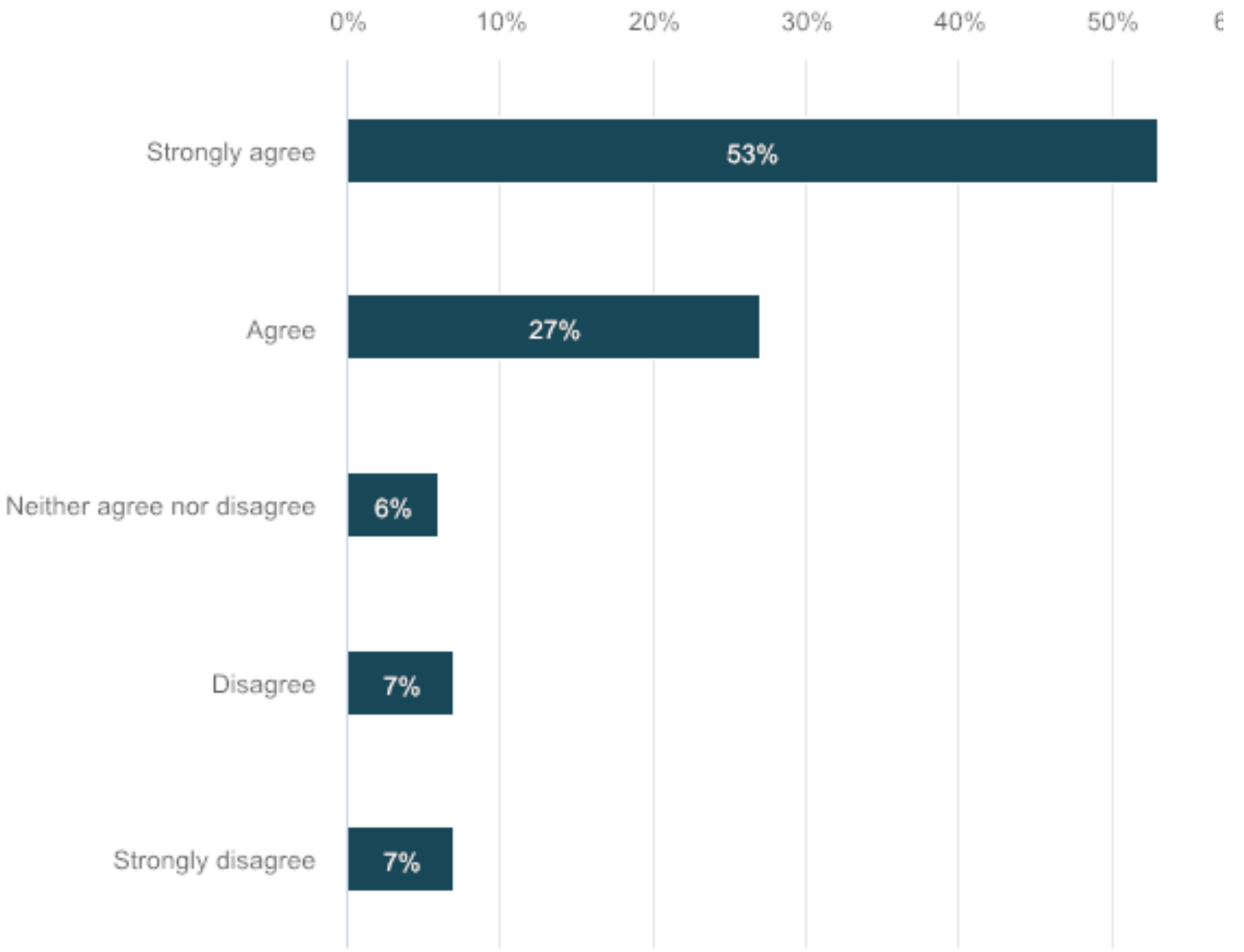

	n	Percent
Strongly agree	8	53.33%
Agree	4	26.66%
Neither agree nor disagree	1	6.67%
Disagree	1	6.67%
Strongly disagree	1	6.67%

52. Please add any comments here.

Number of respondents: 11

Responses
IV contrast cannot be given at the same time as 4DCT. However, an IV contrasted CT should be obtained and fused to the 4DCT.<br><br><br><br>The PTV should encompass the tumor in it's entirety. It should not be compromised, unless a CRITICAL organ at risk is closeby (PBT, esophagus, trachea, spinal cord, plexus)
I would accept compromises in re-irradiation. It's a tricky area and evidence is lacking. I favour a pragmatic approach with a considered and careful approach to patient consent.
depends on the % of underdosage
#46 - IV contrast is not helpful for peripheral tutors at all. It is useful for central tumors<br><br>#48 - please see IAEA guidelines on use of PET for contouring (Gerry Hanna is an author). There are substantial limitations. I would say it is optional.<br><br>#49. The ITV is not expanded to form the CTV. It is the other way around. Did you mean iGTV to ITV? Many do not use CTV margins with re-irradiation. Normal structures are usually edited out if they are barriers to spread. Lung is a "normal structure"
Note the nomenclature here is incorrect - it should be expansion of iGTV to ITV = 5mm, by definition, ITV concept already contains a margin for microscopic disease (CTV)
I think better to say recomended CTV is 0.5, but can be reduced. I would edit/ omit CTV, but not PTV margin.<br><br>Coverage to GTV, CTV and PTV can be compromised for OAR
46: Non-contrast 4DCT is our standard, with IV if needed<br>47: Deformable dose accumulation alone (too) error prone, especially when e.g. post-RT fibrosis from initial treatment, therefore suggest always also do rigid assessment<br>49: 0mm as minimum ITV-CTV expansion since in some situations margin reduction to try and limit risks of high-grade toxicity is indicated
ITV is created from CTV, not the other way around. We use iGTV, which is a composite of the volume encompassing physiologic motion of the GTV, then add 5 mm to create ITV
Question 46: 4D-CT with iv contrast is not possible for many CT scanner -> sequential 4D-CT and 3D with iv contrast<br><br>Question 47: I would not recommend deformable registration as a standard procedure in clinical routine -> QA concerns
48) Particularly if previous RT has resulted in a lot of background interstitial changes. However, PET fusion has its own challenges which need further

investigation.
Note for 49. I think you mean Gtv to. CTV. ITV and CTV are similar just that ITV is CTV w motion

53. VMAT (or other forms of IMRT) is the preferred radiotherapy technique for radical re-irradiation.
Number of respondents: 15

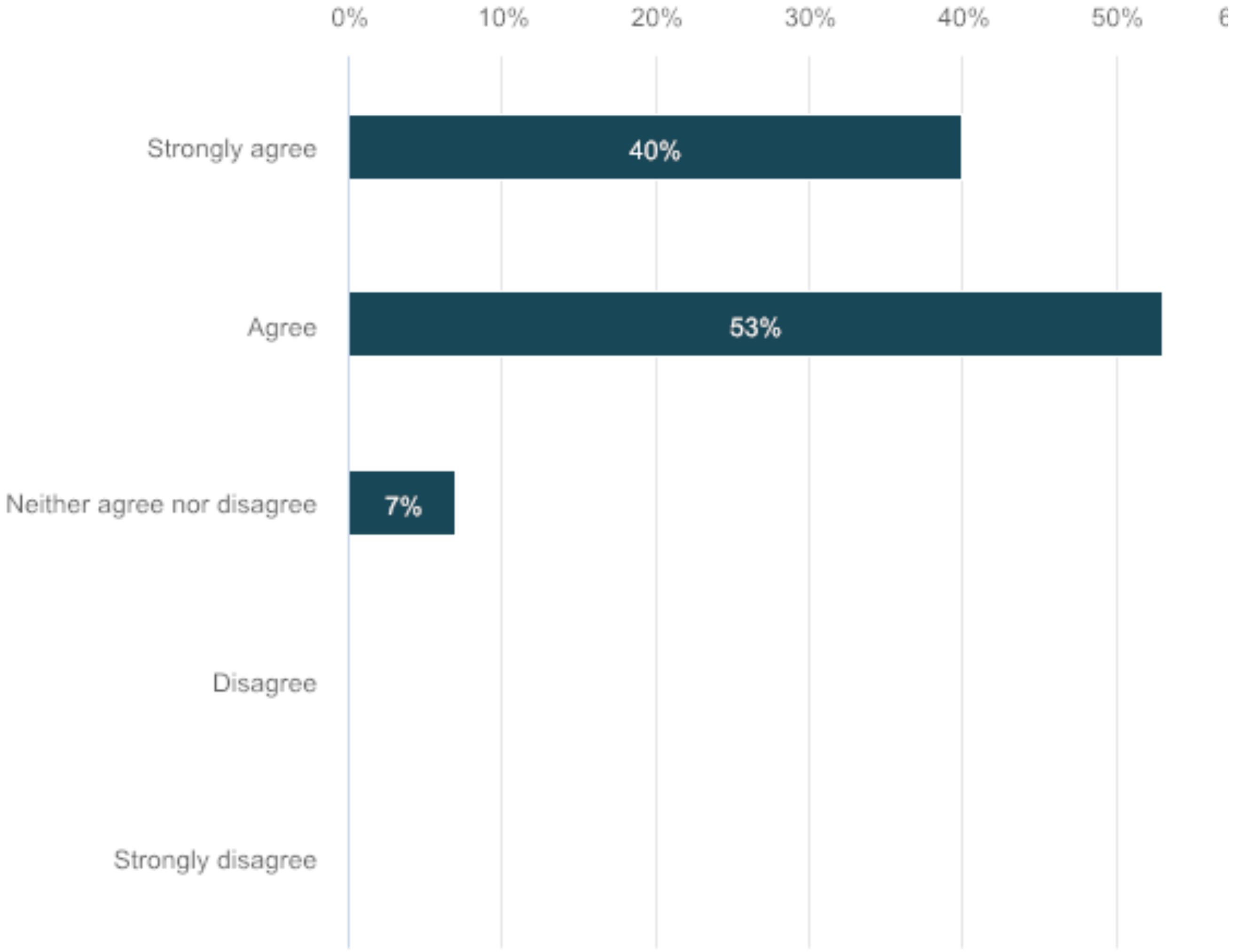

	n	Percent
Strongly agree	6	40%
Agree	8	53.33%
Neither agree nor disagree	1	6.67%
Disagree	0	0%
Strongly disagree	0	0%

54. SABR is the preferred re-irradiation technique where the tumour is not ultra-central, the tumour volume is small and there is minimal overlap with OARs.
Number of respondents: 15

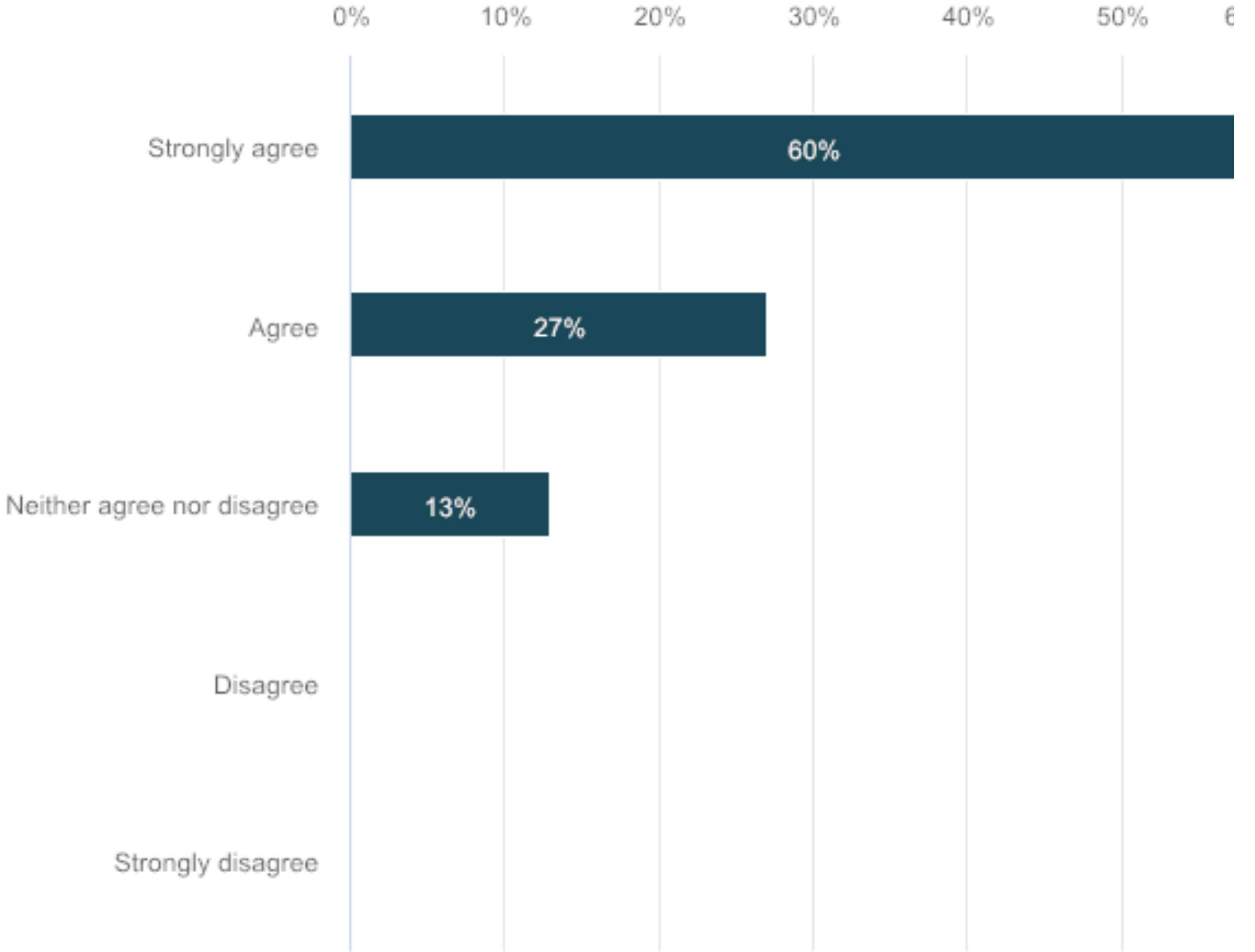

	n	Percent
Strongly agree	9	60%
Agree	4	26.67%
Neither agree nor disagree	2	13.33%
Disagree	0	0%
Strongly disagree	0	0%

55. Protons may have a role for re-irradiation but at present should not be used routinely and requires further evaluation in the context of a clinical trial.
Number of respondents: 15

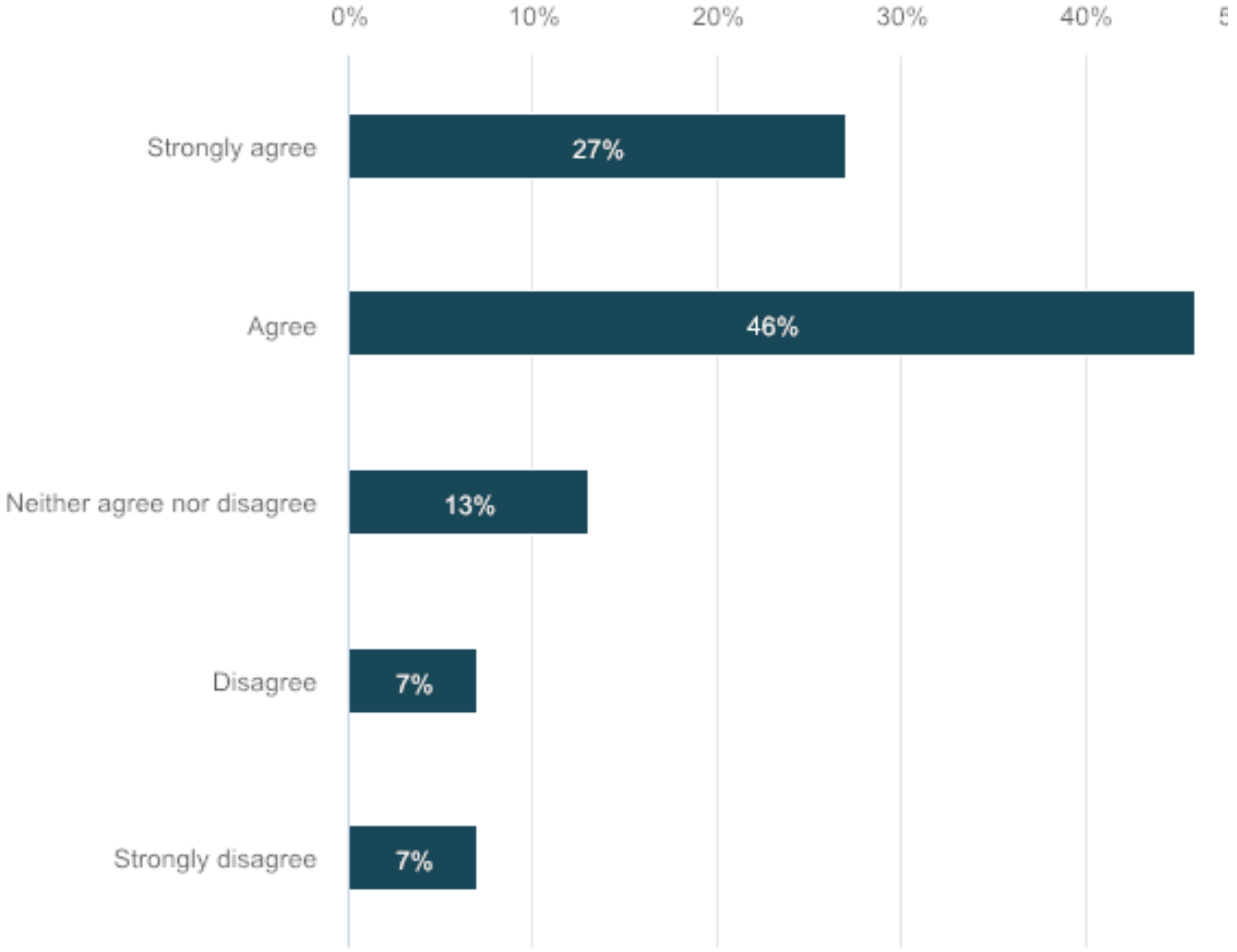

	n	Percent
Strongly agree	4	26.67%
Agree	7	46.66%
Neither agree nor disagree	2	13.33%
Disagree	1	6.67%
Strongly disagree	1	6.67%

56. Acceptable doses for conventionally fractionated radical thoracic re-irradiation are 60Gy in 30 fractions or 55 Gray in 20 fractions once daily for non-small cell lung cancer.

Number of respondents: 15

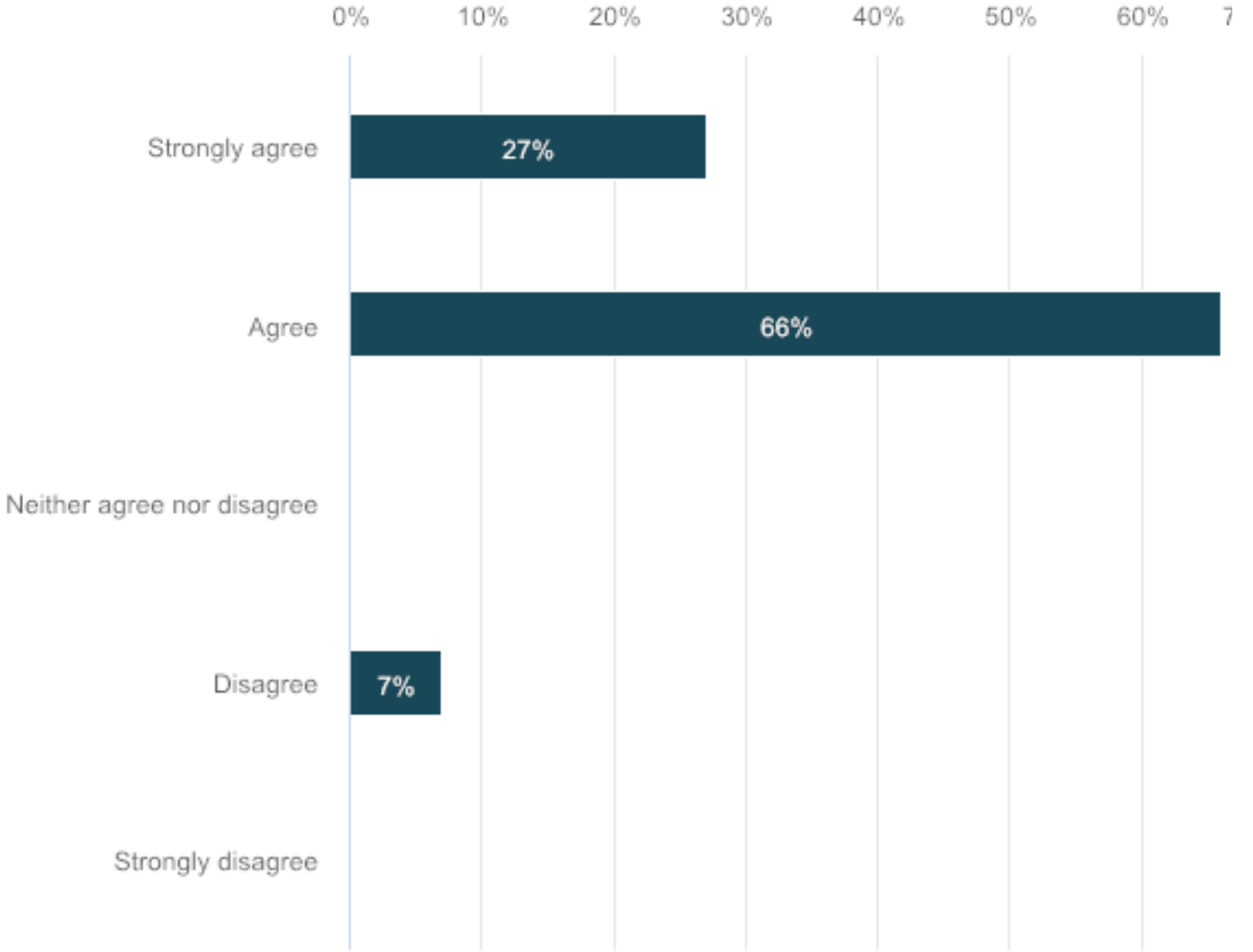

	n	Percent
Strongly agree	4	26.67%
Agree	10	66.66%
Neither agree nor disagree	0	0%
Disagree	1	6.67%
Strongly disagree	0	0%

57. An acceptable dose for conventionally fractionated radical thoracic re-irradiation is 45Gy in 30 fractions (twice daily) for small cell lung cancer.

Number of respondents: 15

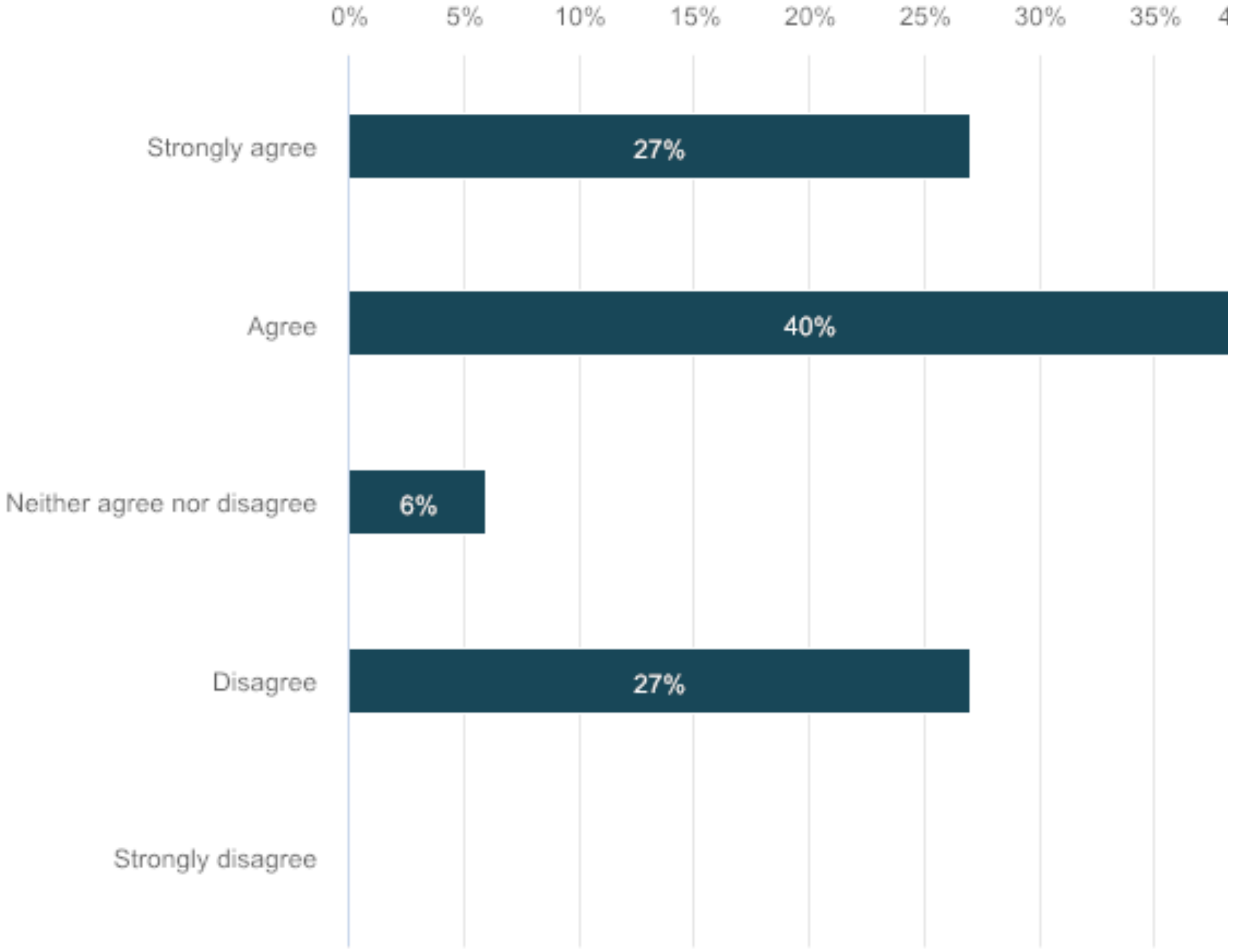

	n	Percent
Strongly agree	4	26.66%
Agree	6	40%
Neither agree nor disagree	1	6.67%
Disagree	4	26.67%
Strongly disagree	0	0%

58. Any dose and fractionation that delivers a BED >100 Gy to the tumour in 4 or more fractions is acceptable for radical re-irradiation using SABR.

Number of respondents: 15

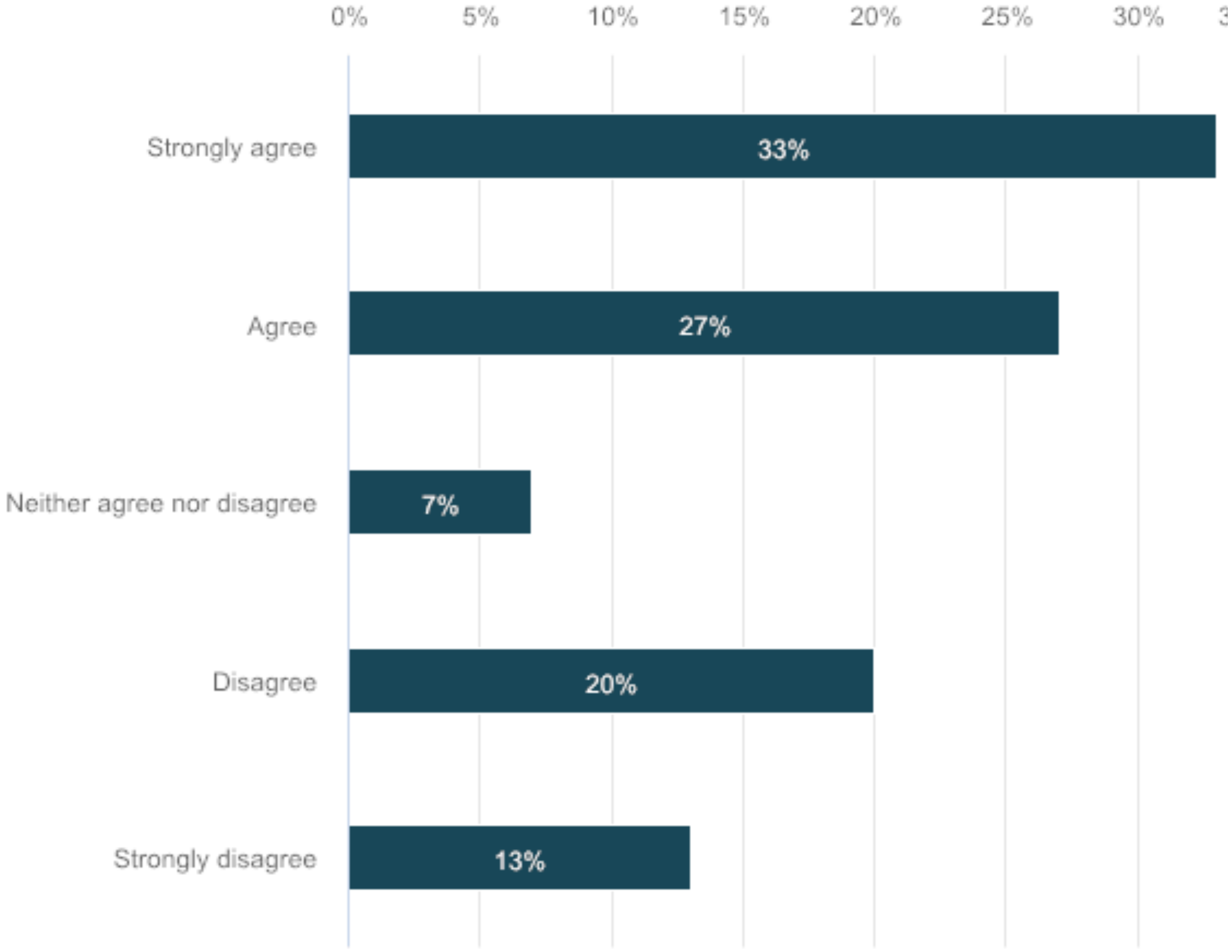

	n	Percent
Strongly agree	5	33.33%
Agree	4	26.67%
Neither agree nor disagree	1	6.67%
Disagree	3	20%
Strongly disagree	2	13.33%

59. Daily cone beam CT is recommended for treatment verification for SABR re-irradiation.
Number of respondents: 15

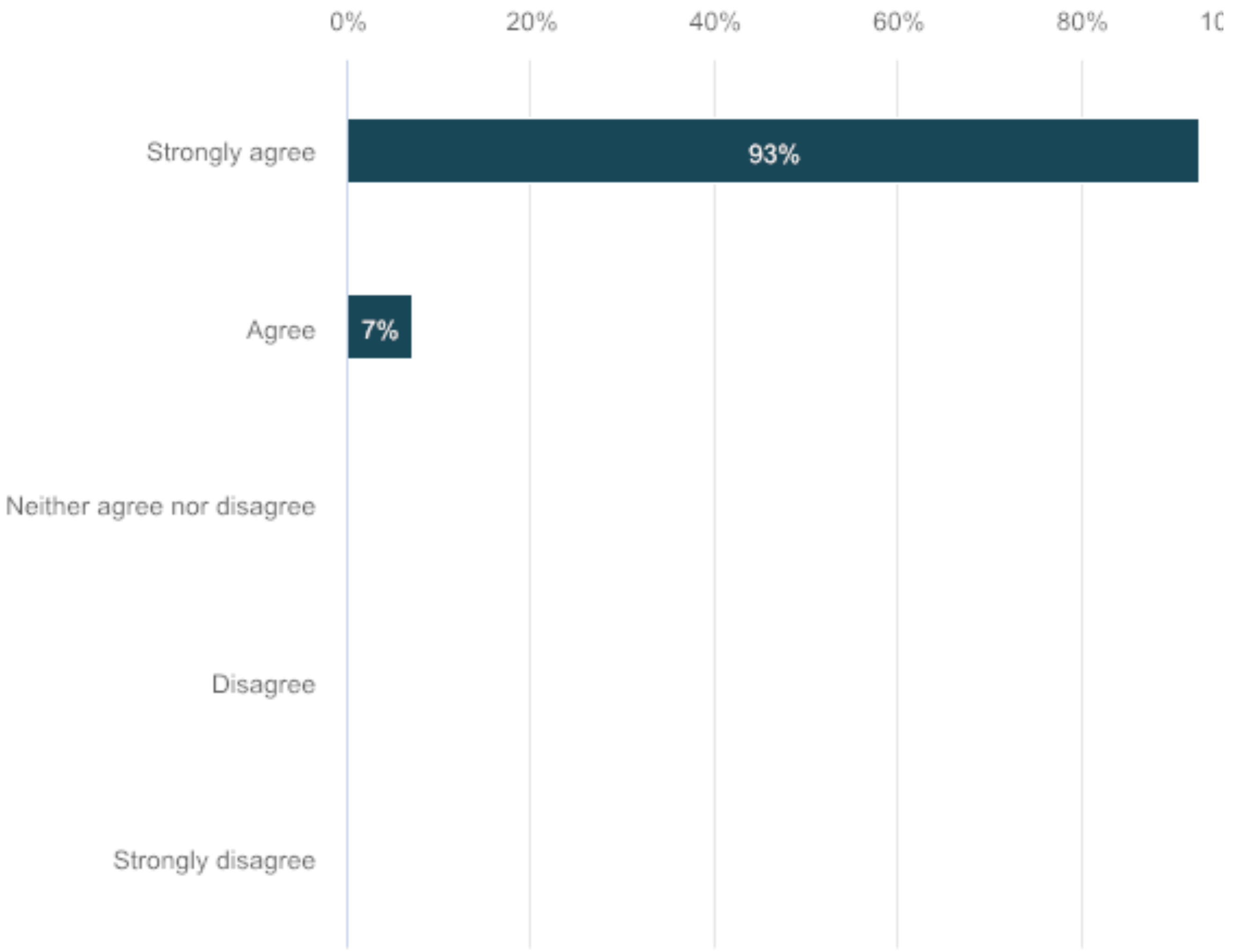

	n	Percent
Strongly agree	14	93.33%
Agree	1	6.67%
Neither agree nor disagree	0	0%
Disagree	0	0%
Strongly disagree	0	0%

60. Daily cone beam CT is recommended for treatment verification for conventionally fractionated re-irradiation.
Number of respondents: 15

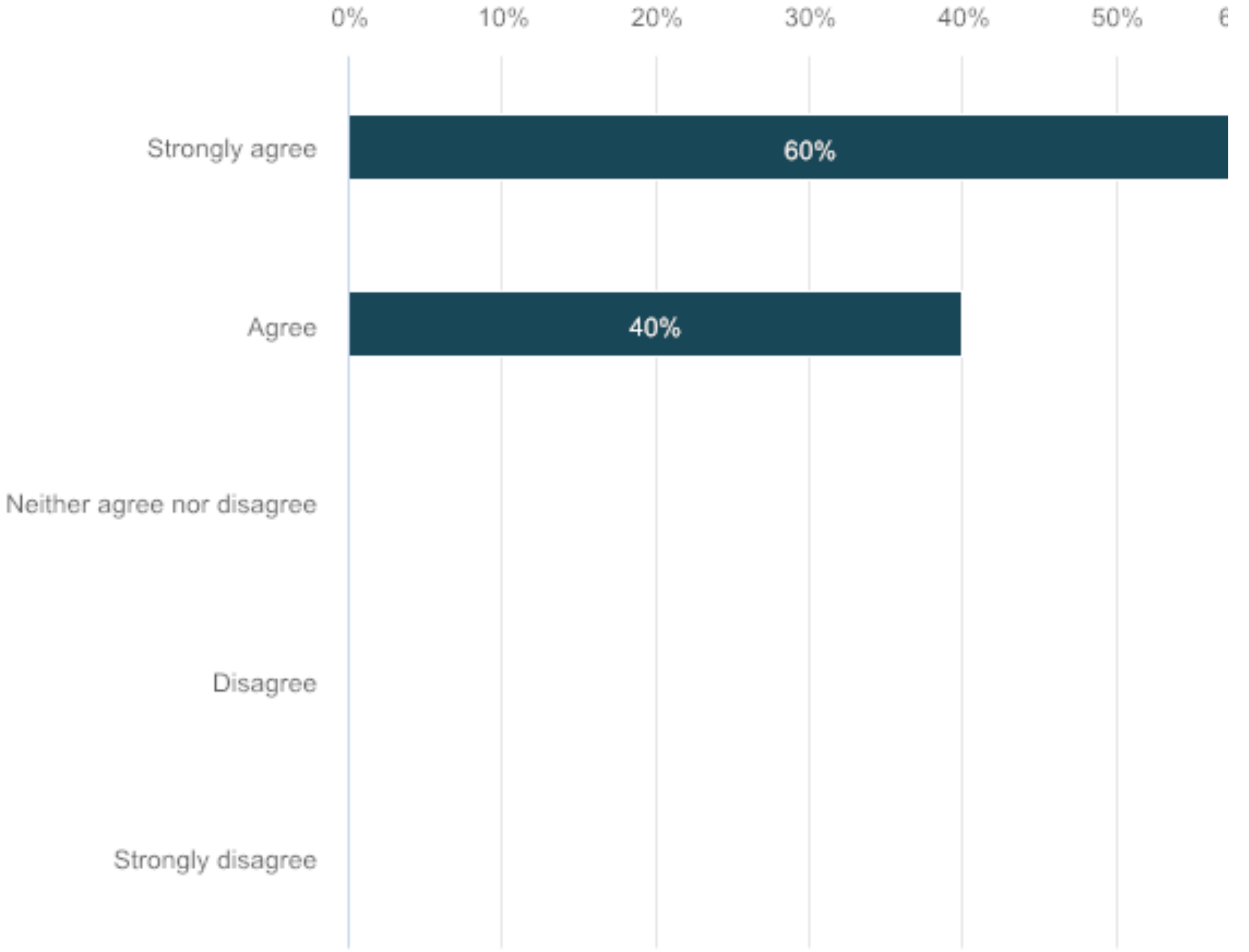

	n	Percent
Strongly agree	9	60%
Agree	6	40%
Neither agree nor disagree	0	0%
Disagree	0	0%
Strongly disagree	0	0%

61. Please add any comments here.

Number of respondents: 11

Responses
We should all aim to either deliver a definitive dose, or palliative dose. Some of the aforementioned dose/fxn are somewhere in between.
Again, difficult!<br><br>Ultra-central disease recurrent should not get SBRT.<br><br>Centrally located disease amenable to SBRT should get risk adapted treatment eg 60/8 or 50/10
none
#55 - the reality is that protons are widely used in the US and "not routinely used" is too strong.<br><br>#57 - I thought this was limited to NSCLC, based on the first screen<br><br>#58 - why 4 or more? We also have to note that BED >100 "if achievable safely". 60 Gy in 15 fractions would be "acceptable". Perhaps say "an acceptable option"?
The limited thoracic SABR re-rads literature would suggest that fewer than four fractions is acceptable. Full dose >100Gy BED is usually used, but i would be comfortable with 54Gy/3# to a primary failure.
I would not do BD fractination in BD treatment<br><br>I would say best to stick with 2 Gy fractions (except for SABR) and allow between 40-60Gy/ 20-30 fx, depedent on OARs<br><br>If one is unable to acheive min dose in PTV above 40Gy, no need for re-irradiation as will be palliative
Would not hypofractionate for conventionallyradical RT and would keep dose at 2Gy.fraction or less
56: Degree of hypo-fractionation depending on dose overlap<br>57: Prefer once-daily for re-RT<br>58: In general, prefer more fractionated SBRT schedules for re-RT (e.g. 5+ depending on location/OARs)
SABR may not be appropriate for reirradiation if there is significant lung fibrosis making identification of the tumour margin difficult
Question 53: despite I agree on VMAT in principle, 3D-CRT is able to achieve excellent SBRT plans. Additionally, it is hard to find arguments against older modulated techniques such as step-and-shoot IMRT. Additionally, VMAT does not cover Tomo or Cyberknife ...<br><br>Question 54: I would also be cautious in re-SBRT in central location. In

general I do not "like" the terms central or ultracentral but prefer to speak about dose overlap in critical serial organs.<br><br>Question 58: why minimum 4 fractions and not 3 ?
In terms of volume of PTV which will allow for re-SBRT, the typical rule of thumb might apply here - max dimension of 5 cm, following the rules of risk adapted fractionation ie. gentler 5 or 8 fraction schedulers for larger lesions vs higher dose or shorter schedules for smaller lesions.<br><br>Overlap with previous high dose area may not necessarily preclude the use of SBRT. Instead, it is probably more important to consider the OARs in the re-irradiation field. Eg. previous high dose to a section of the cord will probably preclude the use of high dose SBRT in the same area. Conversely, previous high dose to the lung may be ok, provided there is a long interval between treatments.

62. For radical re-irradiation, the desirable cumulative dose constraints for the lung are:- Cumulative V5Gy <65%- Cumulative V20Gy <35%- Cumulative Mean lung dose <20Gy

Number of respondents: 15

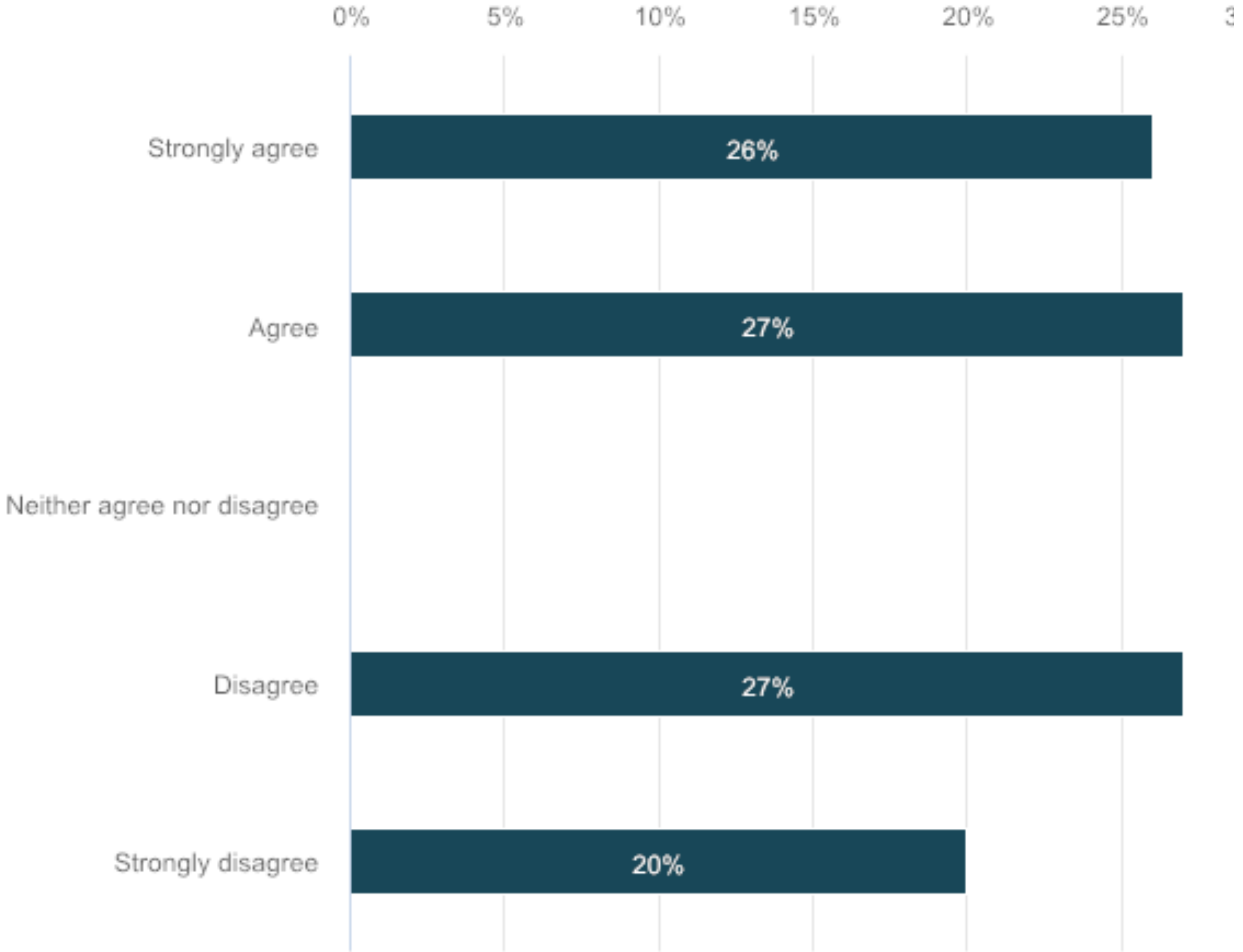

	n	Percent
Strongly agree	4	26.66%
Agree	4	26.67%
Neither agree nor disagree	0	0%
Disagree	4	26.67%
Strongly disagree	3	20%

63. For radical re-irradiation, the desirable cumulative maximum point dose (Dmax) constraint to the bronchial tree is an EQD2 of 80Gy (using an a/b=3) although an EQD2 up to 105Gy is acceptable.

Number of respondents: 15

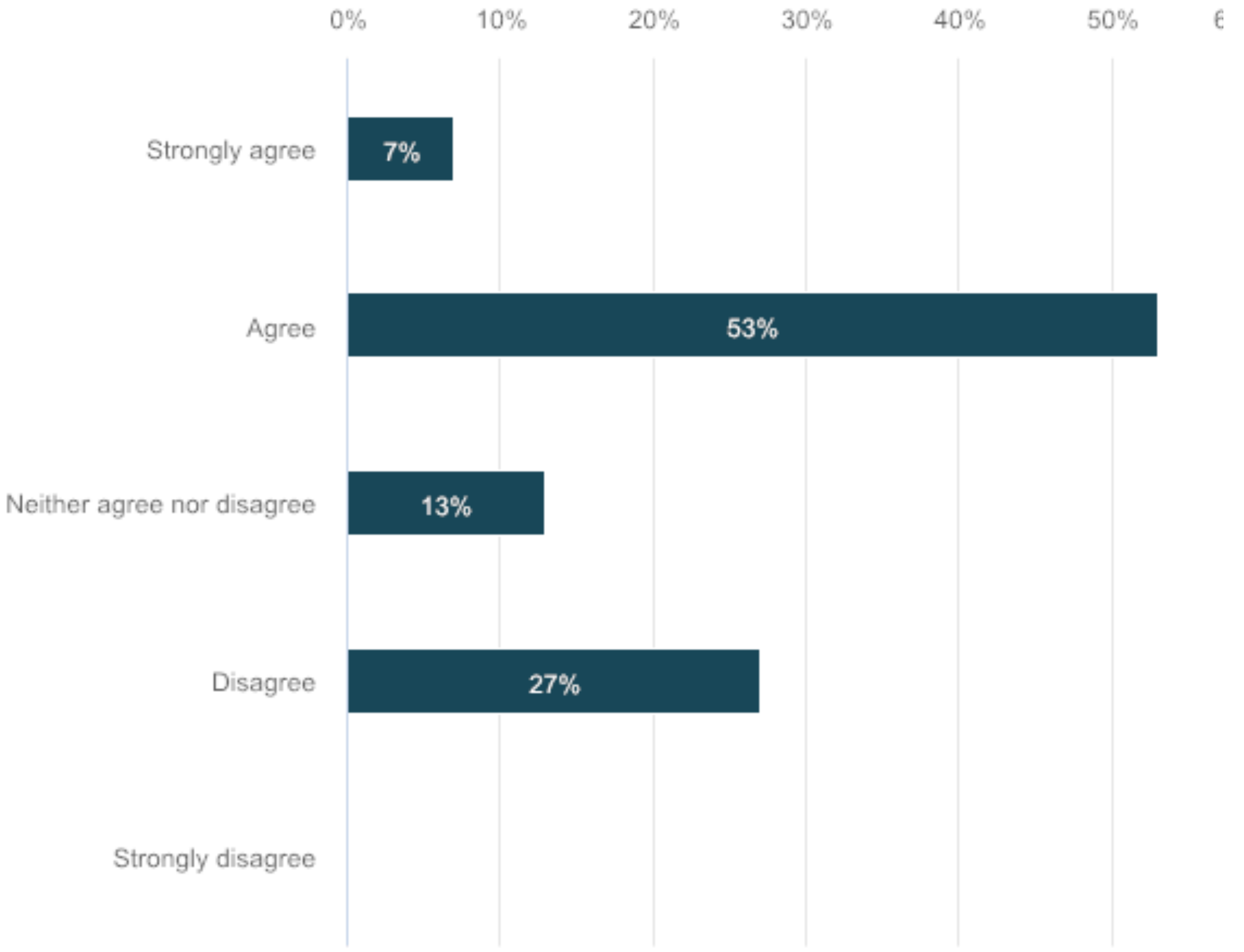

	n	Percent
Strongly agree	1	6.67%
Agree	8	53.33%
Neither agree nor disagree	2	13.33%
Disagree	4	26.67%
Strongly disagree	0	0%

64. For radical re-irradiation, the desirable cumulative maximum point dose constraint to the oesophagus is an EQD2 of 75Gy, although up to 100Gy is acceptable (using an a/b=3), with the volume of the oesophagus getting 55 Gray should be less than 35% (V55Gy<35%).

Number of respondents: 15

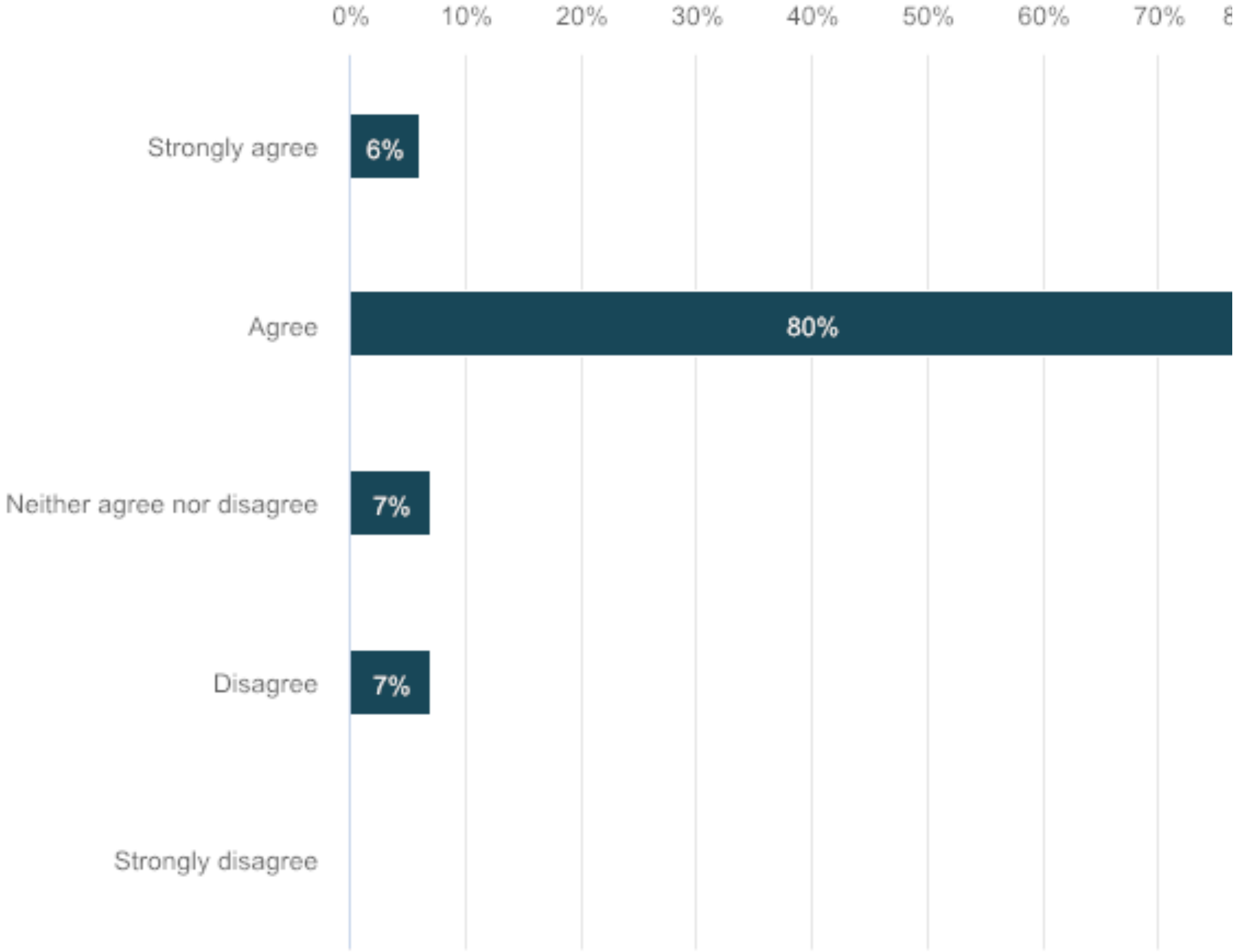

	n	Percent
Strongly agree	1	6.66%
Agree	12	80%
Neither agree nor disagree	1	6.67%
Disagree	1	6.67%
Strongly disagree	0	0%

65. For radical re-irradiation, the desirable cumulative maximum point dose constraint to the spinal cord is an EQD2 of 60Gy (using a/b=2), with a maximum EQD2 of 67.5Gy (provided that the initial re-irradiation dose to the cord did not exceed 50Gy.

Number of respondents: 15

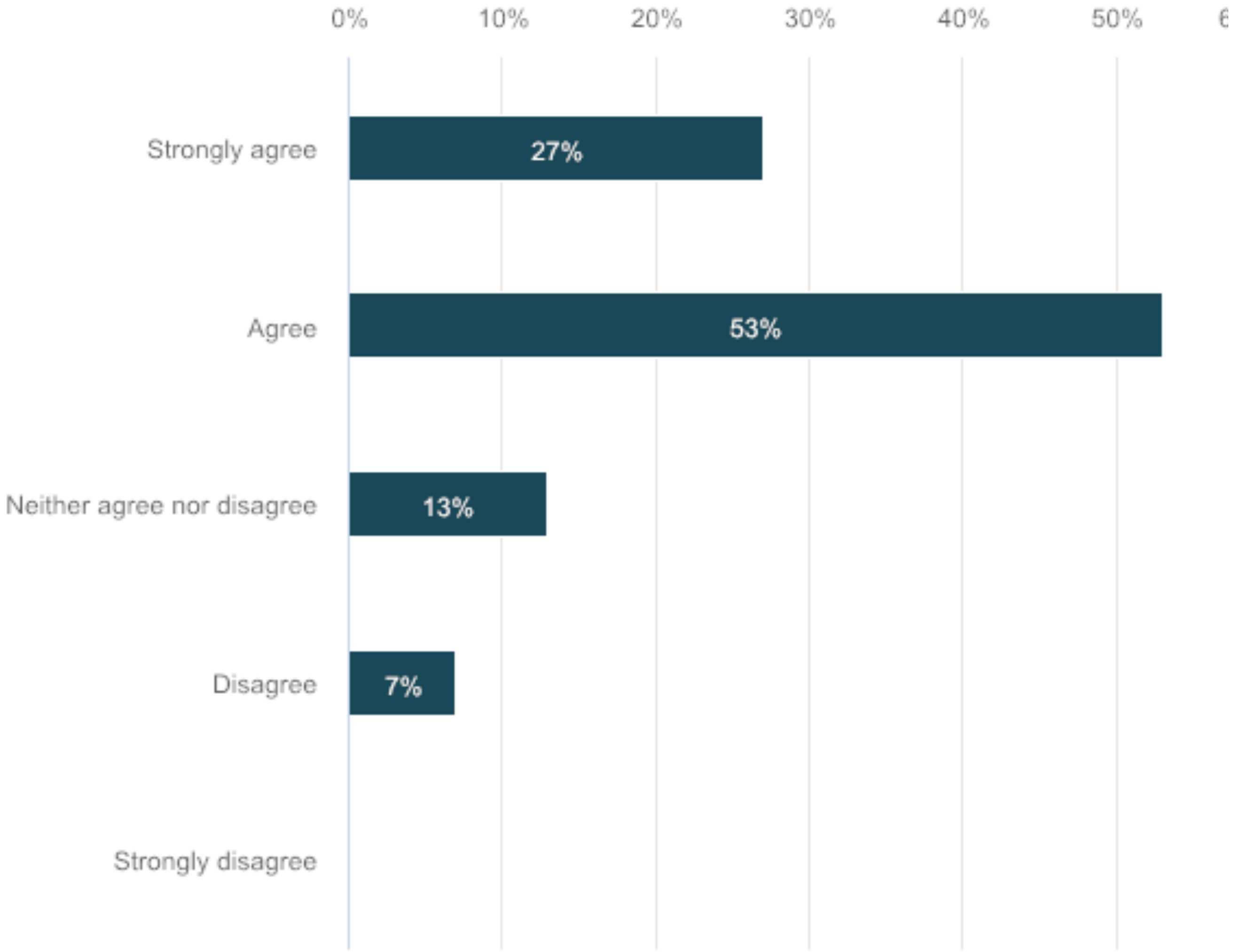

	n	Percent
Strongly agree	4	26.67%
Agree	8	53.33%
Neither agree nor disagree	2	13.33%
Disagree	1	6.67%
Strongly disagree	0	0%

66. For radical re-irradiation, the desirable cumulative maximum dose (Dmax) constraint to the brachial plexus is an EQD2 of 80Gy (a/b=2) and an acceptable cumulative Dmax is 95Gy (if the interval between treatments is greater than 2 years).

Number of respondents: 15

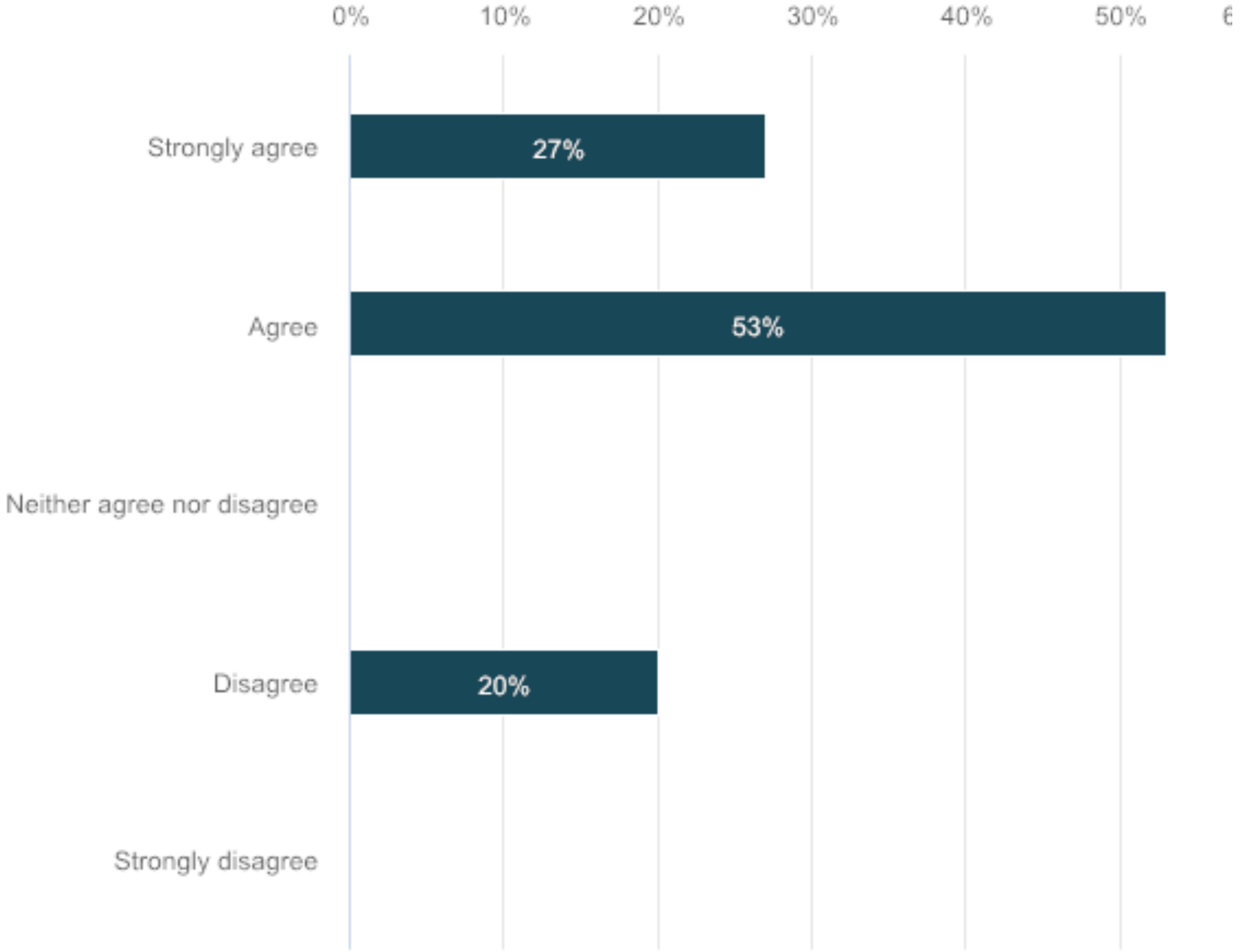

	n	Percent
Strongly agree	4	26.67%
Agree	8	53.33%
Neither agree nor disagree	0	0%
Disagree	3	20%
Strongly disagree	0	0%

67. For radical re-irradiation, the desirable cumulative maximum dose (Dmax) constraint to the aorta is an EQD2 of 120Gy (a/b=3). The desirable cumlative Dmax to the pulmonary artery is an EQD2 of 110Gy.

Number of respondents: 15

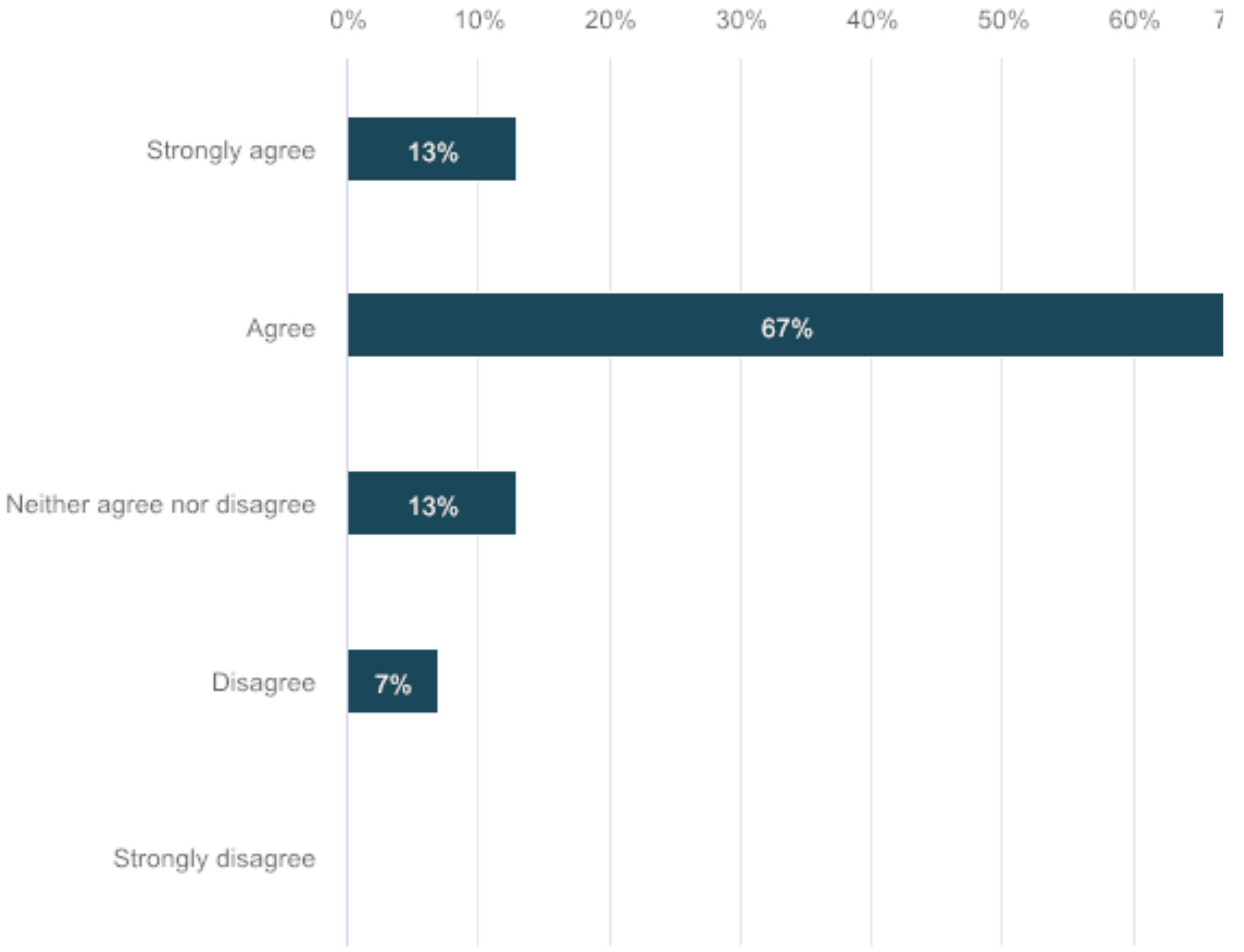

	n	Percent
Strongly agree	2	13.33%
Agree	10	66.67%
Neither agree nor disagree	2	13.33%
Disagree	1	6.67%
Strongly disagree	0	0%

68. There is a lack of information to guide re-irradiation dose constraints for the skin and the heart, therefore the use of other guidelines (e.g. QUANTEC or SABR guidelines) and to keep the dose to these organs as low as reasonably achievable are recommended.
Number of respondents: 15

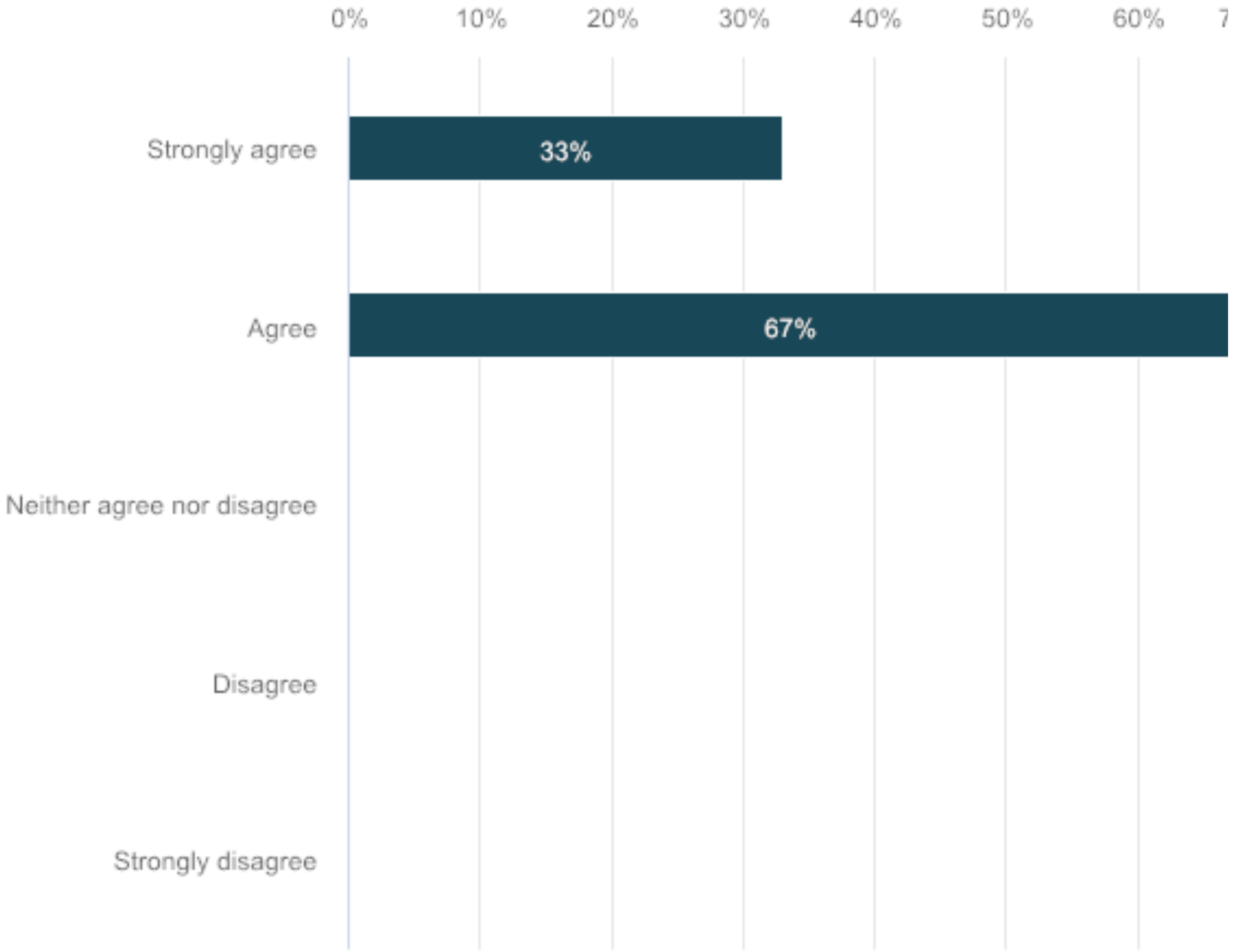

	n	Percent
Strongly agree	5	33.33%
Agree	10	66.67%
Neither agree nor disagree	0	0%
Disagree	0	0%
Strongly disagree	0	0%

69. Please add any comments here.

Number of respondents: 11

Responses
I do not agree with any V5 constraint
Very difficult issue. There are time issues here to be considered (time interval between initial primary and recurrent cancer). However, we also need to remain pragmatic due to the heteregenous nature of recuurent malignancy. I could accpet all proposals above in a trial protocol, but it may need to be amended based on clinical exeriences
None of the dose constraints are well supported. Even in the primary setting, the contraints have a poor predictive value, typically AUC 0.65-0.70. The NPV uncertitude is high. The follow-up is typically short in these series. Nobody knows what the recovery is, certainly not at high cumulative doses.<br><br>I therefore consider ALL re-irradiation always as experimental, except in highly exceptional cases.
#62 - that V5 is very low and many patients with upfront treatment don't meet that.<br><br>#67 - I don't think there's really evidence to say that the PA and aorta should be different, apart from those two values used. We should just quote the range of 110-120 for Aorta and PA.
In my experience, it is not feasible to constrain to the original lung volumetric, particularly after prior chemoRT for stage III. I usually allow a 50% discount to values and aim for the same metrics listed here.
Int J Radiat Oncol Biol Phys. 2012 Jan 1;82(1):107-16. doi: 10.1016/j.ijrobp.2010.08.021. Epub 2010 Oct 15.<br><br>Reirradiation human spinal cord tolerance for stereotactic body radiotherapy.<br><br>Sahgal A1, Ma L, Weinberg V, Gibbs IC, Chao S, Chang UK, Werner-Wasik M, Angelov L, Chang EL, Sohn MJ, Soltys SG, Létourneau D, Ryu S, Gerszten PC, Fowler J, Wong CS, Larson DA.<br><br>We use this for SC when we prescribe SABR when previous was conventional
Think for all these the key is informed consent and peer review. In addition for spinal cord re-treatment I would like dose interval included ? 6 or 12 months to allow for some recovery before giving a combined dose of 60Gy and would be nervous about going to 67.5Gy.
62,63,66,67: We are not using cumulative lung constraints; we allow more dose into parts of the other OARs (and do not distinguish between aorta and PA)<br>64: Not sure about the basis for the 55Gy volume constraint, but agree that a steep dose fall-off in the oesophagus is most desirable<br>65: Agree with the cumulative Dmax of 60Gy in 2Gy/fr with a/b=2 for thoracic re-RT (but not the 2nd part of the statement); spine SBRT different situation

Regarding lung, if these are cumulative doses, it will severely limit the volume and retreatment dose that can be given. Bronchial tree: how defined? I could not access the Feddock article.<br>Oesophagus: Will depend on grade of toxicity experienced with first course; also if concomitant chemotherapy is given.
It will be important to stress that "these are not absolute limits and may need to be exceeded given the clinical situation"
For 62) while the cumulative dose constraints stated above are desirable, a cumulative dose of V5<65% may be prohibitive especially if there is minimal overlap between fields and there is significant fibrosis post first course of RT. If the interval is long (eg >6 mo), it may be reasonable to consider the re-RT dose to lung, on its own.

70. In patients who are fit for further treatment after radical re-irradiation, surveillance CT is recommended every 3 to 6 months for the first 2 years, then 6 to 12 monthly thereafter.
Number of respondents: 15

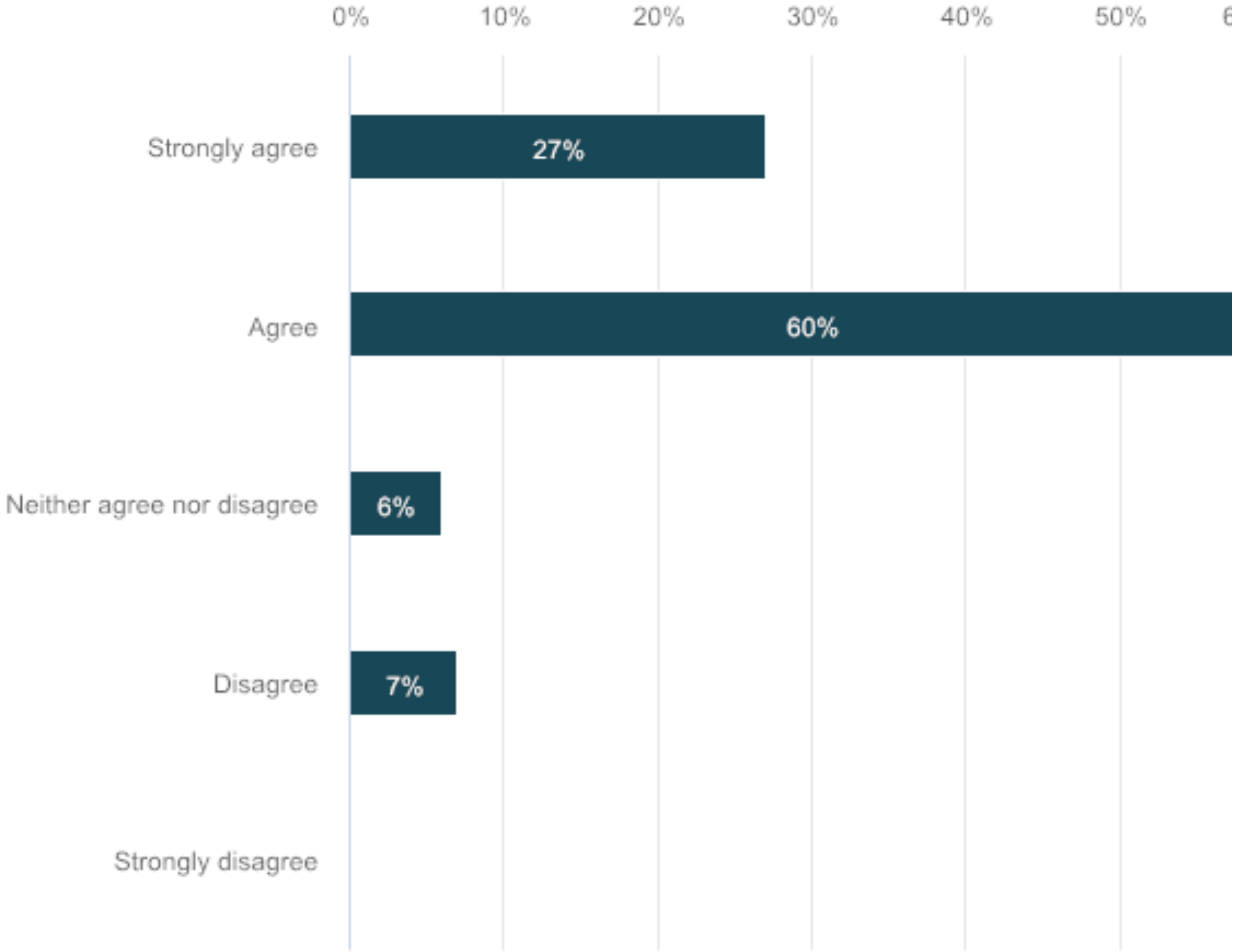

	n	Percent
Strongly agree	4	26.66%
Agree	9	60%
Neither agree nor disagree	1	6.67%
Disagree	1	6.67%
Strongly disagree	0	0%

71. Please add any final comments here.
Number of respondents: 5

Responses
Seems reasonable. Imaging is required to provide outcome data on clinical effectiveness
what does just a CT add? We do it, but what does it learn? What are the therapeutic consequences after 2x high-dose re-irradiation?
Nice work!
I agree with imaging up to 2 years.<br><br>However, is there evidence for further imaging after 2 years?
No evidence for CT surveillance in lung cancer and with limited resources think 6 monthly is acceptable but 3 monthly is too frequent.

An international Delphi consensus on the use of radical thoracic re-irradiation and acceptable cumulative dose constraints - Round 3

Total number of respondents: 15

1. Please enter your identification number (found on the e-mail with the link to this survey)

Number of respondents: 15

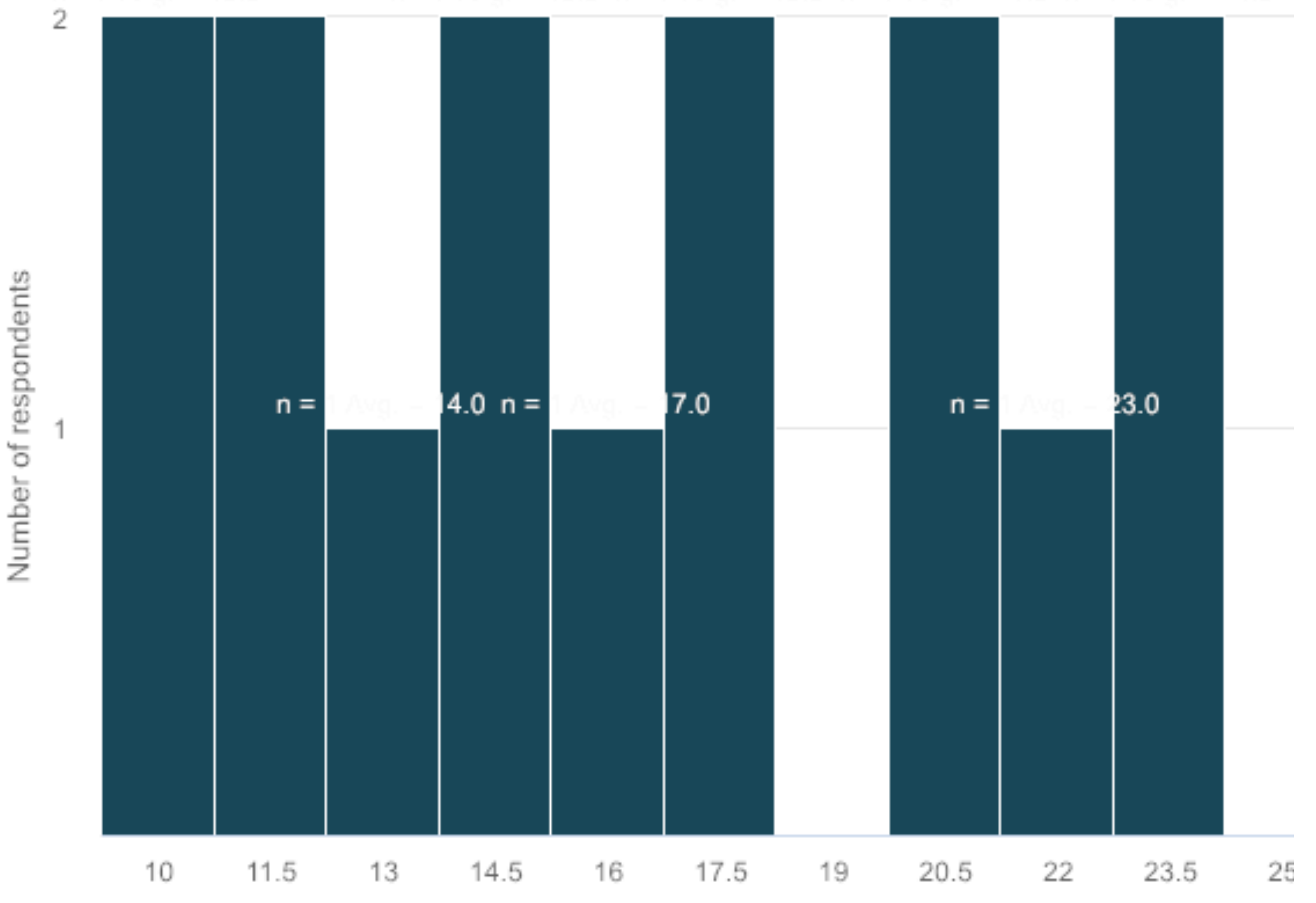

	Min value	Max value	Average	Median	Sum	Standard Deviation
	10	25	17.33	17	260	4.88

2. Any dose of radical radiation for lung cancer, after prior radiotherapy to the thorax or surrounding tissues for any tumour histology:(1) where the contribution of the first course of radiotherapy exceeds the prescription dose of the second planning target volume, and/or;(2) where the contribution of the planned second dose exceeds the institution's OAR dose constraints of a single course of radical lung radiotherapy (with no consideration for repair).

Number of respondents: 15

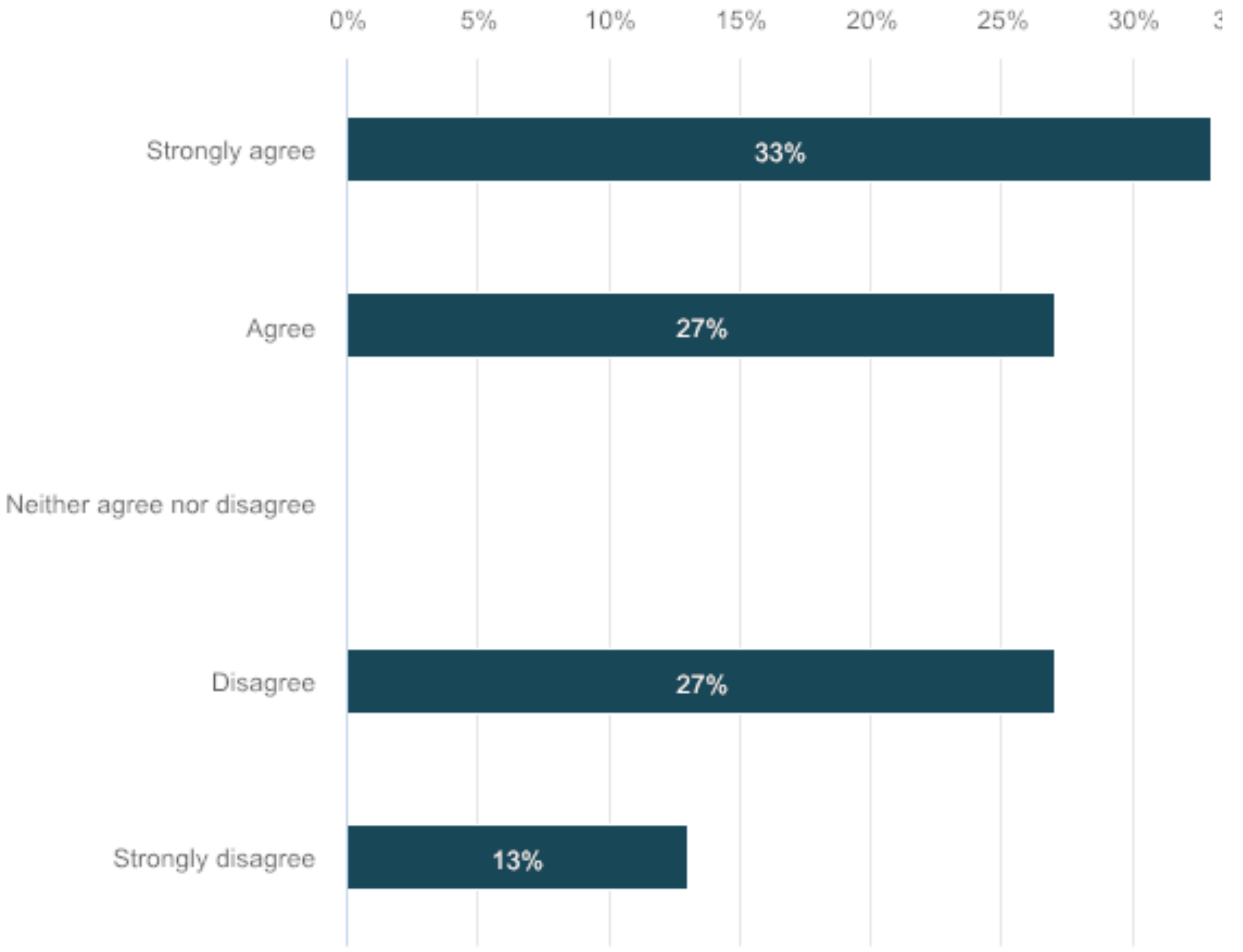

	n	Percent
Strongly agree	5	33.33%
Agree	4	26.67%
Neither agree nor disagree	0	0%
Disagree	4	26.67%
Strongly disagree	2	13.33%

3. Please add any comments about either definition here:

Number of respondents: 11

Responses
I think this definition, unfortunately, is no better than the first two. As a simple example, you could give SBRT twice to the same lesion and not meet this definition. You also could in theory treat several locations, some more than once, and still not meet this definition. Perhaps work with definition #1 above and try to adjust and get feedback to get consensus.
That is a great solution to this dilemma. Nice work!
definition 2 makes most sense
I prefer the first definition that was proposed.
It is becoming quite a complex definition. Entirely understandable though!
I still prefer on balance, option A from Round 2:<br>"Any dose of radical radiation for lung cancer, after initial radical radiotherapy to the thorax or surrounding tissues for any tumour histology, provided there is any overlap of previous dose in either the PTV or the OARs"<br>I would add a comment that (1) attention should be given to non-standard OARs like the chest wall, and that (2) this definition differentiates overlapping treatments from non-overlapping treatments like SBRT for multiple primaries at distant locations, where cumulative, non-overlapping dose (e.g. lung) may still be an issue
1 allows for a wide range of doses and risks, some of which are likely to be minimal, but at least it flags a warning not to take the previous treatemnt for granted.
I agree with this def:<br><br>"Any dose of radical radiation for lung cancer, after initial radical radiotherapy to the thorax or surrounding tissues for any tumour histology, provided there is any overlap of previous dose in either the PTV or the OARs"<br><br>You can not say institutional OAR constraints as are different worldwide
The wording is quite complex, I would prefer the one given above:<br><br>Reirradiation is defined as a situation where the predicted cumulative dose of first course of radiotherapy and reirradiation received by any OAR or the PTV exceeds the dose constraints of a single course of radical radiotherapy to the lung with no correction for recovery.
I think this is a good compromise between the 2 views. I suggest that this nuance be discussed in detail in the manuscript
The wording of point (1) is very difficult. 'Contribution of 1st course exceeds the prescription dose of second PTV' - this is inherently always going to be true if the first course has any dose in the same region of the PTV, as any contribution > 0 Gy will trigger this point.<br><br>If this is indeed the intention, then the initial wording regarding " "Any dose of radical radiation for lung cancer, after initial radical radiotherapy to the thorax or surrounding tissues for any tumour histology, provided there is any overlap

of previous dose in either the PTV" is a much clearer way of describing this concept

4. When re-irradiating patients (where the planned cumulative doses to the PTV exceed the initial prescription dose and/or where the cumulative dose to any OAR exceeds the dose constraints from a single course of radical treatment), the minimum interval between the first and second treatment is:
Number of respondents: 15

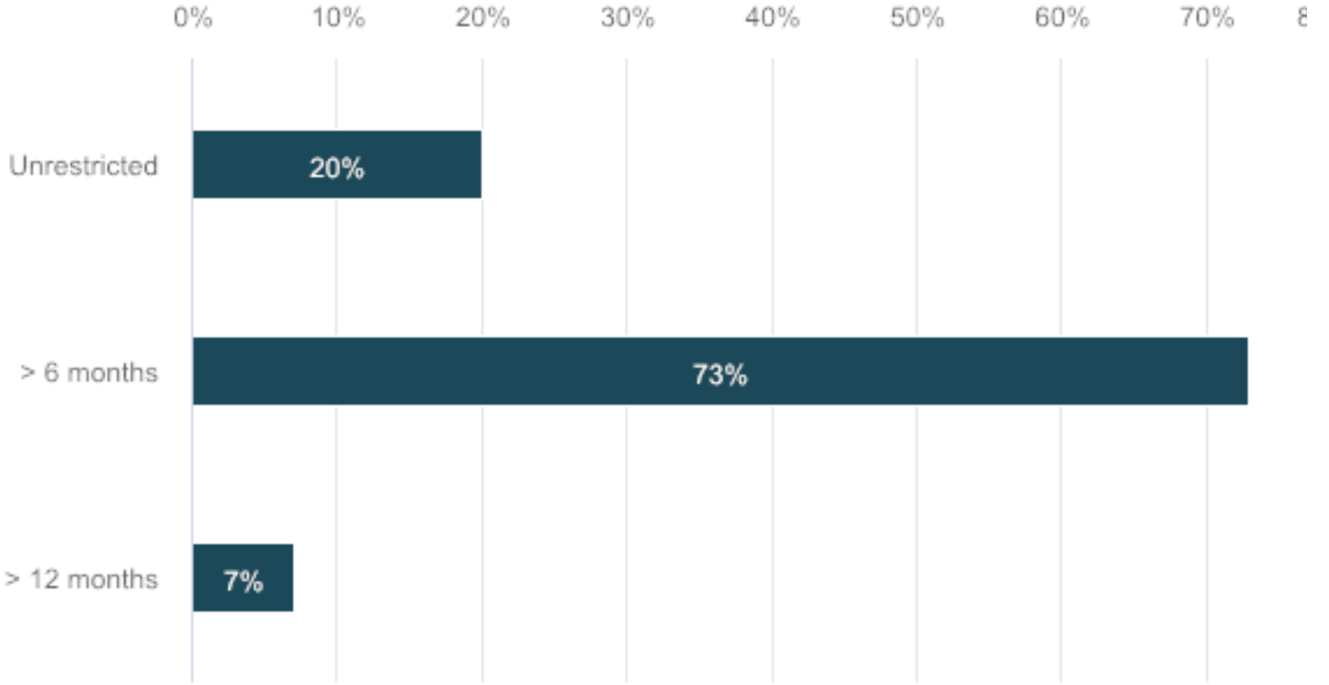

	n	Percent
Unrestricted	3	20%
> 6 months	11	73.33%
> 12 months	1	6.67%

5. Please add any comments regarding the interval between treatments here:

Number of respondents: 9

Responses
I would remove the parantheses.
I would be OK with either 6 or 12 months.
There is simply no evidence base to restrict the timing of re-irradiation. However, there should be a caveat that local recurrence must be clearly demonstrated to consider re-irradiation eg PET-Positive growing diease or biopy proven recurrrence. Most patients are re-imaged at least 3/12 after completion of primary RT, so will be very rare to have local recurrences within 3/12 period at least
I have answered "Unrestricted" on balance (since an answer is mandatory), but it is an imperfect answer; I think that the new definition of re-irradiation makes this harder to answer, with the risk that things are becoming over-complicated<br>On balance I preferred the Round 2 scenarios
It will depend on the OAR's, but if it is oesophagus or spinal cord, for example, some recovery must be permitted, so "unrestricted" is not an acceptable comprehensive answer.
but can allow higher doses if longer interval
Again, the term " planned cumulative doses to the PTV exceed the initial prescription dose" is very confusing. By definition, if there is any contribution of the second course then dose 'A + B' to the PTV will always be greater than 'A'.<br><br>I am answering this with respect to the OARs which is more important; i don't think it matters what time interval there is when looking at dose to PTV
But suggest softening to read..."the suggested minimum interval between the first and second treatment is:

6. Where a lung cancer has relapsed in both the previously irradiated primary tumour and nodes, there is no prospective evidence to guide the choice between radical re-irradiation or systemic therapy, and a clinical trial is warranted.

Number of respondents: 15

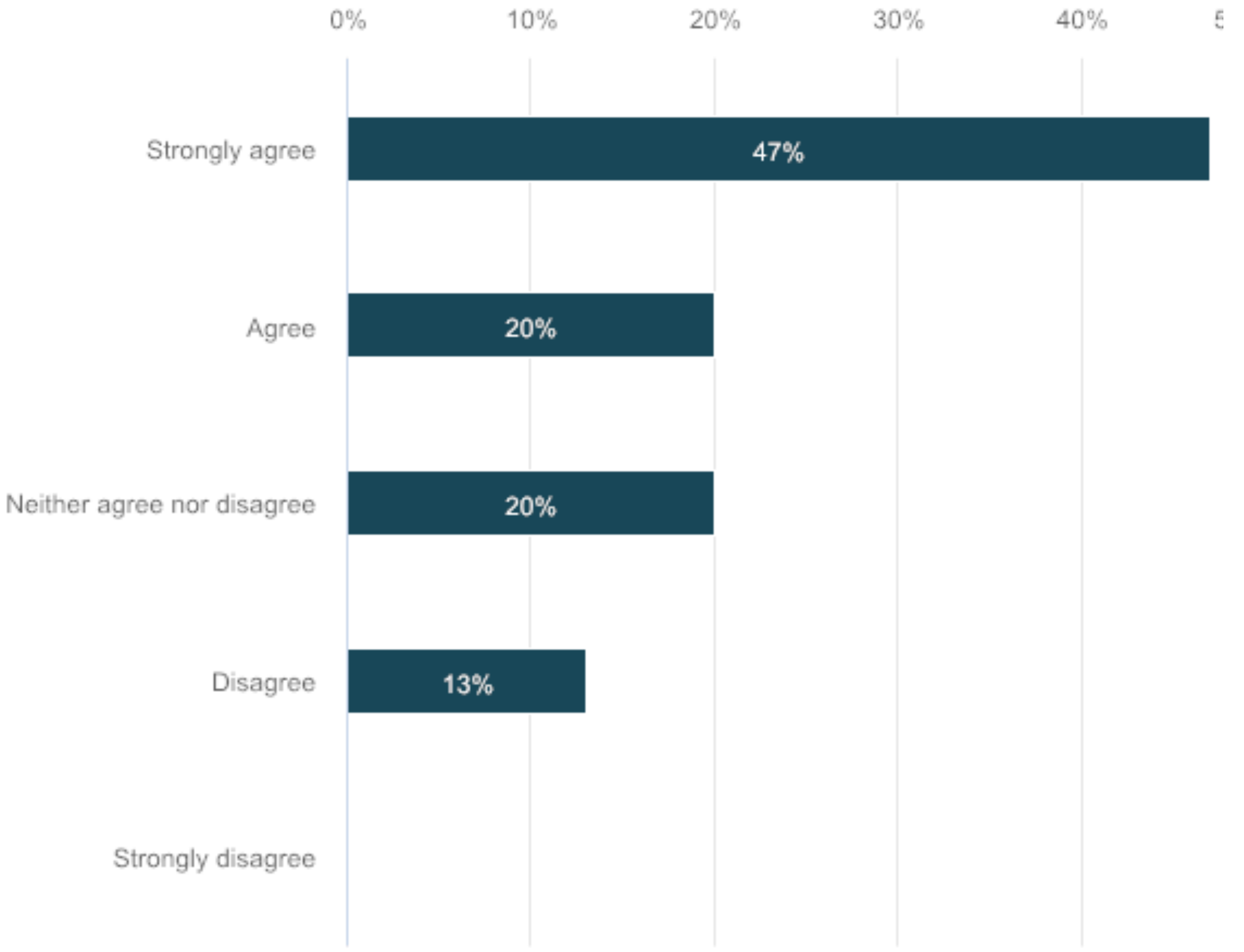

	n	Percent
Strongly agree	7	46.67%
Agree	3	20%
Neither agree nor disagree	3	20%
Disagree	2	13.33%
Strongly disagree	0	0%

7. Please comment here on the above statement.

Number of respondents: 8

Responses
Perhaps also re-send the original statement above and change to "Alternative treatment (e.g. systemic treatment) is GENERALLY preferable to radical re-irradiation...."
I prefer us to provide guidance, as opposed to conclude that there is a problem.
A clinical trial is the best way to asnwer the question, but it would be very hard to accrue the numbers. The trial would need to be international with several large countries recruiting.<br>I think we have to proceed on an individual case by case basis. Re-irradaition can cure. Targetted therapies and IO etc can be used on relapse. Very careful follow up is required if re-irradiation attempted.<br>However, there is a stronger case for systemic targeted therapy if EGFR/ALK/ROS 1 positive, or high PDL1 expression etc.
Depends on for example location of disease, volume and the other options (what they are [e.g. surgery, systemic, targeted, immunotherapy] and likelihood of benefit vs. risks)<br>Adequate and realistic consideration needs to given to whether or not the various scenarios/nuances can be realistically/adequately addressed in an RCT
The number of alternative treatment options to radical re-irradiation has fallen now that consolidation durvalumab is a standard of care for stage III disease post chemoradiation, so there is no obvious choice for a comparator in such a trial.
I would support a clinical trial, but this will likely be where all patients receive re-irradiation, then the trial is for adjuvant therapy.....
We should then add a statement that reirradiation can be considered in appropriately selected patients also outside of clinical trials.
I think the results of the initial question were reasonable, but perhaps with the exception of very long disease free interval.<br><br>Asking for a clinical trial is nice, but it doesn't help the reader all that much.

8. Where a lung cancer has relapsed both locally and with oligo-metastatic disease (less than 3 metastases, all mets treatable with radical radiotherapy), systemic treatment should be considered as the initial management, with subsequent radiotherapy to the primary +/- metastases to be considered in the context of a clinical trial.

Number of respondents: 15

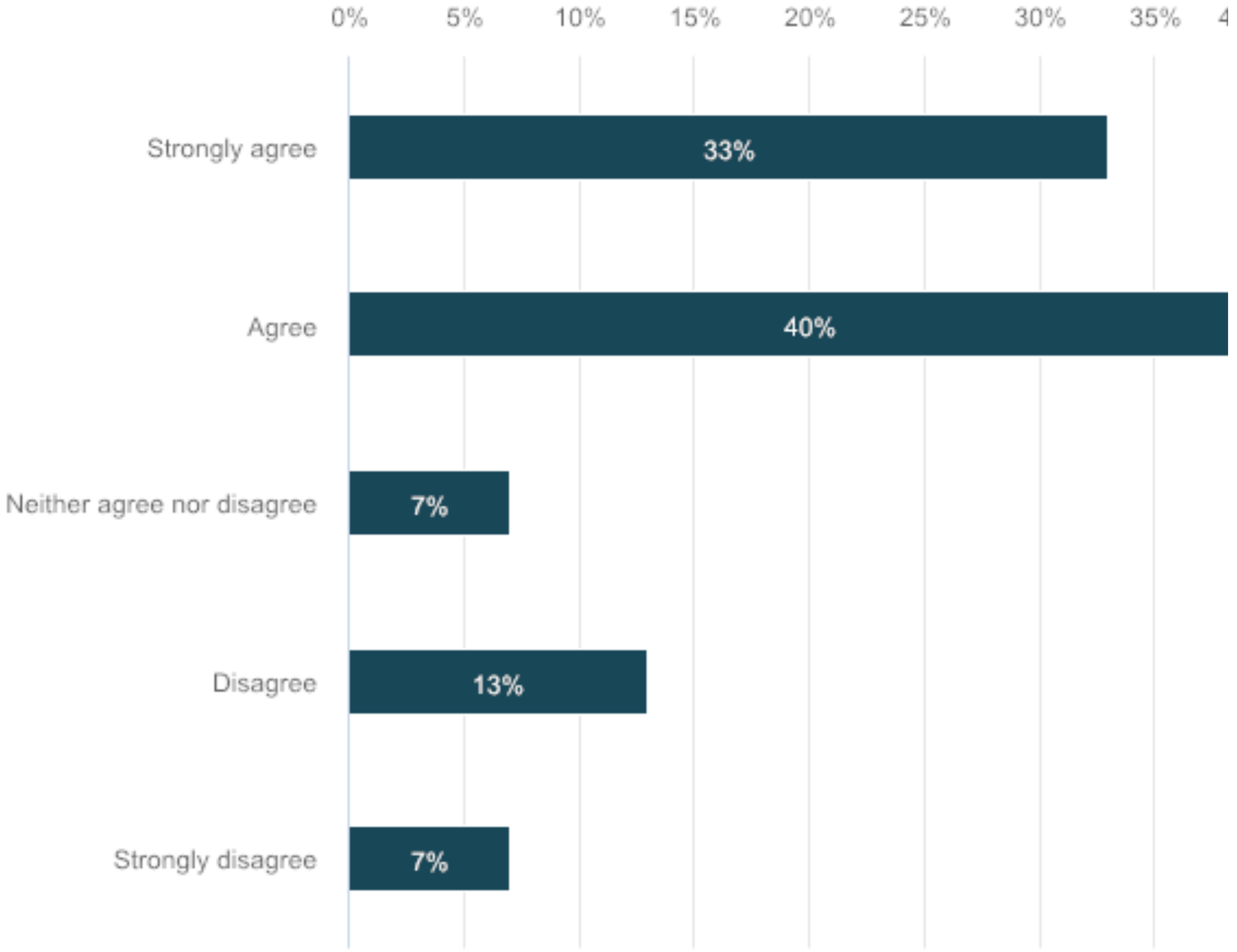

	n	Percent
Strongly agree	5	33.33%
Agree	6	40%
Neither agree nor disagree	1	6.67%
Disagree	2	13.33%
Strongly disagree	1	6.67%

9. Please comment on the above statement here.

Number of respondents: 6

Responses
Clinical trials are often not available - they probably will never be available for this specific question.<br>I would also add "GENERALLY" to this question and you will likely get consensus.
We should avoid restrictive clauses.<br><br>May want to clarify "...has relapsed both locally within a previously irradiated site..."<br><br>There is ambiguity about whether this statement is providing guidance or not. I am wondering if the word "only" was supposed to be inserted before "considered".<br><br>Why not go with this instead?<br><br>"It is appropriate to offer re-irradiation outside of a clinical trial to a patient who has developed local relapse within a previously irradiated field in addition to 3 or less oligometastatic lesions that can all be treated with radical radiotherapy"
Too restrictive<br>Maybe consider something like:<br>"Where a lung cancer has relapsed both locally and with oligo-metastatic disease (less than 3 metastases, all mets treatable with radical radiotherapy), the potential risks/benefits of local vs. systemic treatment, and their sequencing should be considered by the multidisciplinary team; if available a clinical trial may also be an option"
Some patients may develop local relapse that is now be surgically resectable, with surgery or SABR available for the oligometastases, but likelyy to be appropriate for only a small number of selected cases
As stated above, we should then add a statement that reirradiation can be considered in appropriately selected patients also outside of clinical trials.
PLEASE change the wording from "with subsequent" radiotherapy to "with radiotherapy to"<br><br>I would consider upfront RT rather than initial systemic therapy if considering aggressive local management.

10. Systemic therapies with a known risk of causing pneumonitis should not be combined with re-irradiation outside of a clinical trial.

Number of respondents: 15

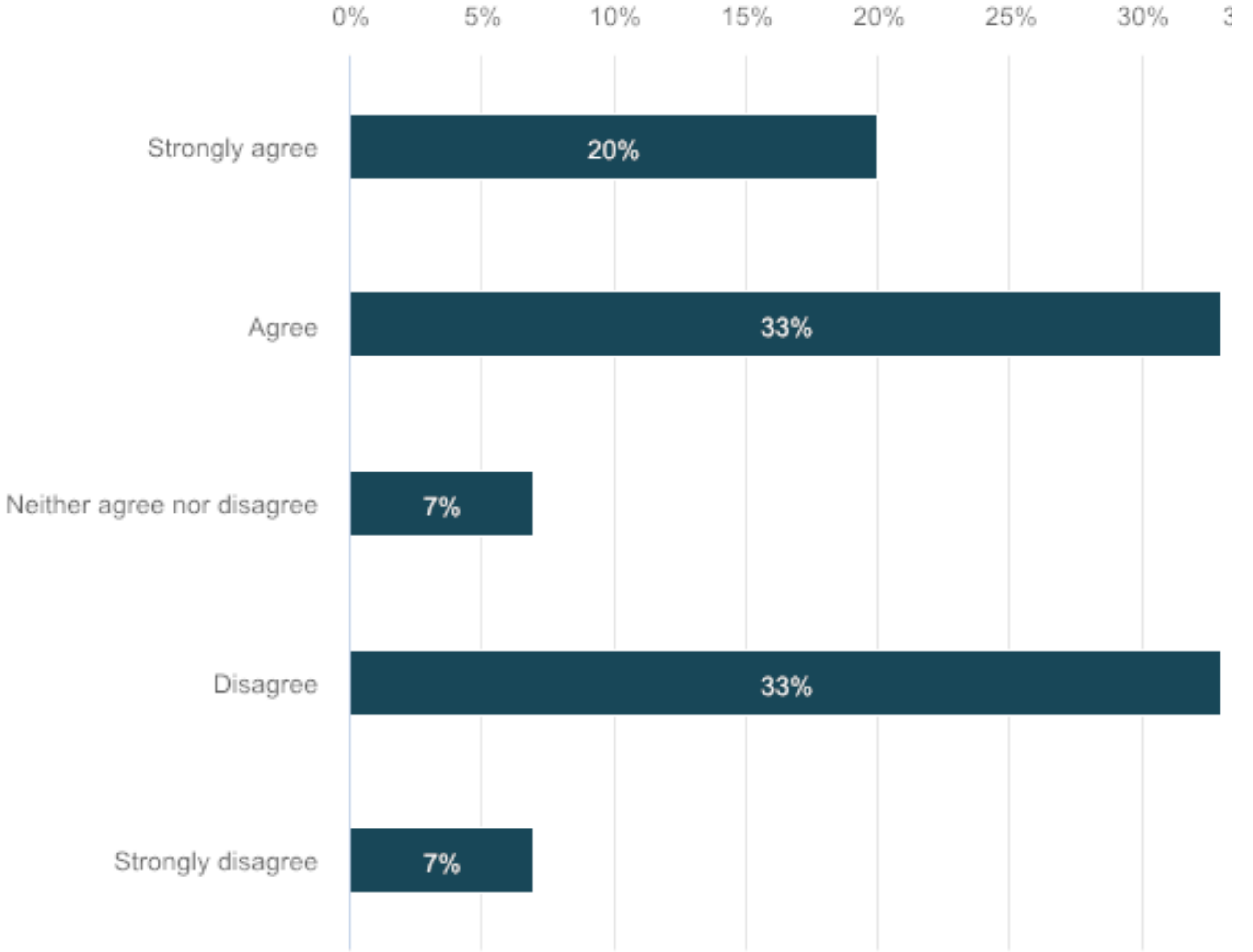

	n	Percent
Strongly agree	3	20%
Agree	5	33.33%
Neither agree nor disagree	1	6.67%
Disagree	5	33.33%
Strongly disagree	1	6.67%

11. Please add any comments here:

Number of respondents: 8

Responses
I disagree with this. There could be a situation where a stage I NSCLC is treated and then later a stage III with carbo-taxol (most common chemo in the US) with some overlap but very reasonable risk. Perhaps just say "should be used with caution."
If we agree on this statement, it is making a clinical recommendation without evidence that may affect patient outcomes. It may be better worded with more uncertainty and importance of shared decisions, since clinical trials aren't widely available to every one.
Risk with TKIs not that high. A trial will never accrue enough patients and will not answer the question. Individual decision again
Appropriate caution should be exercised considering possible risks of continuing/stopping the specific medication concerned; in addition, scheduling of RT and drug administration (e.g. avoiding same days) should be given appropriate consideration
I do not think there is good evidence that radiotherapy increases pneumonitis risk in patients receiving TKI's or immune check point inhibitors, but agree there is enough evidence to avoid concomitant gemcitabine.
There will most likely be no clinical trial testing this question, I would therefore suggest to aadd "consent the patient about the potential risk of added severe toxicity"
Do this all the time - treat with SABR + TKI and SABR + IO in oligoprogression.
Would change it to systemic anti-cancer therapies to make it clearer.

12. Consideration for biopsy must be made in a tumor board/multi-disciplinary team meeting before considering radical re-irradiation.
Number of respondents: 15

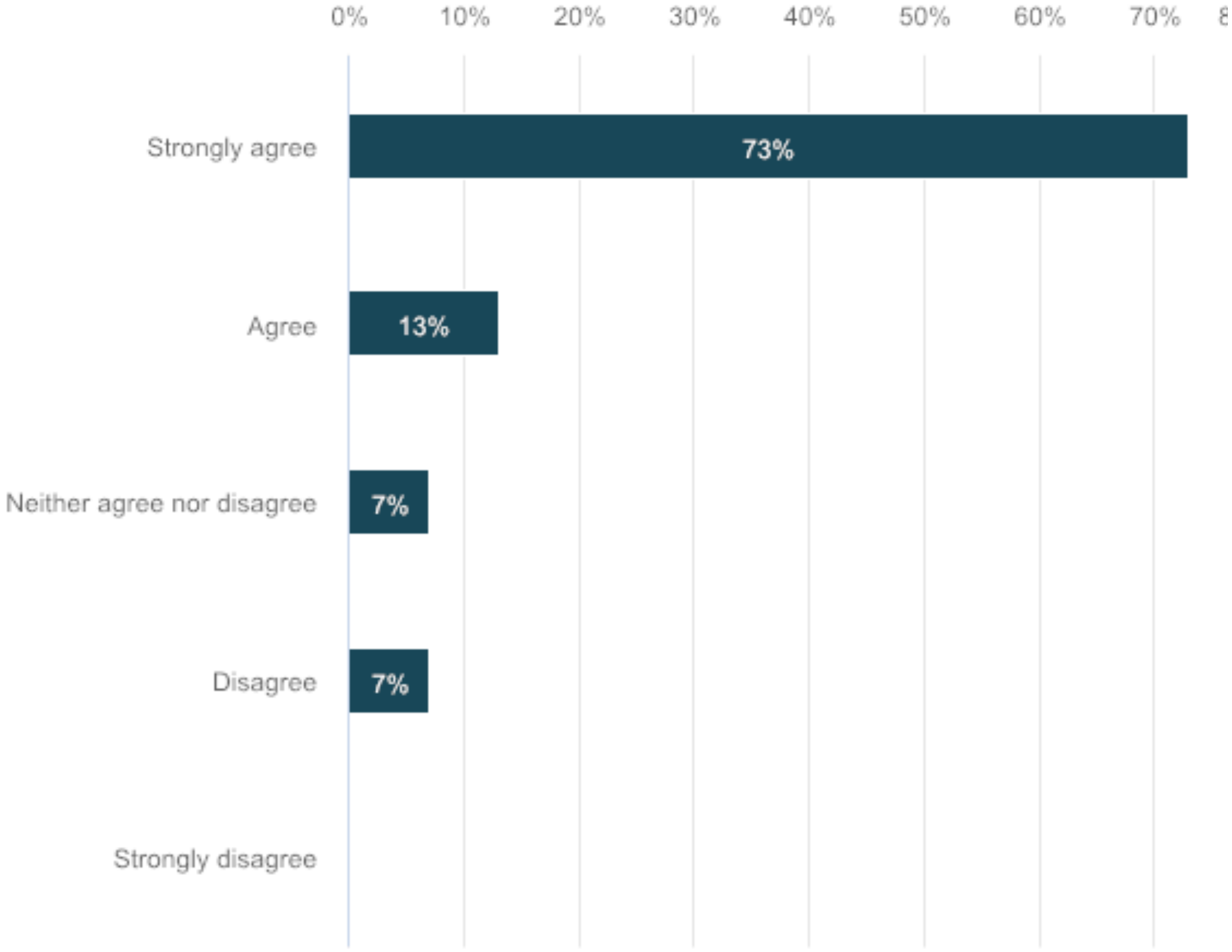

	n	Percent
Strongly agree	11	73.33%
Agree	2	13.33%
Neither agree nor disagree	1	6.67%
Disagree	1	6.67%
Strongly disagree	0	0%

13. Please add any comments here.

Number of respondents: 6

Responses
i like this statement
I don't see the role of tumor board adjudicating on something for which the treating physician is the final authority
Consideration is a good word here. I would even add the word "strong"
This is OK since it does not say that you have to perform a biopsy or that you need tissue conformation before re-RT; you only have to consider a biopsy
This applies to radical re-irradiation. The histology or the molecular characteristics may be different to the primary tumour, allowing for a wider range of options
"Must" is a very strong word. Better to word this as "should"

14. Re-irradiation can be considered where the tumor board/multidisciplinary team agrees that there is a high likelihood of cancer, but despite best efforts, histological confimation of cancer is not possible.

Number of respondents: 15

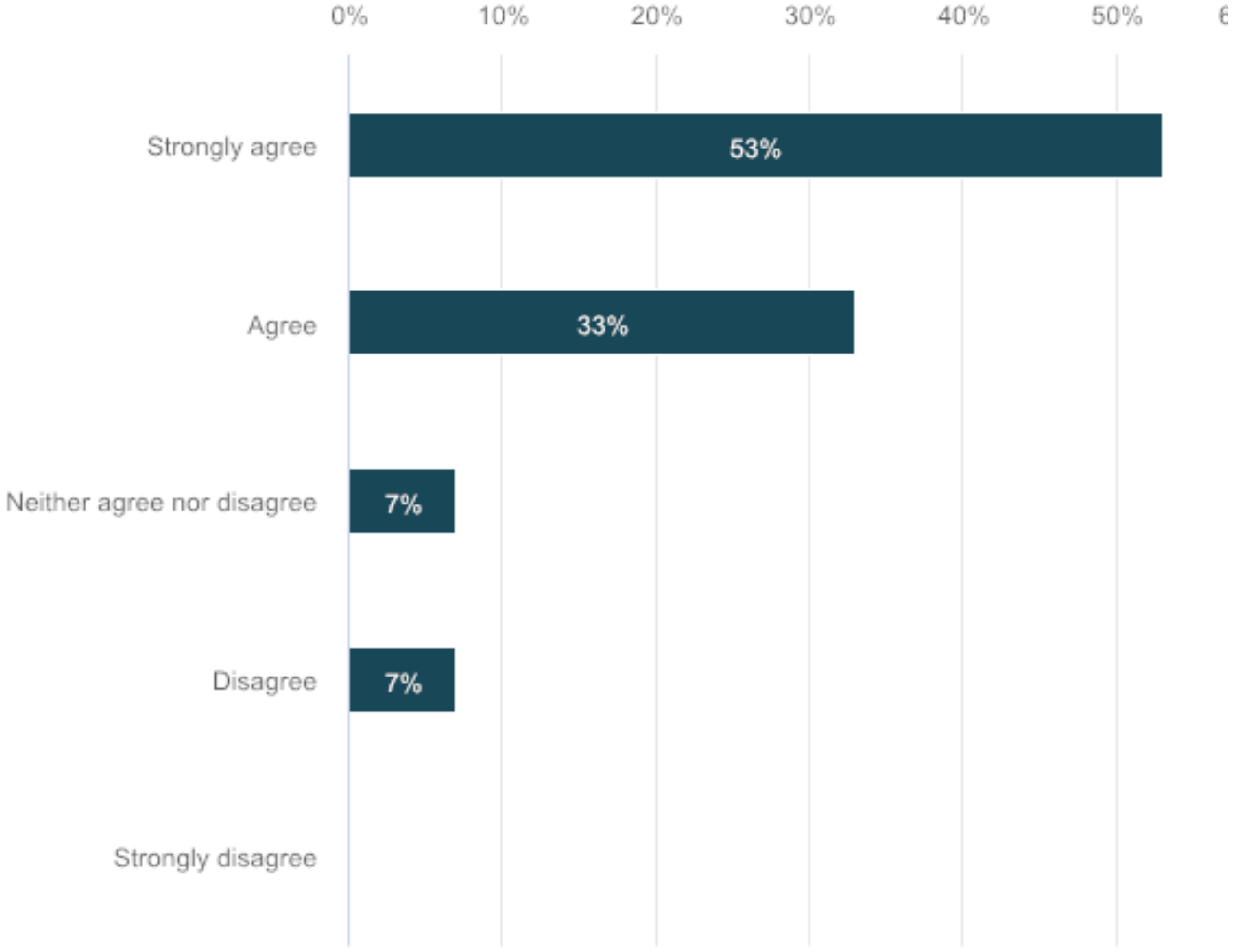

	n	Percent
Strongly agree	8	53.33%
Agree	5	33.33%
Neither agree nor disagree	1	6.67%
Disagree	1	6.67%
Strongly disagree	0	0%

15. Please add any comments here.

Number of respondents: 5

Responses
i like this too
this should be the treating MDs position, not relying on the board
I agree that re-RT can be done without histology, but I find this statement complicates things: I would remove the words "best efforts" - which suggest that an attempt should be made when that may not be appropriate - and "possible" - the definition of which can vary between centers; and suggest something like:<br>"Re-irradiation can be considered where the tumor board/multidisciplinary team agrees that there is a high likelihood of cancer, but does not think that histological confirmation in their hands is practical or appropriate (e.g. possible risks too high)"
Especially for palliative irradiation.
despite best efforts AND DUE TO A POTENTIALLY HIGH RISK OF TOXICITY

16. For conventionally fractionated re-irradiation, the clinician must consider re-treatment to have a positive risk/benefit ratio considering the current pulmonary function tests and the likely exposure of the lung to re-irradiation, with no minimum PFTs values applicable.

Number of respondents: 15

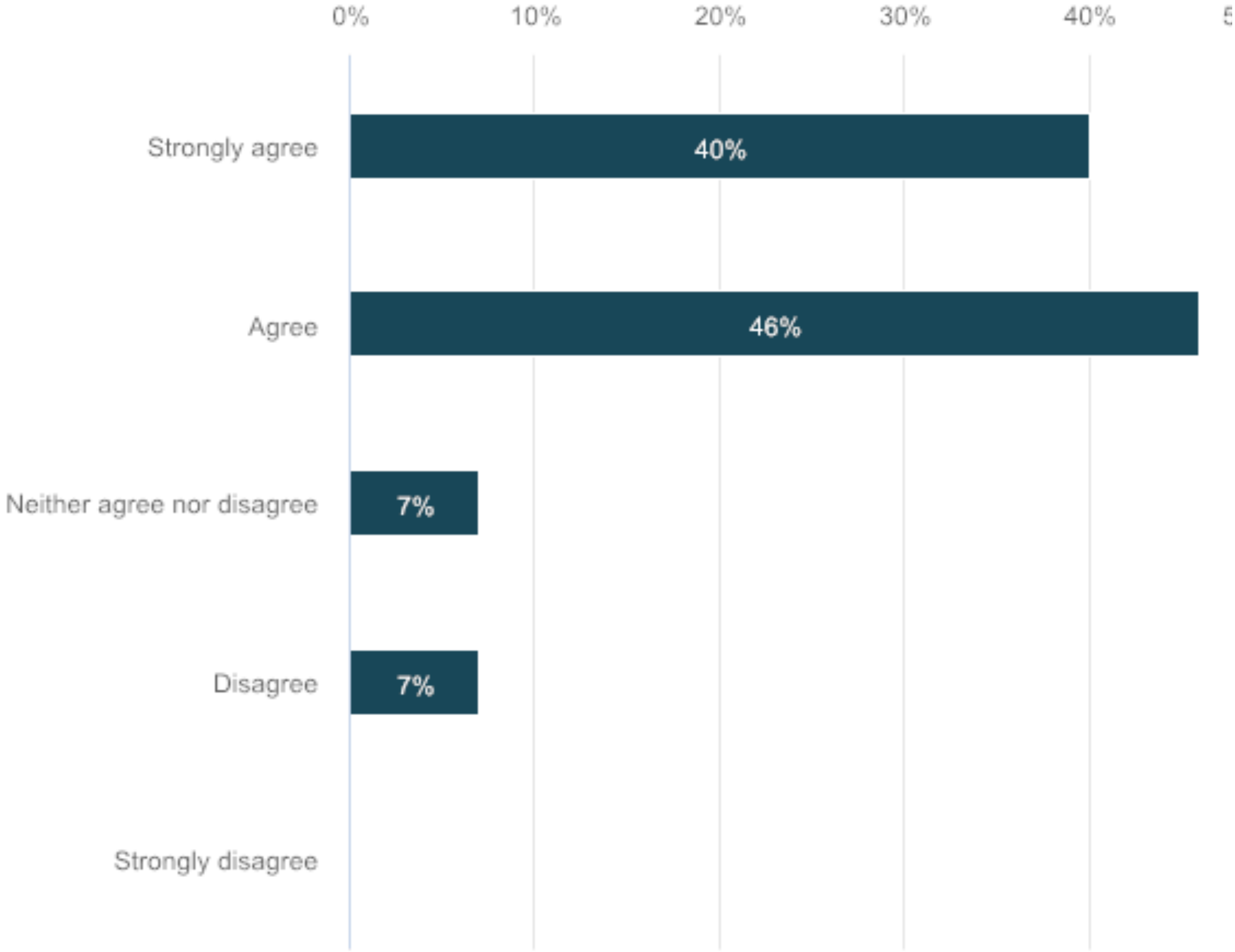

	n	Percent
Strongly agree	6	40%
Agree	7	46.66%
Neither agree nor disagree	1	6.67%
Disagree	1	6.67%
Strongly disagree	0	0%

17. Please add any comments here.

Number of respondents: 3

Responses
Much better
I think no minimum PFTs might be an issue for people here. I don't know what the values should be, but we should probably state have limits. Deterioration of lung funtion after first treatment should also instill caution
question is not understandable

18. Projected grade 1-2 toxicities have minimal influence on the decision to offer re-irradiation, unless deemed significant after discussion with the patient.

Number of respondents: 15

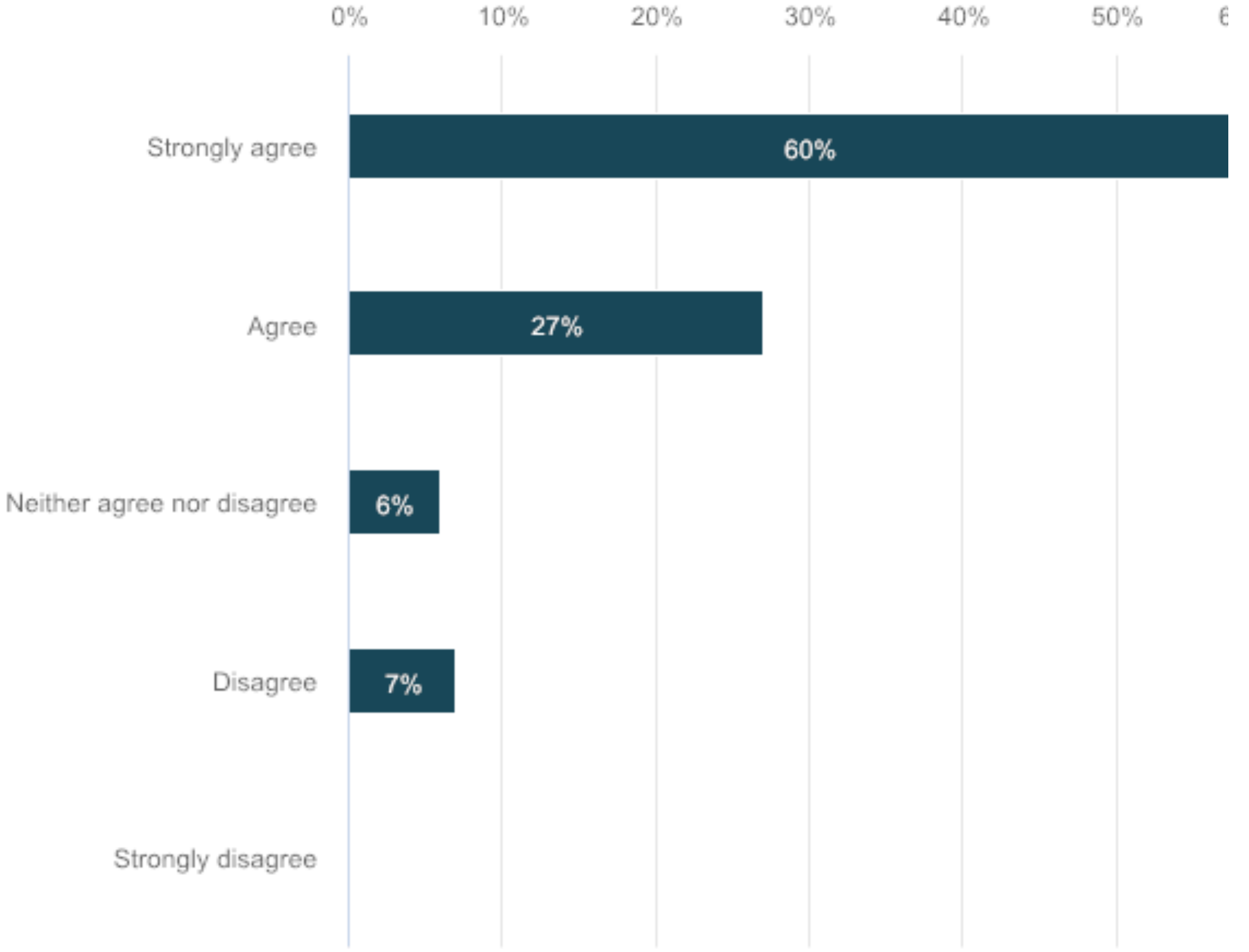

	n	Percent
Strongly agree	9	60%
Agree	4	26.66%
Neither agree nor disagree	1	6.67%
Disagree	1	6.67%
Strongly disagree	0	0%

19. Please add any comments here.

Number of respondents: 3

Responses
I think this is too dismissive of G2 toxicities - like RP requiring steroids - I would state that "Projected grade 1-2 toxicities usually have a minor influence on the decision to offer re-irradiation, unless deemed significant after discussion with the patient"
I would add the word "should" before "have"
what is "projected"?

20. For cumulative dose constraints, the amount of normal tissue recovery has limited evidence and therefore the safest approach is to assume no tissue recovery.
Number of respondents: 15

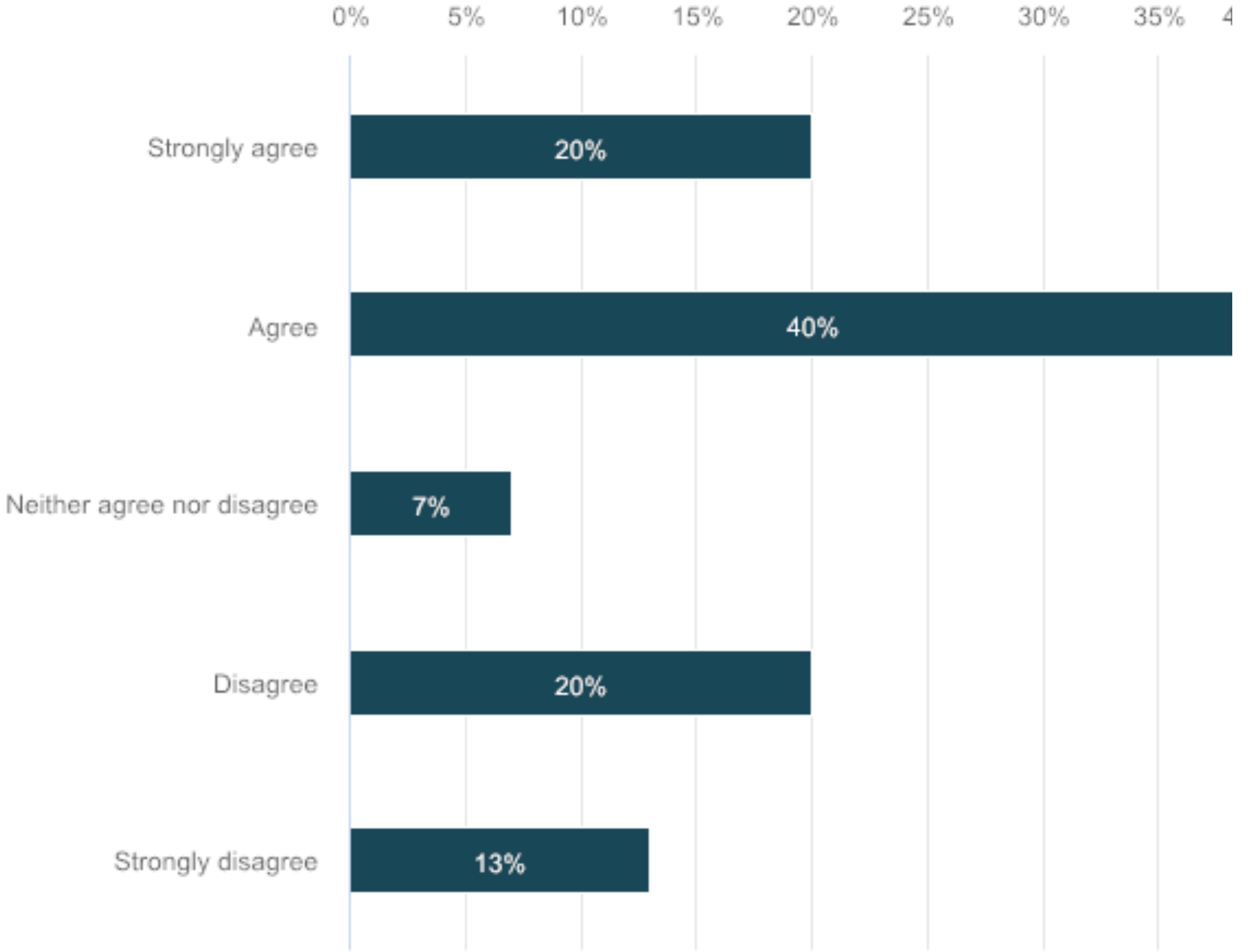

	n	Percent
Strongly agree	3	20%
Agree	6	40%
Neither agree nor disagree	1	6.67%
Disagree	3	20%
Strongly disagree	2	13.33%

21. Please add any comments here.

Number of respondents: 8

Responses
I don't think this is the safest approach because it could lead to underdosing of tumor and recurrence/death, which is unsafe. Perhaps change to "and the safest approach to minimize toxicity is to assume no recovery, but this may limit the dose of re-irradiation and decrease the effectiveness, leading to further recurrence.
Let's see if we can agree on some percentage. It definitely isn't zero.
Organ dependent - more evidence in spine.
This just doesn't fit with the evidence, which clearly suggests tissues recover over time. We just can't quantify it accurately
We have data on spinal cord recovery, so cannot agree that this is a universally valid statement
This statement is most likely true of pulmonary fibrosis as endpoint, but there is most likely some recovery with respect to pneumonitis
i tend to use 50% discount, but yes it is safest to assume no tissue recovery
Think we have to be pragmatic here though and although assuming no recovery is the safest approach it can be used to justify re-irradiation after detailed peer to peer discussion and with the patient's understanding and consent.

22. There is insufficient evidence to suggest volumetric cumulative dose constraints for the lung due to the changes in anatomy and function of the lung after an initial course of radiotherapy.

Number of respondents: 15

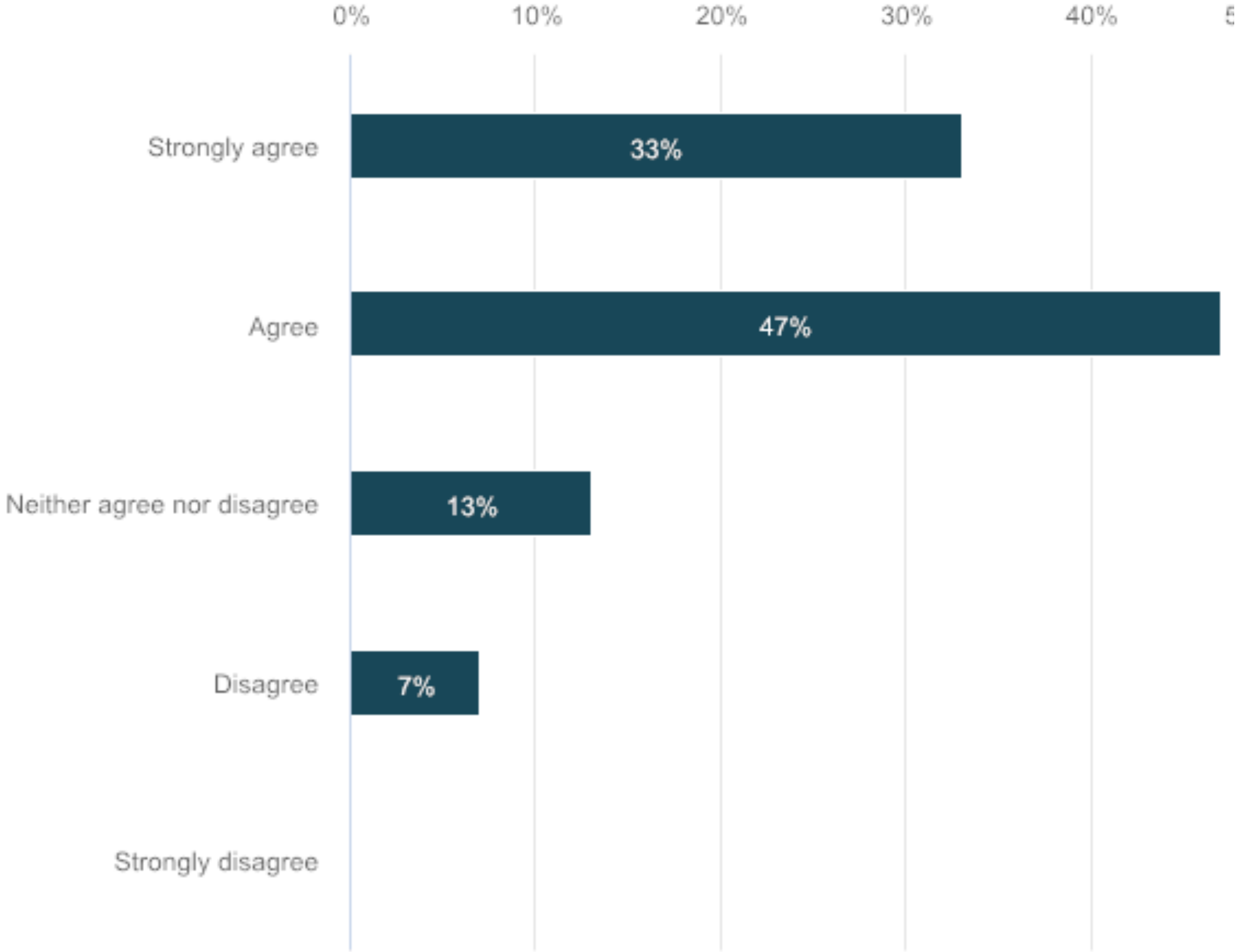

	n	Percent
Strongly agree	5	33.33%
Agree	7	46.67%
Neither agree nor disagree	2	13.33%
Disagree	1	6.67%
Strongly disagree	0	0%

23. Please add any comments here.

Number of respondents: 5

Responses
I think you can just drop the v5 and we'll be okay.
This is a very tough one!! Agree that there is insufficient evidence, but some safety parameters probably need to be suggested, albeit they won't be evidence based
I would remove:<br>"due to the changes in anatomy and function of the lung after an initial course of radiotherapy."<br>It is speculative
Lowered dose constraints after lobectomy and pneumonectomy were specified in the LUNGART study, but not based on evidence to my knowledge. Post radiation, any constraints would be speculative.
But would be reasonable to suggest adhering to standard dose constraints for the summed plan if possible.

24. For radical re-irradiation, the desirable cumulative maximum point dose (Dmax) constraint to the proximal bronchial tree is an EQD2 of 80Gy (using an a/b=3) although an EQD2 up to 105Gy is acceptable.

Number of respondents: 15

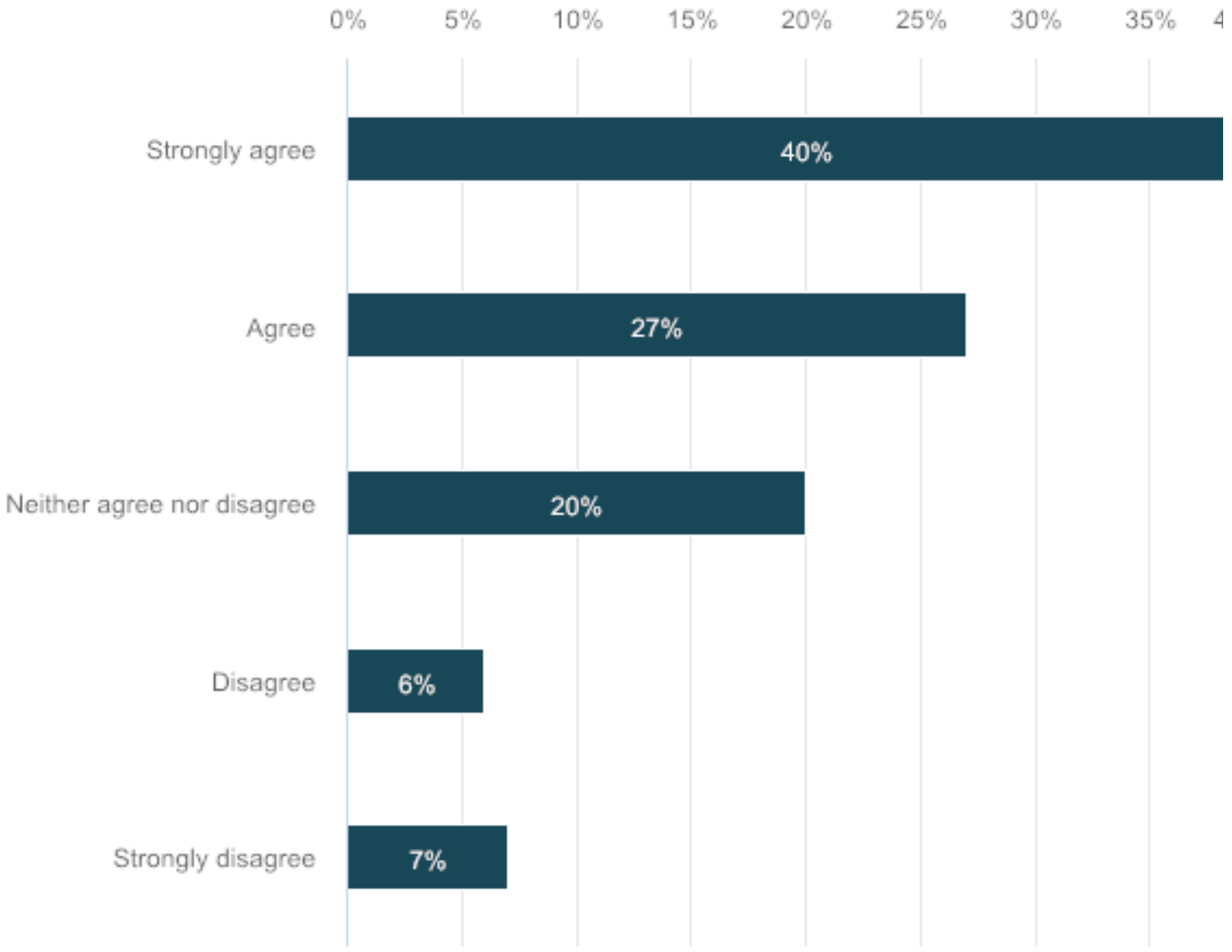

	n	Percent
Strongly agree	6	40%
Agree	4	26.66%
Neither agree nor disagree	3	20%
Disagree	1	6.67%
Strongly disagree	1	6.67%

25. Please add any comments here.

Number of respondents: 5

Responses
again, this is educated guesswork and cannot account for anatomic variations between cases
Not enough data.
I think it depends on whether the tumour involves the bronchial tree, as haemorrhage more likely in this situation.
We may consider higher doses in selected cases
If we assume no recovery of the bronchial tree over time, an EQD2 of 105 Gy may be excessive. Limited data from Cannon (primary hypofractionated radiotherapy) suggested a 5% severe complication rate for a Dmax of 83 Gy (J Clin Oncol 2013; 31:4343). I may have missed it, but where did the figure of 105 Gy come from?

26. For radical re-irradiation, the desirable cumulative maximum point dose constraint to the spinal cord is an EQD2 of 60Gy (using a/b=2), provided that the initial re-irradiation dose to the cord did not exceed 50Gy and the interval between treatments is greater than 6 months.

Number of respondents: 15

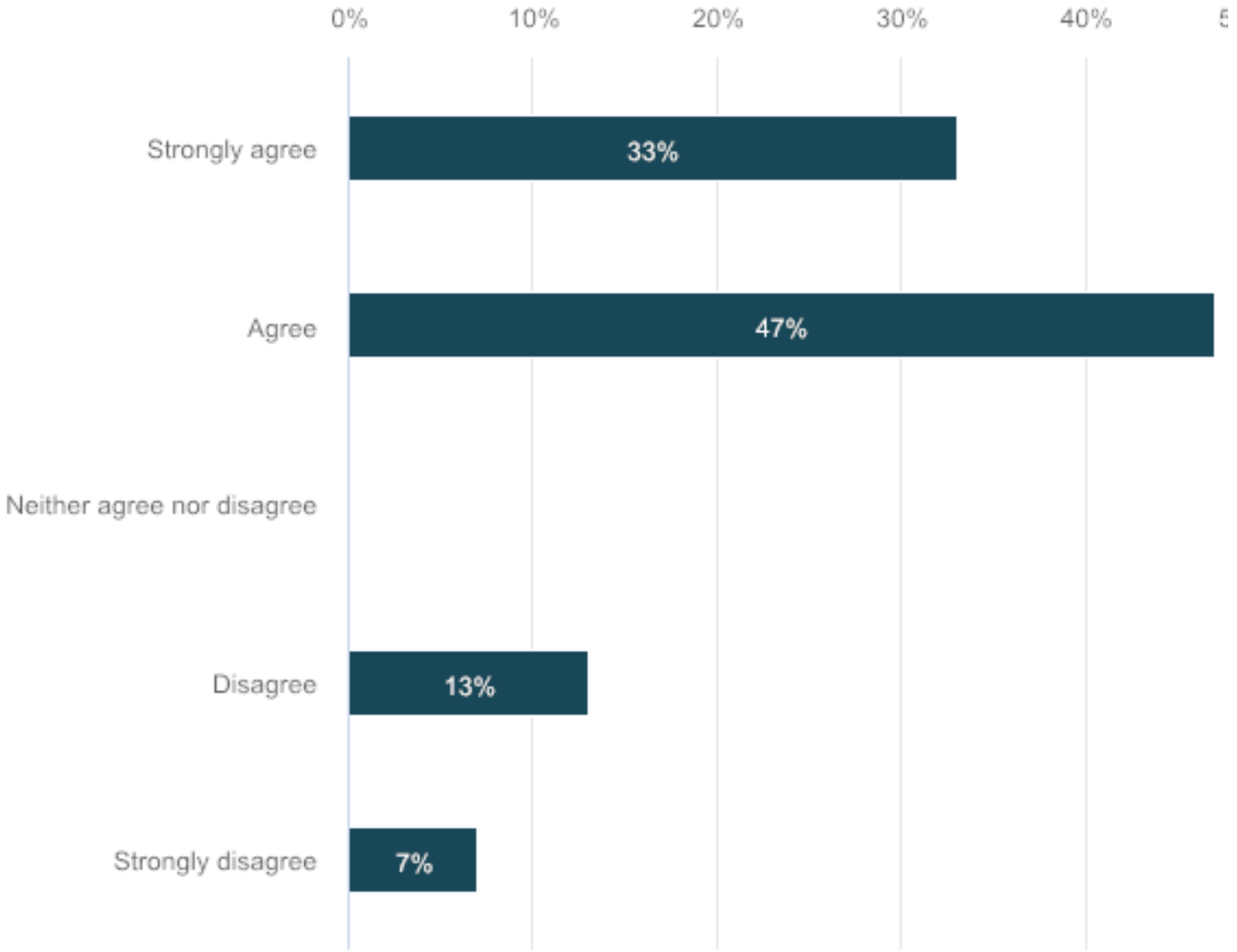

	n	Percent
Strongly agree	5	33.33%
Agree	7	46.67%
Neither agree nor disagree	0	0%
Disagree	2	13.33%
Strongly disagree	1	6.67%

27. Please add any comments here.

Number of respondents: 5

Responses
Can potentially go higher, depending on the interval between courses.
Will probably only be an issue with Pancaost tumours or T4 tunours involving spinal canal.
I don't really agree with the comment about initial RT dose of 50Gy or the interval<br>Instead of cord, a PRV would be preferred, or in practice the spinal canal/canal PRV would be used since it is unlikely that an MRI will be available to identify the cord itself, a PRV also allows mitigation of set-up/dosimetric uncertainty
i would use 0.5cc max dose for all rather than max dose point
You achieved consensus - i suggest reverting back to original language.

28. For radical re-irradiation, the desirable cumulative maximum dose (Dmax) constraint to the great vessels (the aorta and the pulmonary artery) is an EQD2 of 110Gy (a/b=3).

Number of respondents: 15

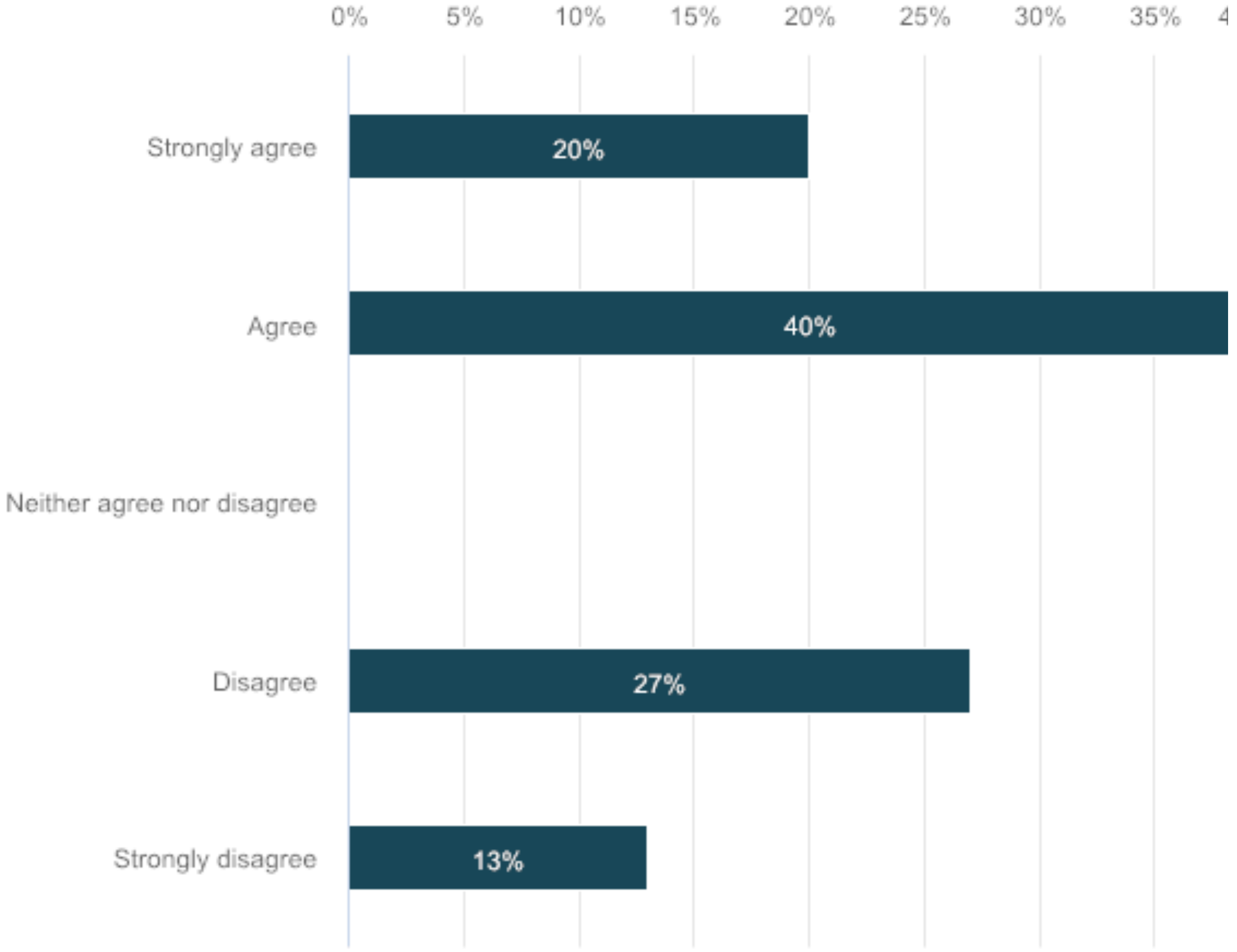

	n	Percent
Strongly agree	3	20%
Agree	6	40%
Neither agree nor disagree	0	0%
Disagree	4	26.67%
Strongly disagree	2	13.33%

29. Please add any comments here.

Number of respondents: 7

Responses
Not enough data.
Aorta can probably take more than this, but not an unreasonble constraint
In selected cases we may allow higher doses to one/other of these vessels
I do not think we have enough data. Aorta is apparently very resilient, but the pulmonary artery is very thin walled and implicated in episodes of exsanguination following endobronchial brachytherapy and SABR.
I would keep them separate
Can we add that 'although an EQD2 up to 120 Gy is acceptable'. This should be safe and acceptable since some constraints from central SBRT protocols in use already allow an EQD2 of 120 Gy.
The original assertion dividing aorta from pulmonary artery was better. The Aorta is very robust and likely can receive greater than 120Gy EQD2. however the pulmonary artery is very thin and is likely subject to injury, and perhaps some of the central lung toxicities can be attributed to this structure. I suggest reverting back to original language as you achieved 80% consensus

30. For radical re-irradiation, 4D-CT is the recommended simulation technique, and where there is nodal involvement, the 4D-CT should be fused with an IV contrast enhanced 3D-CT.

Number of respondents: 15

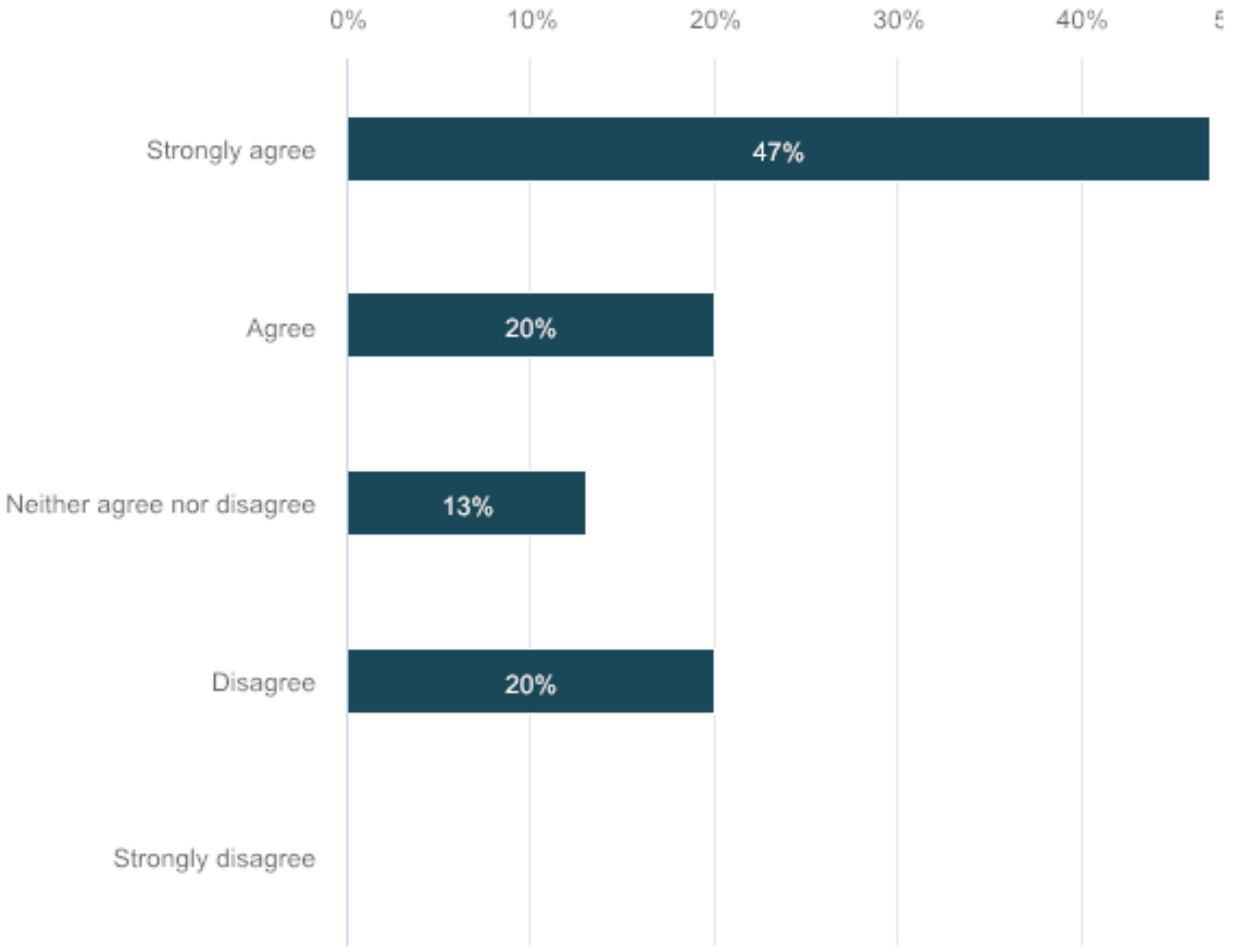

	n	Percent
Strongly agree	7	46.67%
Agree	3	20%
Neither agree nor disagree	2	13.33%
Disagree	3	20%
Strongly disagree	0	0%

31. Please add any comments here.

Number of respondents: 7

Responses
Fusion often doesn't work for nodes with diagnostic CTs, since the latter are done at end-inspiration and the former are free-breathing. I would just change to "may be fused"
The issue isn't just nodal involvement. It's central location where mediastinal structures may be difficult to distinguish from the primary tumor and over/under-contouring can occur.
Lack of evidence to support this, but 4D could help reduce overall volume treated, so this is sensible. Best to use highest qulaity techniques in this situation
Contrast images may not only be for nodes but central disease<br>Again, you could simplify by saying that contrast-enhanced 4DCT may be considered, +/- fusion of contrast-enhanced diagnostic CT, +/- fusion of PET/CT images
We would use PET/CT to aid in nodal definition, especially if there is significant post-radiation fibrosis resulting from the first course now obscuring active disease.
IV contrast needed to better define great vs, brachial plexus
use "registered" instead of "fused"

32. When contouring for conventionally fractionated radical re-irradiation, the recommended expansion from GTV to CTV is 5mm or greater (with normal structures, excepting lung, edited out of the CTV).

Number of respondents: 15

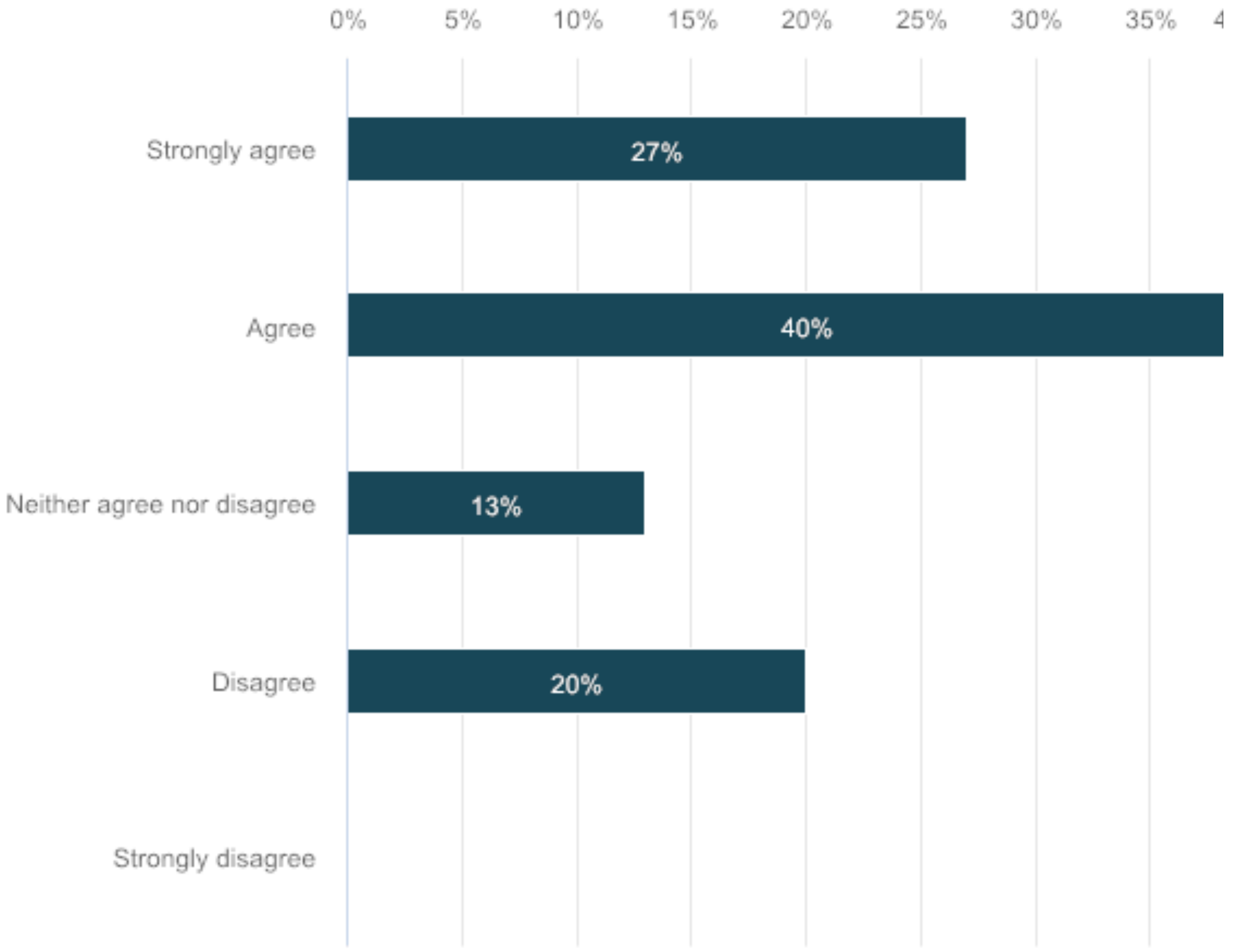

	n	Percent
Strongly agree	4	26.67%
Agree	6	40%
Neither agree nor disagree	2	13.33%
Disagree	3	20%
Strongly disagree	0	0%

33. Please add any comments here.

Number of respondents: 6

Responses
Normal structures should not be edited out unless they are barriers to spread.
Not enough data.
If we are using 4D CT then need to use ITV terminology. I would support 5mm expansion of ITV to PTV
The problem I have is that the concept of GTV contains no provision for the position of the GTV throughout ventilation as detected by 4D CT, which was not available at the time of ICRU 62 (1999). This consensus is not the place to have this discussion, and since treatment planning procedures is no different whether it is primary or re-irradiation, is this section important?
- 5mm is a reasonable margin but can be reduced depending on the situation
Suggest to add '...the recommended expansion from GTV to CTV is 5mm or greater (with normal structures, excepting lung, edited out of the CTV) but may be reduced at the clinician's discretion.'

34. Protons may have a role for re-irradiation and requires further evaluation in the context of a clinical trial.
Number of respondents: 15

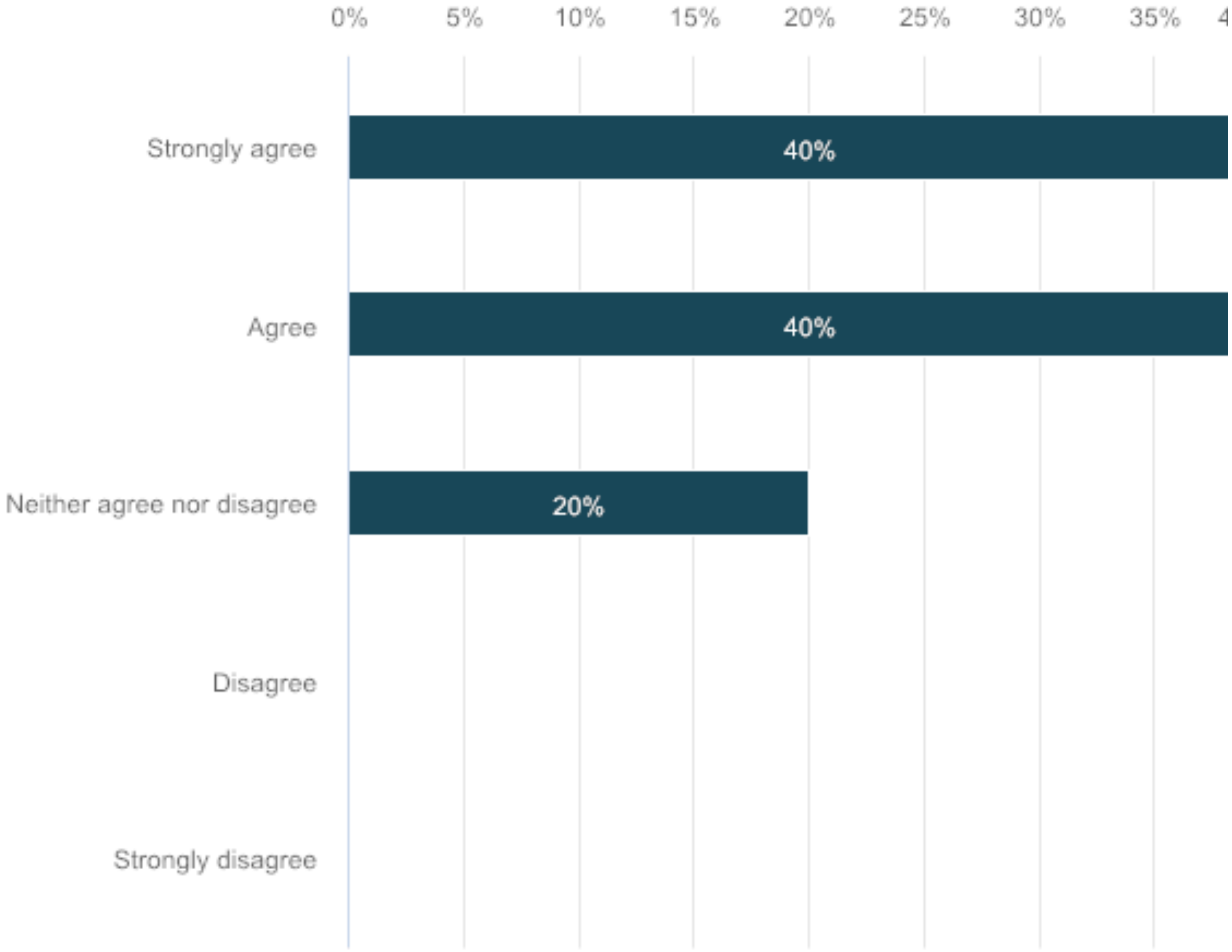

	n	Percent
Strongly agree	6	40%
Agree	6	40%
Neither agree nor disagree	3	20%
Disagree	0	0%
Strongly disagree	0	0%

35. Please add any comments here.

Number of respondents: 2

Responses
I don't think a randomised trial is needed. Manchester and London could set up a prospective datat collection clive<br>study
Suggest adding that "Current clinically available data do not show a strong signal for superiority of Protons compared to Photons"

36. Any dose and fractionation that can safely deliver a BED >100Gy to the tumour is acceptable for radical re-irradiation with SABR.

Number of respondents: 15

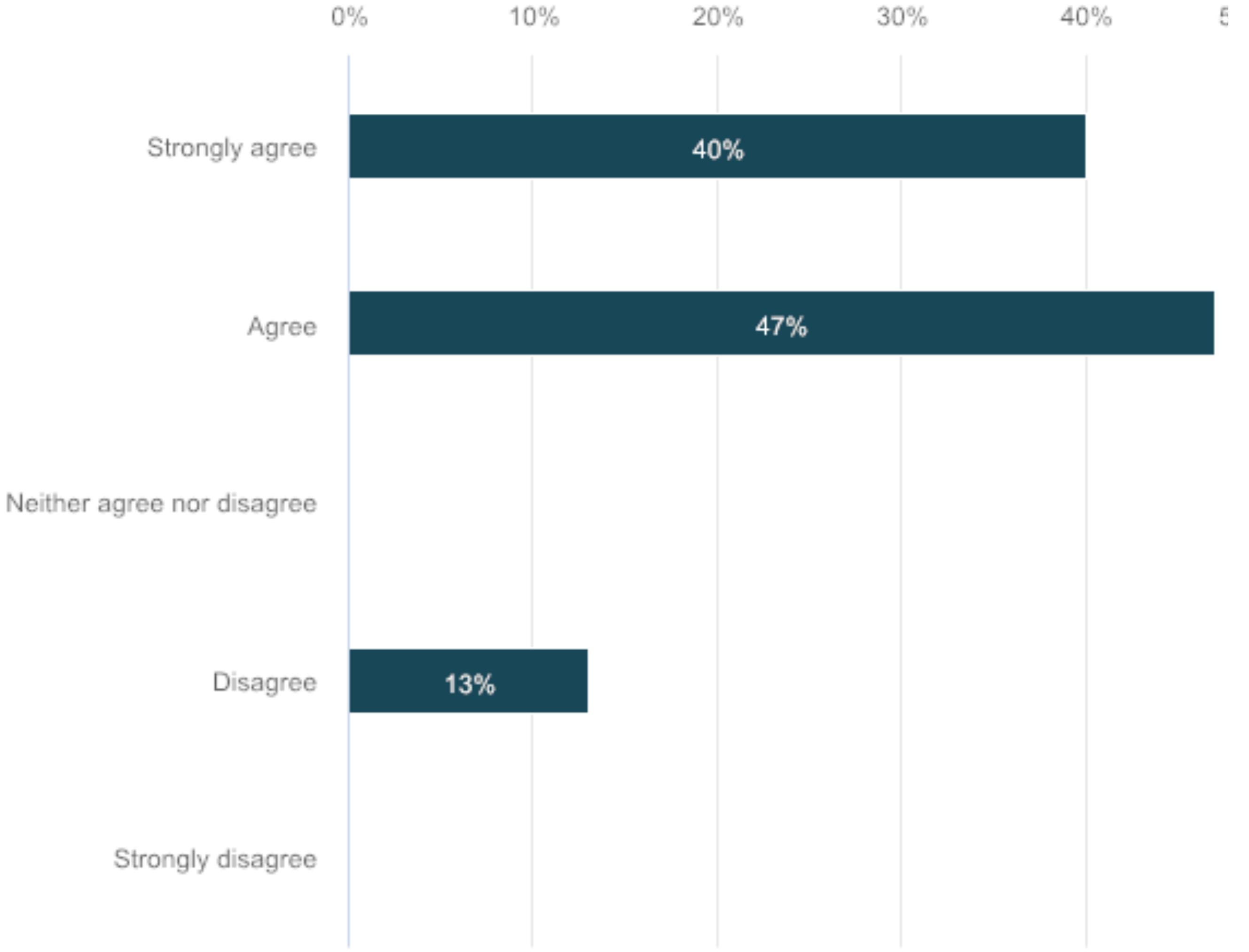

	n	Percent
Strongly agree	6	40%
Agree	7	46.67%
Neither agree nor disagree	0	0%
Disagree	2	13.33%
Strongly disagree	0	0%

37. Please add any comments here.
Number of respondents: 3

Responses
Individualisation sometimes needed
This will depend on whether SABR is appropriate, e.g. if high dose overlap on chest wall. My preference here is for more fractionation.
Some additional guidance in the manuscript may be useful to the reader. For example, using more fractionated schedules to re-irradiate larger lesions.

38. Radical re-irradiation should be performed using highly conformal radiotherapy techniques (e.g. VMAT, Tomotherapy, Cyberknife).
Number of respondents: 15

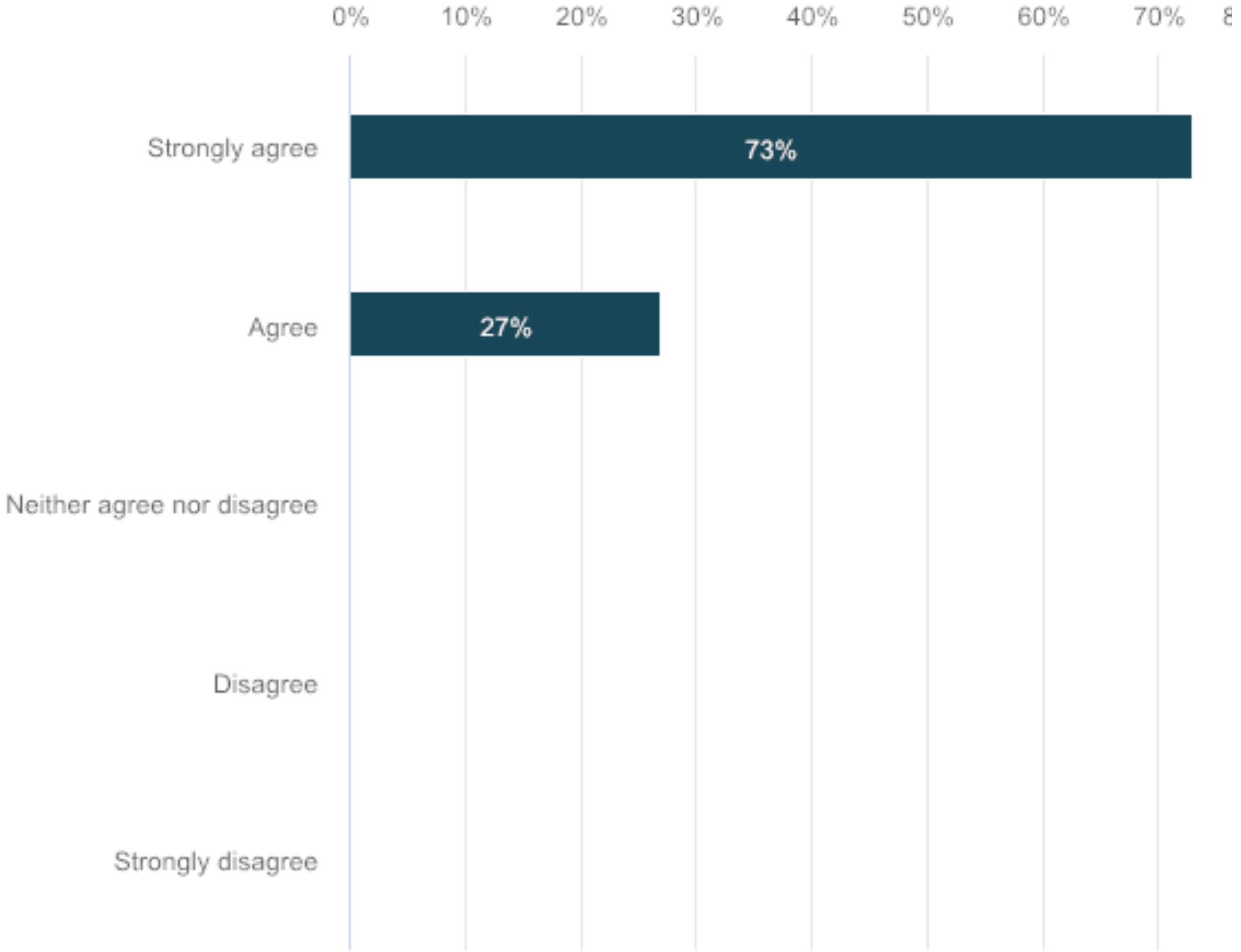

	n	Percent
Strongly agree	11	73.33%
Agree	4	26.67%
Neither agree nor disagree	0	0%
Disagree	0	0%
Strongly disagree	0	0%

39. Please add any comments here.

Number of respondents: 1

Responses
I would probably add the term "image guided"

Round 4

An international Delphi consensus on the use of radical thoracic re-irradiation and acceptable cumulative dose constraints - Final Round

Total number of respondents: 14

1. Please enter your identification number (found on the e-mail with the link to this survey)

Number of respondents: 14

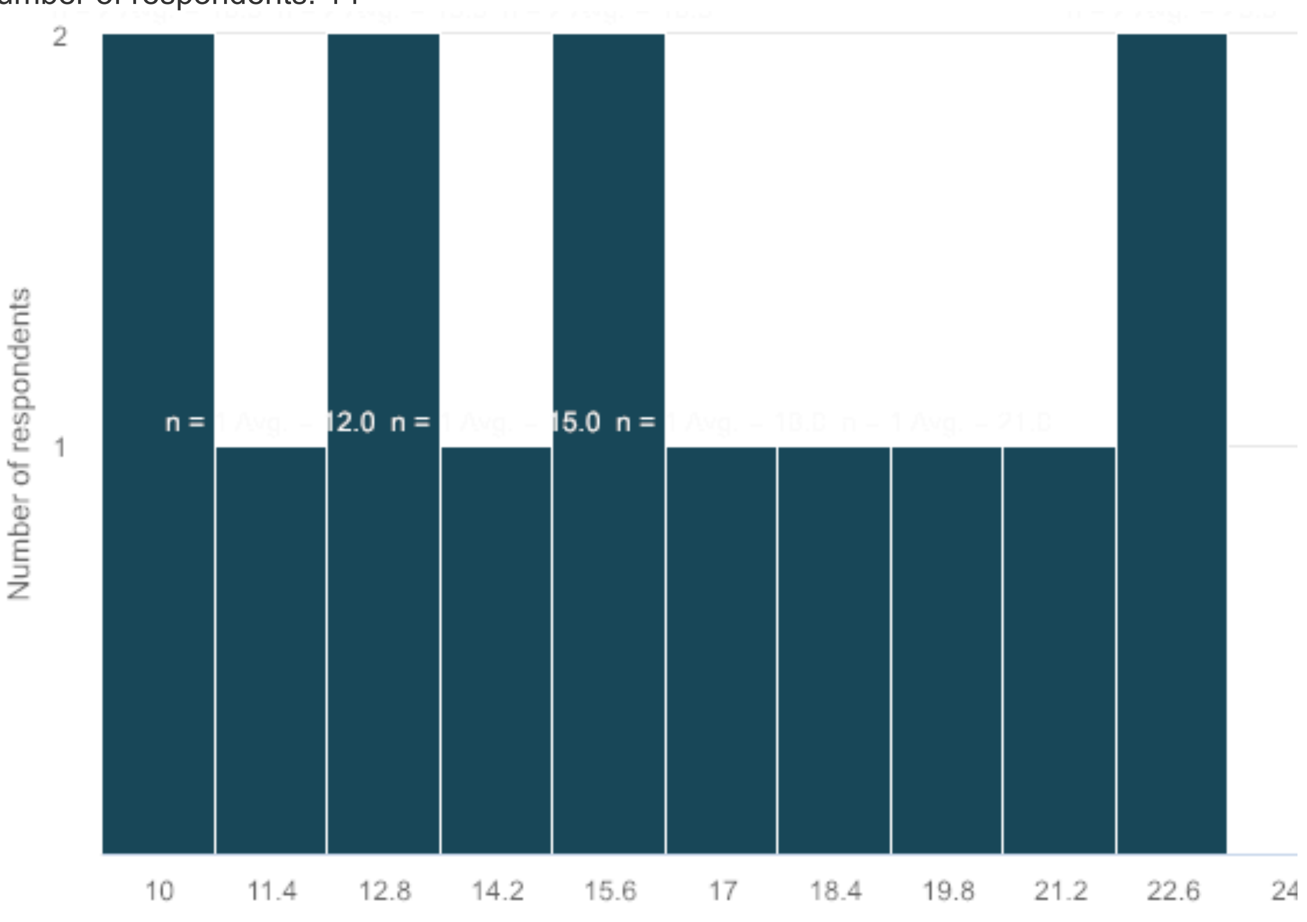

	Min value	Max value	Average	Median	Sum	Standard Deviation
	10	24	16.79	16.5	235	4.56

2. Which definition do you prefer?

Number of respondents: 14

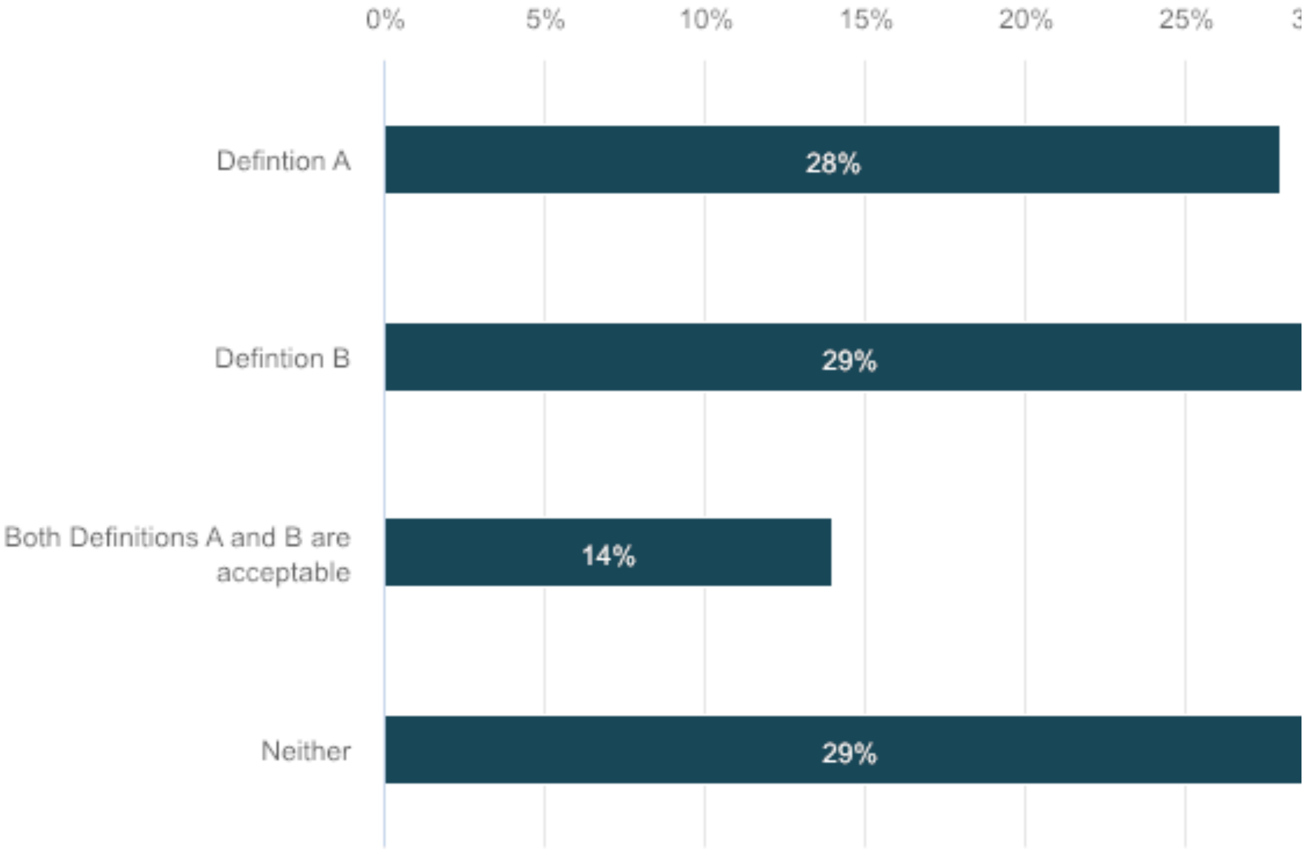

	n	Percent
Defintion A	4	28.57%
Defintion B	4	28.57%
Both Definitions A and B are acceptable	2	14.29%
Neither	4	28.57%

3. Please add any comments about either definition here:

Number of respondents: 11

Responses
For reasons stated above, agree that it's helpful to distinguish between the 2 types of re-irradiation. Definition B is more pragmatic and easier to apply in the clinic. Given that anatomic shifts can occur following first course of RT, the arbitrary cut-off of 1 cm or 25% isodose line also accounts for some of this uncertainty.
I don't think that treatment of a tumor in a different part of the lung should be called re-irradiation. It just confuses the issue.<br><br>I think a good place to start is 2A from the previous rounds and get feedback as to how to make that better.
I think both are reasonable - and clearly the team has put a lot of thought into these options. My feeling is that whatever is decided on, needs to be something that has practicality for the practicing RO.
having two definitions is appropriate and helps clarify the issues. I don't think it is possible to ever get a perfect definition but these two help distinguish well the concern of target vs. OAR
I like the idea of type I and type II very much, but this should be independent from the indication for radiotherapy<br><br>I would propose the following<br><br>Type I reirradiation: thoracic re-irradiation where a second dose of radiotherapy is given and the cumulative dose of both course of radiotherapy exceeds the dose constraint of a serial organ at risk from a single course without correction for tissue recovery.<br><br>Type II reirradiation: thoracic re-irradiation where a second dose of radiotherapy is given without relevant overlap of both radiotherapy doses but where cumulative doses to parallel organs at risk are associated with an increased risk of toxicity.
I would not limit to PTV volume/ or proximity to it.<br>I would just say re-irradiation = any radical XRT where dose to OARs would exceed constraints for single XRT course<br>Can call type B: repeat radiotherapy<br><br>restricting to having overlap/ 25% is not imp on its own and is v difficult to establish. What is more imp is dose to OARs<br><br>I agree with round 3 def
I think 25% is very arbitrary and therefore prefer the hard definitions of option A.<br><br>One minor edit; I would change "where there is any overlap between the initial treatment PTV and the new lesion" to " where there is any overlap between the initial treatment PTV and the new lesion PTV" - to acknowledge that it is

the PTV overlap of full dose between targets which is the issue.
Prefer definition B. However can live wiht definition A.
Prefer B<br>Couple modifications suggested below (in capitals):<br>Type I radical thoracic re-irradiation is where a second radical dose of radiotherapy is given to a NSCLC TUMOR (PRIMARY OR METASTASIS) located either within 1cm of the initial PTV of a previously radically irradiated thoracic tumour of any histology, AND/or having overlap with the 25% or greater prescription isodose line of the initial radiotherapy plan.<br><br>Type II radical thoracic re-irradiation is where a radical dose of radiotherapy is given to a NSCLC TUMOR (PRIMARY OR METASTASIS) located greater than 1cm outside of the initial PTV of a previously radically irradiated thoracic tumour of any histology and less than the 25% prescription isodose line of the initial radiotherapy plan.
Thank you for including the "neither" option. I'm not a fan of creating new definitions. Especially when the classification is driven by consensus and not necessarily evidence. I prefer pushing the group to find consensus and getting to an agreement that fosters common sense which will be more readily learned and remembered by our field. Such as "any overlap" of beams. It's actually good practice to dichotomize whether there is or isn't any overlap, and then figure out what to do from there. Since whenever the latter, folks can carry on with little anxiety about increased risks.
I definitely prefer definition A because it is simpler. It still suffers from the problem of not taking account of time and potential recovery of tissues, into the cumulative dose. The type II re-irradiation definition also suffers from the use of somewhat arbitrary parameters. However, this is completely understandable and unavoidable without hard data to guide us. Radiobiology can help, but this is more problematic with SBRT because the models are less reliable. This remains a very tricky topic, but I think we have something useful to build on here.
