# Supplementary material for: An International Expert Survey on the Indications and Practice of Radical Thoracic Reirradiation for Non-Small Cell Lung Cancer
Source: Adv Radiat Oncol. 2021 Jan 20;6(2):100653. doi: 10.1016/j.adro.2021.100653 (PMC8022147; doi:10.1016/j.adro.2021.100653)
Supplement: Appendix 2 [file mmc2.pdf]

Supplementary Data B

Respondent	Years of lung radiotherapy experience	Publications		Additional pertinent information
		Related to re-irradiation/recurrence/ Toxicity	Total lung cancer publications	
1	12	13	112	Currently participating in National German guidelines, ESTRO ACROP guidelines and ESR guidelines for lung cancer.
2	10	4	40	Fellow of the Royal Australian and New Zealand College of Radiologists, Fellow of the Royal College of Radiologists of London, Supporting Ambassador of the European Society for Therapeutic Radiology and Oncology (ESTRO), Founding Board Member of the Thoracic Oncology Group of Australia, Member of the Royal College of Physicians of London, Member of American Society for Therapeutic Radiology and Oncology (ASTRO), Member of the Trans-Tasmanian Radiation Oncology Group (TROG), Member of Cancer Trials Ireland (All-Ireland Cooperative Oncology Research Group), Member of British Thoracic Oncology Group (BTOG), Member of International Society for the Study of Lung Cancer (IASLC), Member of the Australian Lung Cancer Trials Group (ALTG).
3	15	2	84	Member of ESTRO and NVRO (Dutch Radiation Oncology Organization), Fellow of RCR (UK)
4	16	2	9	Member of the steering committee of BTOG, formerly a member of the UK SABR Consortium committee. On trials management group (TMG) for SOCCAR trial and ADSCaN trial, clinical lead for I-START arm of the study. Member of IASLC and ESTRO
5	23	4	97	Site leader of The Radiosurgery Society accreditation program: “Distinction in Radiosurgery”, member of ACCP guideline committee for the treatment of stage I and II NSCLC, member of the IASLC

				advanced radiation technology committee, member of the ASTRO lung radiotherapy guidelines committee, member of the RTOG advanced technology intergration committee, member of the RTOG and SWOG Lung cancer committees
6	10	2	77	Leader of lung cancer clinical trials and chair of a lung cancer clinical trials group (CAPRI), Member IASLC advanced radiation technology committee
7	7	3	77	Member of IASLC/ASTRO
8	34	3	270	Membership of Dutch Society Radiation Oncology, ESMO, ESTRO, IASLC
9	7	1	50	Led the initiation of local lung cancer re-irradiation service; now treating 20-30 re-RT cases/ year, Director of ESTRO lung cancer contouring workshops, developing proton lung cancer re-RT clinical study
10	33	2	330	Chair of the Australian Lung Cancer Guidelines, member of the IASLC Lung Cancer Staging Committee 1999-2018 which developed the 7th and 8th editions of TNM, and an Editor of the IASLC Thoracic Oncology textbook
11	9	1	67	Member of the IASLC and the ALTG (Australasian Lung Cancer Trials Group), chief investigator of thoracic multi-centre national randomised phase II trial through the TransTasman Radiation Oncology Group (TROG 13.01 SAFRON II) and am co-PI of ALTG NIVORAD clinical trial in non-small cell lung cancer
12	12	0	22	PI of phase III trial, grants reviewer for DOD and UK Cancer Research
13	15	1	20	Member of RCR/IASLC/ESTRO, member of National Cancer Research Institute (NCRI) Lung Group and founding member of UK SABR Consortium. Chief investigator for national and international trials (SABR-TOOTH, PREHABS, AVOIDING CARDIAC TOXICITY IN LUNG RADIOTHERAPY and PACIFIC-4).
14	11	5	90	Member of ASTRO, ASCO, and IASLC, chief investigator of

				radiotherapy clinical trials
15	8	1	13	Member of advanced radiation committee at IASLC, member of RCR, ASTRO, ASCO, ESMO, AACR, abstract reviewer for ASTRO, research interests include development of biomarkers for radioresistance and precision radiotherapy techniques

Types of re-irradiation articles:

	Number
Re-irradiation review articles	12
Re-irradiation retrospective studies	12
Re-irradiation dose constraints	3
Re-irradiation planning studies	1
Detection of recurrence	7
Radiotherapy toxicity	5
Other e.g. path studies	4
Total	44
