# Supplementary material for: An International Expert Survey on the Indications and Practice of Radical Thoracic Reirradiation for Non-Small Cell Lung Cancer
Source: Adv Radiat Oncol. 2021 Jan 20;6(2):100653. doi: 10.1016/j.adro.2021.100653 (PMC8022147; doi:10.1016/j.adro.2021.100653)
Supplement: Appendix 3 [file mmc3.pdf]

Supplementary Data B

For all tables: SA/A: Strongly agree/agree, N: Neutral, D/SD: Disagree/Strongly Disagree.

1. *Definition*

	**No consensus agreed**	SA/A	N	D/SD	Round of highest agreement	Median
Q1	Any dose of radical radiation for lung cancer, after initial radical radiotherapy to the thorax or surrounding tissues for any tumour histology, provided there is any overlap of previous dose in either the PTV or the OARs	67%	7%	26%	R2	Agree

2. Indications for radical re-irradiation

	**Consensus agreed**	SA/A	N	D/SD	Round of highest agreement	Median
Q2	Radical re-irradiation can be considered for suspected new lung primaries with minimal overlap with previous radiotherapy fields.	93%	7%	0%	R2	SA
Q3	Radical re-irradiation can be considered for lung tumours which develop new nodal disease after an initial course of radiotherapy only to the primary tumour (therefore minimal overlap).	100%	0%	0%	R2	SA
Q4	Radical re-irradiation can be considered where a lung tumour relapses locally (or develops a suspected second primary tumour with >50% overlap with the original primary tumour), but low overlap with serial structures in the thorax.	93%	0%	7%	R2	SA
Q5	Alternative treatments (e.g. systemic therapy) are preferred to radical re-irradiation to the primary lung cancer where the lung tumours have relapsed both locally and with widespread metastatic disease.	93%	7%	0%	R2	A
	**No consensus achieved**					
Q6	Where a lung cancer has relapsed both locally and with oligo-metastatic disease (less than 3 metastases, all mets treatable with radical radiotherapy), systemic treatment should be considered as the initial management, with subsequent radiotherapy to the primary +/- metastases to be considered in the context of a clinical trial.	73.3%	6.7%	20%	R3	A
Q7	Systemic therapies with a known risk of causing pneumonitis should not be combined with re-irradiation outside of a clinical trial.	53.3%	6.7%	40%	R3	A

3. Pre-treatment evaluation
3a. Patient eligibility

	**Consensus agreed**	SA/A	N	D/SD	Round of highest agreement	Median
Q8	In general, patients should have an ECOG performance status (PS) of 0 - 2 to be considered for radical dose re-irradiation, with exceptions being made for selected PS 3 patients (e.g. SABR re-irradiation, or PS 3 due to non-respiratory issues).	93%	0%	7%	R2	SA
Q9	Re-irradiation should be avoided in patients with interstitial lung disease.	86%	7%	7%	R2	SA
Q10	Re-irradiation should be performed cautiously with patients who developed grade 3 or higher toxicity with their initial radiation treatment.	86%	7%	7%	R2	A
Q11	Surgery should be considered in all appropriate patients being assessed for re-irradiation.	93%	0%	7%	R2	A
Q12	In locally advanced recurrent lung cancer, where there is an increased likelihood of response to immunotherapy (e.g. PD-L1 >50%), immunotherapy may be preferable to high-risk radical re-irradiation.	80%	0%	20%	R2	A
Q13	In locally advanced recurrent lung cancer, where there is an actionable mutation (e.g. EGFR mutation, ALK fusion), targeted treatment may be preferable to high-risk radical re-irradiation.	79%	7%	14%	R2	A
	**No consensus agreed**					
Q14	When re-irradiating patients (where the planned cumulative doses to the PTV exceed the initial prescription dose and/or where the cumulative dose to any OAR exceeds the dose constraints from a single course of radical treatment), the minimum interval between the first and second treatment is:				R3	>6m
	Unrestricted	Agree = 20%				
	>6 months	Agree = 73.3%				
	>12 months	Agree = 6.67%				

3b. Pre-treatment assessment

	**Consensus agreed**	SA/A	N	D/SD	Round of highest agreement	Median
Q15	Investigations prior to commencing radical re-irradiation are: Whole body PET-CT, CT chest + contrast, and CT/MRI brain.	>93%	-	-	R2	Essential
Q16	Consideration for biopsy must be made in a tumour board/multi-disciplinary team meeting before considering radical re-irradiation.	86.6%	6.7%	6.7%	R3	SA
Q17	Re-irradiation can be considered where the tumour board/multi-disciplinary team agrees that there is a high likelihood of cancer, but despite best efforts, histological confirmation of cancer is not possible.	86.6%	6.7%	6.7%	R3	SA
Q18	For conventionally fractionated re-irradiation, the clinician must consider re-treatment to have a positive risk/benefit ratio considering the current pulmonary function tests and the likely exposure of the lung to re-irradiation, with no minimum PFTs values applicable.	86.6%	6.7%	6.7%	R3	A
Q19	For re-irradiation with SABR, no minimum PFTs apply.	87%	0%	13%	R2	A

4. Treatment planning
4a Expected toxicities

	**Consensus agreed**	SA/A	N	D/SD	Round of highest agreement	Median
Q20	Projected grade 1-2 toxicities have minimal influence on the decision to offer re-irradiation, unless deemed significant after discussion with the patient.	87%	6%	7%	R3	SA
Q21	In general, the expected grade 5 toxicity rate of radical thoracic re-irradiation is less than 5%.	80%	7%	13%	R2	SA

4b Cumulative dose constraints

	Consensus agreed	SA/A	N	D/SD	Round of highest agreement	Median
Q22	There is insufficient evidence to suggest volumetric cumulative dose constraints for the lung due to the changes in anatomy and function of the lung after an initial course of radiotherapy.	80%	13.3%	6.7%	R3	A
Q23	For radical re-irradiation, the desirable cumulative maximum point dose constraint to the oesophagus is an EQD2 of 75Gy, although up to 100Gy is acceptable (using an a/b=3), with the volume of the oesophagus getting 55 Gray should be less than 35% (V55Gy<35%).	86%	7%	7%	R2	A
Q24	For radical re-irradiation, the desirable cumulative maximum point dose constraint to the spinal cord is an EQD2 of 60Gy (using a/b=2), with a maximum EQD2 of 67.5Gy (provided that the initial re-irradiation dose to the cord did not exceed 50Gy.	80%	13%	7%	R2	A
Q25	For radical re-irradiation, the desirable cumulative maximum dose (Dmax) constraint to the brachial plexus is an EQD2 of 80Gy (a/b=2) and an acceptable cumulative Dmax is 95Gy (if the interval between treatments is greater than 2 years).	80%	0%	20%	R2	A
Q26	For radical re-irradiation, the desirable cumulative maximum dose (Dmax) constraint to the aorta is an EQD2 of 120Gy (a/b=3). The desirable cumulative Dmax to the pulmonary artery is an EQD2 of 110Gy.	80%	0%	20%	R2	A
Q27	There is a lack of information to guide re-irradiation dose constraints for the skin and the heart, therefore the use of other guidelines (e.g. QUANTEC or SABR guidelines) and to keep the dose to these organs as low as reasonably achievable are recommended.	100%	0%	0%	R2	A
	**No consensus agreed**					
Q28	For radical re-irradiation, the desirable cumulative maximum point dose (Dmax) constraint to the proximal bronchial tree is an EQD2 of 80Gy (using an a/b=3) although an EQD2 up to 105Gy is acceptable.	66.7%	20%	13.3%	R3	A
Q29	For cumulative dose constraints, the amount of normal tissue recovery has limited evidence and therefore the safest approach is to assume no tissue recovery.	60%	6.7%	33.3%	R3	A

4c Re-irradiation simulation and contouring

	**Consensus agreed**	SA/A	N	D/SD	Round of highest agreement	Median
Q30	When combining initial and re-irradiation plans, either rigid or deformable dose registration are acceptable methods (although there are considerable uncertainties in either process and further investigation is warranted).	80%	6%	14%	R2	SA
Q31	18-FDG-PET-CT is recommended to aid tumour volume delineation.	86%	7%	7%	R2	SA
Q32	When contouring for conventionally fractionated radical re-irradiation, an acceptable minimum expansion from CTV to PTV is 5mm (or follow institutional guidelines where available).	86%	7%	7%	R2	A
Q33	PTV coverage can be compromised to achieve acceptable OAR doses.	80%	6%	14%	R2	SA
Q34	Radical re-irradiation should be performed using highly conformal radiotherapy techniques (e.g. VMAT, Tomotherapy, Cyberknife).	100%	0%	0%	R3	SA
Q35	SABR is the preferred re-irradiation technique where the tumour is not ultra-central, the tumour volume is small and there is minimal overlap with OARs.	87%	13%	0%	R2	SA
Q36	Protons may have a role for re-irradiation and requires further evaluation in the context of a clinical trial.	80%	20%	0%	R3	A
Q37	Acceptable doses for conventionally fractionated radical thoracic re-irradiation are 60Gy in 30 fractions or 55 Gray in 20 fractions once daily for non-small cell lung cancer.	93%	0%	7%	R2	A
Q38	Daily cone beam CT is recommended for treatment verification for conventionally fractionated re-irradiation.	100%	0%	0%	R2	SA
Q39	Any dose and fractionation that can safely deliver a BED >100Gy to the tumour is acceptable for radical re-irradiation with SABR.	86.7%	0%	13.3%	R3	A
Q40	Daily cone beam CT is recommended for treatment verification for SABR re-irradiation.	100%	0%	0%	R2	SA
	**No consensus agreed**					
Q41	For radical re-irradiation. IV contrast enhanced 4D-CT is the recommended simulation technique.	71.4%	7.1%	21.5%	R2	A
Q42	When contouring for conventionally fractionated radical re-irradiation, the recommended expansion from GTV to CTV is 5mm or greater (with normal structures, excepting lung, edited out of the CTV).	66.7%	13.3 %	20%	R3	A

5. Follow-up

	**Consensus agreed**	SA/A	N	D/SD	Round of highest agreement	Median
Q43	In patients who are fit for further treatment after radical re-irradiation, surveillance CT is recommended every 3 to 6 months for the first 2 years, then 6 to 12 monthly thereafter.	86%	7%	7%	R2	A
